# A shared agenda for gender and COVID-19 research: priorities based on broadening engagement in science

**DOI:** 10.1136/bmjgh-2022-011315

**Published:** 2023-05-22

**Authors:** Asha S. George, Claudia A. Lopes, Lavanya Vijayasingham, Mamothena Carol Mothupi, Ronald Musizvingoza, Gita Mishra, Jacqui Stevenson, Michelle Remme

**Affiliations:** 1 School of Public Health, University of the Western Cape Faculty of Community and Health Sciences, Cape Town, South Africa; 2 United Nations University International Institute for Global Health, Kuala Lumpur, Malaysia; 3 Faculty of Epidemiology and Population Health, London School of Hygiene & Tropical Medicine, London, UK; 4 School of Public Health, Centre for Longitudinal and Life Course Studies, University of Queensland, Brisbane, Queensland, Australia; 5 The Global Fund to Fights AIDS, Tuberculosis and Malaria, Geneva, Switzerland

**Keywords:** COVID-19, Health policy

## Abstract

While the acute and collective crisis from the pandemic is over, an estimated 2.5 million people died from COVID-19 in 2022, tens of millions suffer from long COVID and national economies still reel from multiple deprivations exacerbated by the pandemic. Sex and gender biases deeply mark these evolving experiences of COVID-19, impacting the quality of science and effectiveness of the responses deployed. To galvanise change by strengthening evidence-informed inclusion of sex and gender in COVID-19 practice, we led a virtual collaboration to articulate and prioritise gender and COVID-19 research needs. In addition to standard prioritisation surveys, feminist principles mindful of intersectional power dynamics underpinned how we reviewed research gaps, framed research questions and discussed emergent findings. The collaborative research agenda-setting exercise engaged over 900 participants primarily from low/middle-income countries in varied activities. The top 21 research questions included the importance of the needs of pregnant and lactating women and information systems that enable sex-disaggregated analysis. Gender and intersectional aspects to improving vaccine uptake, access to health services, measures against gender-based violence and integrating gender in health systems were also prioritised. These priorities are shaped by more inclusive ways of working, which are critical for global health as it faces further uncertainties in the aftermath of COVID-19. It remains imperative to address the basics in gender and health (sex-disaggregated data and sex-specific needs) and also advance transformational goals to advance gender justice across health and social policies, including those related to global research.

**Summary box**

- Integration of sex and gender in health research has long been highlighted, but continues to be inadequate, including in COVID-19 research.
- Although calls to decolonise global health research are more widely heard, the production of knowledge in global health is skewed against those who have multiple and overlapping forms of disadvantage.
- This research agenda setting collaboration offers practice-based learning for amplifying voices and perspectives from low/middle-income country stakeholders that are otherwise often disproportionately under-represented in global health research.
- The resulting gender and COVID-19 research agenda is wide ranging, inclusive of sex and gender equity in clinical trials, social and behavioural research, health service delivery reforms and gender mainstreaming in health systems and public governance.
- The gains made in fostering solidarity and collective aims through this research agenda are one example of the inclusive global partnerships needed to address complex future global health crises and to advance gender equality effectively.

## Introduction

Although we have emerged from the crisis phase of the pandemic, there were still an estimated 8.60 billion SARS-CoV-2 infections and 3.04 million COVID-19 deaths in 2022,^1^ tens of millions of people suffering from long COVID and sustained impacts on life expectancy in many countries.^2^ The rise in infections in China as it relaxed restrictions in early 2023 is a stark reminder that the pandemic is not behind us and there are likely to be many challenges ahead.

Almost every facet of the evolving nature of COVID-19 demonstrated the importance of sex and gender as key markers of difference and disadvantage interlaced with other forms of discrimination that must be reckoned with.^3–6^ At the same time, global knowledge production, even in gender and COVID-19, is highly skewed along multiple intersecting lines of privilege.^7^ This double challenge spurred the need for a prioritised research agenda to address gender and COVID-19 in ways that are more widely shared and owned (box 1). A collaboration was formed to provide a systematic and inclusive way to articulate sex, gender and COVID-19 needs to support policy-relevant and programming-relevant research. Our work demonstrates the strengths and challenges involved in applying feminist principles^8^ to make global health research processes more inclusive and effective.

Box 1 **Our feminist principles for this research prioritisation process**

- Co-creation and participatory design.
- Valuing different forms of knowledge, complexity, nuances, human experience and voice.
- Reflexive declarations on the identities, positions, and privileges of participants and mindfulness of power relations among participants.
- Consideration of gender dynamics faced by women, men and gender non-binary populations in an intersectional manner.
- Gender responsiveness of solutions, with the inclusion of gender transformation, gender equality and the redistribution of gender power dynamics as important outcomes.
- Disrupting the dominance of researchers based in high-income countries in synthesising evidence and defining research priorities, and advocating for researchers based in low/middle-income countries partnered with local and global stakeholders.

Our approach was also informed by previous research prioritisation processes. In a review of 165 exercises available on PubMed from 2001 to 2014, Child Health and Nutrition Research Initiative (CHNRI) (26%) and Delphi (24%) were the most commonly used, followed by consultations (19%), online surveys (8%), combined literature review with questionnaires (9%) and the James Lind Alliance method (8%).^9^ In another review of 116 WHO prioritisation exercises, expert consultation was the most commonly used approach (86%) (26% as only method, and while 52% of the total priorities described the use of literature review, all did so in combination with expert consultation).^10^ In low/middle-income countries (LMICs), common research prioritisation processes include the use of physical workshops or conference events, CHNRI and a combination of literature reviews, in-depth interviews and consultations.^11^

In essence, multiple methods and inputs are used in research prioritisation applying both consensus and metrics-based approaches and with varying degrees of formalisation. The consensus approach supports acceptability and buy-in, with the caveat that metrics-based ranking can prevent the dominance of select voices.^12^ Metrics-based approaches provide structure to the process of discussions and prioritisation. However, the use of surveys with selected criteria can remain complex to navigate, erases context-specific nuances and provides a false sense of objectivity. It also prioritises forms of knowledge that are accepted by mainstream science over other forms and ways of sharing knowledge.^8 13 14^ Strikingly, we could not find a single research prioritisation effort that explicitly followed feminist principles, although several addressed women’s health issues,^15^ and those related to sexual and gender-based violence have followed co-production principles.^16 17^ Based on past experience with these methodologies^18^ and WHO guidance,^12^ we developed our own approach (online supplemental file A), which emphasised distributed and consultative leadership and public engagement to compliment a prioritisation survey.

10.1136/bmjgh-2022-011315.supp1 Supplementary data

## Distributed and consultative leadership

**Figure 1:**
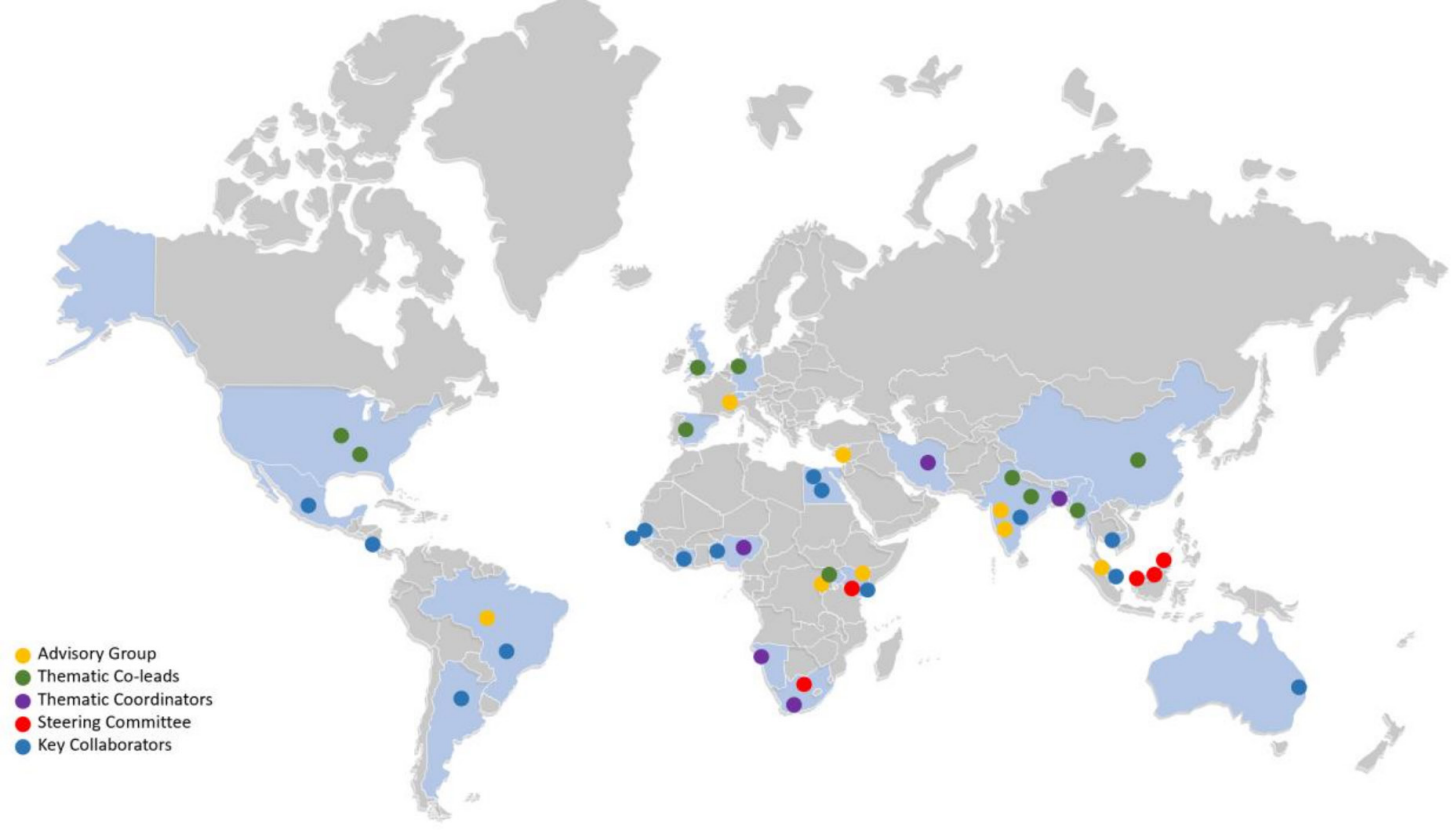
Geographical spread of leadership and key collaborators shaping a shared gender and COVID-19 research agenda.

While the scope of our work was global, in that it was inclusive of issues dealt with at global, regional, national, subnational or community contexts and not solely among international actors, we were also mindful of the inequalities that pervade global health research and the under-representation of researchers from LMIC contexts in global health processes. As a result, while a steering committee from the University of the Western Cape’s School of Public Health, South Africa, and the United Nations University International Institute for Global Health, Malaysia, co-convened the initiative, leadership guiding the collaboration in terms of advisory group members, thematic co-leads and group coordinators, as well as key collaborators, was openly invited and purposefully distributed across all regions of the world (figure 1).

In addition, we consulted with UN agencies, the gender and COVID-19 working group and the Sexual Violence Research Initiative to ensure that we were policy relevant, inclusive of key constituencies, and abreast of the latest practices in participatory forms of research prioritisation. To address any potential misunderstandings or abuses of power, a member of our advisory group served as an ombudsperson for the collaboration to ensure that anyone who had a query or complaint could access someone independent from the steering committee driving the process.

**Figure 2:**
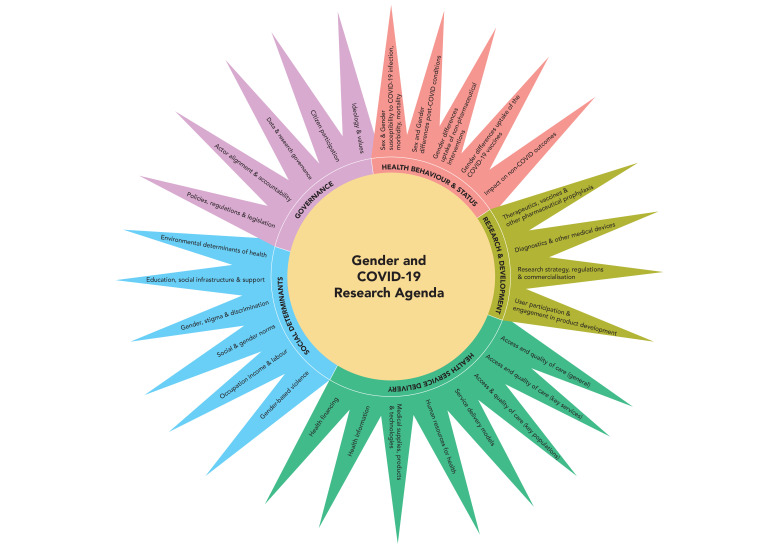
Thematic groups and subthemes shaping a shared gender and COVID-19 research agenda.

Given that gender dynamics cut across so many dimensions of the pandemic, the steering committee in consultation with the advisory group identified five themes to guide the prioritisation process: health behaviour and status, research and development, health service delivery, social determinants and health governance. Throughout the collaboration, participants engaged most with the theme of social determinants of health, a core foundation of gender and health research and policy. However, the collaboration also gave voice to feminist constituencies that are smaller in size but critical for COVID-19, namely those engaged in laboratory and clinical research and development. It therefore made more visible areas of feminist engagement in technical areas of health outside the realm of most laypeople. Creating these thematic groups therefore enabled thematic co-leads and coordinators to facilitate more cohesive and in-depth dialogue including those related to subthemes (figure 2), as well as support constituency building specific for these themes for better knowledge translation.

## Public engagement

**Figure 3:**
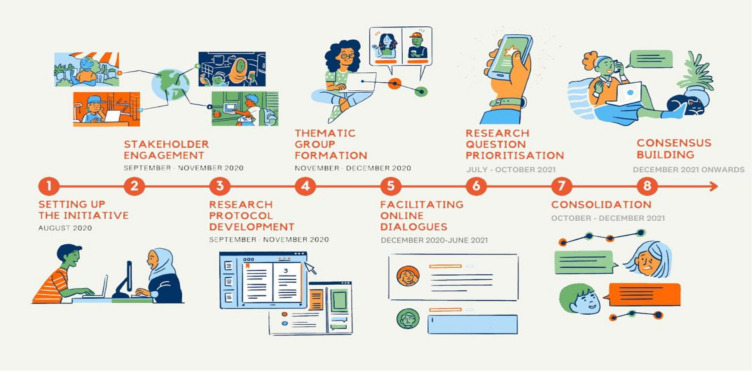
Trajectory of activities followed in developing shared gender and COVID-19 research priorities.

We started with stakeholder mapping and eliciting expressions of interest (figure 3). Participants were also iteratively recruited through the lifetime of the collaboration through open calls posted on social media, through emails sent to listservs and to individuals approached via steering committee members’ networks. We had no fixed targets or quotas, but there was an emphasis on ensuring LMIC representation and engagement by stakeholders other than researchers. Visual maps posted in real time the characteristics of stakeholders who participated voluntarily and we used this to galvanise further engagement. At every stage, representation of participants was tracked, reviewed and acted on with the aim of supporting participation from groups that were less well represented in the process, whether from specific regions or key audiences such as implementers, policymakers and donors.

In terms of linguistic inclusion, English remained the main language of the collaboration. However, the webinars, discussion board, prioritisation questionnaire and other materials and modes of communication were available in Arabic, Chinese, French, Spanish and Portuguese at different points during the 16 months of collaboration.

Finally, while emphasising inclusion, we did ask participants to disclose any links they had to organisations harmful to health to guard against vested interests. We also asked discussion board participants to voluntarily answer reflexive questions about their backgrounds and subjective viewpoints as a form of feminist community building.

A total of 504 stakeholders responded to the stakeholder mapping and call for expressions of interest to initially participate in the research agenda setting collaboration. Subsequently, more participants joined through various public online meetings and platforms (table 1). Over 400 participants took part in eight global meetings and 201 people participated in six regional consultations (table 2). These meetings had distinct purposes over the course of the agenda-setting exercise. They helped to finalise the research protocol and form the five thematic groups mentioned earlier (health behaviour and status, research and development, health service delivery, social determinants and health governance), facilitated online dialogues specific to key themes or regions, and built consensus on emerging research priorities. In particular, given the centralising assumptions made in global health knowledge processes,^7^ regional webinars were convened to enable regional constituency building and exchange, in addition to informing the global process. In addition to the online meetings, asynchronous forms of online participation were used (Google Docs, discussion board and surveys) as participants were engaging across diverse time zones. Discussion board participants included 441 stakeholders, with 159 contributing to online reports on the five thematic areas formed. Participants were invited to as many of the five thematic groups as they were interested in. All in all, over 1000 unique participants were logged in our master database through these varied engagements.

**Table 1:** Demographics of participants in the different activities of the gender and COVID-19 research agenda setting (column percentages)

	Expression of interest/stakeholder mapping (n=504)	Buzzboard participation (n=441)	Regional webinars (n=201)	Online survey (n=173)
Gender identity				
Women	72.8	74.6	72.2	72.2
Men	24.8	22.2	24.1	27.2
Non-binary	1.6	1.6	1.1	0.6
Regions as defined by WHO regions				
African	31.8	24.9	31.0	22.0
Pan American	11.2	11.5	15.0	20.8
Eastern Mediterranean	5.8	5.5	32.1	4.6
European	20.6	27.2	13.5	16.2
South-East Asian	17.0	19.4	3.6	19.1
Western Pacific	13.0	10.6	5.0	17.3
Country’s income				
High	29.1	35.5	26.4	37.0
Upper middle	20.0	20.7	34.3	23.7
Lower middle	36.1	34.1	33.6	31.8
Low	14.8	9.7	5.7	7.5
Organisational base				
NGO	40.8	24	28.9	23.1
University	40	40.1	36.84	40.5
Donor/government/multilateral	15.1	31.6	31.56	21.9
Independent	4.1	4.3	2.64	14.5

NGO, non-governmental organisation.

**Table 2:** Global meetings and regional consultations convened or presented by the gender and COVID-19 research agenda setting collaboration

Title	Convenor(s)	Type of meeting	Date	Processes (figure 3)	
Stakeholders Feedback Meeting on Research Protocol	School of Public Health-University of the Western Cape (SOPH-UWC), United Nations University International Institute of Global Health (UNU-IIGH)	Global meeting	4 November 2020	Research protocol development and validation of 5 thematic groups	Stakeholder engagement and knowledge translation
Launch of the Gender and COVID-19 Discussion Board	SOPH-UWC, UNU-IIGH	Global meeting	27 January 2021	Thematic group development, online dialogue	
Informal consultation on research on gender and COVID-19 in Latin America	Pan American Health Organisation, SOPH-UWC, UNU-IIGH	Regional consultation	25 May 2021	Online dialogue	
Consultation on gender and COVID-19: Research in the Middle East and North Africa	ABAAD (https://www.abaadmena.org/), WHO Eastern Mediterranean Regional Office, Arab Institute of Women-Lebanese American University, UN Women, UNU-IIGH	Regional consultation	6 June 2021	Online dialogue	
West and Central African Webinar on Gender and COVID-19	African Population and Health Research Center, International Research Development Center, SOPH-UWC, UNU-IIGH	Regional consultation	15 June 2021	Online dialogue	
Gender & COVID-19 elements of clinical trials, clinical characterization & mental health presented at COVID-19 in Africa Tele-Convening #4	African Academy of Science, African Union Development Agency, African Institute for Development Policy, UK Foreign, Commonwealth and Development Office, Cochrane Africa	Regional consultation	30 June 2021	Online dialogue	
Catalyzing feminist COVID-19 health responses across benches, beds, boardrooms and beyond at the Gender Equality Forum	SOPH-UWC, UNU-IIGH, National Center for Gender Equity and Reproductive Health, Mexico Ministry of Health, Development Alternatives with Women for a New Era, BBC Media Action, Realizing Sexual and Reproductive Justice, Gender Equality Working Group of the SDG 3 Gap: GAVI, GFF, GFATM, ILO, UNAIDS, UNDP, UNFPA, UNICEF, UNITAID, UN Women, World Bank, WFP and WHO	Global meeting	1 July 2021	Online dialogue	
Gender and Health Research Agenda Setting for COVID-19: Initial Results	SOPH-UWC, UNU-IIGH	Global meeting	1 September 2021	Research question prioritisation	
Gender and COVID-19 Vaccinations- The Asia Pacific context	UNU-IIGH, UN Women Asia Pacific, WHO South East Asia Regional Office & Western Pacific Regional Office, Gates Foundation, Third World Network	Regional consultation	21 October 2021	Consolidation	
Gender and Health Research: Agenda Setting for COVID-19: Update for Partners Meeting	Feminist People’s Vaccine	Global meeting	16 November 2021	Consolidation	
Gender and Health Research Agenda Setting for COVID-19/Presentation for Reinventing the Future: Cycle of Reflections South to South	Federal University of Pernambuco, Brazil & UWC, South Africa	Regional consultation	24 November 2021	Consolidation	
Launch Event: A Shared Gender & COVID-19 Research Agenda	SOPH-UWC, UNU-IIGH	Global meeting	9 December 2021	Consensus building	
A Shared Agenda for Gender & COVID-19 Research Final Results adapted for Panel on Envisioning a Post-COVID-19 Health System, International Conference on COVID-19	BRAC School of Public Health, Bangladesh Health Watch	Global meeting	21 January 2022	Consensus building	
A Shared Agenda for Gender & COVID-19 Research: Abbreviated Results for WHO Global Research and Innovation Forum	WHO Roadmap	Global meeting	24 February 2022	Consensus building	

GFATM, Global Fund to Fight AIDS, Tuberculosis and Malaria; GFF, Global Financing Facility for Women, Children and Adolescents; ILO, International Labour Organization; SDG, Sustainable Development Goal; UNAIDS, Joint United Nations Programme on HIV/AIDS; UNDP, United Nations Development Programme; UNFPA, United Nations Population Fund; WFP, World Food Programme.

The online discussion board was a unique feature of this research agenda setting methodology. It served to support community building and enable visibility of individuals and their contributions, as critical aspects of the virtual research prioritisation process, which otherwise may seem faceless. Various discussion board blog posts and information guides oriented participants about the origins, purposes and principles guiding the collaboration. We highlighted some discussion board responses to questions about research investment and regional engagement on Twitter as social tiles and videos to give voice and encourage a balanced participation of stakeholders (online supplemental file B). The discussion board also served as an archive for the collaboration with all documents (research protocol, thematic reports) available to members for review and comment.

10.1136/bmjgh-2022-011315.supp2 Supplementary data

Lastly, real-time reporting of survey results through interactive online dashboards also encouraged further engagement. Alongside updates on the discussion board and Twitter, these multiple and overlapping interactive virtual interfaces sought to sustain as much participation as possible with a view to rapidly feed back emerging priorities to participants and key decision-makers.

## Prioritisation survey

### Survey development

An evidence map of the literature on gender and COVID-19 was shared on the discussion board and each of the five thematic reports further focused on what was known and what further needed to be explored before framing research questions. Thematic reports (online supplemental files C–G) were drafted by thematic coordinators and co-leads with support from steering committee members based on a thematic analysis of discussion board and webinar inputs, expert knowledge of the theme, review of the literature and outreach to subject experts. Thematic reports and their corresponding questions were revised iteratively based on feedback received on various versions posted on the discussion board. The steering committee took all the questions proposed in these thematic reports and developed a final list of questions. In total, there were 214 research questions with the number per theme varying between 36 and 54 questions.

10.1136/bmjgh-2022-011315.supp3 Supplementary data

10.1136/bmjgh-2022-011315.supp4 Supplementary data

10.1136/bmjgh-2022-011315.supp5 Supplementary data

10.1136/bmjgh-2022-011315.supp6 Supplementary data

10.1136/bmjgh-2022-011315.supp7 Supplementary data

Criteria to prioritise the research questions were drawn from other research agenda-setting exercises^19^ and WHO guidance^12^ before being finalised with the advisory group. Research questions were rated according to: (a) public health benefit of answering the question; (b) the likeliness that answering the question will improve gender equality and empower all women and girls; and (c) urgency of answering the question.

The preliminary version of the online survey underwent extensive piloting and discussion with a group of 10 respondents and subject experts. Subsequently, the wording of instructions, criteria and response scales were further improved. The translated questionnaires underwent back-translation and review by language and subject matter experts to ensure translation equivalence. The questionnaire was designed in Alchemer software and included detailed instructions, demographic questions and research questions organised by themes and subthemes.

### Survey implementation

Six brief videos were developed: one to explain the overall process of the research prioritisation process, one to guide participants through the online research prioritisation survey and four featuring diverse stakeholders encouraging survey participation in their respective regions. Initially, unique invitations to participate in the survey were sent to those who previously engaged through the expression of interest/stakeholder mapping, webinars and discussion board. This was done to maintain the strong LMIC representation of participants established. Our previous experience was that initial open invitations and open links not supported by active facilitation and LMIC outreach were often predominantly responded to by those based in high-income countries.

The survey was open in English from the 1 July to 31 October 2021, and in Spanish, French and Portuguese from 9 September to 31 October 2021. At the last stage of survey recruitment, a general link to the questionnaires was disseminated openly through mailing lists and social media to reach as many respondents as possible.

Across all thematic surveys, 224 responses were received from 173 participants. Participants were invited to answer as many of the five thematic surveys developed as they were interested and available to. The response rate for completed questionnaires was 24.4% for 709 participants invited through a unique link. This increased to 39.2% when the denominator focuses on all 441 thematic group members who received the link. Participants were asked to score the research questions from high to low importance according to the three criteria and could answer any number of the thematic surveys made available to them. We detail the technical aspects of our survey analysis in online supplemental file H.

10.1136/bmjgh-2022-011315.supp8 Supplementary data

## Reflecting on the gender and COVID-19 research agenda results

Gender questions related to COVID-19 research and development, acceptance and uptake of vaccines, health service access and gender-based violence were prioritised relatively higher than many research questions belonging to health service delivery inputs, social determinants and governance themes. Nonetheless, top research questions were prioritised across each of the themes (table 3), demonstrating that while some questions were seen as more urgent, a comprehensive approach for addressing gender and intersectional needs to COVID-19 was valued. The gender issues prioritised, as further discussed below, are critical for COVID-19, but also for those concerned about pandemic preparedness and responses more broadly, as well as a post-COVID-19 future characterised by varied uncertainties that face global health.

**Table 3:** Gender and COVID-19 top research questions (n=21) across all thematic groups with adjusted means per criteria

Top research questions scored by criteria: public health (PH) (1 low–4 high), gender equality (GE) (1 not likely–4 highly likely), urgency (U) (1 long (3–5 years), 2 medium (1–2 years), 3 short (6 months))		PH	GE	U
Thematic group on health status and behaviour				
RQ27	**Acceptance and uptake of COVID-19 vaccines**: do gender differences in the trust, acceptance and uptake of COVID-19 vaccines vary across social categories (such as race, disability, migrant status, age, sexuality and pre-existing conditions)?	3.65	3.53	2.61
RQ26	**Acceptance and uptake of COVID-19 vaccines**: are there gender differences in the trust, acceptance and uptake of COVID-19 vaccines?	3.58	3.39	2.66
RQ14	**Post-COVID-19 conditions**: how do post-COVID-19 conditions affect pregnant and postpartum women, their pregnancies and breastfeeding children across various contexts?	3.57	3.50	2.34
RQ5	**COVID-19 infections, acute morbidity and mortality**: what are the infection, acute morbidity and mortality levels of COVID-19 among pregnant and postpartum women across various contexts?	3.54	3.35	2.40
RQ36	**Mental health and other NCDs**: what were the impact of COVID-19 measures on the mental health outcomes of women, men, girls, boys, LGBTQIA+ (Lesbian, Gay, Bisexual, Trans, Queer, Intersex, Asexual, plus) persons and gender-diverse persons?	3.54	3.39	2.19
Thematic group on research and development				
RQ40	**Participation and engagemen**t: how can pregnant and lactating females be ethically and safely included in phase 3 and 4 studies for COVID-19 R&D?	3.83	3.90	2.25
RQ33	**Regulation, funding and commercialisation**: in what ways are sex-related and gender-related variables integrated into national and global vaccine safety surveillance systems?	3.74	3.46	2.38
RQ8	**Therapeutics and vaccine-specific population outcomes**: do safety, efficacy and optimal dosing regime, and protective duration of the different COVID-19 vaccines differ in pregnant and lactating women, and their pregnancies and breastfeeding children?	3.71	3.68	2.48
RQ5	**Therapeutics and vaccine outcomes**: does safety, efficacy and optimal dosing regime of different therapeutic interventions for COVID-19 and post-COVID-19 conditions differ by sex, age and race?	3.65	3.40	2.46
RQ9	**Therapeutics and vaccine-specific population outcomes**: does safety, efficacy and optimal dosing regime of different therapeutic interventions for COVID-19 and post-COVID-19 conditions differ in pregnant and lactating women, and their breastfeeding children?	3.61	3.56	2.44
RQ39	**Participation and engagement**: what is the extent of the enrolment and participation of women in ongoing and completed COVID-19 clinical trials across various sites and countries?	3.59	3.59	2.08
RQ21	**Digital health**: how can digital health intervention algorithms used in the pandemic be built to correct for gender and race bias?	3.58	3.39	2.21
Thematic group on health service delivery				
RQ12	**Service delivery models**: how did health service delivery measures respond to the needs of pregnant women who tested positive for COVID-19?	3.64	3.37	2.50
RQ6	**Access**: to what extent, and how has, utilisation of quality sexual, reproductive and maternal health and violence against women and girls services changed because of COVID-19?	3.63	3.39	2.26
RQ4	**Access**: how has the prioritisation of COVID-19 services affected access to services for non-COVID-19 health conditions by gender and its intersection with other social categories in various contexts?	3.61	3.52	2.44
RQ1	**Access**: how do access and quality of services for COVID-19 differ by gender and its intersection with other social categories in various contexts?	3.61	3.38	2.49
RQ2	**Access**: what strategies were used to improve gender and other inequities in access and quality of care for COVID-19 services (testing, facility-based care, quarantine care, etc) and how effective were they?	3.61	3.47	2.32
RQ19	**Service delivery models**: what are the different service reorganisation models implemented to ensure continuity of sexual, reproductive and maternal health and violence against women and girls services during the pandemic, and how effective are they?	3.60	3.38	2.01
Thematic group on social determinants				
RQ1	**Gender-based violence**: how has the prevalence, incidence, severity and frequency of different types of gender-based violence (including online violence and child marriage) changed during the different phases of the COVID-19 pandemic?	3.68	3.37	2.43
RQ2	**Gender-based violence**: which women and girls facing intersecting forms of discrimination (including age, poverty, disability, sexuality, etc) are the most vulnerable to, and affected by, different types of gender-based violence during the pandemic?	3.60	3.39	2.33
RQ4	**Gender-based violence**: what are the determinants and pathways of increased gender-based violence in the context of COVID-19?	3.61	3.39	2.25
RQ7	**Gender-based violence**: what policies, programmes and interventions have been successful and most cost-effective in preventing gender-based violence during the pandemic and over the long term?	3.61	3.38	2.05
Thematic group on governance				
RQ33	**Data and research governance**: how can national statistical systems be supported to produce and use sex and gender data during COVID-19 and future pandemics?	3.67	3.34	2.07
RQ1	**Gender mainstreaming**: what do responsive and resilient health systems that address gender bias and advance gender equality look like?	3.66	3.36	2.07
RQ11	**Gender mainstreaming**: to what extent, and how, is gender considered in the current decision-making and learning processes for COVID-19?	3.61	3.36	2.47

NCDs, non-communicable diseases; R&D, research and development.

The research question with the highest score related to the participation of pregnant and lactating women in clinical trials. Questions about the effects of COVID-19 and access to services for pregnant and lactating women repeatedly were identified as top research questions across several themes. While structures such as the Task Force on Research Specific to Pregnant Women and Lactating Women have been established in the USA,^20^ a more global landscaping of similar approaches in other countries is needed, as well as a look into effective strategies for implementing such guidelines and the ethical issues involved.^21–25^ While the focus of this agenda setting was COVID-19, strengthening structures to track and guide inclusivity in clinical trials will improve the quality of science involved, contribute towards preparedness for future epidemics and improve health outcomes for all.

Participants also prioritised research questions that examine the effects of sex and gender for vaccine and therapeutics research and development. For such research to be possible, sex-disaggregated analysis in clinical trials, safety surveillance systems and basic health management information systems must be prioritised. A number of initiatives to address the under-reporting of sex and gender have been developed, including the Sex and Gender Equity in Research guidelines.^26 27^ Health journals including the *BMJ*, *Lancet* and *Nature* have endorsed this approach. Yet, only 4% of 4420 registered SARS-CoV-2/COVID-19 studies explicitly reported a plan to include sex/gender for analysis, and only 8 of the 45 COVID-19 randomised controlled trials with results published by December 2020 reported sex-disaggregated results or subgroup analyses.^28^ Focusing on COVID-19 vaccine trials, between December 2019 and April 2021, only 24% presented their main outcome data disaggregated by sex, and only 13% included any discussion of the implications of their study for women and men.^29^ When it comes to routine information systems, things are not much better. At the height of the pandemic, only 6 out of 200 countries ever reported sex-disaggregated data across the COVID-19 testing-to-outcome pathway, with none doing so consistently for an extended period of time.^30^

It is striking that the gender and COVID-19 research prioritisation process highlights how the basic elements of making science and services inclusive for pregnant and lactating women and making sex-disaggregated data available for basic monitoring have not been addressed. At the same time, research questions about frontier elements of global health were also prioritised as critical for gender and COVID-19, whether it be the mental health impacts of COVID-19 measures or about how digital health algorithms are corrected for gender and race bias. Finally, research that examines and addresses the power dynamics that frame the lived realities of those most marginalised were also highly valued. This includes evidence to understand and respond to gender-based violence in the context of COVID-19, and policy analysis of how to integrate gender concerns into COVID-19 responses.

A key strength of the collaboration was that it sought to comprehensively address gender and COVID-19 issues, creating consensus and building constituencies around cross-cutting themes, rather than falling into the dichotomous tensions that have marked the evolution of the gender and health community.^31–33^ As the pandemic evolved, certain topics rose in importance in unanticipated ways (vaccine equity, long COVID, etc) and the comprehensive cross-cutting thematic structure of the research agenda was able to dynamically respond accordingly. Nonetheless, we do think vaccine-related questions were more highly prioritised as vaccine rollout coincided with the timing of the prioritisation surveys.

Many of the research questions proposed and prioritised are largely descriptive in nature, trying to assess the extent of gendered experiences and impacts, particularly given the lack of sex-disaggregated reporting flagged as a priority. Only 7 of the 21 top research questions focused on assessing interventions or policies designed to address gender and COVID-19 inequalities. As noted by Rasanathan and Diaz, health equity research can only move forward if we move from describing inequalities, as important as that is, to research that builds an evidence base on how best to change such inequalities.^34^

Taken together, we reframe the top 21 priority research questions into seven key areas for gender and COVID-19 that must be included in established COVID-19 research and research platforms (box 2). These varied dimensions of a gender and health research agenda are imperative for COVID-19, but also as the world moves forward to face new pandemics and global uncertainties.

Box 2 **Key areas for increased investment based on a shared gender and COVID-19 research agenda**

- Needs of pregnant and lactating women and people.
- Sex and gender in vaccine and therapeutics research and development.
- Real-time research on vaccine acceptance, trust, confidence and uptake.
- Indirect and long-term impacts on health and well-being, including gender-based violence, mental health and other non-communicable diseases by sex and gender.
- Implementation research to design, evaluate and learn from gender-responsive policies, responses and adaptations in health service delivery that promote gender equality or mitigate gender inequalities.
- Research that supports multisectoral action to address gendered social determinants and consequences of COVID-19 on those most marginalised.
- Research that reveals and transforms the gender power dynamics in health system decision-making for COVID-19.

## Reflections on facilitating more inclusive research agenda setting processes

Several aspects require reflecting on with respect to the 16-month process undertaken to develop and prioritise a gender and COVID-19 research agenda shared more broadly across the diverse stakeholders that must drive it. With over 1000 unique participants, the collaboration consulted both established experts and welcomed contributions from many new participants to global health deliberations notably from LMIC contexts, broadening its potential impact. It is hard to verify the implications of this. While journal publications and WHO proceedings build a public record that is long lasting, readership of such outputs can be relatively small. It is hoped that through this consultative and inclusive process, a broader audience will remember their engagement, own the outputs more broadly and follow up in their local contexts. In other words, we aimed to support evidence-informed practice by giving voice and respecting the dignity of those involved.^35^

A key characteristic of the collaboration was that it explicitly used feminist principles to broaden participation in research prioritisation efforts and therefore invested heavily in varied engagement strategies that encouraged participation from stakeholders not usually included as participants in global health processes. We acknowledge that the practice of global health, even its language and classification systems, is fraught with power dynamics shaped by historical and contemporary political economies.^36^ Even with acknowledging these limitations in classifying global health stakeholders, we achieved substantial numerical inclusion, although with limitations. Some groups, such as those from low-income countries, and donors or multilateral organisations were harder to involve, despite targeted outreach efforts. Those from countries that control public access to the internet were also likely to be harder to reach through our online forms of engagement.

Moving beyond aggregate numbers, to quality of engagement, we were concerned that webinars were not an effective way of facilitating a global conversation for pragmatic reasons and due to our feminist and decolonial principles which acknowledged historical power imbalances in knowledge exchange. Yet, respondents logged on and spoke up at webinars even if with relatively little notice. Nonetheless, enthusiastic participation in contributing to shared documents or in webinars did not always result in corresponding participation in the thematic surveys. Similarly, while almost 500 people answered the expressions of interest and stakeholder mapping exercise or logged onto the discussion board, this did not mean they were all active participants. Continuous online engagement requires active facilitation and the generation of value for participants across the platforms created. Questions of purpose, expectations around participation from those who are less privileged and power dynamics inherent to research need constant revisiting.^37^ Most concerning was that as the research prioritisation efforts became more mainstream, fewer participants engaged. Only a subset completed the surveys, and despite our attempts at supporting broad and diverse authorship of resulting documents (thematic reports in online supplemental files), the resulting *BMJ* publications do not reflect the breadth of contributions made.

Significant time was invested by the steering committee in providing opportunities for diverse participants to voice their priorities and mentor the leadership and skills of less experienced participants from LMIC contexts. Weekly calls were held with thematic coordinators and co-leads to develop a common approach and understanding of the issues that were emerging. Many involved had little prior engagement with global collaborations of this kind and were supported to present in global webinars and included in extensive drafting and redrafting processes. The enthusiastic response from participants who were relatively unknown to established gender and health networks was particularly refreshing and key for strengthening a mass base to advance the agenda forward. Particularly, if one wants to strengthen feminist knowledge creation, exchange and practice in local contexts where it must be applied.^38^

Inclusion was also the result of constant innovation and investment in formats that were visually appealing and welcoming. Timelines were constantly reset to ensure participation and were only possible due to the high value given to inclusive processes, although at the cost of more elite and more expert-driven processes, which may have been more publication focused. While we did have a research protocol for the research agenda setting process (online supplemental file A), its actual operationalisation changed dynamically throughout the collaboration, as many planning assumptions were found to be invalid and unanticipated needs arose. The time spent on ensuring quality processes underpinning the collaboration, and its appreciation of different forms of knowledge and engagement, at times crowded out the technical oversight that was also required.

The total operating expenses for the online collaboration were not high, particularly compared with the cost of convening 20–30 people in person globally at least once or twice from across the world. However, the in-kind time costs by a core team who were funded to support the collaboration through a variety of budgets, and largely provided their inputs on top of an already full work load were very high. Similarly, thematic co-leads and all those who logged on to contribute to the webinars, discussion board and surveys all volunteered their time, which they could have spent otherwise on other activities.

While timelines were continuously extended to ensure outreach to under-represented groups, the team was also mindful of the urgency of delivering results given the acute impact and dynamic nature of COVID-19. We moved forward with thematic surveys with relatively large numbers of questions for each theme, on the assumption that we would have a second prioritisation survey to rank high-priority questions across themes. We realised mid-way that organising a second survey inclusively would take more time that we could not afford both in terms of urgency, and in terms of participant fatigue and team exhaustion. In hindsight, following a less democratic process, where a smaller group spent time narrowing down the research questions, might have enabled more survey respondents completing each of the thematic surveys, with more ability to discriminate between questions, in a more timely manner. Issues of democracy, justice and efficiency in knowledge creation^37 38^ amidst a pandemic that further increases social inequality^7^ are filled with more nuance than anticipated.

Another substantial challenge faced was that every team member was impacted personally by COVID-19 or other national crises. Team members, predominantly women, fell ill themselves, were working from home with children who were not able to attend school, were simultaneously supporting family members living under lockdown restrictions or experienced the loss of family members and colleagues. While privileged in many ways, the lived experience of team members and collaborators was also mirrored in the research agenda they were co-producing. As the direct experiences of the initial year of COVID-19 become more distant, it is critical to not lose sight of its gendered personal significance and the impact it continues to have on knowledge production.^39^

## Conclusions

Given the uncertainties faced during COVID-19 and the implications for the lives and livelihoods for all those involved, but particularly for those most marginalised, deliberative forms of knowledge creation are essential for overcoming the blind spots of policymaking.^40^ Efforts to broaden engagement of science to ensure its quality and responsiveness are all the more important for COVID-19, because it reflected and amplified historical and contemporary forms of inequality driven by colonial and corporate greed.^7^ Efforts at inclusion, if well facilitated, can aim to mitigate and address historical power relations, but not transform them in any one activity, or even in a series of activities over 16 months, but it can provide a basis for further change. This offers further justification for exploring new ways of convening and assessing research agenda-setting exercises with open, collaborative and feminist methodologies. It necessitates transformations in research guidelines, investments and platforms that influence not only research on the evolving pandemic but across future public health as a whole. Funding, resources and opportunities are needed to enable the further development and refinement of approaches and methodologies that advance decolonial and feminist practices. If not, scientific enquiry and solutions will remain inadequate for a large part of the population, and we will not realise the transformation needed in how health systems serve their populations to accelerate health and well-being for all during pandemic and non-pandemic times.

## Data Availability

Data are available upon reasonable request. All data relevant to the study are included in the article or uploaded as supplemental information. Data for quantitative survey may be available upon request from the second author. All other data have been uploaded as online supplemental files.
