# Supplementary material for: A shared agenda for gender and COVID-19 research: priorities based on broadening engagement in science
Source: BMJ Glob Health. 2023 May 22;8(5):e011315. doi: 10.1136/bmjgh-2022-011315 (PMC10230361; doi:10.1136/bmjgh-2022-011315)
Supplement: Supplementary data [file bmjgh-2022-011315supp001.pdf]

UNITED NATIONS UNIVERSITY
UNU-IIGH
International Institute for Global Health

Gender & Health Hub
Knowledge. Policy. Action.

Gender and COVID-19

Final Protocol

Collaborative health research agenda setting for evidence informed action and accountability

November 12, 2020

Developed by steering team:

Dr Lavanya Vijayasingham
Dr Claudia Lopes
Dr Michelle Remme
Prof Asha George

Contents

1. Aim of the document and summary of research prioritization process 5

2. Context and scope 6
- 2.1 Background and rationale 6
- 2.2 Definitions 7
- 2.3 Thematic foci 8
- 2.4 Timeframe for research priorities 8
- 2.5 Types of research questions 8
- 2.6 Intended beneficiaries and audience 8

3. Governance and team 9

4. The research agenda-setting process 10
- 4.1 Analytic perspective and design principles 10
- 4.2 Planning 11
- 4.3 Thematic research prioritisation 12
  - 4.3.1 Thematic group formation: the sign up (1 week) 13
  - 4.3.2 Group connection and reflexivity 13
  - 4.3.3 Group organization: division of roles and responsibilities within group, establishing ground rules and timelines (process) (1 week) 14
  - 4.3.4 Scope and context for each thematic foci (content) (2 weeks) Group data collection and interactions using online discussion boards 16
  - 4.3.5 Generation and clustering of thematic research questions 16
  - 4.3.6 Voting of thematic research questions 17
  - 4.3.7 Outputs and deliverables of thematic groups 17
- 4.4 Overall prioritisation across thematic groups 18

5. Final research agenda and dissemination 18
- 5.1 Format 18
- 5.2 Discussion meeting 18
- 5.3 Journal Series 18

6. Evaluation, success indicators and participant feedback 18

7. Impact 19

8. Funding 19

9. Conflicts of interest 19

Appendix for protocol 22

Appendix 1:
Crafting a Feminist and Decolonial Research Agenda-setting Process 23

Appendix 2:
Literature on research agenda-setting processes 29

Appendix 3:
Comparison of established methods for research agenda-setting in health: 35

Abbreviations and Acronyms

CSO	Civil society organization
FENSA	Framework of Engagement with Non-State Actors
GBV	Gender-based violence
IPV	Intimate partner violence
2SLGBTQI	Two spirit lesbian, gay, bisexual, transgender, queer or questioning and intersex
LMIC	Low- and middle-income country
M&E	Monitoring and evaluation
MoH	Ministry of Health
NCD	Non-communicable disease
NGO	Non-governmental organization
R&D	Research and development
SDGs	Sustainable Development Goals
SOPH UWC	School of Public Health, University of Western Cape
SRHR	Sexual and Reproductive Health and Rights
UHC	Universal health coverage
UNU-IIGH	United Nations University International Institute for Global Health.
WHO	World Health Organization

1. Aim of the document and summary of research prioritization process

This document seeks to outline the process for collaboratively undertaking health research prioritisation on gender and COVID-19. The format is guided by the REPRISE reporting guidelines for research prioritisation.[1]

The research agenda-setting process will be based on stakeholder opinion, with reference to emerging research on gender and COVID-19, as well as gender dynamics in past pandemics. The United Nations University's Institute of Global Health (UNU-IIGH) and the School of Public Health at the University of the Western Cape (SOPH UWC) jointly constitute the steering team leading the collaboration, which receives strategic input from an advisory group. The team aims to develop and facilitate the research agenda setting process in an inclusive and participatory manner. Figure 1 outlines the four phases of the process (i. planning, ii. thematic group scoping and prioritization, iii. overall prioritization, iv. Evaluation and impact).

Figure 1: Summary of process

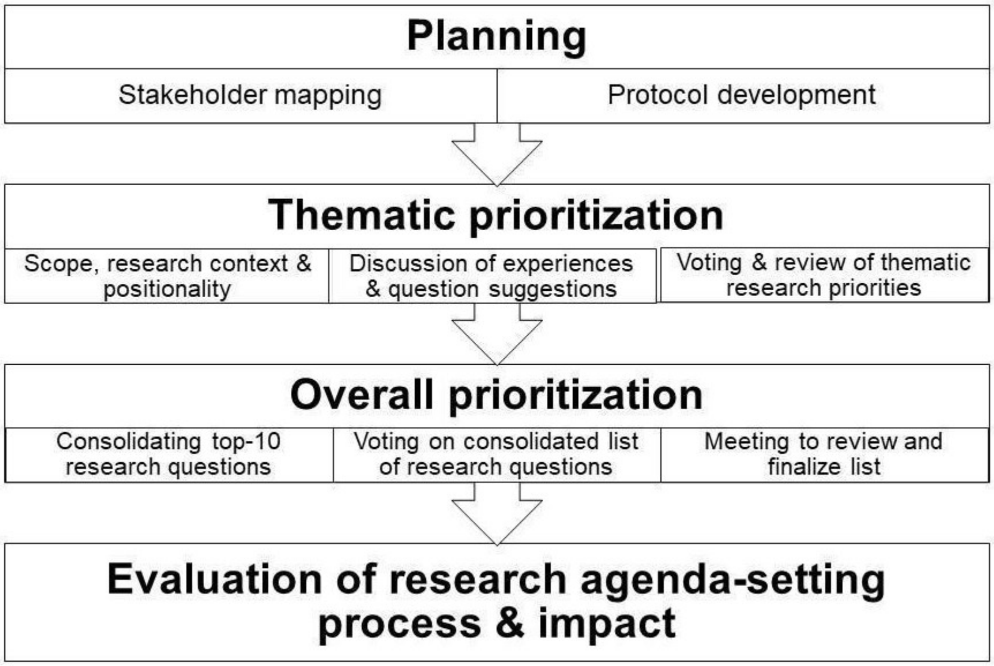

Initial planning includes targeted **stakeholder mapping and outreach** to ensure diverse stakeholder participation alongside **an open call to participate** and provide inputs along the research agenda setting process. **A draft protoco**l will be circulated for feedback among those who signed up through the open call and adapted based on collective inputs. An interpretive lens, and alignment with decolonized and feminist methodologies is proposed, including participatory design, and consideration of reflexive declarations on the identities, positions, privileges, and conflicts of interest of participants.

Given the complexity and breadth of gender and COVID-19 topics, **thematic sub-groups** will be established, to collectively identify and discuss the scope and research needs of each topic. Group deliberations mindful of power dynamics will be supported through an online discussion board to achieve convenient and asynchronous engagement, considering the multiple time zones and busy schedules of stakeholders, as well as the internet connectivity demands of other means of engagement. Roles and responsibilities will be assigned within each group to implement the process- based on a crowdsourcing design. Conflicts of interests (required) and reflexive disclosures (voluntary) will help to build trust and transparency in the process. A long list of questions will be generated from the thematic group discussions, and the group will score this list with criteria common to all the groups to establish the top 20 research priorities from each theme.

The 20 prioritized research questions from each thematic group will be combined and an overall **scoring** of this combined list will be undertaken. **The final research agenda**-will include sub-analysis of specific stakeholder groups to deepen understanding and facilitate contextualization of gender and COVID-19 research priorities. This report will be shared with participants and discussed at a **group meeting to validate the findings**, and plan further for the next phase-implementation of the research agenda. A post-exercise survey will be conducted to obtain **feedback** on participants' thoughts and experiences of the process. Evaluations of process will also be based on data collected from digital data such as forms, stakeholder outreach and engagement within discussion boards.

2. Context and scope

2.1 Background and rationale

Attention to the sex and gender implications of COVID-19 was galvanised early in the pandemic by a quickly mobilised constituency of researchers, policy makers and civil society actors concerned about gender and COVID-19 dynamics. However, real-time understanding and response was limited by extensive invisibility of the evolving situation, contributed by imperfect data systems, enduring vulnerability of marginalised groups, and siloed responses across sectors. This underscored the need for a research agenda-setting process to develop evidence informed action and accountability for gender and COVID-19 priorities.

There are several opportunities and limitations in terms of planning a research agenda-setting exercise on gender and COVID-19. First, prior exercises have included formative systematic scoping and review of literature to establish gaps and needs. At this stage, research on gender and COVID-19 is still emerging. Comprehensive formal needs-assessments from multiple stakeholders and reviews of literature is time-consuming. Therefore despite reference to the emerging literature, there will be a higher dependence on stakeholder opinions to inform

research prioritization. Second, the need for an agenda is time-sensitive, as the pandemic continues to evolve dynamically. Various stakeholders are already drawing attention to the gendered problems and suggesting actions that are informed from past pandemics. Researchers are already generating self-directed knowledge and seeking funding for pandemic-related research. As a result, these time constraints also shape the research agenda setting process. Lastly, the restrictions of face to face meetings, enables us to integrate the conversations and calls from the global health community in implementing new and adaptive ways of work that are primarily online and therefore are able to reach a broader range of stakeholders, notwithstanding the time constraints, competing commitments and internet challenges involved.

Given the urgency of guiding research investments and corresponding programming and policy responses to address COVID-19 by the health sector, we propose a collaborative health research agenda-setting exercise for gender and COVID-19, as part of UNU-IIGH's Gender and Health Policy Hub's inaugural scope of work.

This is a health research agenda-setting process, with focus on areas that the health sector has mandates and strengths in. Drawing on established frameworks on social determinants of health, health in all policies and 'whole of government, whole of society' approaches, we also recognize the non-health sector influences that contribute as social, economic, legal and commercial determinants of health. Therefore, it is crucial that while the research agenda is focused on health, it is generated through engagement with all critical sectors to collectively achieve greater health impacts as a society.

Building on current need and past experiences, the aims of the proposed research agenda-setting exercise are to:

- Harness current momentum on gender equality to support policy and programming-relevant research and accountability
- Identify a shared and prioritized research agenda and framework for evidence-informed action to address gender and intersectionality in the global health and intersectoral COVID-19 response
- Facilitate feminist solidarity in understanding, voice, and action from multiple communities of stakeholders

2.2 Definitions

Global: The scope of the work is global, in that it is inclusive of issues dealt with at global, regional, national, sub-national or community contexts and not solely among international actors. See appendix 1 for further elaboration.

Gender: Our conceptualization of gender includes women, men, and people of non-binary gender identities, with further focus on their intersectional status. See appendix 1 for further elaboration.

COVID-19: Coronaviruses are a large family of viruses which may cause illness in animals or humans. In humans, several coronaviruses are known to cause respiratory infections ranging from the common cold to more severe diseases. COVID-19 is the infectious disease caused by the most recently discovered coronavirus.

Health: We refer to WHO's longstanding definition of health as a '*state of complete physical, mental and social well-being and not merely the absence of disease or infirmity*'.

2.3 Thematic foci

Given the breadth of gender and COVID-19 health research, spanning biomedical and social research approaches, we propose five themes to enable sufficient depth in the deliberations, while still enabling engagement with a diverse group of stakeholders to ensure consideration of multiple perspectives and experiences. These five themes are discussed in the section below on thematic prioritisation (Section 4.3).

2.4 Timeframe for research priorities

Given the time sensitivity and dynamic nature of COVID-19, we propose a shortened time frame for the research questions being identified and prioritized:

- short-term priorities (6 months)
- medium-term priorities (1-2 years)
- longer-term priorities (3-5 years)

We also propose a plan to take stock of the implementation of the research agenda and to accordingly revisit or adapt the list in 2 years.

2.5 Types of research questions

Based on the thematic groups proposed, we suggest that cross-disciplinary research typologies across biomedical, clinical, public health, health systems, gender studies and social sciences be considered that are inclusive of conceptual and empirical research. Research questions should be answerable through diverse research methodologies, and question types that fit the needs of end users of evidence. We suggest the inclusion of descriptive, exploratory, influence, explanatory, predictive and emancipatory types of research questions,[2] that cover a focus on problems, cause and risk factors, solutions and interventions, implementation enablers and barriers, and evaluation of impact as appropriate.[3]

2.6 Intended beneficiaries and audience

The intended beneficiaries of the gender and COVID-19 health research prioritized through this initiative are all populations affected by COVID-19.

The audience responsible for implementing the research priorities include:

- researchers
- research funders
- programme implementers who are commissioning or using the research outputs
- product developers who are designing and testing interventions and diagnostic tools

3. Governance and team

Researchers from United Nations University International Institute for Global Health (UNU-IIGH) and the School of Public Health (SOPH) from the University of Western Cape (UWC), South Africa form the steering committee that plays an initiating and coordinating role to ensure cohesiveness, quality, and timely completion of the process. The team members have broad and diverse research and policy expertise on gender and health, particularly from low and middle income country (LMIC) contexts.

Table 2: Steering committee for gender and COVID-19 research agenda setting

UNU-IIGH, Malaysia	SOPH UWC, South Africa
• Dr. Lavanya Vijayasingham (Post-doctoral Fellow)<br>• Dr. Claudia Abreu Lopes (Research Fellow)<br>• Dr. Michelle Remme (Research Lead)	• Professor Asha George (SARCHI Chair in Health Systems, Complexity and Social Change)<br>• Ms. Mamothena Mothupi (PhD Candidate)

An advisory group with diverse disciplinary, regional and organizational backgrounds will act as a sounding board for the steering committee as it leads the process. This includes existing members of the gender and COVID-19 working group, as well as stakeholders engaged with gender and health more broadly.

The steering committee and advisory group collectively include expertise in gender and health based on clinical, public health, development studies, legal, health economics and anthropology disciplinary training. They have experience in primary and secondary research, evidence synthesis, prioritization and translation, policy making, program implementation and advocacy.

George AS, *et al. BMJ Global Health* 2023; 8:e011315. doi: 10.1136/bmjgh-2022-011315

Table 3: Advisory group for gender and COVID-19 research agenda setting

Name	Position and Organization	Country
Ms Jashodara Dasgupta	Chairperson and social researcher on gender, health and rights Sahayog	India
Mr Anthony Keedi	Masculinities technical advisor at ABAAD – Resource Center for Gender Equality	Lebanon
Dr Rose Oronje	Director, Public Policy & Communications, The African Institute for Development Policy (AFIDEP)	Kenya
Dr Rhona Mijumbi-Deve	Director, The Center for Rapid Evidence Synthesis (ACRES) at Makerere University	Uganda
Prof Deisy Ventura	Professor in Global Health Ethics at Public Health School, Universidade de São Paulo	Brazil
Dr Avni Amin, Dr Anjana Bushan & Dr Tasnim Azim	WHO and WHO SEARO	Global
Prof Pascale Allotey	Director, United Nations University International Institute for Global Health (UNU-IIGH)	Malaysia

4. The research agenda-setting process

4.1 Analytic perspective and design principles

Responding to recent calls for a feminist-oriented focus in global health[4] and decolonizing processes in global health, particularly from early career professionals,[5,6] we propose the use of feminist research values[4,7] a decolonizing focus, and gender analysis frameworks[8–10] to inform the question generation and research agenda-setting process.

Feminist research values include:[4,7]

- empowering all those that participate in the research process
- addressing power, hierarchical blocks, and actively seek out those who are excluded, address participation barriers
- the pursuit of social transformation
- reflections and disclosure of positionality and subjectivity
- sustained and equalizing relationships through inclusive participatory approaches
- valuing complexity, nuances, human experience, and voice
- translation of feminist values across cultures, contexts, institutions, and languages.

Drawing on this, we suggest an interpretive lens, that is inclusive of biomedical data and population statistics, as well as lived experience, multiple cultural and intersectional voices.[11] An interpretive approach,(as we propose) is more suitable for areas such as health systems and policy research, where there is uncertainty and complex pathways, where quantitative approaches alone may not be adequate.[12] Feminist methodologies also include quantitative

empirical methods, aligned to more positivist and numerical approaches of study and evaluation as those established in the biomedical and clinical domains.[7,13]

Concurrently, COVID-19 is exposing historically embedded structural inequalities both at population level, and in the systems of work within the global health community.[5,6] Even prior to the pandemic, there have been global movements to decolonize global health that call for disrupting implicit biased perceptions of LMICs, and shift leadership and visibility to LMIC organizations and people to initiate and drive global health projects.[5] Appendix 1 outlines the conceptual background and guiding principles further.

4.2 Planning

Initial planning steps include stakeholder mapping and protocol development.

4.2.1 Stakeholder enrollment and mapping

In line with principles of being inclusive, we have not established an inclusion criterion for stakeholders nor do we have a minimal value. Stakeholder engagement will be tracked throughout the phases so that adjustments can be made to ensure diversity. The purpose for this is to represent diverse ideas, voices and insights from multiple stakeholders. Figure 2 identifies the range of stakeholders we would like to engage in the priority-setting process.

Stakeholders will be invited through an open call, which will be monitored so that additional outreach can aim to ensure a diverse and globally representative group of stakeholders, by gender, region, organizational location and disciplinary background. A concept note explaining the initiative with an expression of interest survey form will be disseminated through email, social media, snowball referrals and targeted invitations. Participation by stakeholders in the research prioritization process is voluntary.

The call to enroll in the process will remain open until deliberations within the thematic groups begin. Until that time the outcomes of the open call will be updated weekly through the stakeholder map/dashboard on Tableau (stakeholder map link), which provides the latest information on number of stakeholders enrolled, gender, geographic, organizational background, level of work and type of expertise.

Figure 2: Stakeholders to engage with in defining gender and COVID-19 research agenda

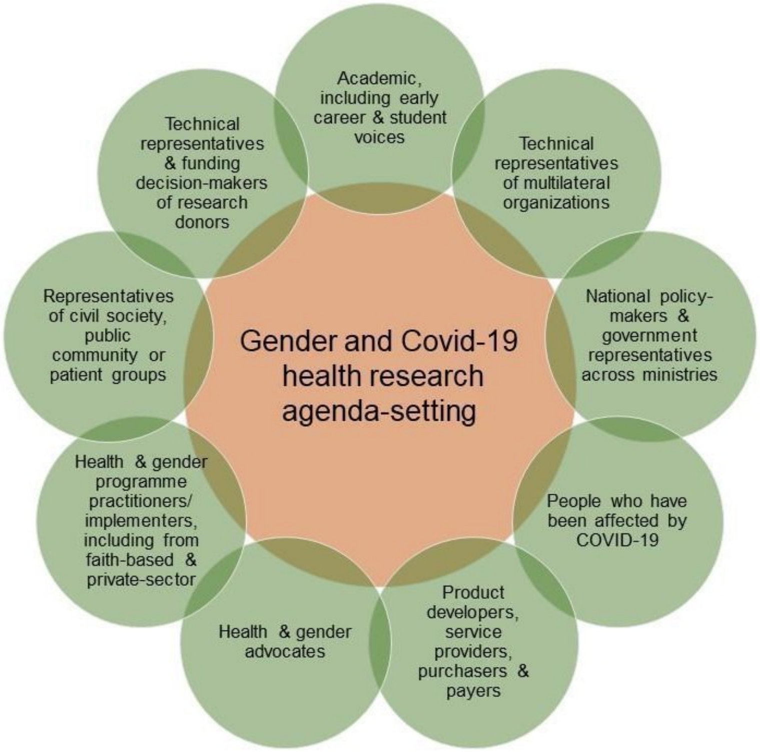

4.2.2 Protocol development

The steering committee will draft the protocol for research prioritization and seek feedback from the advisory group, as well as to all those who signed up to provide feedback on defining the process of research prioritization through the open call. The aim is to provide broad understanding of the research prioritization process and adapt it to fit stakeholder needs and feminist and decolonial perspectives. The team has considered and referenced prior research prioritization efforts and literature. (Appendix 2 &3

4.3 Thematic research prioritisation

As mentioned earlier, given the breadth of gender and COVID-19 health research, spanning biomedical and social research approaches, we propose five themes to enable sufficient depth in the deliberations, while still enabling engagement with a diverse group of stakeholders to ensure consideration of multiple perspectives and experiences. Thematic groups will be formed to aid consensus building and guard against fragmentation. The number of thematic groups formed also has logistical and management implications, with consequences for a timely completion of the agenda-setting process. Based on stakeholder responses received, the steering group will finalize the list of thematic areas.

The following five thematic groups are proposed that consider sex and gender in:

1. **Health knowledge, behaviour and status** of those directly and indirectly affected by COVID-19 across the lifecourse i.e. mental health, NCDs, SRHR, including health promotion and prevention interventions

2. **Health service delivery implications and** impacts of COVID-19, including utilization, supply chain, workforce, financing, digital health etc.

3. COVID-19 **therapeutic, diagnostic & digital,** ie biomedical, clinical, tech research and product development

4. **Structural determinants of gender dynamics** affecting or impacted by COVID-19, including gender-based, intimate partner violence and sexual harassment, social protection, employment, etc

5. Governance of COVID-19 health systems, including relationships with non-health **multi-sectoral, private, and political actors for health** (feminist movements, civil society, parliamentarians, private-sector etc.).

4.3.1 Thematic group formation: the sign up (1 week)

Given the workload, the voluntary nature of participation, and the timeliness of the initiative, interested participants will be invited to sign up for no more than two thematic groups, and volunteer for specific roles in only one group. Group membership should ideally include at least 50% global south representation. While signing up, they will also be asked to provide consent to use their insights as data and their willingness to use their full names and affiliations in the process (quotes will not be directly attributed to named individuals in reports).

They will also be asked to answer several questions on conflicts of interest. This includes disclosures on personal salaries, gratuities, and professional funding from commercial entities. This data will strengthen transparency and accountability on how the final agenda was set by the participants, and by whom. Conflicts of interest will be posted within group boards for members to consider. This section outlines conflicts of interest and the terms of engagement with stakeholders who are funded by or represent organizations that undermine global health interests.

4.3.2 Group connection and reflexivity

The discussion board will also provide an opportunity for group members to interact with and establish a rapport and serve to build relational understanding for the research agenda setting process.

As part of the ice-breaking and introduction to one another, participants will be asked to reflexively answer some questions, to share with their peers, (where willing), how their personal and professional identities, experiences etc. may influence their subjective insights, perceptions, and analytic lens during the research process. They will also be asked to reflect on the representation of their voice and stakeholder group, and how they will remain accountable to the groups they represent.

Reflexive disclosure are also an important step to:

- understand the extent of representation and types of voices at the discussion
- strengthen the rigor, accountability, transparency, and credibility of this process
- account for the diverse subjectivities influenced by the participants' position
- contextualize the relational differences between and within groups.

This activity is also a key part of the feminist methodology proposed in this research agenda setting process. Suggested questions for reflexive sharing and disclosure include:

- What personal and professional factors shape your understanding and engagement with the broad area of gender, health and COVID-19?

- What stakeholder group/voice do you represent and how are you accountable towards them in terms of research, decisions, actions and reporting/communication?

4.3.3 Group organization: division of roles and responsibilities within group, establishing ground rules and timelines (process) (1 week)

The steering team will receive nominations to co-lead the thematic groups, review them and submit recommendations for co-leads to the advisory group for confirmation. Balanced joint representation from academia and other stakeholder groups, as well as from the global south will be sought.

Given the broad range of issues and their complexity, initial scoping and prioritization will be done within thematic sub-groups.

A representative of the steering committee will be included in all thematic groups, and communicate ground rules, guidance on how to frame questions. A group SOP will be provided, that includes disagreement resolution or grievance procedures. Each thematic group will be guided by the research protocol and will be responsible to collectively identify and rank research priorities within the theme.

Roles that may be combined and that need to be assigned to group members include:

Roles	Tasks
**Co-leads**	Provide:<br>● Strategic input on the scope of the thematic area, dissemination, and implementation strategy<br><br>Drive:<br>● inclusive team discussions and rapport-building- assign roles and responsibilities, establish time commitment and availabilities<br>● completion of group/individual reflexive exercise<br>● moderate online discussions, manage conflicts or disruptive behaviours (if any)<br>● ensure deliverable outputs are completed in time, to highest quality possible<br>● Co-author research publication on thematic group research agenda<br><br>Identify:<br>● translation needs and work with members and steering committee to address them where possible
**Forum moderators**	● Ensure group engagement, respectful communication on discussion boards<br>● Probe discussions to obtain richer insights<br>● Answer member questions on boards
**Data analyst & report writers**	Conduct:<br>● rapid review to summarize research on the topic<br>● first-level thematic coding and categorize themes<br><br>Refine long-list of questions for in-meeting voting, based on suggestions on discussion board<br><br>Produce short brief on summary synthesis of emerging literature, forum discussions, (<3 pages) and long-list of suggested questions
**Translators**	Check automated language translations on page where required for group members.
**General members**	Participate and contribute to thematic group activities including:<br>● group discussions,<br>● reflexive exercise,<br>● generation of research questions on discussion board<br>● scoring in meeting and in survey

Given the workload and speed of output required, we propose to establish **one paid role within each thematic group** to ensure that all the tasks required and outputs from each thematic group are produced according to the timelines required. We suggest that the ideal candidate should be at post-doctoral level or higher, with established experience in qualitative research analysis and report writing. In line with the decolonial principles that guide this work, we suggest that preference should be given to candidates from LMICs, and from LMIC institutions. Their workload should take no more than 20 full-time days over 6-8 weeks, and payment represents a nominal contribution to the time costs of the coordinator, rather than a market based or daily rate.

UNU-IIGH will finalize terms of reference for this role with thematic groups and arrange the contract. We expect this to be a deliverable-based contract that will run for the course of the thematic group. (4-6 weeks)

4.3.4 Scope and context for each thematic foci (content) (2 weeks) Group data collection and interactions using online discussion boards

Asynchronous online platforms or discussion forums enable participants to provide their insights at their convenience, in a rich and structured manner, while also being able to engage with the responses from their peers.

Using the discussion board and online meeting, thematic groups will contribute to the following discussion threads to help further establish a common understanding among stakeholders, as well as outline the scope and context for the thematic research agenda:

- scope of thematic group- inclusion and exclusion of subject area
- research gaps and neglected areas
- the change or outcomes they would like for the generated evidence to achieve, and
- the required types of evidence, research design, questions and timelines that are required to support this change
- suggestions for how the research agenda can be implemented within their institutions, networks or collectively.

Outputs from these discussions will be posted on the discussion boards, so that steering committee members and other thematic group members can review to minimize overlap before questions are generated.

4.3.5 Generation and clustering of thematic research questions

Thematic group members will be invited to suggest questions that should be considered for the list of prioritized thematic group questions. When formulating questions, participants will be asked to consider the following domains:

- magnitude of health benefits,
- impact on intersectionality and inclusion of marginalized groups,
- ability to be adapted and implemented across diverse socio-cultural contexts

A designated thematic group member will collate and organize the suggested questions, removing duplicates, combining questions that are similar, and organizing questions by sub-themes.

4.3.6 Voting of thematic research questions

Once this list of thematic research questions is finalized, thematic group members will score the questions on a 10 point Likert scale, and will be asked to consider the following criteria when making their decisions:

- perceived need and magnitude of health benefits,
- consideration of gender-dynamics, human-rights, equity, intersectionality, and inclusion of marginalized groups
- likelihood of success to obtain funding and conduct within national contexts

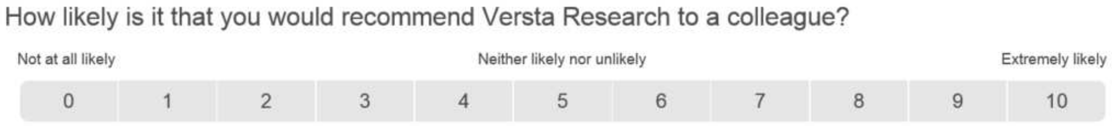

Analyses will review the distribution of responses and determine a reasonable threshold for identifying high priority questions. Thematic working groups will reconvene to discuss initial findings and the proposed list of prioritized research questions. Depending on the volume of prioritized research questions, a second round of scoring may be undertaken.

A maximum of the 10 highest priority questions from the thematic group will be collated to create a larger list of research priorities from all thematic groups.

4.3.7 Outputs and deliverables of thematic groups

Each thematic group will be expected to produce a report with the following contents based on the steps outlined above:

- Group members & brief discussion on their stakeholder type, and positionality
- Scope and context established
- Impact goals for the research, and research, design and translation process suggested
- Proposed research agenda implementation strategies and timeline
- Long list of questions
- List of top 10 questions

The coordinator or assigned member is responsible for developing this document as a live and working document during the course of the discussion that can be viewed by all members.

4.4 Overall prioritisation across thematic groups

The top-10 questions from each thematic group will be consolidated into a single form, for the entire group of participants to score-using a Likert scale, based on predetermined criteria (as used in thematic groups). This data will be analysed by WHO region and stakeholder type. The aim is to develop overall priorities across all the thematic priorities already identified. The analysis plan is iterative and will be adapted based on the group dynamics, data quality and emerging needs.

5. Final research agenda and dissemination

5.1 Format

The research agenda format proposed is a set of the combined top 10 research questions from each thematic group, with analysis of how different actors, regions and different thematic groups prioritized the combined list. The report will include a background section explaining the process that derived it, themes represented, criteria etc., strengths and weaknesses of the prioritization, and as mentioned analysis of prioritisation by a sub-group of participants.

5.2 Discussion meeting

After the analysis is complete, and the report is drafted, the steering team will organize a series of online calls/webinars, with an aim to discuss and validate the agenda, and obtain group consensus to establish and finalize the agenda. The draft will be shared with all participants prior to the meeting for their comments. Two to three meetings will be conducted to facilitate participation from different time zones and busy schedules. The report will be finalized, published, and disseminated after the meeting.

5.3 Journal Series

The steering team is also considering options to publish a journal series as an output of this activity. This will include authorship contributions from thematic groups.

6. Evaluation, success indicators and participant feedback

While there are no agreed metrics to assess the success of the agenda-setting process, there is a recently proposed evaluation framework (REPRISE) to assess the quality of the process and outputs.[1] We have been guided by this framework to create this protocol. We will also conduct a post-exercise survey to collect feedback on the process.

During the agenda-setting process, we will gather data produced by the digital platforms (forms, discussion boards) to assess the quality of the engagement with a breakdown by geography, demographics and type of organization. This digital data will be analysed to create ongoing indicators of success of the different stages of the consultation process. This information will allow us to adjust our engagement strategies throughout the process to ensure breadth of representation of multiple stakeholders.

7. Impact

Thematic group members will be asked to provide ideas and action plans on how to strengthen uptake and implementation of the research agenda. After the establishment of this agenda and action framework, there is merit to sustain the momentum and chart the progress and impact of implementation.

At this stage, we suggest:

- Webinars and small remote meetings to further broker dialogue between policy-makers, global actors, and academia to focus on the implementation and impact of the research, policy, and programming goals.

- A database of research that is supplemented by the creation of a series of policy briefs. This database could also be used as a means for accountability and tracking of implementation.

8. Funding

UNU-IIGH is non-aligned, independent and interdisciplinary. It is endowed by the Malaysian government and is also externally funded for specific activities. Funding from the Bill and Melinda Gates Foundation for the Gender and Health Hub supports this research prioritisation process.

Asha George is supported by the South African Research Chair's Initiative of the Department of Science and Technology and National Research Foundation of South Africa (Grant No 82769) and the South African Medical Research Council. Any opinion, finding and conclusion or recommendation expressed in this material is that of the author and the NRF does not accept any liability in this regard.

9. Conflicts of interest

UNU-IIGH acknowledges the need for public-private partnership[s and health intersectoralism in the pursuit of SDG 3, and is committed to engage with diverse global health actors involved in health research, advocacy, policy, and programming, and to advance public-private partnerships in health. Nevertheless, we are also guided by WHO's FENSA guidelines in the engagement with private sector actors in global health.

We consider conflicts in interest from corporate, advocacy, philanthropic and research organizations that have links to, and funding from corporate parent organizations, that:

- sell products that directly and adversely impact human health, and
- engage in neoliberal practices or corporate influence that undermine global health messaging, policies, and practices,
- benefit from ill health

We will not accept resources from these organizations but acknowledge that there are circumstances in which engagement can be deemed necessary for the advancement of global health research and policy. In this event, we will defer to the advice of the UNU legal team and determine decisions on a case by case basis. Engagement can include activities such as their

attendance of presentations, dialogues or meetings, technical collaborations, and involvement in research process and evidence generation.

UNU-reserves the right to exclude organizations and interactions that are perceived to undermine organizational and global health interests. Industries with the most obvious conflicts in interest include:

- Tobacco
- Alcohol
- Weapons
- Gambling
- Sugary drinks
- Fast-food

References

1 Tong A, Synnot A, Crowe S, *et al*. Reporting guideline for priority setting of health research (REPRISE). *BMC Med Res Methodol* 2019; **19**: 243.

2 George AS, Campbell J, Ghaffar A, *et al*. Advancing the science behind human resources for health: highlights from the Health Policy and Systems Research Reader on Human Resources for Health. *Hum Resour Health* 2018; **16**: 35.

3 Terry RF, Charles E, Purdy B, Sanford A. An analysis of research priority-setting at the World Health Organization – how mapping to a standard template allows for comparison between research priority-setting approaches. *Health Res Policy Syst* 2018; **16**: 116.

4 Davies SE, Harman S, Manjoo R, Tanyag M, Wenham C. Why it must be a feminist global health agenda. *The Lancet* 2019; **393**: 601–3.

5 Büyüm AM, Kenney C, Koris A, Mkumba L, Raveendran Y. Decolonising global health: if not now, when? *BMJ Glob Health* 2020; **5**: e003394.

6 The Lancet Global Health. Decolonising COVID-19. *Lancet Glob Health* 2020; **8**: e612.

7 Jenkins K, Narayanaswamy L, Sweetman C. Introduction: Feminist values in research. *Gend Dev* 2019; **27**: 415–25.

8 Morgan R, George A, Ssali S, Hawkins K, Molyneux S, Theobald S. How to do (or not to do)… gender analysis in health systems research. *Health Policy Plan* 2016; **31**: 1069–78.

9 Theobald S, Morgan R, Hawkins K, Ssali S, George A, Molyneux S. The importance of gender analysis in research for health systems strengthening. *Health Policy Plan* 2017; **32**: v1–3.

10 Pederson A, Greaves L, Poole N. Gender-transformative health promotion for women: a framework for action. *Health Promot Int* 2015; **30**: 140–50.

11 Hall KS, Samari G, Garbers S, *et al.* Centring sexual and reproductive health and justice in the global COVID-19 response. *The Lancet* 2020; **395**: 1175–7.

12 Bennett S, Jessani N, Glandon D, *et al.* Understanding the implications of the Sustainable Development Goals for health policy and systems research: results of a research priority setting exercise. *Glob Health* 2020; **16**: 5.

13 Wigginton B, Lafrance MN. Learning critical feminist research: A brief introduction to feminist epistemologies and methodologies. *Fem Psychol* 2019; : 095935351986605.

Appendix for protocol

Table of Contents

Appendix 1:
Crafting a Feminist and Decolonial Research Agenda-setting Process 2

Appendix 2:
Literature on research agenda-setting processes.. 8

Appendix 3:
Comparison of established methods for research agenda-setting in health: brief description of the approaches and processes 14

George AS, *et al. BMJ Global Health* 2023; 8:e011315. doi: 10.1136/bmjgh-2022-011315

Appendix 1:
Crafting a Feminist and Decolonial Research Agenda-setting Process

Through this research prioritisation process, we hope to build on the recent conversations around gender and COVID-19, and bring together an epistemic community that is diverse and inclusive to build a responsive, actionable, and accountable research agenda for gender and COVID-19.

We propose the use of feminist research values[1,2] a decolonizing focus, and gender analysis frameworks[3–5] to inform the ways of work, the agenda output, as well as guidance for the implementation of research from the set agenda. This design is also our response to recent calls for a feminist-oriented focus in global health[2] and decolonizing processes in global health, particularly from early career professionals.[6,7] To achieve a gender-responsive global agenda for research, we need feminist solidarity[2] in understanding, voice and action from multiple communities of stakeholders.

Key characteristics includes:

- co-creation of process and participatory design,
- reflexive declarations on the identities, positions, and privileges of the thematic group and research agenda implementers
- consideration of gender dynamics of the issues faced by women, men and non-gender binary populations, the influence of intersectionality
- gender responsiveness of solutions, with the inclusion of gender transformation, gender equality, redistribution of gender power dynamics as the endpoint, and key outcome
- establishing an agenda that advocates for the planning and implementation of research driven by researchers in LMICs, in partnerships with local and global stakeholders.

Conceptualization of Gender and Global Health

Gender is the socially constructed, contextually, culturally and temporally fluid characteristics associated with being a woman, girl, man, boy or person of a non-binary gender group (i.e. transgender and intersex), and is also relational across these groups.[8] This framing is different from, but overlaps with sex as a biological category, determined by genetic, biochemical and physiological factors such chromosomes, hormones and reproductive organs, and cis-gender, which is the continuous identification with designations of sex and gender at birth. As a social identity and status, gender is also historical, hierarchical, institutional,[9] and intersects with broader social and economic inequalities such as ethnicity, socio-economic status, disability, age, sexual orientation and geographic locations.[8]

Of relevance to the health sector is how gender and sex influence health, prevention and treatment outcomes, including through protective behaviours, service uptake and interactions within health-focused institutions. Within households, communities, society and health systems, gender-inequitable norms, including the burden of unpaid care responsibilities, power imbalances, violence against women and girls lead to constrained personal agency, and normalised experiences of discrimination and violence, and inequitable access and/or control over resources that influence their health status.[11–17].

Global Health, as we conceptualize for the purpose of this exercise, is the interconnected attention on community, national, regional, and global factors, and actors, that contribute to the health status of the world. This requires a focus on both actors with primary aims to influence health, and also multiple other actors whose motivations, services, products, ideologies or ways of function also indirectly influence health. This also requires the convergence of multiple types and fields of knowledge to achieve cross-cutting intersectoral and structural level action, including on human rights, international security, political and economic systems.

Global health remains diversely understood and framed by many who identify as working within this field. Some conceptualizations include a 'collection of problems' which 'turn on the quest for equity'; equity in health indices within and between the national boundaries of high, middle or low-income countries'.[18,19] Others broadly describe it as an organizing framework, a conceptual framing, a set of legal norms, and as a distinct field of practice, as an emerging science, an area of policy and research and as a formative disciplinary field of study.[20] Overall, the global health system uses 'polylateral arrangements' to govern, finance and deliver health resources within a global context, but also is influenced by how actors interrelate, and are influenced by each other.[21]

Feminist values and analytic lens

Feminist research values include:[1,2]

- empowering all those that participate in the research process
- addressing power, hierarchical blocks, and actively seek out those who are excluded, address participation barriers
- the pursuit social transformation
- reflections and disclosure of researcher positionality and subjectivity
- sustained and equalizing relationships through inclusive participatory approaches
- valuing complexity, nuances, human experience, and voice
- translation of values across cultures, contexts, institutions, and languages.

Feminist and constructivist scholars highlight that research is not neutral or value-free, and is often derived from the assumptions and perceptions of the researchers that design, conduct and analyze the findings.[1] The feminist approach is also not a case of involving only women- not all women are feminist by virtue of being a woman, and not all men are patriarchal, anti-feminist or unsupportive of gender mainstreaming.[2] Instead, it advocates for the focus on structural and social power inequalities.

We suggest an interpretive lens, with a focus that includes, but also that pushes beyond biomedical data and population statistics is required, to include lived experience, multiple cultural and intersectional voices.[22] Appropriate analytic lenses and approaches for research agenda-setting are tied to the main disciplinary norms and perspectives. For instance, an interpretive approach,(as we propose) is more suitable for areas such as health systems and policy research, where there are great uncertainty and complex pathways, where quantitative algorithms may not be adequate.[23] Relatedly, feminist and constructivist scholars highlight that research is not neutral or value-free, and is often derived from the assumptions and perceptions of the researchers that design, conduct and analyze the findings.[1]

Feminist methodologies also include quantitative empirical methods, aligned to more positivist and numerical approaches of study and evaluation as those established in the biomedical and

clinical domains.[1,24] The feminist approach is also not a case of involving only women- not all women are feminist by virtue of being a woman, and not all men are patriarchal, anti-feminist or unsupportive of gender mainstreaming.[2] Instead, it advocates for the focus on structural and social power inequalities.

Decolonial approach

Concurrently, COVID-19 is exposing historically embedded structural inequalities both in the populations, and in the systems of work within the global health community.[6,7] The dominance of global north representation, influence and agenda-setting even of research priorities and problem gaze is rampant, and is often used as a strategy to address global south problems.[25] Even prior to the pandemic, there have been global movements to decolonize global health that call for:[6]

- changing paradigms and knowledge,
- disrupting implicit biased perceptions of LMICs,
- shifting leadership and visibility to LMIC organizations and people to initiate and drive global health projects.

In this process, we will actively seek to approach and include voices and project leaders from the global south including the steering and advisory groups, and respond to language requirements should there be any requests from those who have responded to our invitations. All communication and group discussions will be online, including thematic group discussions, long-listing of research questions and ranking exercise. Where appropriate and feasible, the steering team will seek to compensate use of internet data for participants from low-income countries or who do not have organizational resources or infrastructure to participate in the discussions.

This decolonizing principle is also aligned with the feminist way of work and analytic lens.[24,26] In the field of education for instance, there are calls to 'decolonize feminist solidarity'[26,27] through:

- 'stepping forward' (of actors' from global south and LMICs)
- 'standing with' (of actors with existing visibility, resources, and capabilities, usually from global north and high-income countries)
- 'staying connected' through building shared knowledge, relational expertise, and agency

The proposed way of work

The ways of work will also align to the feminist values that drive this project:

- Relational principles of interdependency & cooperation; respect, & trust-building.
- Shared and equal power between team members; cultural and language sensitivity, valuing and listening to all voices and ideas.
- Interdisciplinary learning and listening
- Transparent process and decision-making
- Use of online platforms to overcome geographic limitations
- Clear lines of communication, articulation of roles and responsibilities and timelines
- Multiple languages of communication- where required

Reflexivity and positionality

This research agenda-setting process will be based on the experiences and expertise of the collective group of participants. To strengthen the rigor, accountability, transparency and credibility of this process, we propose for the collection of reflexivity statements prior to the start of the group discussions, to situate the researchers' and participants' position, perspective, and presence in the produced knowledge.[28,29]

This is an important step to:

- understand the extent of representation and types of voices at the discussion
- account for the diverse subjectivities influenced by the participants' position, but also helps
- contextualize the relational differences between and within groups.

The reflexive exercise involves the collection of statements on:

- identity: personal (class, gender, ethnic, racial, or national, and history) and professional factors (institutional location, areas of work, mandate, and agenda)
- history & experience
- representation of and accountability towards other stakeholders
- perceptions on future and shifts required

References

1 Jenkins K, Narayanaswamy L, Sweetman C. Introduction: Feminist values in research. *Gend Dev* 2019; **27**: 415–25.

2 Davies SE, Harman S, Manjoo R, Tanyag M, Wenham C. Why it must be a feminist global health agenda. *The Lancet* 2019; **393**: 601–3.

3 Morgan R, George A, Ssali S, Hawkins K, Molyneux S, Theobald S. How to do (or not to do)… gender analysis in health systems research. *Health Policy Plan* 2016; **31**: 1069–78.

4 Theobald S, Morgan R, Hawkins K, Ssali S, George A, Molyneux S. The importance of gender analysis in research for health systems strengthening. *Health Policy Plan* 2017; **32**: v1–3.

5 Pederson A, Greaves L, Poole N. Gender-transformative health promotion for women: a framework for action. *Health Promot Int* 2015; **30**: 140–50.

6 Büyüm AM, Kenney C, Koris A, Mkumba L, Raveendran Y. Decolonising global health: if not now, when? *BMJ Glob Health* 2020; **5**: e003394.

7 The Lancet Global Health. Decolonising COVID-19. *Lancet Glob Health* 2020; **8**: e612.

8 WHO. Gender and Health: Fact Sheet. WHO. 2018; published online Aug 23. http://www.who.int/news-room/fact-sheets/detail/gender (accessed Sept 20, 2018).

9 WHO. WHO Gender, equity, human rights. WHO. 2020. http://www.who.int/gender-equity-rights/en/ (accessed Oct 20, 2020).

10 Andersson N, Cockcroft A. Choice-Disability and HIV Infection: A Cross Sectional Study of HIV Status in Botswana, Namibia and Swaziland. *AIDS Behav* 2012; **16**: 189–98.

11 Cockcroft A, Marokoane N, Kgakole L, Tswetla N, Andersson N. Access of choice-disabled young women in Botswana to government structural support programmes: a cross-sectional study. *AIDS Care* 2018; **30**: 24–7.

12 Bekker L-G, Alleyne G, Baral S, *et al.* Advancing global health and strengthening the HIV response in the era of the Sustainable Development Goals: the International AIDS Society—Lancet Commission. *The Lancet* 2018; **392**: 312–58.

13 Bloom S, Negroustoueva S. Compendium of Gender Equality and HIV Indicators — MEASURE Evaluation. 2014 https://www.measureevaluation.org/resources/publications/ms-13-82 (accessed Oct 12, 2018).

14 Richardson ET, Collins SE, Kung T, *et al.* Gender inequality and HIV transmission: a global analysis. *J Int AIDS Soc* 2014; **17**. DOI:10.7448/IAS.17.1.19035.

15 Amin A. Addressing gender inequalities to improve the sexual and reproductive health and wellbeing of women living with HIV. *J Int AIDS Soc* 2015; **18**. DOI:10.7448/IAS.18.6.20302.

16 Orza L, Bass E, Bell E, *et al.* In Women's Eyes: Key Barriers to Women's Access to HIV Treatment and a Rights-Based Approach to their Sustained Well-Being. *Health Hum Rights* 2017; **19**: 155–68.

17 Morgan R, Ayiasi RM, Barman D, *et al.* Gendered health systems: evidence from low- and middle-income countries. *Health Res Policy Syst* 2018; **16**: 58.

18 Farmer P, Kim JY, Kleinman A, Basilico M. Reimagining Global Health: An Introduction. University of California Press, 2013 https://www.amazon.com/Reimagining-Global-Health-Introduction-Anthropology/dp/0520271998 (accessed Oct 20, 2020).

19 Abimbola S. On the meaning of global health and the role of global health journals. *Int Health* 2018; **10**: 63–5.

20 Taylor S. 'Global health': meaning what? *BMJ Glob Health* 2018; **3**: e000843.

21 Hoffman SJ, Cole CB. Defining the global health system and systematically mapping its network of actors. *Glob Health* 2018; **14**: 38.

22 Hall KS, Samari G, Garbers S, *et al.* Centring sexual and reproductive health and justice in the global COVID-19 response. *The Lancet* 2020; **395**: 1175–7.

23 Bennett S, Jessani N, Glandon D, *et al.* Understanding the implications of the Sustainable Development Goals for health policy and systems research: results of a research priority setting exercise. *Glob Health* 2020; **16**: 5.

24 Wigginton B, Lafrance MN. Learning critical feminist research: A brief introduction to feminist epistemologies and methodologies. *Fem Psychol* 2019; : 095935351986605.

25 Reidpath DD, Allotey P. The problem of 'trickle-down science' from the Global North to the Global South. *BMJ Glob Health* 2019; **4**: e001719.

26 Walters S, Butterwick S. Moves to Decolonise Solidarity through Feminist Popular Education. *Forg Solidar* 2017; : 27–38.

27 D'Arcangelis CL, Huntley A. No More Silence. In: Manicom L, Walters S, eds. Feminist Popular Education in Transnational Debates: Building Pedagogies of Possibility. New York: Palgrave Macmillan US, 2012: 41–58.

28 Finlay L. "Outing" the Researcher: The Provenance, Process, and Practice of Reflexivity. *Qual Health Res* 2002; **12**: 531–45.

29 Corbin J, Strauss A. Basics of Qualitative Research: Techniques and Procedures for Developing Grounded Theory. SAGE Publications, 2014.

Appendix 2:
Literature on research agenda-setting processes

Benefits of research agenda-setting processes

Global research prioritization or agenda-setting aims to guide research that fills knowledge gaps related to the unmet policy, programming and community needs to achieve higher health impact, equity and social justice.[1] Coordinated research agendas can also channel resources and capabilities to avoid overlaps and duplicative actions.[2] This is particularly necessary given the need to address underfunded research areas within finite research investments.[1] The recent WHO's Covid-19 R&D Blueprint[3] demonstrates the value of supporting global research consensus, agenda and tracking. An unprecedented level and speed of global collaboration was achieved, with an aim to contain the spread of the epidemic, open channels of information exchange between countries, provide timely care, and develop therapeutics and vaccines. As of July 2020, the WHO research database[4] contains close to 40,000 full-text research entries.

In the last ten years, the range and number of research agenda-setting efforts has increased.[5] A review of 165 research agenda setting exercises found about 50% focused on high-income countries, 28% on low and middle income countries (LMIC), and 22% at a global level.[5] Within LMIC research prioritization, LMIC governments and academics only establish about 32% and 15% of research agendas respectively.[6] Without formal research agendas, priorities in LMIC are often determined by external funders, and may not respond to the specific health or system needs of the country.[6] Sometimes, priorities are set by those who are not involved in the implementation, those who are not affected by the problems, or without mechanisms to ensure accountability towards the agenda, compromising the shared value and legitimacy of the agenda.[1,7]

Overview of research prioritisation approaches

Past reviews have noted an increase in research agenda-setting in the last ten years.[5] A review of past research agenda setting exercises found about 50% focused on high-income countries, and 22% at a global level.[5] With research priorities for low and middle income countries (LMIC) on the other hand, most occurred at global (46%) or national (43%) level.[6] However, LMIC governments and academics only establish about 32% and 15% of research agendas respectively, and only12% report an implementation or follow-up strategy.[6] Without formal research agendas, priorities in LMIC are often determined by external funders, and may not respond to the specific health or system needs of the country.[6] Indeed, many investments in health research are prompted by diverse, and at times, conflicting aims. Sometimes, priorities are also set by those who are not involved in the implementation, those who are not affected by the problems, or without mechanisms to ensure accountability towards the agenda, compromising the shared value and legitimacy of the agenda.[1,7]

There is no 'gold-standard' approach to research agenda-setting, but there are multiple established process frameworks that can be considered based on the context, need and circumstance of the research areas.[1] The literature highlights several key learnings and suggestions on how to strengthen design and implementation of research agenda-setting processes.

Overall, most agenda-setting processes follow three phases of implementation:[1]

- Preparation
  planning, information gathering, stakeholder engagement

- Deciding priorities
  methods using relevant criteria and methods to direct discussions

- Implementing priorities

Decision-making is largely informed by two broad approaches-consensus based (group) or metrics based (using algorithms and pooled ranking) approaches.[1] Strategies to identify priorities have used various combinations of consultations or surveys to collect expert opinion through multistage participatory processes,[8–10] qualitative interviews,[11] literature reviews to identify gaps and needs, and ranking based on opinion or weighted criteria.[2] In most exercises in established research areas, review and critique of credible existing evidence[7] usually supplements the decision-making process.

There are established formal processes that are commonly used, and past research has compared the individual advantages and disadvantages of these .[5] (Appendix 2-attached) These include:

- Essential National Health Research (ENHR)
- Combined Approach Matrix (CAM- consensus-based)
- Council on Health Research for Development (COHRED);
- Child Health Nutrition Research Initiative (CHNRI- metrics-based
- James Lind Alliance Method
- Delphi

Planning and scoping: the design of the process

In a comparative review of 165 exercises, CHNRI (26%) and Delphi (24%) were the most commonly used, followed by consultations (19%), online surveys (8%), combined literature review with questionnaires (9%) and James Lind Alliance method (8%).[5] In another review of approaches used in 116 exercises by WHO (2018), expert consultation was the most commonly used approach (86%) to establish priorities (26% as only method, 52% in combination with a literature review).[2] In LMICs, common processes include the use of physical workshops or conference events, CHNRI and combination of literature review, in-depth interviews and consultation.[6] In essence, multiple methods and inputs are used in research prioritisation with varying degrees of formalization.

A literature review (systematic, scoping, evidence mapping) and available data sources such as technical reports, etc. are used as formative inputs into the preparatory phase to identify the scope and needs of the process.[7] In some cases, consultations with stakeholders such as policy-makers also inform the preparatory phase to establish the scope and needs of the process.[9,10] These activities help establish the context, scope, beneficiaries, of the agenda, and

influences of stakeholders such as mandates, capacity, and resources early in the implementation.

Who should participate in process?

In the past, research prioritisation exercises have privileged the opinion and input of a closed and selected group of experts, without engaging with diverse stakeholders, including those who play a role in implementing the agenda or are influenced by the agenda i.e. patient groups, communities, health providers, policy-makers. Even amongst experts, research agenda-setting at individual and organizational levels can be a gendered process, influenced by the complex and accumulative gender inequalities within academic life- publication rate, grants, decision-making, tenureship, leadership etc.[12]

Research prioritisation processes can be ‘complex, political and value-laden’, as accommodating different perspectives, priorities and values of stakeholders can be challenging.[7] However, inclusion of diverse sets of stakeholders can support the alignment of academic and political interests, and co-generated evidence and consequent knowledge translation can better meet the needs of stakeholders across diverse contexts.[13] The inclusion of diverse stakeholders is now an increasingly normal and standard practice, since this is crucial to create a shared sense of responsibility and accountability towards the implementation and ownership of the research agenda.[7] Creating joint ownership of the agenda and its implementation can be achieved through early involvement of all stakeholders in the design and work process.[11]

Research prioritisation exercises should be designed to include and share power with community, and non-expert voices-including the terms by which the exercise is undertaken, how they participate and influence the outputs.[11,14] Token representation or presence without meaningfully including their voice, placing weight on their input, or addressing the associated power dynamics that exclude their epistemic processes and perspectives must be avoided.[11] Admittedly, all stakeholders are not equally experts in multiple areas, but are able to bring diverse epistemic understanding to the decision-making table. To counter this and balance the subjectivities involved, many exercises combine consensus with metrics-based approaches, such individually proposing priority themes and then ranking based on criteria.[1]

Decision-making on priorities

Decision-making is largely informed by two broad approaches-consensus based (group) or metrics based (using algorithms and pooled ranking) approaches.[1] Strategies to identify priorities have used various combinations of consultations or surveys to collect expert opinion through multistage participatory processes,[8–10] qualitative interviews,[11] literature reviews to identify gaps and needs, and ranking based on opinion or weighted criteria.[2] In most exercises in established research areas, review and critique of credible existing evidence[7] usually supplements the decision-making process.

Different strategies have been used to collect the long-list of research priorities-interviews, focus groups, workshops, and surveys, conducted both online and in person.[7] These inputs are often collated and categorized into refined lists, by also removing duplicates, near-similar or out-of-

scope contributions. Some exercises have also further cross-checked the refined list using established evidence- i.e. systematic reviews or evidence mapping.[7]

The prioritization phase often includes practices such as scoring, ranking, voting, and ordering, using similar platforms or modes of engagement as in the previous phase of establishing a long-list.[7] There are merits to both consensus and metrics-based approaches, and in many cases, there is a combined use of consensus-building and metrics-based listing.[1] The consensus approach supports acceptability and buy-in, with the caveat that metric based ranking can prevent the dominance of select voices.[1] It is critical to aim to be inclusive of the different forms of knowledge, values and viewpoints, particularly amongst non-homogenous groups of stakeholders.[1]

Metrics-based approaches provide structure to the process of discussions and prioritization. However, the use of selected criteria can also add complexity, and deprioritize other domains of value to stakeholders, and though it provides structure to the decision-making process, it can provide a false sense of objectivity.[7] At the same time, the lack of transparency in decision-making has been highlighted as a weakness is many research prioritisation approaches, that affects the validity and strength of the research agenda.[2,7] For instance, the benefits of a metric based approach is its ability to provide room for objectivity and replicability of process.[15] Prioritisation is based on an established quantitative threshold, such as rank scores, proportions or votes, or other criteria, relative to others in the list.[7]

The CHNRI process has a systematic list of criteria that was advocated at its inception.[15] These are:

- answerability,
- effectiveness,
- deliverability,
- the potential for a substantial reduction of disease burden
- the impact on equity

Adaptations to this list have since been applied by subsequent implementers of this approach. Some of the other domains include:

- low cost
- sustainability
- acceptability
- feasibility
- relevance

Outputs

Clarity and sufficient details should be included based on the needs of the implementers. Past initiatives have advocated for the use of PICO (Population, Intervention, Comparator, Outcome) format, particularly in biomedical or therapeutic research areas.[7] In others, research themes were considered sufficient.[7] Others have categorized needs based on research cycles, such as the WHO Strategy on Research for Health, which include descriptions of the research problem, cause and risk factors, solutions and interventions, implementation enablers and barriers, and evaluation of impact.[2]

George AS, *et al. BMJ Global Health* 2023; 8:e011315. doi: 10.1136/bmjgh-2022-011315

The number of priorities is context-dependent on the need and group of stakeholders. Past exercises have included a diverse range from over a 100 in the 'WHO Public Health Research Agenda for Influenza' (2017 update) to less than 10 in 'Transgender People and HIV' (2015). [2] In LMICs, the number has ranged from 5 to 588, with a median of 29.[6]

Evaluation and implementation of agenda

Evaluations of acceptability and usefulness after a research agenda has been set, including determining stakeholder satisfaction with the process and their ability to contribute meaningfully, is also an important practice.[7] While there are no agreed metrics to assess the success of agenda-setting process, there is a recently proposed evaluation framework (REPRISE) to assess the quality of the process and outputs.[7]

Implementation of the agenda is highlighted as weakness in past literature,[6] and so planning early for implementation and uptake, including identifying the key beneficiaries and implementation actors,[7,9] should be included in the early phases of these exercises.[1] This should include potentially- funders, national and sub-national governments, researchers, intersectoral actors [9] At a basic level, past strategies have included informing and obtaining buy-in for national governments, policy-makers and funding agencies to also prioritize resource allocation on the set agenda, and working with researchers to develop proposals.[7] Additionally, constraints on health research in LMICs need to be considered to support and enable locally-led implementation and pursuit of impact.[6]

References

1 Viergever RF, Olifson S, Ghaffar A, Terry RF. A checklist for health research priority setting: nine common themes of good practice. *Health Res Policy Syst* 2010; **8**: 36.

2 Terry RF, Charles E, Purdy B, Sanford A. An analysis of research priority-setting at the World Health Organization – how mapping to a standard template allows for comparison between research priority-setting approaches. *Health Res Policy Syst* 2018; **16**: 116.

3 Word Health Organization. R&D Blueprint and COVID-19. 2020. https://www.who.int/teams/blueprint/covid-19 (accessed July 9, 2020).

4 Word Health Organization. COVID-19 :Global literature on coronavirus disease. 2020. https://search.bvsalud.org/global-literature-on-novel-coronavirus-2019-ncov/ (accessed July 9, 2020).

5 Yoshida S. Approaches, tools and methods used for setting priorities in health research in the 21st century. *J Glob Health* 2016; **6**. DOI:10.7189/jogh.06.010507.

6 McGregor S, Henderson KJ, Kaldor JM. How Are Health Research Priorities Set in Low and Middle Income Countries? A Systematic Review of Published Reports. *PLoS ONE* 2014; **9**. DOI:10.1371/journal.pone.0108787.

7 Tong A, Synnot A, Crowe S, *et al.* Reporting guideline for priority setting of health research (REPRISE). *BMC Med Res Methodol* 2019; **19**: 243.

8 Scott K, Jessani N, Qiu M, Bennett S. Developing more participatory and accountable institutions for health: identifying health system research priorities for the Sustainable Development Goal-era. *Health Policy Plan* 2018; **33**: 975–87.

9 Bennett S, Jessani N, Glandon D, *et al.* Understanding the implications of the Sustainable Development Goals for health policy and systems research: results of a research priority setting exercise. *Glob Health* 2020; **16**: 5.

10 Glandon D, Meghani A, Jessani N, Qiu M, Bennett S. Identifying health policy and systems research priorities on multisectoral collaboration for health in low-income and middle-income countries. *BMJ Glob Health* 2018; **3**: e000970.

11 Pratt B. Towards inclusive priority-setting for global health research projects: recommendations for sharing power with communities. *Health Policy Plan* 2019; **34**: 346–57.

12 Santos JM, Horta H, Amâncio L. Research agendas of female and male academics: a new perspective on gender disparities in academia. *Gend Educ* 2020; : 1–19.

13 Essink DR, Ratsavong K, Bally E, *et al.* Developing a national health research agenda for Lao PDR: prioritising the research needs of stakeholders. *Glob Health Action* 2020; **13**: 1777000.

14 Boaz A, Hanney S, Borst R, O'Shea A, Kok M. How to engage stakeholders in research: design principles to support improvement. *Health Res Policy Syst* 2018; **16**: 60.

15 Rudan I, Yoshida S, Chan KY, *et al.* Setting health research priorities using the CHNRI method: VII. A review of the first 50 applications of the CHNRI method. *J Glob Health* 2017; **7**: 011004.

Appendix 3:
Comparison of established methods for research agenda-setting in health: brief description of the approaches and processes

Taken from: Approaches, tools and methods used for setting priorities in health research in the 21st century (Yoshida, 2016)

	Process	Participation	Research prioritization process	Scoring Criteria	Scoring options	Advantages	Disadvantages
**ENHR**	ENHR was developed by Commission on Health Research for Development in 1990. It is a step by step guide for national research priority setting focused on equity in health and development. Strategy focused on inclusiveness in participation, broad–based consultations at different levels, both quantitative and qualitative information used, and stewardship by small working group.	Participants are involved through a small representative working group which can facilitate the process, through various consultations. These stakeholders have a major stake in the goal of equity in health and development. The four major categories of participants include: researchers, decision makers, health service providers and communities.	Stakeholders suggest priority areas, via evidence based situation analysis (such as looking at health status, health care system, health research system). Research ideas are gathered from a nomination process from different stakeholders. Consensus building using methods such as brainstorming, multi–voting, nominal group technique, round–table is then used to select research ideas.	Criteria is selected as to be:<br>• Appropriate to the level of the action of the *i.e.* global, national, district;<br>• Detailed in definition;<br>• Independent of each other;<br>• Contain information base;<br>• Reflect equity promotion and development;<br>• Manageable number;<br>• Expressed in a common language.<br><br>Criteria are agreed on by brainstorming of large collection of possible criteria, clearly defining the meaning of each criterion	Each criteria is scored: Point score to each criteria OR Number of score choices to each criteria	• Broad based inclusion and participation of different stakeholders.<br>• Multidisciplinary and cross-sectoral approach<br>• Partnership development<br>• Transparent process<br>• Systematic analyses of health needs	• Vague criteria and lack of transparency in individual process used by countries<br>• Few countries had guidelines on how to develop nor apply criteria<br>• Needs stronger representation of groups such as private sector, parliamentarians, donors, international agencies<br>• Does not provide methodology for identifying participants

**CAM**	Developed by the Global Forum for Health Research, CAM was to bring together economic and institutional dimensions into an analytical tool with the actors and factors that play a key role in health status of a population.<br><br>It also aims to organise and present a large body of information that enters the priority setting process.<br><br>This will help decision makers make rational choices in investment to produce greatest reduction in burden of disease.	Institutional approach involving: individual, household and community; health ministry and other health institutions; other sectors apart from health; and macroeconomic level actors.	Five step process including<br>• measuring the disease burden,<br>• analysing determinants,<br>• getting present level of knowledge,<br>• evaluating cost and effectiveness, and<br>• present resource flows.<br><br>For each main disease and risk factor, institutions and stakeholders with particular knowledge are brought together to provide information via workshops and brainstorming. Each institution will feed into matrix; the matrix will reveal	Criteria based on questions of what is a research priority in the context, and what is not known but should be.	NA	• Creates framework of information<br><br>• Identifies gaps in knowledge<br><br>• Facilitates comparisons between sectors<br><br>• Broad inclusion of actors<br><br>• 3D–CAM includes equity	• Difficult and time–consuming as involves multi–stage discussion<br><br>• Does not provide algorithm to establish and score research priorities therefore is not repeatable nor systematic<br><br>• Does not provide methodology for identifying participants

			how little information is available<br><br>Participants choose the priority topics based on CAM evidence, then group and reduce to establish priorities.				
**James Lind Alliance Method**	Focuses on bringing patients, carers and health professionals in order to identify treatment uncertainties which will become research questions. The method uses a mixture of data gathering, quantitative and qualitative analysis to create research priorities in areas of treatment uncertainty.	Participants are identified through Priority Setting Partnerships which brings patients, carers and clinicians equally together and agree through consensus priorities.	Treatment uncertainties defined through systematic reviews<br><br>Recommendation list informed by existing literature, to list of uncertainties, which are then verified through systematic reviews of databases i.e. Cochrane, DARE, NICE, Sign.<br><br>An uncertainty is deemed genuine when a reported confidence interval in a systematic review does not cross the line of effect or line of unity.<br>A virtual interim priority ranking, and a final priority setting workshop	No clear criteria are identified with which to use.	Ranked AND Qualitative consensus	Takes into account underrepresented groups<br><br>Applicable to small scale prioritisation (eg, hospital<br><br>Mixture of methods	– Time consuming to identify and verify treatment uncertainties<br>– Selection of criteria not clear<br>– Not suitable for global level, nor specific disease domains<br>– Very clinically orientated<br>– Disproportionate mix of participants may skew information base

			takes place to agree upon 10 prioritised uncertainties through consensus building.				
**COHRED**	COHRED uses a management process for national level exercises to show important steps for priority setting processes	Participants are identified through the chosen methods outlined in the steps of the COHRED guide.	Identification of priority issues much choose method best suited to local context and needs either through compound approaches (ENHR, CAM, Burden of Disease) or foresighting techniques (Visioning, Delphi). Consider using more than one method to optimize usefulness of results.	COHRED presents ranking techniques that can be used to rank priority issues including direct and indirect valuation techniques.	Ranked	• Overview approach providing steps<br>• Discusses wide range of options<br>• Flexible to contexts and needs	Too general and unspecific<br><br>Lack of criteria transparency
**CHNRI**	The CHNRI methodology was introduced in 2007 by the Child Health and Nutrition Research Initiative of the Global Forum for Health research. The methodology was	Participants are identified by management team based on their expertise (eg, number of publications, experience in implementation research and programmes etc). Participants	Research ideas are generated by participants or by management team based on the current evidence. If former, usually each participant is asked to provide maximum of three	Five standard criteria are usually used:<br>• Answerability<br>• Equity<br>• Impact on burden<br>• Deliverability<br>• Effectiveness.	Each criteria is scored: Point score to each criteria in the scale of 0, 0.5 and 1 or in the scale of 0 to 100.	• Simple, inclusive and replicable and thus systematic and transparent process.<br>• Independent ranking of experts (avoid having the situation where	• Potentially represent collective opinion of the limited group of people who were included in the process.

	developed to address gaps in the existing research priority methods. The CHNRI method is developed to assist decision making and consensus development. The method include soliciting ideas from different carder of participants on the given health topic and use independent ranking system against the pre–defined criteria to prioritise the research ideas.	includes stakeholders who might not have the technical expertise but have view on the health topic of concern.	research questions against the predefined domain of health research (eg, descriptive research, development research, discovery research and delivery research). The ideas are usually submitted via online survey and consolidated by the management team.	Though the five standard criteria are used in more than 70% of the research priority setting exercises, the method offers optional criteria to be used to replace the standard criteria depending on the needs and context of the exercises.<br><br>For example, criteria such as low cost, sustainability, acceptability, feasibility, innovation and originality are used to replace or in addition to the standard criteria.		one strongly minded individual affecting the group<br>• decision)<br>• Less costly	• Scoring affected by currently on–going research
**Delphi**	Delphi, mainly developed in the 1950s, is a systematic, interactive forecasting method which relies on a panel of experts and questionnaires.	Participants are eligible to be invited if they have related backgrounds and experiences concerning the target issue, are capable of contributing, and are willing to revise their initial judgements in order to reach consensus.<br><br>Participants are considered	In the first round an open–ended questionnaire is sent to solicit information about a content area from Delphi participants. Investigators will then turn the responses into a well–structured questionnaire to be used as survey for data collection.<br><br>Through four rounds experts	NA	Rate or ranking AND Consensus building	• Multiple iterations and feedback process<br>• Flexible to change<br>• Anonymity of respondents	• Does not provide methodology for identifying participants<br>• Lack of criteria transparency<br>• Potential for low response rate due to multiple iterations<br>• Time–consuming<br>• Potential for investigators and

		and selected through investigators, ideally through a nomination process, or selection from potential leaders or authors through publication. It is suggested that the three groups are used: top management decision makers who will utilise outcomes of Delphi study; professional staff members and their support team; respondents to the Delphi questionnaire. It is recommended to use the minimally sufficient number to generate representative pooling of judgements – however no consensus yet as to optimal number of subjects.	answer questionnaires; the facilitator summarises anonymously the forecast after the first round and the experts are then asked to revise their earlier answer thereby decreasing the range of answers and converging towards the correct answer. Up to four iterations can be used				facilitators to bias opinions

Essential National Health Research (ENHR); Combined Approach Matrix (CAM); Council on Health Research for Development (COHRED); CHNRI method- Child Health Nutrition Research Initiative

Reference

Yoshida, S., 2016. Approaches, tools and methods used for setting priorities in health research in the 21st century. J. Glob. Health 6. https://doi.org/10.7189/jogh.06.010507
