# Supplementary material for: A shared agenda for gender and COVID-19 research: priorities based on broadening engagement in science
Source: BMJ Glob Health. 2023 May 22;8(5):e011315. doi: 10.1136/bmjgh-2022-011315 (PMC10230361; doi:10.1136/bmjgh-2022-011315)
Supplement: Supplementary data [file bmjgh-2022-011315supp002.pdf]

Supplementary file: Social media visuals used for the shared gender and COVID-19 research agenda setting collaboration

Process of public engagement in setting shared gender and COVID-19 research priorities

Key Social Media Metrics

The following metrics are tracked across mentions of #ResearchAgendaSetting, #GHHBuzzBoards and #BetterCOVIDScience and associated keywords and phrases.

Total mentions
967

Total Engagement
1908

Potential Reach (Twitter)
3 million (impressions)

Influencers (by reach)

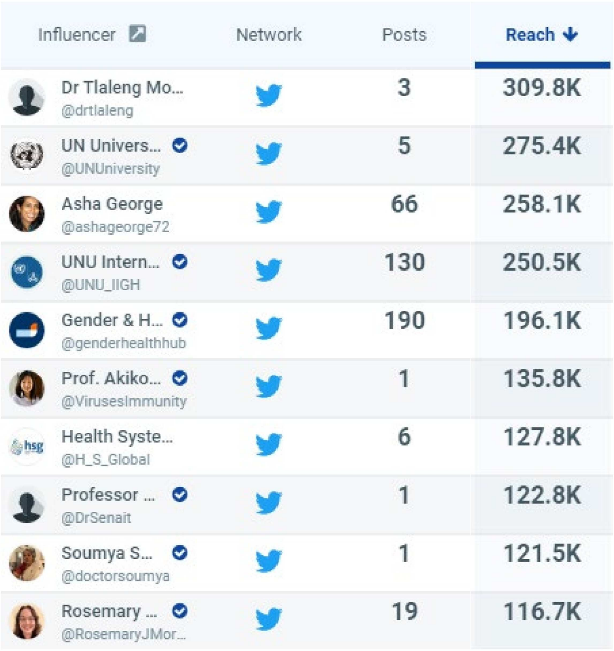

Influencer	Network	Posts	Reach
Dr Tlaleng Mo... @drtlaleng		3	309.8K
UN Univers... @UNUniversity		5	275.4K
Asha George @ashageorge72		66	258.1K
UNU Intern... @UNU_IIGH		130	250.5K
Gender & H... @genderhealthhub		190	196.1K
Prof. Akiko... @VirusesImmunity		1	135.8K
Health Syste... @H_S_Global		6	127.8K
Professor ... @DrSenait		1	122.8K
Soumya S... @doctorsoumya		1	121.5K
Rosemary ... @RosemaryJMor...		19	116.7K

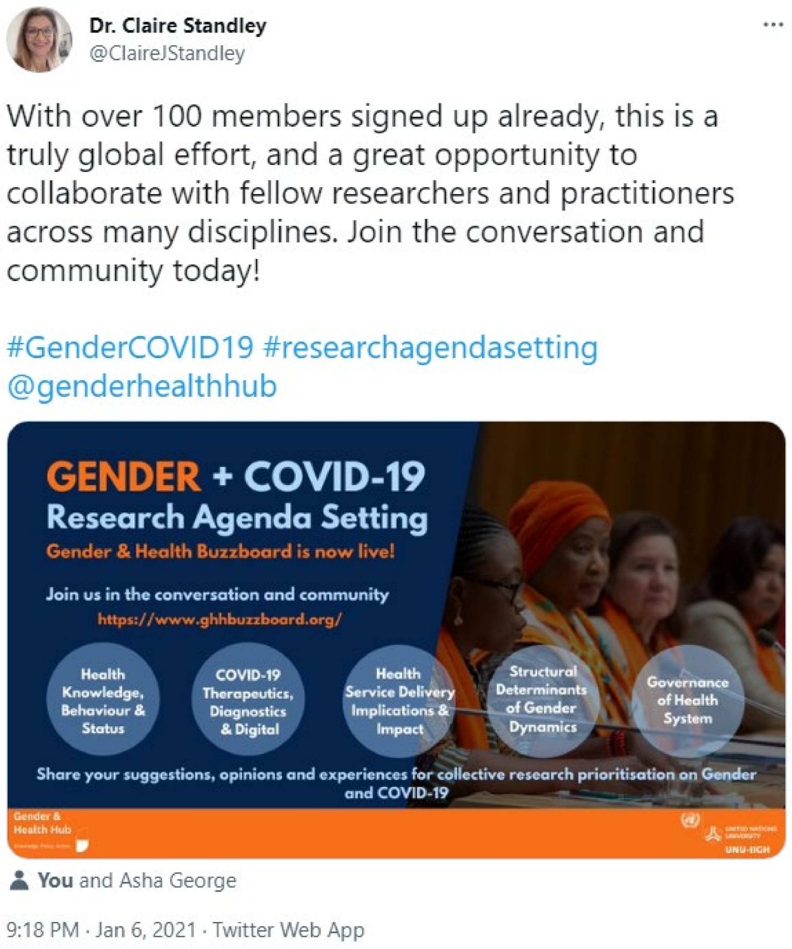

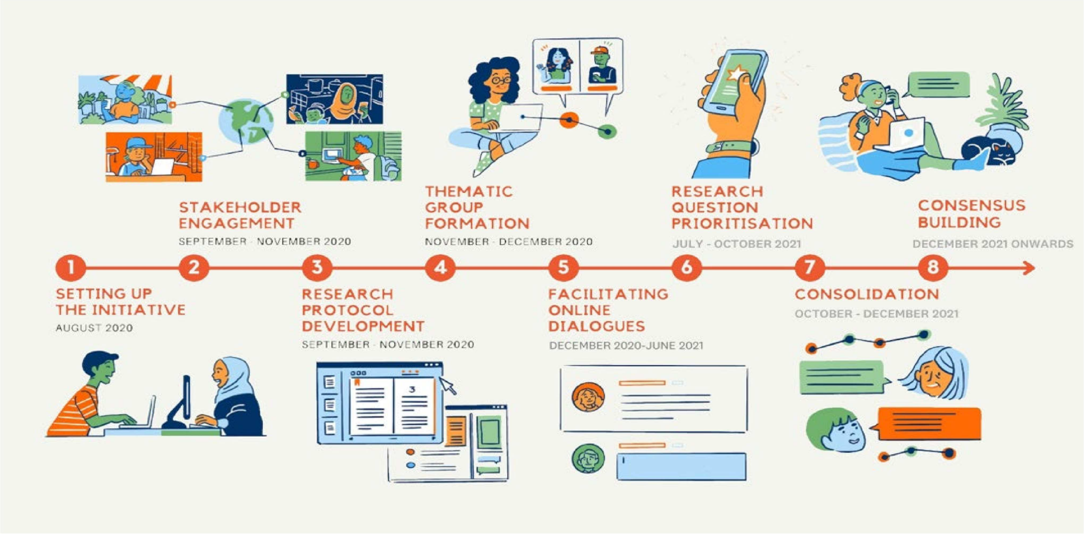

Voices articulating research priorities

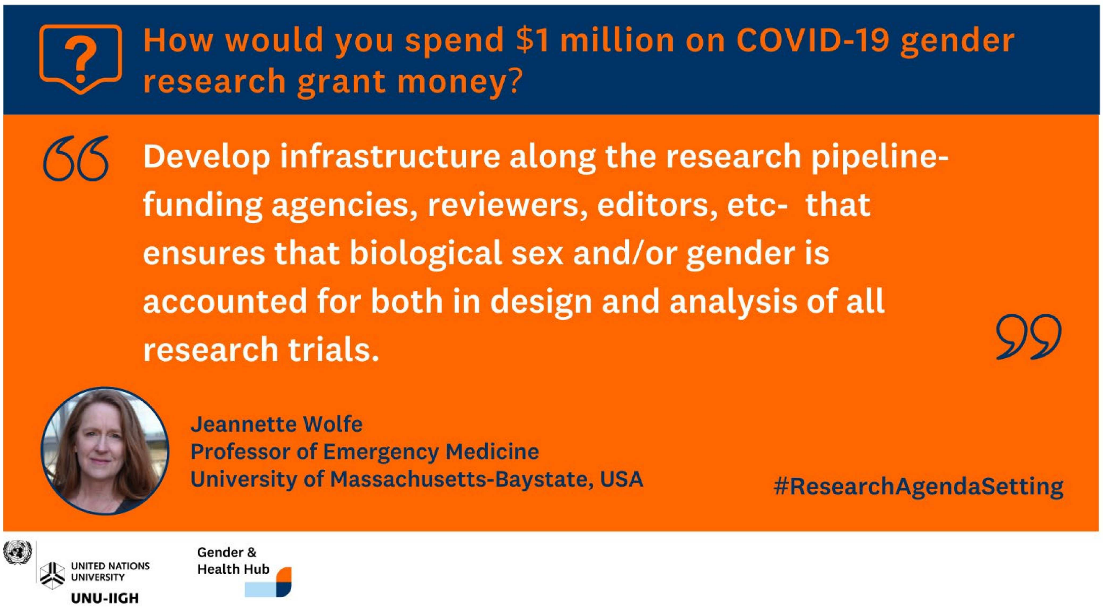
How would you spend $1 million on COVID-19 gender research grant money?
Develop infrastructure along the research pipeline- funding agencies, reviewers, editors, etc- that ensures that biological sex and/or gender is accounted for both in design and analysis of all research trials.
Jeannette Wolfe
Professor of Emergency Medicine
University of Massachusetts-Baystate, USA
#ResearchAgendaSetting
UNITED NATIONS UNIVERSITY
UNU-IIGH
Gender & Health Hub

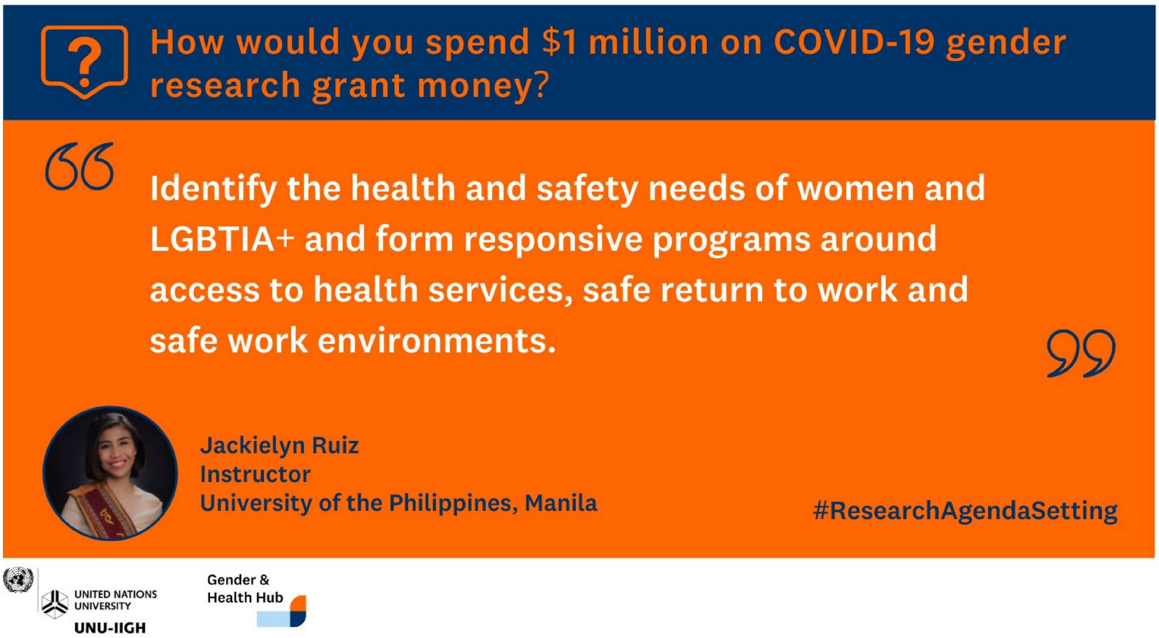
How would you spend $1 million on COVID-19 gender research grant money?
Identify the health and safety needs of women and LGBTIA+ and form responsive programs around access to health services, safe return to work and safe work environments.
Jackielyn Ruiz
Instructor
University of the Philippines, Manila
#ResearchAgendaSetting
UNITED NATIONS UNIVERSITY
UNU-IIGH
Gender & Health Hub

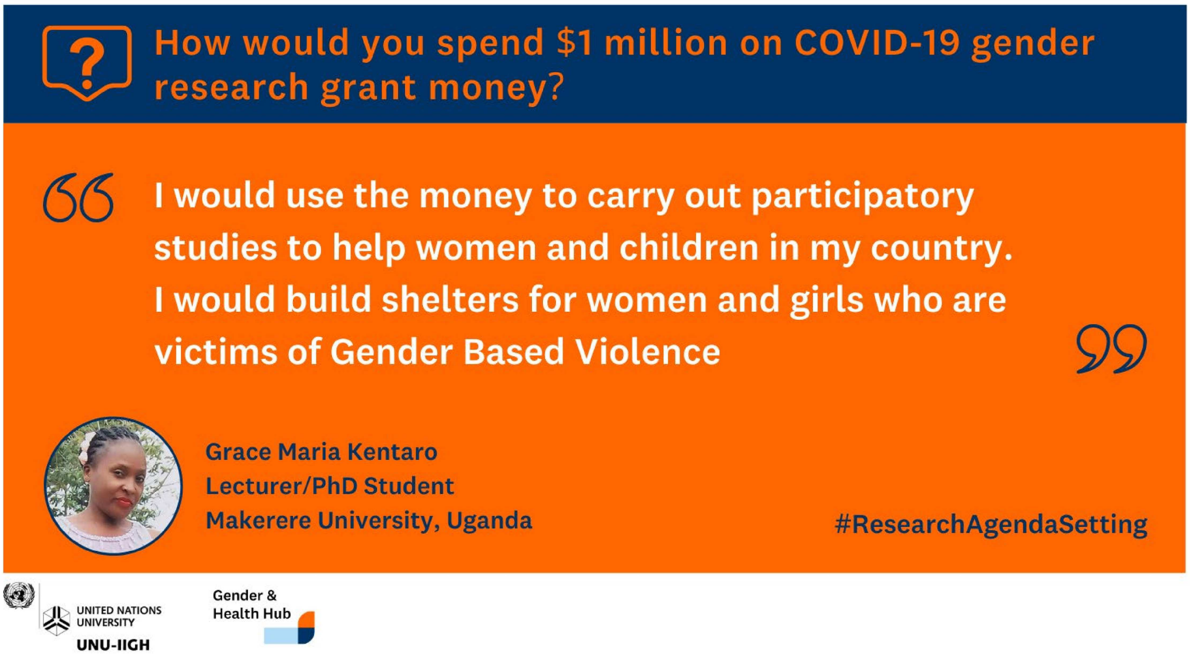
How would you spend $1 million on COVID-19 gender research grant money?
I would use the money to carry out participatory studies to help women and children in my country. I would build shelters for women and girls who are victims of Gender Based Violence
Grace Maria Kentaro
Lecturer/PhD Student
Makerere University, Uganda
#ResearchAgendaSetting
UNITED NATIONS UNIVERSITY
UNU-IIGH
Gender & Health Hub

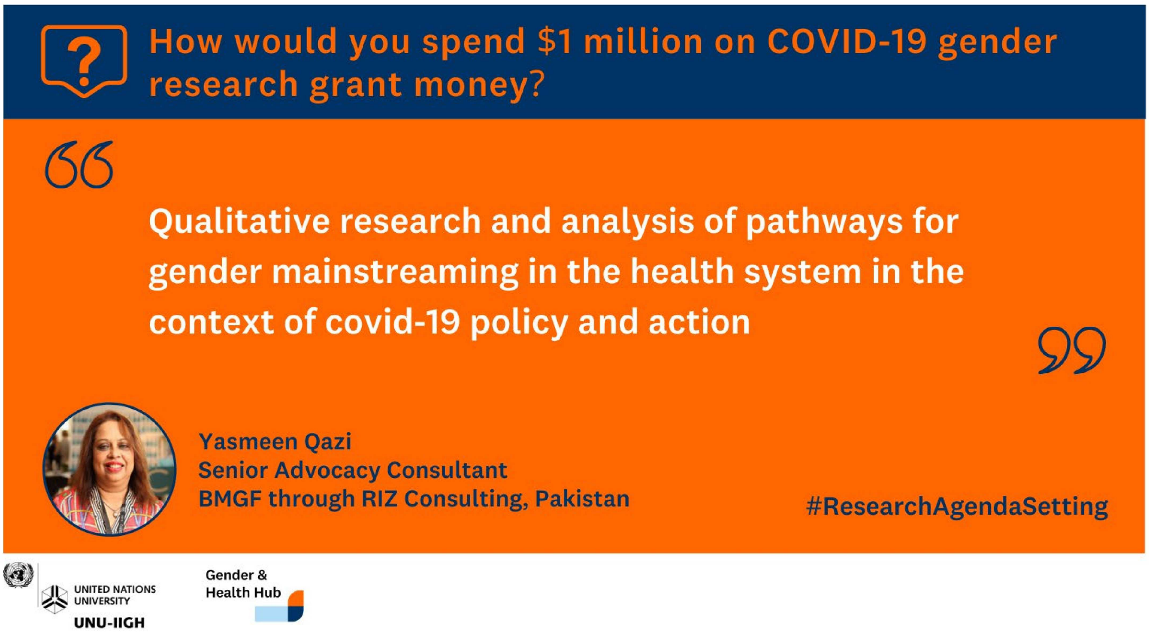
How would you spend $1 million on COVID-19 gender research grant money?
Qualitative research and analysis of pathways for gender mainstreaming in the health system in the context of covid-19 policy and action
Yasmeen Qazi
Senior Advocacy Consultant
BMGF through RIZ Consulting, Pakistan
#ResearchAgendaSetting
UNITED NATIONS UNIVERSITY
UNU-IIGH
Gender & Health Hub

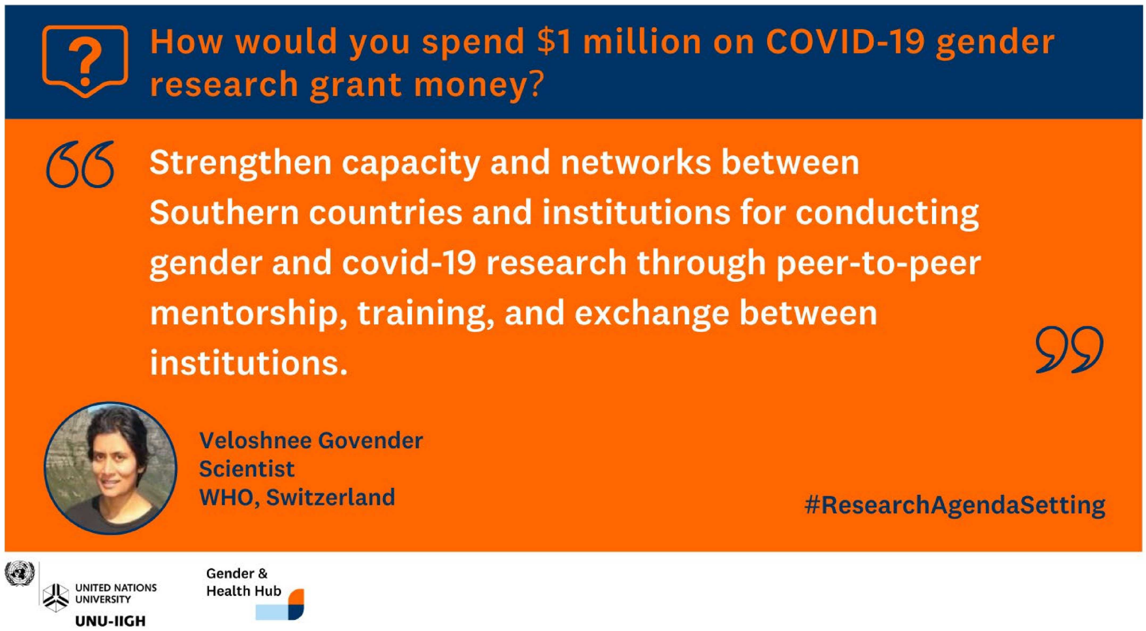
How would you spend $1 million on COVID-19 gender research grant money?
Strengthen capacity and networks between Southern countries and institutions for conducting gender and covid-19 research through peer-to-peer mentorship, training, and exchange between institutions.
Veloshnee Govender
Scientist
WHO, Switzerland
#ResearchAgendaSetting
UNITED NATIONS UNIVERSITY
UNU-IIGH
Gender & Health Hub

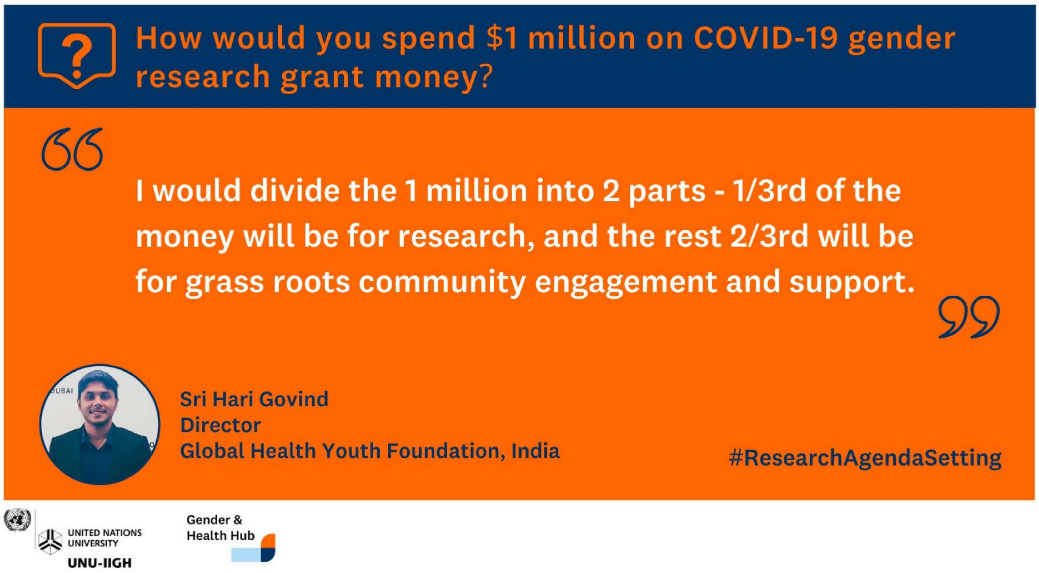
How would you spend $1 million on COVID-19 gender research grant money?
I would divide the 1 million into 2 parts - 1/3rd of the money will be for research, and the rest 2/3rd will be for grass roots community engagement and support.
Sri Hari Govind
Director
Global Health Youth Foundation, India
#ResearchAgendaSetting
UNITED NATIONS UNIVERSITY
UNU-IIGH
Gender & Health Hub

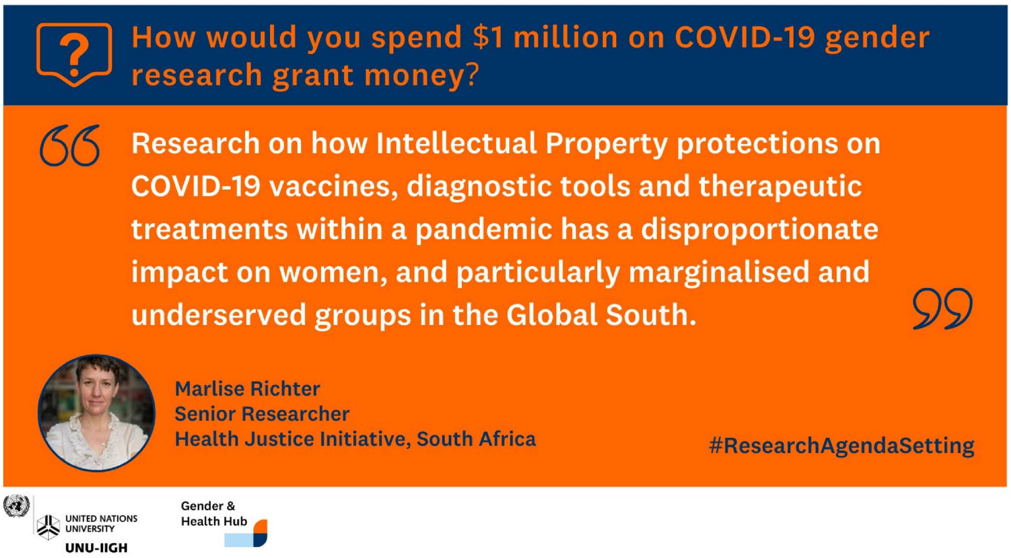

Regional collaborators encouraging participation

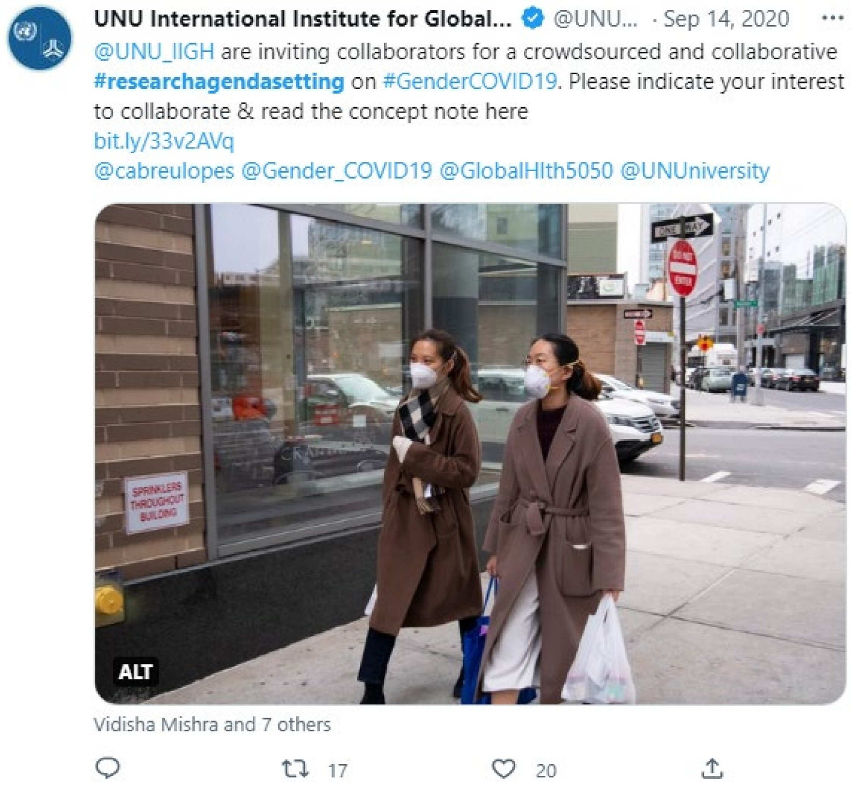

Gender & Health Hub
@genderhealthhub

📡 @VianaMarisa, @RESURJ convida vozes de países lusófonos a participar na @genderhealthhub #GéneroCOVID19 #InquéritoPriorizaçãoAgendaInvestigação!

⏱️ Data limite: Quinta-feira, 30 de Setembro
🔗 Link para o inquérito: go.unu.edu/ePHMr

Translate Tweet

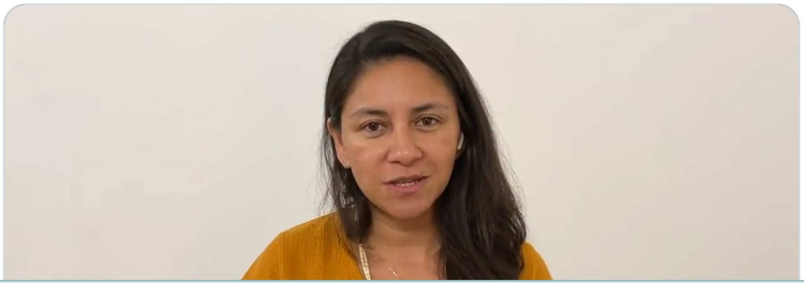

← **Tweet**

📡 Dr Kéfilath Bello, @cerrhud appelle les diverses voix francophones à participer à l'Enquête de Priorisation de la recherche sur le genre et la COVID-19.

#GenderCOVID19 #ResearchAgendaSetting

⏱️ Date limite : jeudi 30 septembre
🔗 Lien vers l'enquête: go.unu.edu/sejdM

Translate Tweet

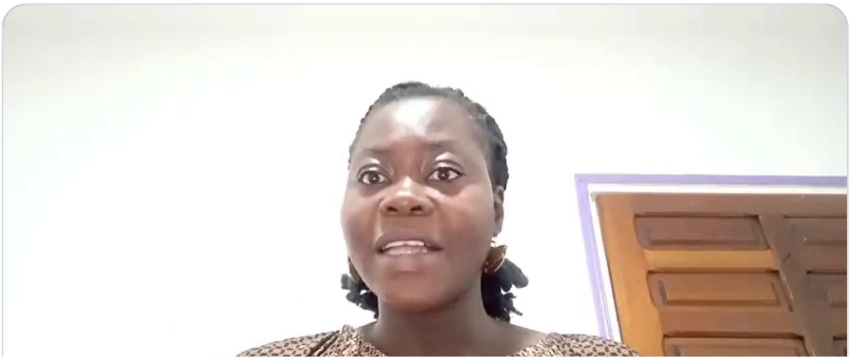

Lavanya Vijayasingham @lavanyav149 · Oct 9, 2020

Update: **#researchagendasetting** on #GenderCOVID19 Stakeholder reach: over 60% interest from LMICs! shorturl.at/irABG Call still open, protocol coming soon! @cabreulopes
The concept note: bit.ly/3bWF4Va
Tell us you're interested here: bit.ly/33v2AVq

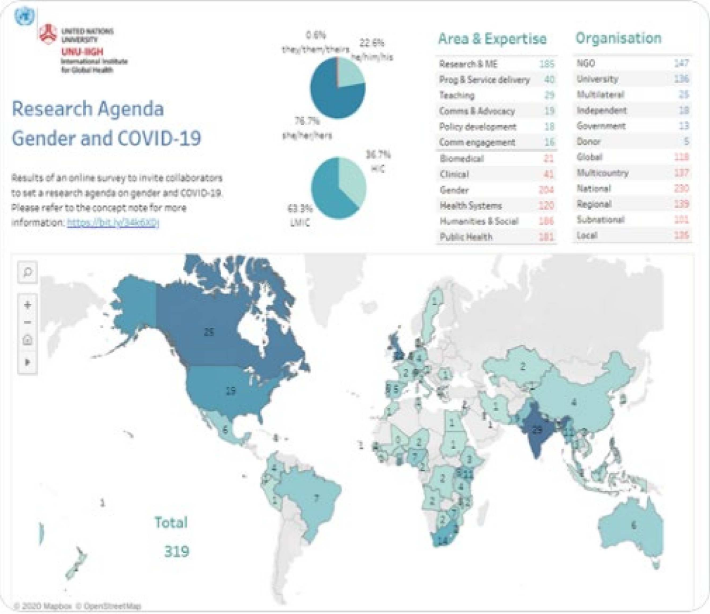

UNU International Institute for Global Health and 5 others

9 12

Raul Mercer @FLACSOARGENTINA llama a diversas voces en América Latina y el Caribe a participar en las encuestas del @genderhealthhub para establecer una agenda de investigación solidaria para #GéneroCovid19!
⏱Fecha límite:Jueves 30 de Septiembre
🔗Enlace: go.unu.edu/vyufl

Translate Tweet

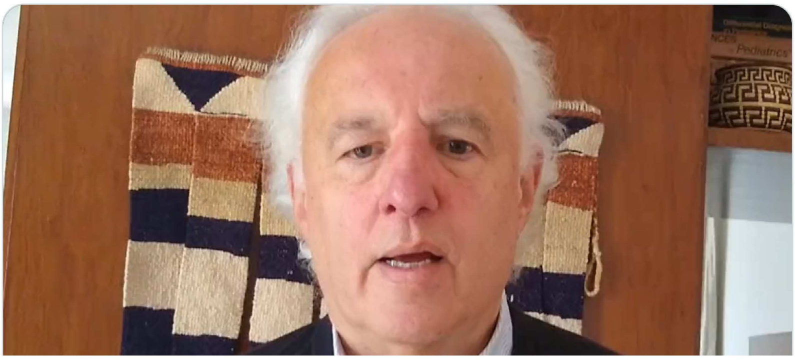

Gender & Health Hub @genderhealthhub · Dec 3, 2020

Our research agenda-setting exercise for #gender and #COVID19 involves 470 stakeholders from across the world, with over 66% representation from LMICs.

Get to know our community and join us: go.unu.edu/pk7Rd
#researchagendasetting

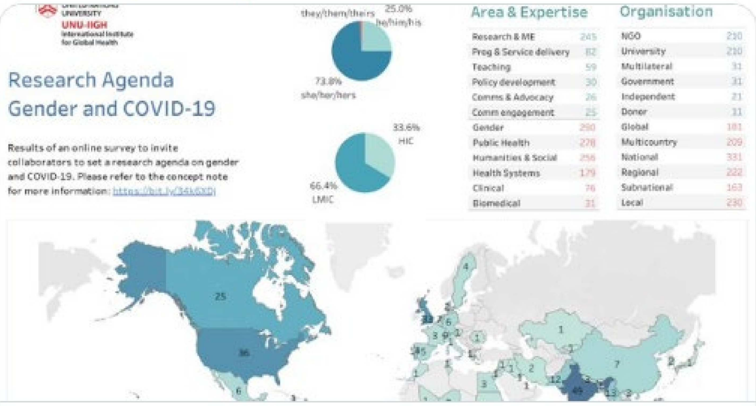

public.tableau.com
Research Agenda Setting (EoI)
Research agenda-setting - results of the expression of interest questionnaire

4 · 6

Gender & Health Hub @genderhealthhub · Jan 12, 2021

We want to hear your inputs on social and structural determinants of gender dynamics affecting or impacted by COVID-19!

Go to #GBV and #COVID19 on thematic group 4 #buzzboard here ➡ go.unu.edu/oNnsN

#genderhealthhub #infographics **#ResearchAgendaSetting** #GenderCOVID19

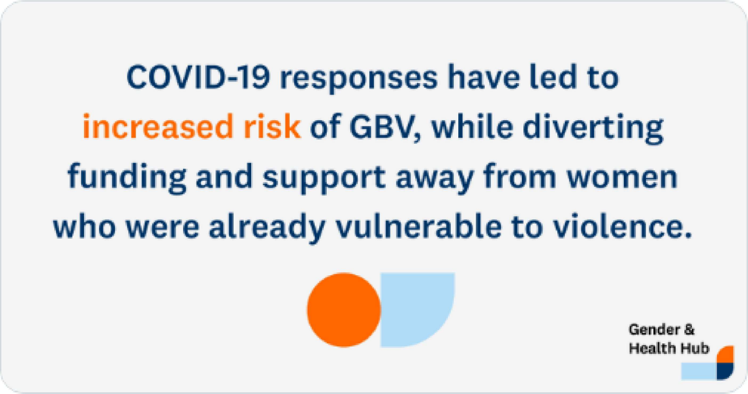

1

Gender & Health Hub @genderhealthhub · Jan 13, 2021

#GenderCOVID19 research should be based on global and nuanced needs.

Researchers, let's talk to one another about priorities on next steps, on our website: ghhbuzzboard.org

#ResearchAgendaSetting

Image: Locations of unique visits.

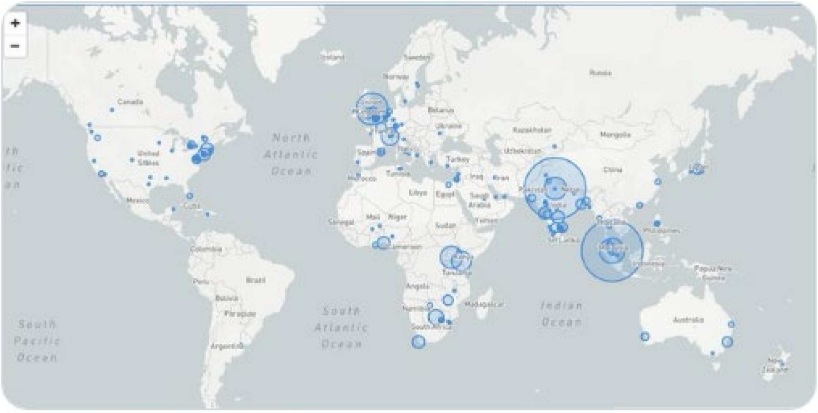

Alyson J. McGregor and 9 others

1 7 12

Gender & Health Hub
@genderhealthhub

📟 Launch Event of the GHH Buzzboard for #GenderCOVID19 and #ResearchAgendaSetting: Info session and live thematic group discussions.

📝 Sign up here to join the discussion: go.unu.edu/yQd69

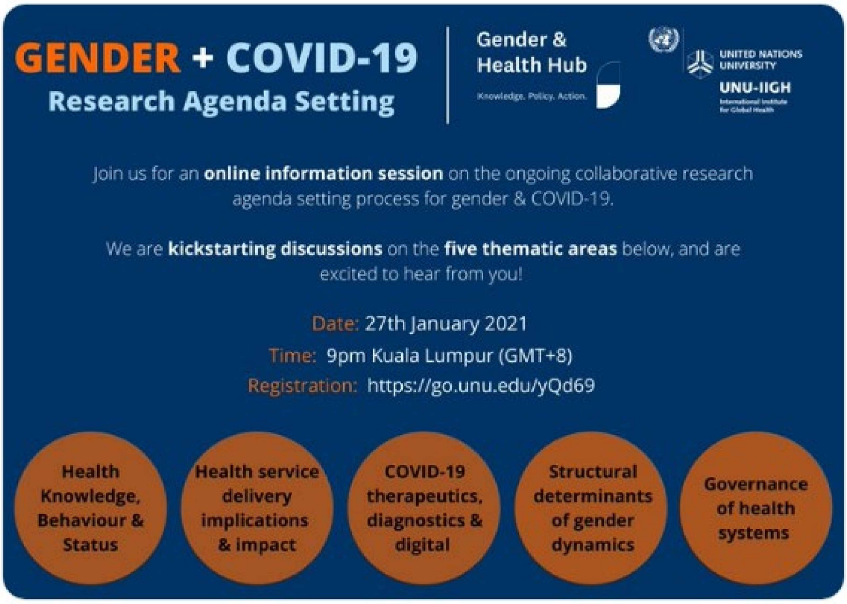

UNU International Institute for Global Health and 4 others

10:24 PM · Jan 25, 2021 · Twitter Web App

View Tweet analytics

6 Retweets **1** Quote Tweet **8** Likes

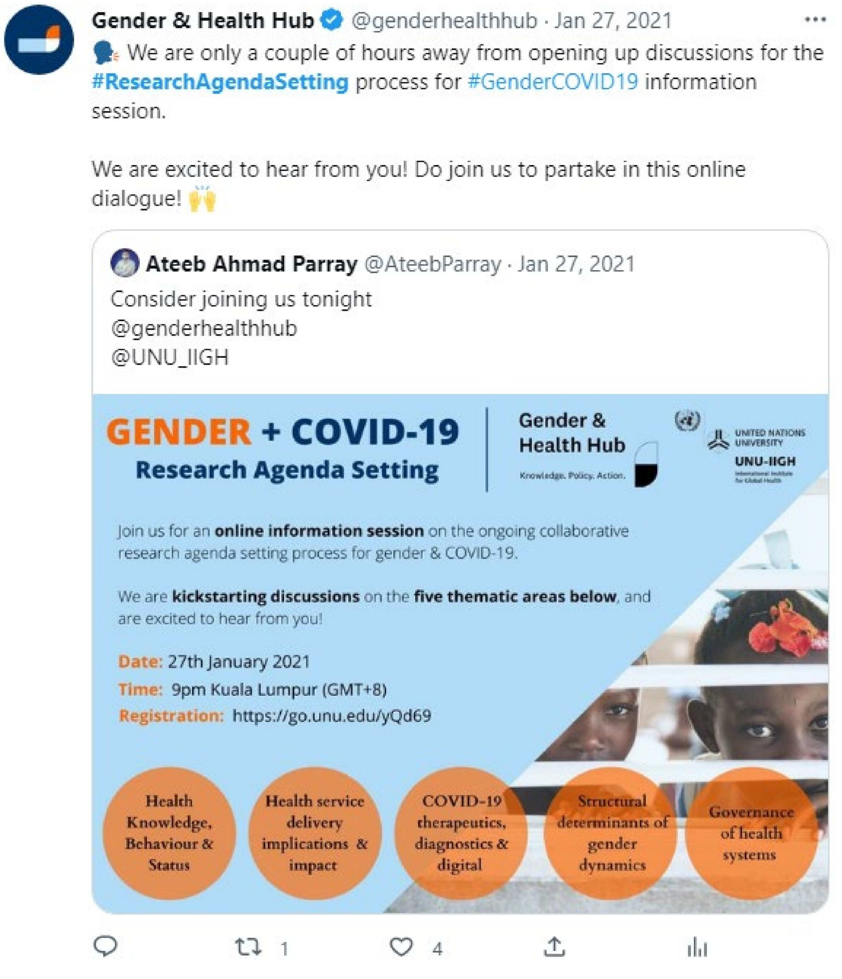
Gender & Health Hub @genderhealthhub · Jan 27, 2021
We are only a couple of hours away from opening up discussions for the #ResearchAgendaSetting process for #GenderCOVID19 information session.
We are excited to hear from you! Do join us to partake in this online dialogue!
Ateeb Ahmad Parray @AteebParray · Jan 27, 2021
Consider joining us tonight
@genderhealthhub
@UNU_IIGH
GENDER + COVID-19
Research Agenda Setting
Gender & Health Hub
Knowledge. Policy. Action.
UNITED NATIONS UNIVERSITY
UNU-IIGH
Join us for an online information session on the ongoing collaborative research agenda setting process for gender & COVID-19.
We are kickstarting discussions on the five thematic areas below, and are excited to hear from you!
Date: 27th January 2021
Time: 9pm Kuala Lumpur (GMT+8)
Registration: https://go.unu.edu/yQd69
Health Knowledge, Behaviour & Status
Health service delivery implications & impact
COVID-19 therapeutics, diagnostics & digital
Structural determinants of gender dynamics
Governance of health systems
1
4

Lavanya Vijayasingham @lavanyav149 · Jan 29, 2021

What a great call & vibrant conversation on the 5 thematic areas for #GenderCOVID19 **#ResearchAgendaSetting**!

Big thank you to all who joined!

📺Here are the recordings: go.unu.edu/Sv1jH

✍ Post priority research questions on ghhbuzzboard.org before 15 Feb 2021!

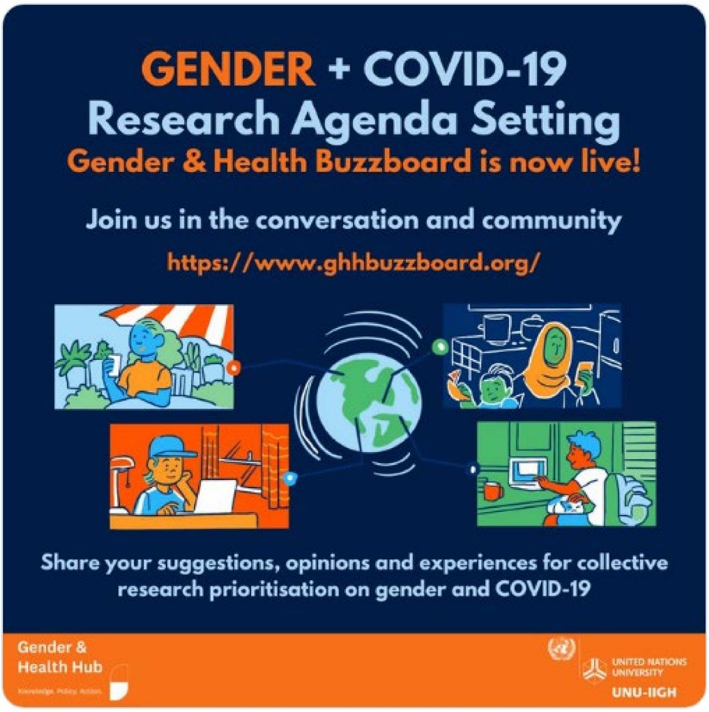

You and 6 others

6 6

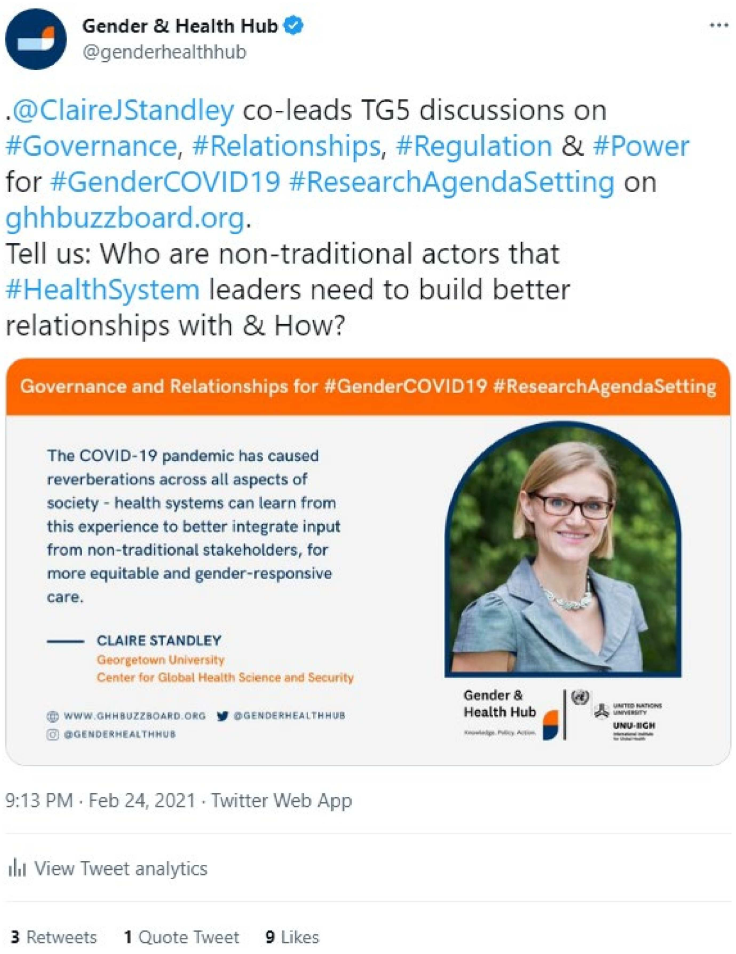
Gender & Health Hub
@genderhealthhub

.@ClaireJStandley co-leads TG5 discussions on #Governance, #Relationships, #Regulation & #Power for #GenderCOVID19 #ResearchAgendaSetting on ghhbuzzboard.org.
Tell us: Who are non-traditional actors that #HealthSystem leaders need to build better relationships with & How?

9:13 PM · Feb 24, 2021 · Twitter Web App

View Tweet analytics

3 Retweets **1** Quote Tweet **9** Likes

Gender & Health Hub
@genderhealthhub

@RosemaryJMorgan, co-lead of Thematic Grp 3 on Health Service Delivery in the #GenderCOVID19 #ResearchAgendaSetting initiative shares with us why strategic research is needed.

Tell us what research you think is important on ghhbuzzboard.org!

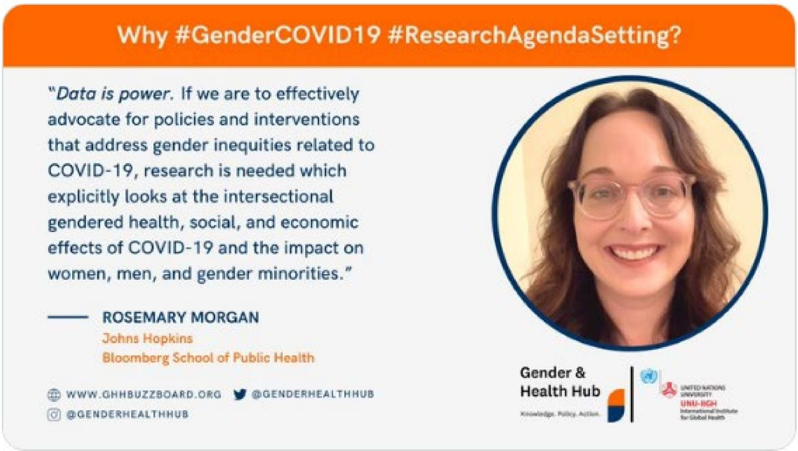

7:55 PM · Feb 22, 2021 · Twitter Web App

View Tweet analytics

11 Retweets **1** Quote Tweet **12** Likes

Gender & Health Hub @genderhealthhub · Mar 5, 2021

Nearly 400 people have signed up to #GenderCovid19 **#ResearchAgendaSetting**! 💡

✍️ Members are also sharing their thoughts on skills that we, in gender and health, should master.

What are your thoughts? 💭

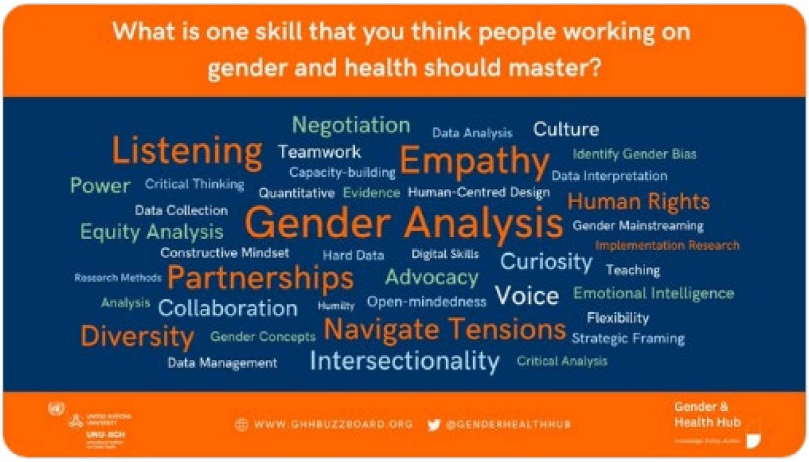

Pascale and 5 others

11 18

Gender & Health Hub @genderhealthhub · May 13, 2021

Contributing to the WHO COVID-19 Research Road Map?

Follow us for more ▶️ ghhbuzzboard.org

#GenderAndCOVID19 **#ResearchAgendaSetting** #BetterCOVIDScience

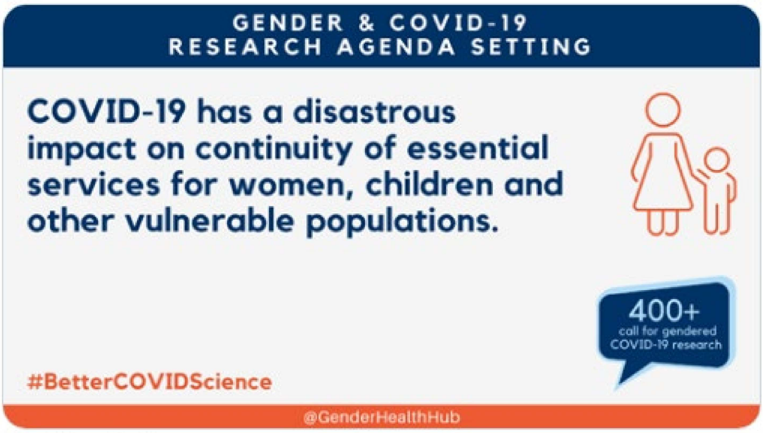

PMNCH and 9 others

1 6 10

Gender & Health Hub @genderhealthhub · May 14, 2021

Contributing to the WHO COVID-19 Research Road Map?

Follow us for more ▶ ghhbuzzboard.org

#GenderAndCOVID19 **#ResearchAgendaSetting** #BetterCOVIDScience

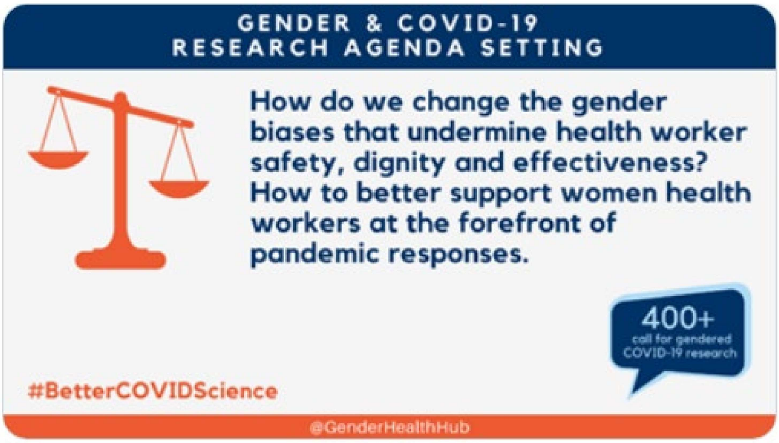

Jim Campbell and 3 others

1 5 4

Gender & Health Hub @genderhealthhub · May 14, 2021

Contributing to the WHO COVID-19 Research Road Map?

Follow us for more ▶ ghhbuzzboard.org

#GenderAndCOVID19 **#ResearchAgendaSetting** #BetterCOVIDScience

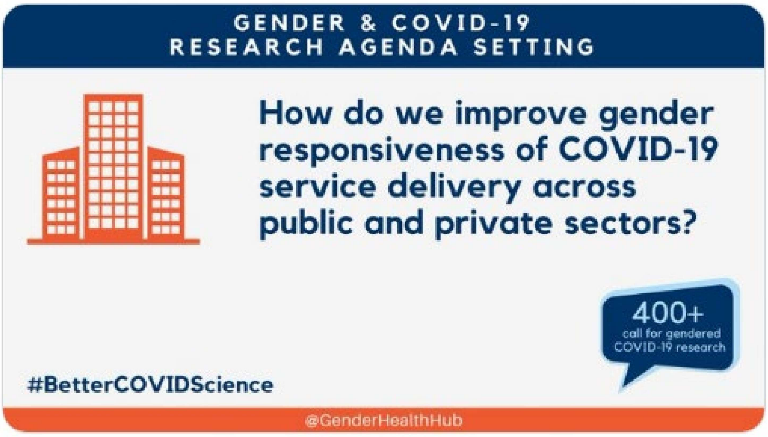

Dr Agnes Soucat and 2 others

2 5

Gender & Health Hub @genderhealthhub · May 15, 2021

Contributing to the WHO COVID-19 Research Road Map?

Follow us for more ▶ ghhbuzzboard.org

#GenderAndCOVID19 **#ResearchAgendaSetting** #BetterCOVIDScience

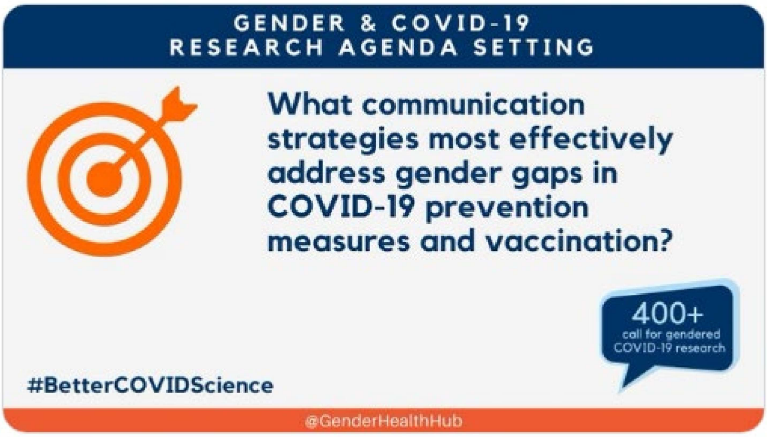

Tina D Purnat and 4 others

2 4

Gender & Health Hub @genderhealthhub · May 15, 2021

Day 2 | @WHO #RDBlueprint for Epidemics #COVID19 Global Research & Innovation Forum

Visit our page for key messages from the #GenderCovid19 **#ResearchAgendaSetting** process

🌍What do 400+ stakeholders call for?

#BetterCOVIDScience

@ashageorge72 @lavanyav149 @PascaleAllotey

> **Gender & Health Hub** @genderhealthhub · May 13, 2021
>
> Contributing to the WHO COVID-19 Research Road Map?
>
> Follow us for more ▶️ ghhbuzzboard.org
>
> #GenderAndCOVID19 #ResearchAgendaSetting #BetterCOVIDScience

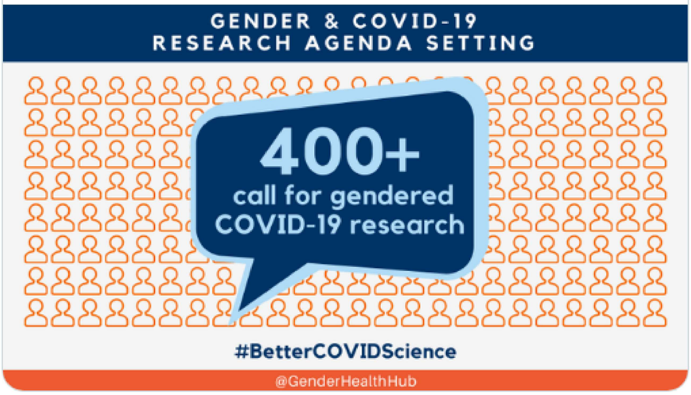

3 4

Gender & Health Hub @genderhealthhub · Jun 14, 2021

Join us, @aphrc, @UNU_IIGH, @UWConline & @IDRC_CRDI for the Gender & COVID-19 Research Agenda-Setting: Consultation for Central & West Africa event.

15 June, Tue
11am GMT | 12pm CAT

Register: go.unu.edu/tjd3X

In ENG & FRENCH

#ResearchAgendaSetting

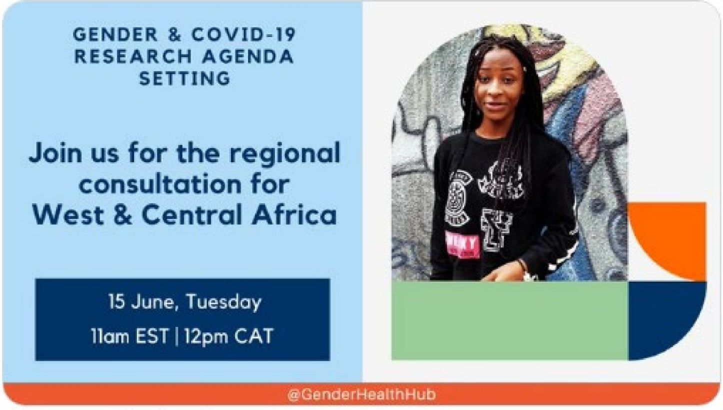

Africa Data Hub and 8 others

4 6

Gender & Health Hub @genderhealthhub · Jun 8, 2021

Join us, @UNU_IIGH, @Ai4Women & @ABAADMENA for the Gender & COVID-19 Research Agenda-Setting: Consultation for the Middle East East & North Africa.

9 June, Wed
Register: genderhealthhub.org/articles/consu...

#ResearchAgendaSetting
@LinaAbiRafeh @MimiSfeir @anthony_keedi @JenniferSkulte

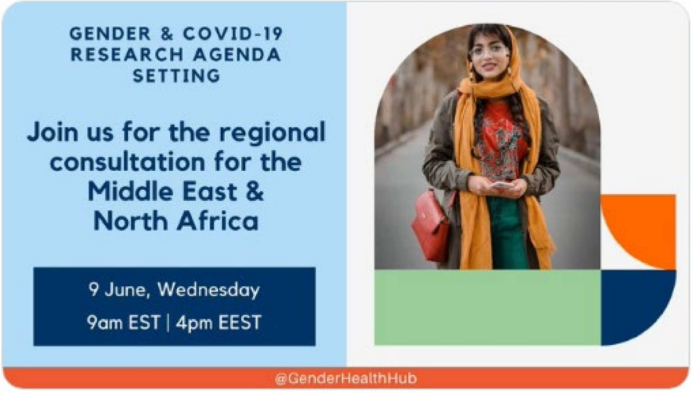

Jill Filipovic and 9 others

6 6

Gender & Health Hub @genderhealthhub · Jul 2, 2021

1. You can learn more about our global collaborative #ResearchAgendaSetting for #GenderCOVID19 here 💻

Solidarity ➡️ Scale ➡️ Impact

Survey: ghhbuzzboard.org

@ashageorge72 @SOPHUWC @DrMichelleRemme @lavanyav149

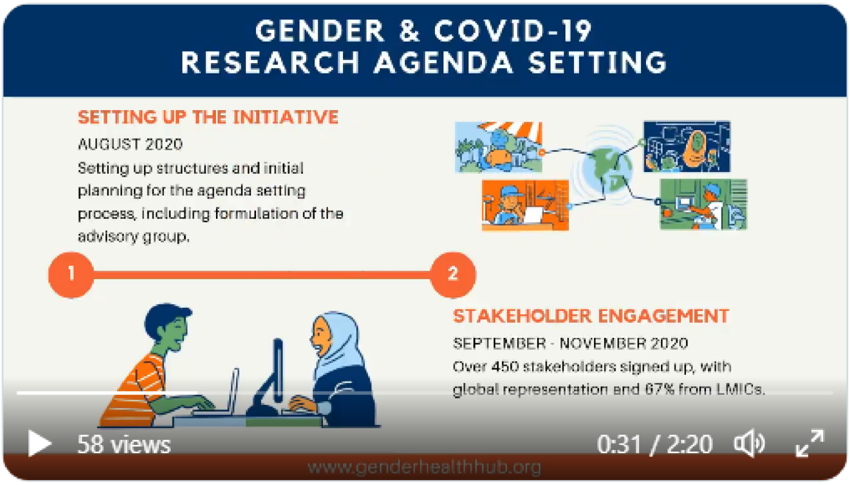

1 3 4

Gender & Health Hub @genderhealthhub · Jul 13, 2021

❗♀🦠Emerging priority questions for Gender, COVID-19 & Health Service Delivery - Do you agree or disagree?

Check your email for your personalised link or email agendasetting@unu.edu

#GenderCOVID19 **#ResearchAgendaSetting**
@PamojaUK @mamothena

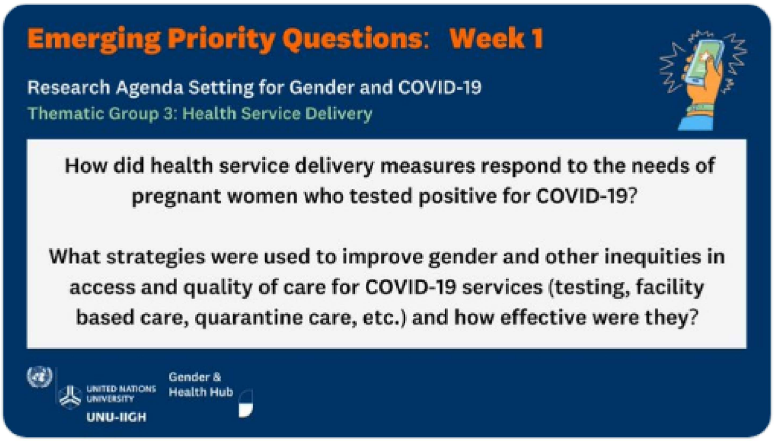

UNU International Institute for Global Health and 6 others

3 6

Gender & Health Hub @genderhealthhub · Jul 12, 2021

❗♀🦠Emerging priority questions for Gender in COVID-19 Governance - Do you agree or disagree?

#PatentSuspension for #Tocilizumab in #LMICs is a good example

Check your email for your personalised link or email agendasetting@unu.edu

#GenderCOVID19 **#ResearchAgendaSetting**

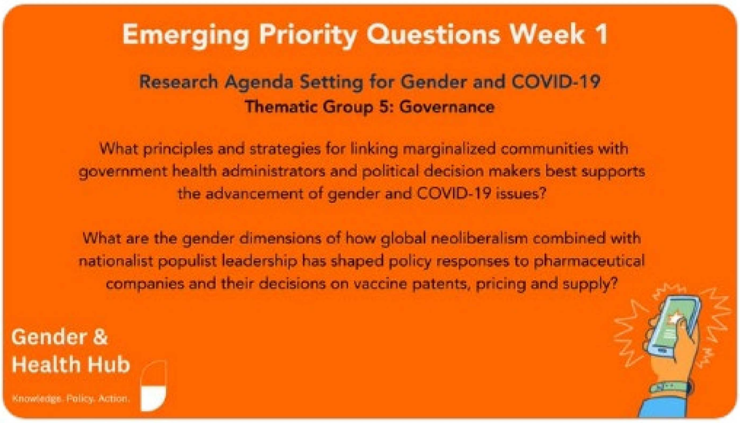

UNU International Institute for Global Health and 8 others

8 8

Gender & Health Hub @genderhealthhub · Jul 9, 2021

💡 Emerging priority questions for Sex and Gender in COVID-19 Therapeutics & Diagnostics - Do you agree or disagree?

📝To complete the questionnaire: Check your email for your personalised link or email agendasetting@unu.edu

#GenderCOVID19 #ResearchAgendaSetting

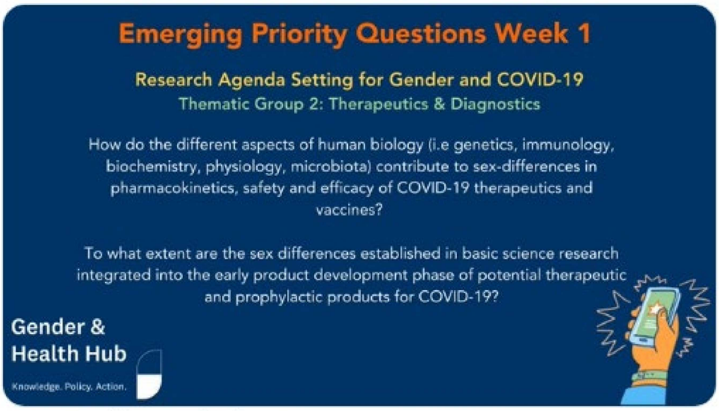

Jeannette Wolfe MD and 7 others

6 10

Gender & Health Hub @genderhealthhub · Sep 1, 2021

📡📡 We are now live with the Gender and Health Research Agenda Setting for COVID-19: Initial Results session!

Join us, as our participants build consensus on research priorities to address a gender transformative health sector response to COVID-19.

#ResearchAgendaSetting

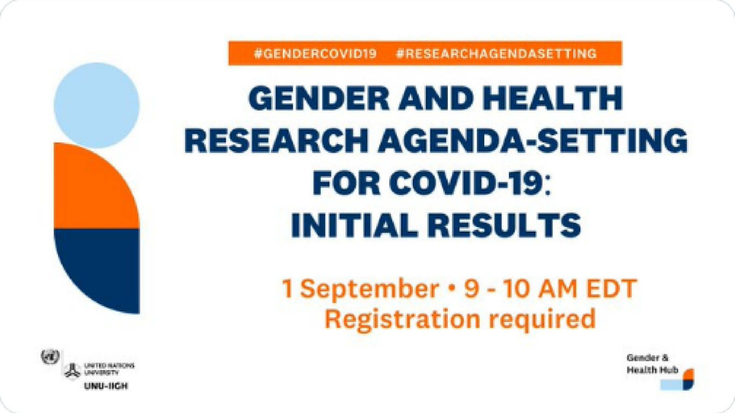

1

Gender & Health Hub @genderhealthhub · Sep 1, 2021

💭Join us in a collaborative gender & COVID-19 RAS exercise for the Gender & Health #ResearchAgendaSetting for COVID-19: Initial Results Webinar!

Introducing members of TG 5👇
@ashageorge72
@UWConline
@mmabzy
@Afrolutionist
@asoucat
@AFD_France

Register👉go.unu.edu/iNsih

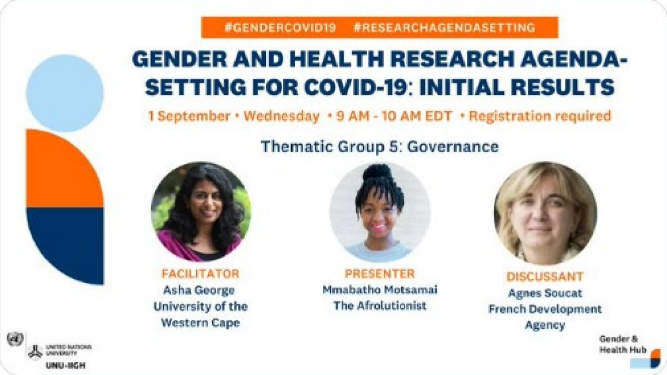

1 5 7

Gender & Health Hub @genderhealthhub · Aug 30, 2021

Join us in a collaborative gender & COVID-19 RAS exercise for the Gender & Health #ResearchAgendaSetting for COVID-19: Initial Results Webinar!

Members of TG 3

@PascaleAllotey
@UNU_IIGH
Sedighe Hosseinijebeli, IUMS
@jeanvegamorales
@Fonasa

Register go.unu.edu/iNsih

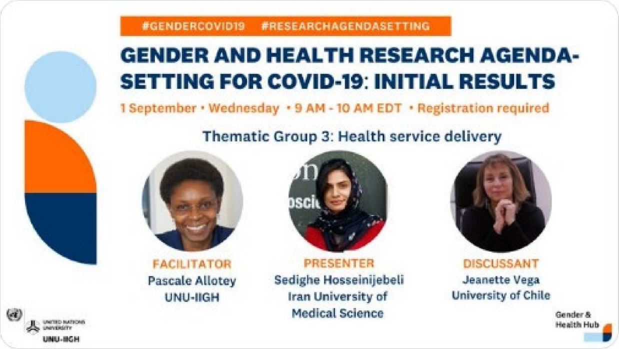

11 16

Gender & Health Hub @genderhealthhub · Aug 30, 2021

Join us in a collaborative gender & COVID-19 RAS exercise for the Gender & Health #ResearchAgendaSetting for COVID-19: Initial Results Webinar!

Introducing members of TG 1

@cabreulopes
@UNU_IIGH
@mishra_gita
@UQ_News
@TDPurnat
@WHO

Register go.unu.edu/iNsih

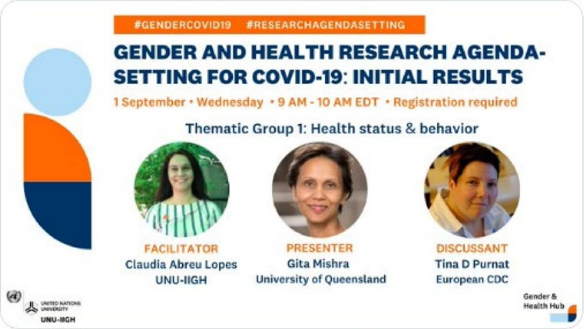

3 9

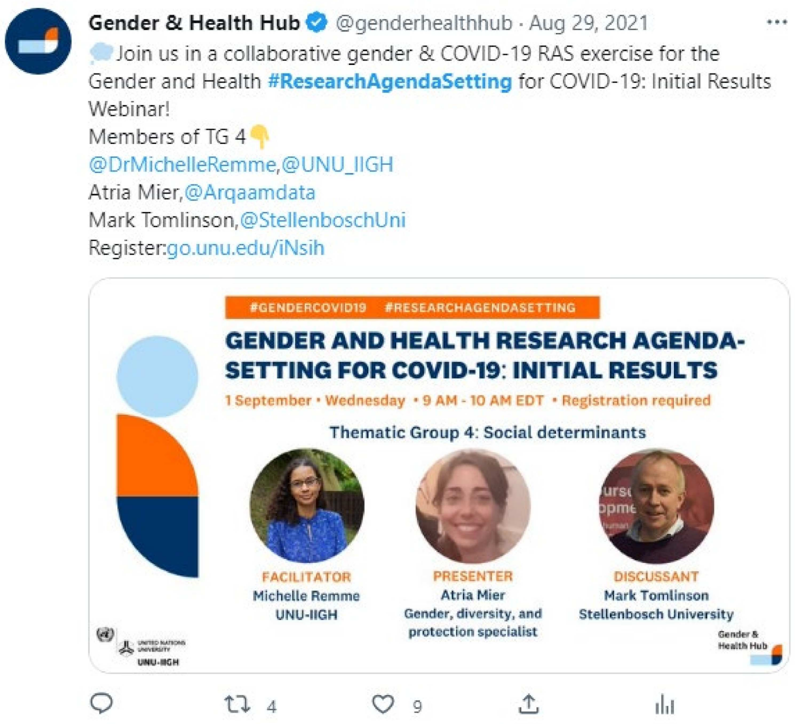
Gender & Health Hub @genderhealthhub · Aug 29, 2021
Join us in a collaborative gender & COVID-19 RAS exercise for the Gender and Health #ResearchAgendaSetting for COVID-19: Initial Results Webinar!
Members of TG 4
@DrMichelleRemme,@UNU_IIGH
Atria Mier,@Arqaamdata
Mark Tomlinson,@StellenboschUni
Register:go.unu.edu/iNsih
#GENDERCOVID19 #RESEARCHAGENDASETTING
GENDER AND HEALTH RESEARCH AGENDA-SETTING FOR COVID-19: INITIAL RESULTS
1 September • Wednesday • 9 AM - 10 AM EDT • Registration required
Thematic Group 4: Social determinants
FACILITATOR
Michelle Remme
UNU-IIGH
PRESENTER
Atria Mier
Gender, diversity, and protection specialist
DISCUSSANT
Mark Tomlinson
Stellenbosch University
UNU-IIGH
Gender & Health Hub
4
9

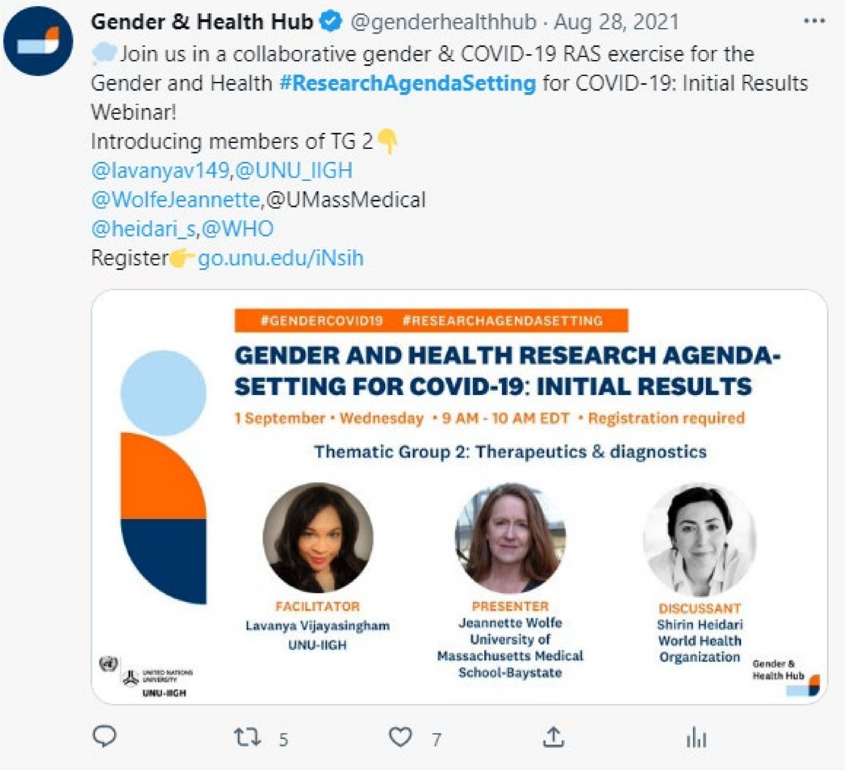
Gender & Health Hub @genderhealthhub · Aug 28, 2021
Join us in a collaborative gender & COVID-19 RAS exercise for the Gender and Health #ResearchAgendaSetting for COVID-19: Initial Results Webinar!
Introducing members of TG 2
@lavanyav149,@UNU_IIGH
@WolfeJeannette,@UMassMedical
@heidari_s,@WHO
Register go.unu.edu/iNsih
#GENDERCOVID19 #RESEARCHAGENDASETTING
GENDER AND HEALTH RESEARCH AGENDA-SETTING FOR COVID-19: INITIAL RESULTS
1 September • Wednesday • 9 AM - 10 AM EDT • Registration required
Thematic Group 2: Therapeutics & diagnostics
FACILITATOR
Lavanya Vijayasingham
UNU-IIGH
PRESENTER
Jeannette Wolfe
University of Massachusetts Medical School-Baystate
DISCUSSANT
Shirin Heidari
World Health Organization
UNU-IIGH
Gender & Health Hub
5
7

Gender & Health Hub @genderhealthhub · Oct 8, 2021

💡 REMINDER | The English version of the #ResearchAgendaSetting for #GenderCovid19 is now available!

Please participate by using this link: go.unu.edu/1FsUO

Or contact us at agendasetting@unu.edu

Deadline for participation is 15 October 2021

Now available in 🇵🇹 🇪🇸 🇫🇷

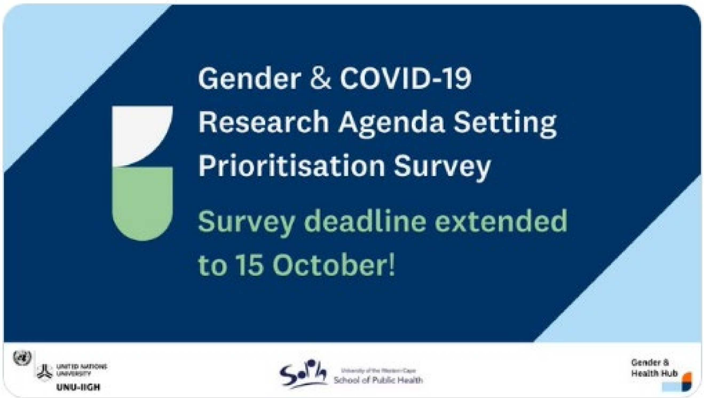

1 2 2

Gender & Health Hub @genderhealthhub · Sep 15, 2021

🗣️ Des voix diverses autour du monde ont répondu à l'enquête #ResearchAgendaSetting #GenderCovid19!

🌍Découvrez qui participe déjà, et participez aussi :

La date limite de participation à l'enquête : 30 septembre 2021.

go.unu.edu/FlcTW

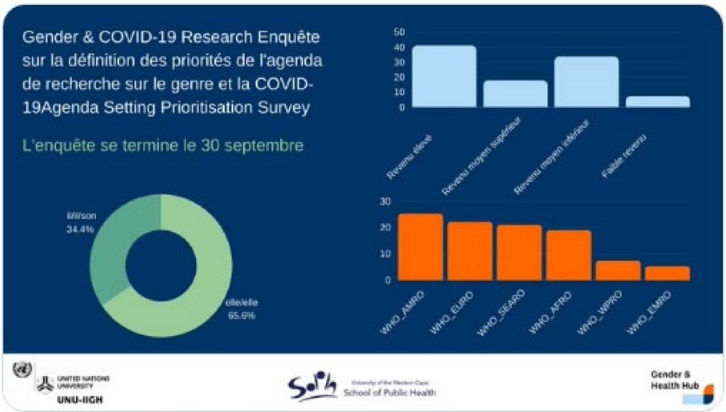

SMART Women's Initiative and 8 others

1 4 3

Gender & Health Hub @genderhealthhub · Oct 8, 2021

💡 RAPPEL | La version française de l'enquête #ResearchAgendaSetting pour #GenderCovid19 est maintenant disponible!

Veuillez participer en utilisant ce lien: go.unu.edu/sejdM

Ou contactez-nous à agendasetting@unu.edu

Date limite de participation : 15 octobre 2021

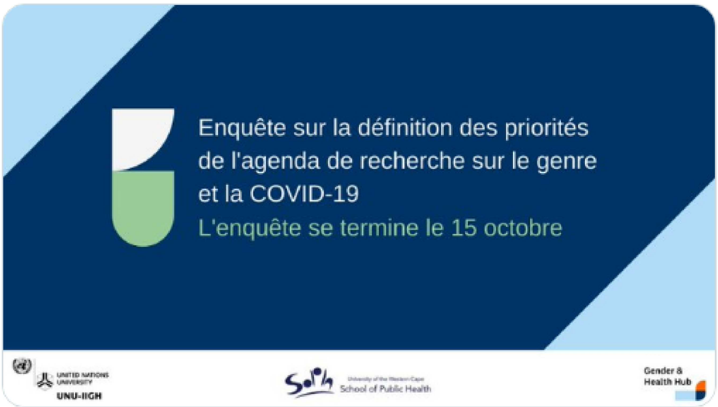

U of M Rady Faculty and 3 others

1 1

Gender & Health Hub @genderhealthhub · Oct 8, 2021

💡 LEMBRETE | A versão Português de #ResearchAgendaSetting para #GenderCovid19 já está disponível!

Use este link para participar: go.unu.edu/ePHMr

Ou contacte-nos em agendasetting@unu.edu

A data limite para participação na pesquisa é 15 de outubro de 2021.

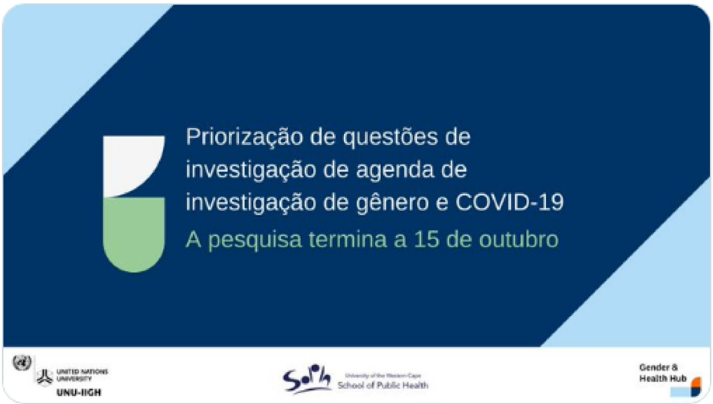

ULisboa and 2 others

1

Gender & Health Hub @genderhealthhub · Oct 8, 2021

💡 RECORDATORIO | ¡La versión en español de #EstablecimientoDeLaAgendaDeInvestigación para #GéneroCovid19 ya está disponible!
Por favor participe usando este enlace: go.unu.edu/vyufl
Contacto: agendasetting@unu.edu
La fecha límite para participar es el 15 de octubre de 2021

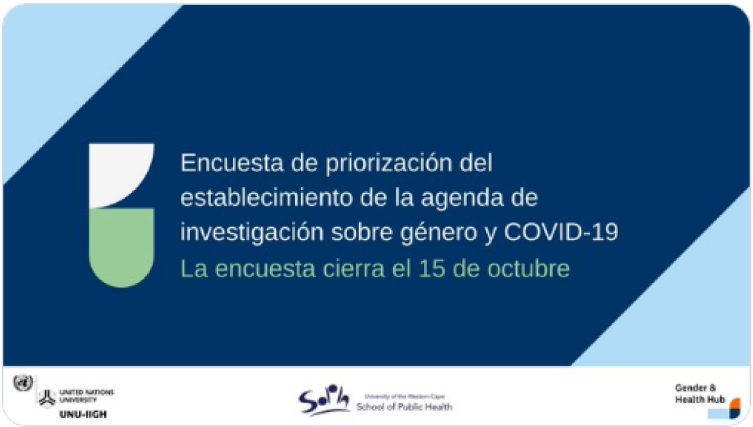

Universidad San Francisco de Quito and 3 others

Gender & Health Hub @genderhealthhub · Oct 13, 2021

🚨NEW | Reflections on emerging COVID-19 research priorities for gender and governance of health systems

✍By Manasee Mishra @ashageorge72 @ClaireJStandley @mmabzy @asoucat

Link ➡️ genderhealthhub.org/articles/refle...

#ResearchAgendaSetting #GenderCOVID19

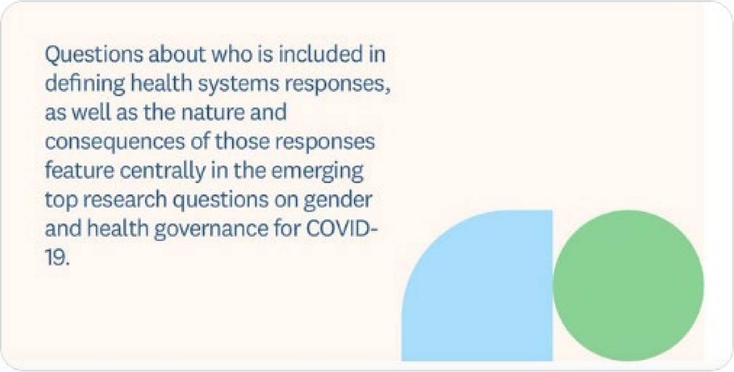

UN University and 9 others

8 7

UNU International Institute for Global... @UNU_... · Oct 21, 2021

The @genderhealthhub is now live with its 'Gender & COVID-19 Research Agenda Setting in Asia Pacific: Vaccinations' webinar!

🎥 Tune in to watch the live stream session here: go.unu.edu/ZRuQu.

#ResearchAgendaSetting #GenderCOVID19 #Fem4PeoplesVaccine

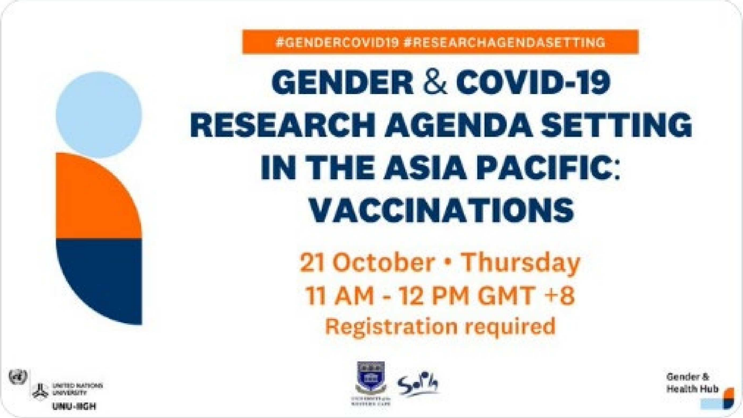

SOPH UWC and 2 others

1 1 2

Gender & Health Hub @genderhealthhub · Oct 20, 2021

Gender & COVID-19 Vaccination

🙋Join our webinar to learn strategies to promote & support research implementation of the collaboratively set agenda in #AsiaPacific

#GenderAndCOVID19 **#ResearchAgendaSetting**

📅 Thu, 21 Oct
⌚ 11am GMT

➡️ Register: go.unu.edu/QV0nZ

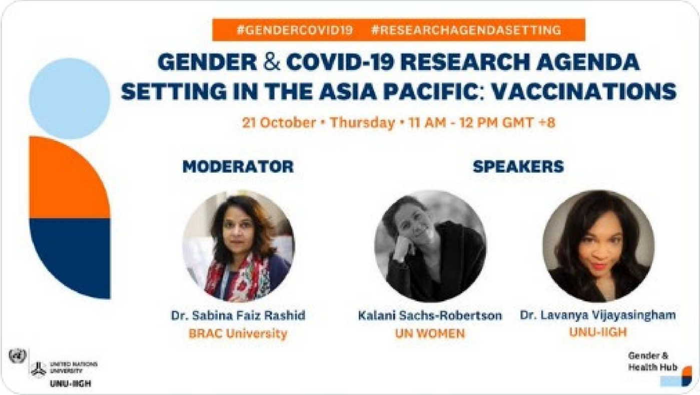

BRAC James P Grant School of Public Health and 6 others

2 2

Gender & Health Hub @genderhealthhub · Oct 20, 2021

Barriers to COVID-19 vaccination access & delivery:
❌Restrictive #GenderNorms
❌Physical access
❌Information gaps
❌Intersecting vulnerabilities

Learn more, register for our webinar
➡️go.unu.edu/QV0nZ

📅Thu, 21 Oct
⌚11am GMT

#ResearchAgendaSetting #Fem4PeoplesVaccine

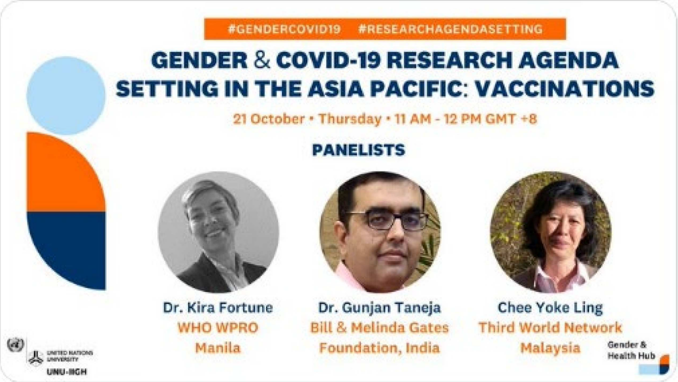

World Health Organization Philippines and 7 others

5 12

Gender & Health Hub @genderhealthhub · Oct 21, 2021

🙌 Thanks so much to all of our guests at today's webinar!

🎤 Moderator:
Dr. Sabina Faiz Rashid

📢 Guests:
Kalani Sachs-Robertson, @lavanyav149, Dr. Kira Fortune, @gtanejatweets, @YokeLing15, @gita_sen

#ResearchAgendaSetting #GenderCOVID19 #GHHTalks @WHOWPRO

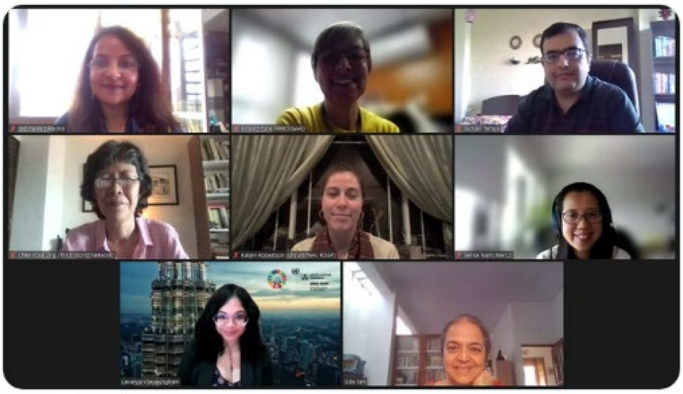

DAWN and 3 others

1 5 11

UNU International Institute for Global Health
@UNU_IIGH

🌏 900 people globally were mobilised for #COVID19 #ResearchAgendaSetting

📌 Register for @genderhealthhub's forum: go.unu.edu/wVB9f

With:
@PascaleAllotey @ashageorge72 @KarlaBerdichev1 @gita_sen @gmleunghku
Ana Maria Henao-Restrepo

#GHHForum2021 #Fem4PeoplesVaccine

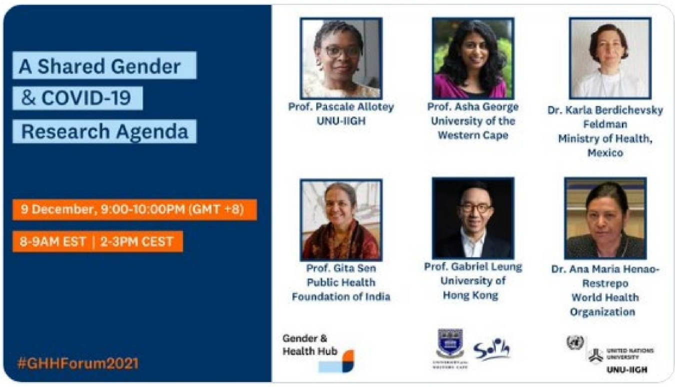

HKU – University of Hong Kong and 7 others

6:09 PM · Dec 2, 2021 · Twitter Web App

Gender & Health Hub @genderhealthhub · Dec 10, 2021

Prof @AshaGeorge72 of @SOPHUWC takes us through the year-long journey of collaboratively developing a research agenda to guide the application of a gender lens to #COVID-19 research investments, policy, & programming.

#GHHForum2021 **#ResearchAgendaSetting**

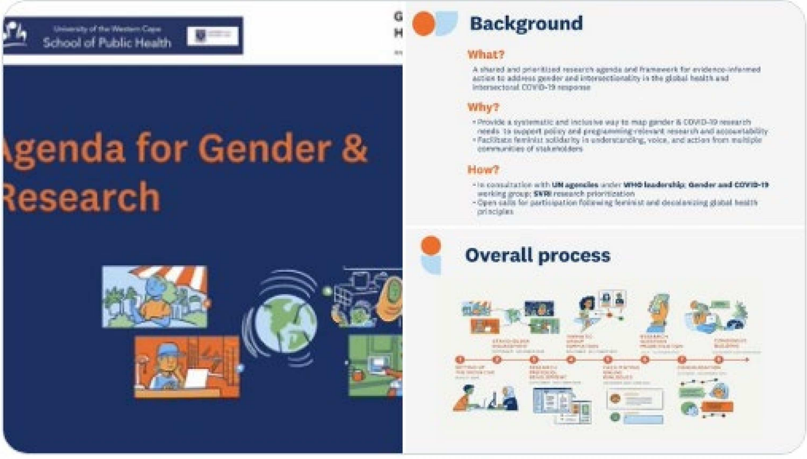

5 9

DAWN @DAWNfeminist · Dec 10, 2021

🌏 900 people globally were mobilised for the #Covid19 **#ResearchAgendaSetting**

📺 Join us at #GHHForum2021 LIVE youtu.be/HMQZygpn5XA and see the results for yourself.

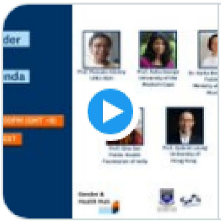

youtube.com
Setting the Agenda for Action | Launch of the Gender & COVID-19...

2

Gender & Health Hub @genderhealthhub · Dec 10, 2021

Dr Ana Maria Henao-Restrepo talks about the work of @WHO in terms of gender, making the collection & analysis of gender data possible, & future steps on R&D & innovation for COVID-19.

#GHHForum2021 **#ResearchAgendaSetting**

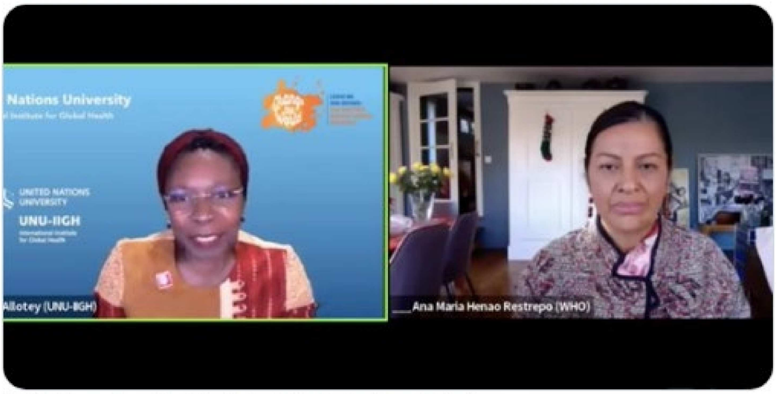

UNU International Institute for Global Health and 2 others

2 6

Gender & Health Hub @genderhealthhub · Dec 10, 2021

"In India, sex-disaggregated data is available in vaccinations but not on infections, mortality, let alone an intersectional approach. It's not that information is not available; it's that it doesn't get reported," notes @gita_sen

#GHHForum2021 **#ResearchAgendaSetting**

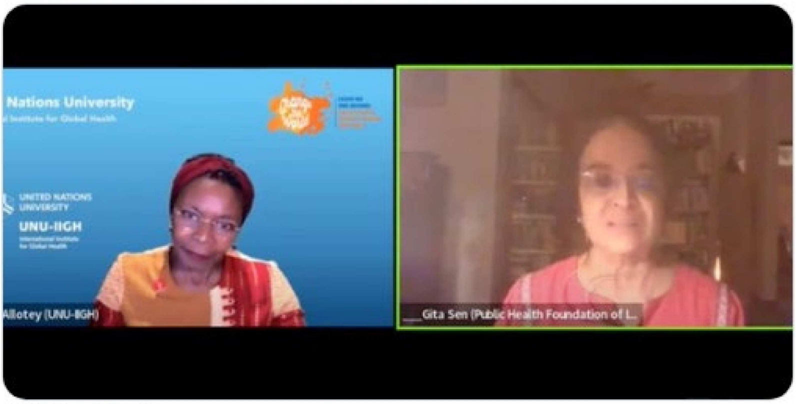

DAWN and 2 others

3 6

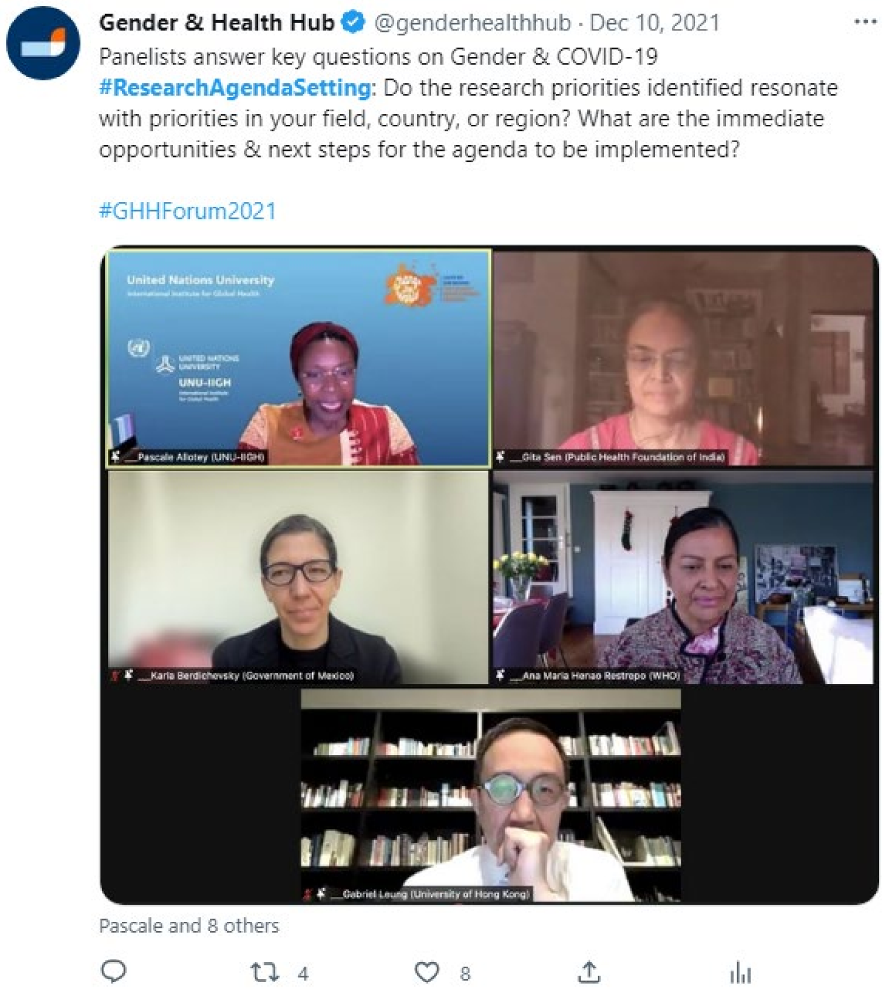
Gender & Health Hub @genderhealthhub · Dec 10, 2021
Panelists answer key questions on Gender & COVID-19
#ResearchAgendaSetting: Do the research priorities identified resonate with priorities in your field, country, or region? What are the immediate opportunities & next steps for the agenda to be implemented?
#GHHForum2021
United Nations University
Pascale Allotey (UNU-IIGH)
Gita Sen (Public Health Foundation of India)
Karla Berdichevsky (Government of Mexico)
Ana Maria Henao Restrepo (WHO)
Gabriel Leung (University of Hong Kong)
Pascale and 8 others
4
8

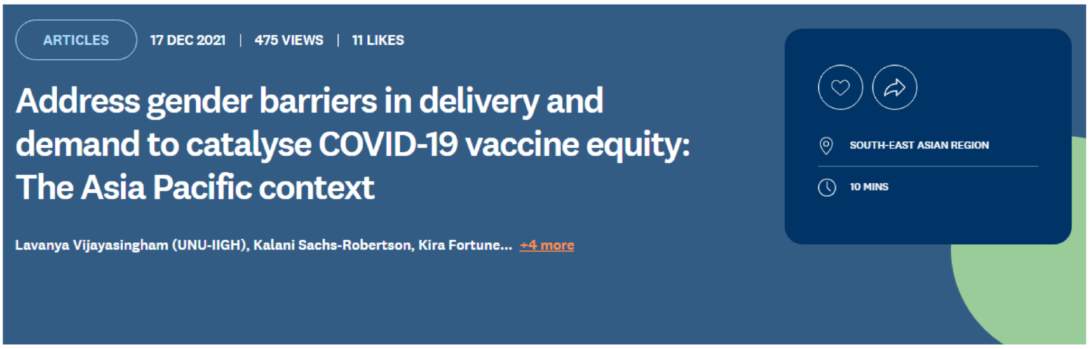
ARTICLES
17 DEC 2021 | 475 VIEWS | 11 LIKES
Address gender barriers in delivery and demand to catalyse COVID-19 vaccine equity: The Asia Pacific context
Lavanya Vijayasingham (UNU-IIGH), Kalani Sachs-Robertson, Kira Fortune... +4 more
SOUTH-EAST ASIAN REGION
10 MINS

UNU International Institute for Global Health
@UNU_IIGH

Read the new @genderhealthhub article highlighting broad principles to support the better integration of gender-responsive programme design and implementation & watch the webinar on #GenderCOVID19 #ResearchAgendaSetting in Asia Pacific: Vaccinations here 👇

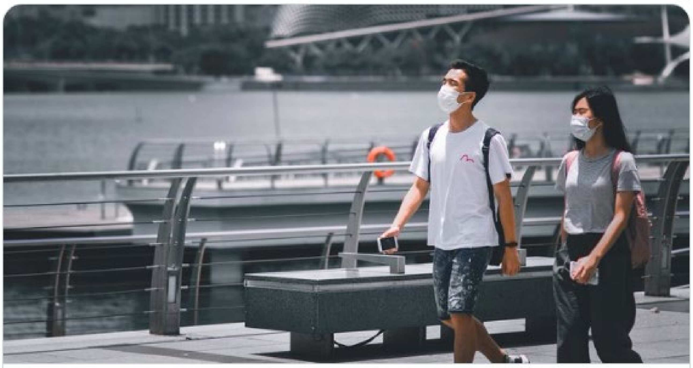

genderhealthhub.org
Address gender barriers in delivery and demand to catalyse COVID-19 vaccine equity: The Asia...

10:23 PM · Dec 20, 2021 · Twitter Web App

Gender & Health Hub @genderhealthhub · Feb 24

💬 Gender issues: critical research for future pandemic preparedness presented by @ashageorge72

📅 24 Feb 6:25pm CET
Join the livestream: go.unu.edu/o7itB

#ResearchAgendaSetting #HealthResearch #GenderAndCOVID19

Read more: go.unu.edu/FCS5j

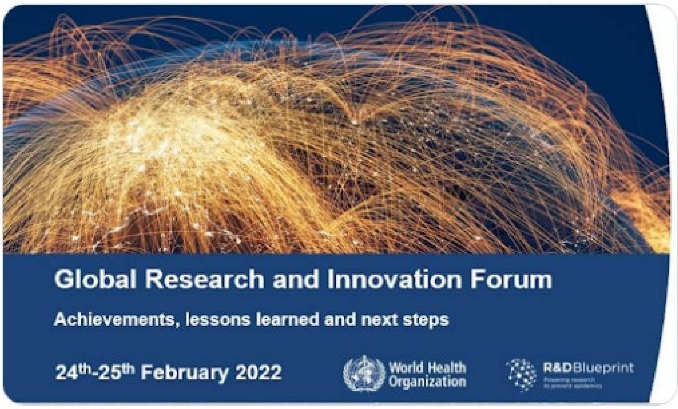

Pascale and 7 others

10 11

Gender & Health Hub @genderhealthhub · Feb 24

🚨 Don't miss @who @rd_blueprint's COVID-19 Global Research and Innovation Forum: An invitation to the research community

Put it in your diary!
📅 24 Feb 6:25pm CET
Join the livestream: go.unu.edu/S0PUb

#ResearchAgendaSetting #GlobalHealth #GenderAndCOVID19
@mamothena

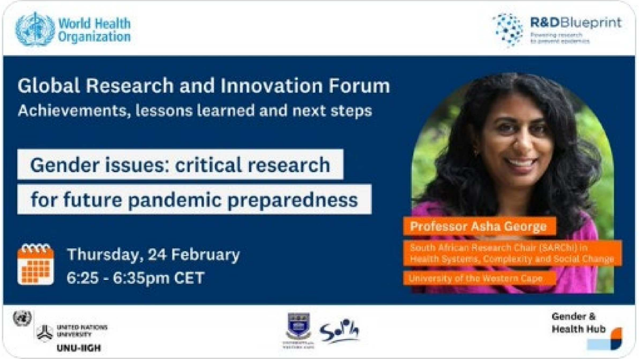

Asha George and 5 others

5 17

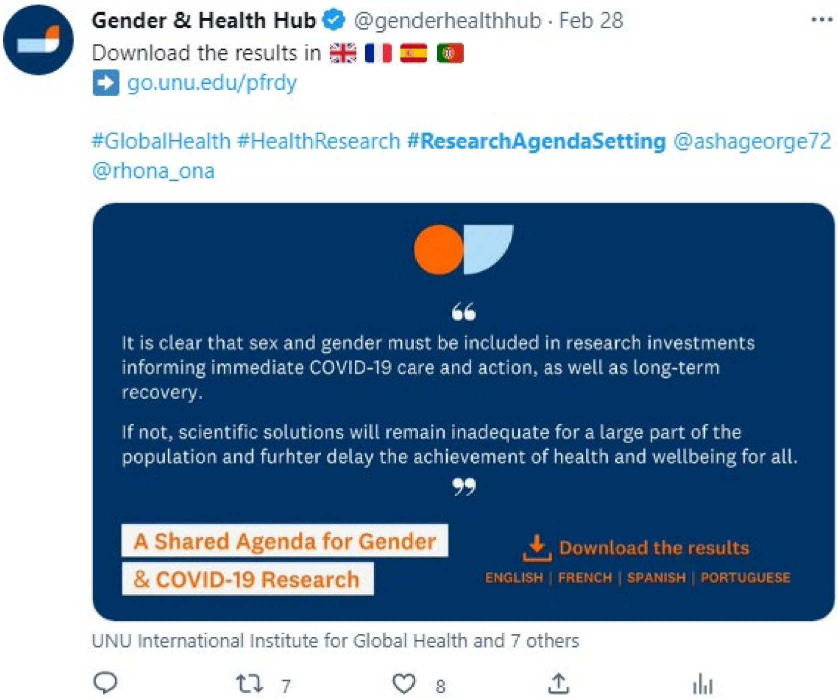
Gender & Health Hub @genderhealthhub · Feb 28
Download the results in
go.unu.edu/pfrdy
#GlobalHealth #HealthResearch #ResearchAgendaSetting @ashageorge72 @rhona_ona
It is clear that sex and gender must be included in research investments informing immediate COVID-19 care and action, as well as long-term recovery.
If not, scientific solutions will remain inadequate for a large part of the population and furhter delay the achievement of health and wellbeing for all.
A Shared Agenda for Gender & COVID-19 Research
Download the results
ENGLISH | FRENCH | SPANISH | PORTUGUESE
UNU International Institute for Global Health and 7 others
7
8
