# Supplementary material for: A shared agenda for gender and COVID-19 research: priorities based on broadening engagement in science
Source: BMJ Glob Health. 2023 May 22;8(5):e011315. doi: 10.1136/bmjgh-2022-011315 (PMC10230361; doi:10.1136/bmjgh-2022-011315)
Supplement: Supplementary data [file bmjgh-2022-011315supp003.pdf]

Gender & COVID-19 Research Agenda-Setting

Draft Thematic Reports for External Consultation

Responses invited through www.ghhbuzzboard.org or via Google drive

Thematic group 1

Health status and behavior

The gendered susceptibility to COVID-19 and the impact of the pandemic on the health behaviour and health status of people

Contents

Section 1: Introduction to the overall collaboration 2

Section 2. Thematic group participation and engagement 3

Section 3. Thematic group background 5

3.1 Definitions and scope 5

3.2 Current understanding, challenges, gaps and neglected areas 6

3.3 Desired impact of the proposed research 11

3.4 Actors and strategies to implement and promote uptake 11

3.5 Population, contexts, study design/methodologies 12

Section 4. Research questions proposed for prioritisation 12

Section 5. Resources shared 14

Section 6: Tables with Results of Prioritised Questions

Section 1: Introduction to the overall collaboration

From the start of the COVID-19 pandemic, how it affected and continues to affect: women, girls, men, boys, and non-binary gender diverse persons (non-binary persons, transgender men and women, intersex persons) are complex and evolving. Apart from the direct effects of the COVID-19 illness, pandemic responses also amplified existing gender inequalities across multiple dimensions. Context and the intersecting influence of other social determinants or identities have worsened gender inequalities during the pandemic, with cumulative effects on health.

Early high-level calls and advocacy from researchers such as through the Gender and COVID-19 working group, were made for gender considerations to be integrated in the crisis response. Nevertheless, real-time response to the gender dynamics was limited by extensive invisibility of the evolving situation, incomplete data systems and evidence gaps. As the world steps into the second year of the COVID-19 crises, given the gender dynamics involved, we must include gender in the investments being made in research informing both immediate action and long-term recovery from the health and socio-economic consequences of the pandemic.

The United Nations University International Institute for Global Health is co-convening a collaborative gender and COVID-19 research agenda-setting exercise, as part of its Gender and Health Hub's inaugural scope of work. The process is co-developed through real-time learning, and open calls to a broad range of stakeholders to comment and contribute to its design, scope and content. Collective contributions and questions for prioritization are supported by a community discussion board (www.ghhbuzzboard.org). Please visit this discussion board for further information.

The output of the exercise will be a shared research agenda that can be utilized by researchers, funders, and policy-makers to guide COVID-19 research investments and corresponding programming and policy actions by the health sector.

The draft thematic group reports emerging from this collective endeavour are a synthesized version of the contributions made to the discussion board combined with additional inputs from thematic group coordinators, co-leads and steering committee members. They document participation and engagement to date, provide a background section outlining definitions, scope, gaps, impact and audiences, before listing research questions for prioritisation.

We welcome your comments on the discussion board or through Google Drive to be posted on the discussion board to ensure we respect the inclusive and transparent ethos of the collaboration. If you comment via Google Drive please make sure we can identify your comments (do not use anonymous). Given the devastating and dynamic nature of COVID-19, we must be inclusive but also timely.

Section 2. Thematic group participation and engagement

Coordinator: Ateeb Ahmad Parray (Bangladesh)

Co-leads: Dr Lisa Thorley (United Kingdom) and Dr Yi Myint Swe (Myanmar)

Steering committee focal point: Dr Claudia Lopes (Malaysia)

Other thematic group contributors: (34)

1. ***Adaeze Ore*** (Nigeria) - Head of Department Planning Research and Statistics, National Blood Transfusion Service
2. ***Ae Mon Htun****
3. ***Amany Refaat*** (Egypt) - Professor Emeritus Public Health, Suez Canal University
4. ***Angela Wei****
5. ***Anne Gatuguta*** (United Kingdom) - Lecturer, Department of Global Health and Infection in the Brighton and Sussex Medical School, University of Sussex
6. ***Apurvakumar Pandya*** (India) - Scientist, Indian Institute of Public Health Gandhinagar
7. ***Asha George*** (South Africa) - Professor, SARCHI Chair in Health Systems, Complexity and Social Change, University of West Cape
8. ***Bernadette Ateghang-Awankem*** (Germany) - Biochemist and certified Clinical Research Associate Executive Director, African Women 4 Empowerment
9. ***Ciin Lun****
10. ***Claire Standley*** (Germany) - Assistant Research Professor within the Center for Global Health Science and Security, Georgetown University
11. ***Clara Rodriguez Ribas*** (Spain) - PhD Candidate, Department of Political and Social Sciences, Universitat Pompeu Fabra
12. ***Diana Carolina Pinzón Silva*** (Colombia) - Specialised Professional, Universidad Nacional de Colombia
13. ***Fui Ching Lam*** (Malaysia) - Data Scientist, Public Health England
14. ***Gita Mishra*** (Australia) - Professor of epidemiology, Principal Research Fellow, University of Queensland,
15. ***Galila Esam Al-samawi*** (Malaysia) - Research Intern, United Nations University International Institute for Global Health
16. ***Gideon Inyangudo*** (Nigeria) - Obafemi Awolowo University
17. ***Godwin Aja*** (Nigeria) - Professor of Public Health, Adventist International Institute of Advanced Studies
18. ***Htet Yin Win****
19. ***Jessica Oyugi*** (Uganda) - Community Health Specialist, UNICEF
20. ***Johnson Jament*** (India) - Postdoctoral Fellow, University of Sussex
21. ***Julia Critchley*** (United Kingdom) - Professor of Epidemiology, St George’s, University of London
22. ***Kathryn H. Jacobsen*** (United States of America) - Professor of Epidemiology, George Mason University
23. ***Kimberly Dickman*** (United States of America) - Assistant Professor, US Air Force
24. ***Laura Middleton*** (Canada) - Associate Professor, University of Waterloo

25. ***Lavanya Vijayasingham*** (Malaysia)- Postdoctoral fellow, UNU-IIGH
26. ***Lignet Chepuka*** (Malawi) - Lecturer, University of Malawi, Kamuzu College of Nursing
27. ***Manasee Mishra*** (India) - Public Health Researcher, Independent
28. ***Peace Musiimenta*** (Uganda) - University Lecturer/Researcher, Makerere University
29. ***Prabha Tangaraj*** (India) - Assistant Professor, Community Medicine, Trichy SRM Medical College Hospital and Research Centre
30. ***Ranjini Raghavendra*** (India) - Research Scientist and Assistant Professor, Ramalingaswami Centre on Equity and Social Determinants of Health
31. ***Sambit Dash*** (India) - Assistant Professor of Biochemistry, Melaka Manipal Medical College
32. ***Sarita Pandey****- Global Challenges Research Fellow, Department of Politics, University of Sheffield
33. ***Thu Kha Chan****
34. ***Vincent Wagne***r (Canada) - Researcher in health sciences, and clinical psychologist, Institut Universitaire Sur Les Dépendances

Timeline of the report

First draft report posted on discussion board	27-01-2021
Second draft report posted on discussion board	01-02-2021
Third draft report posted on discussion board	01-03-2021
Revised research questions shared with participants	22-03-2021

Section 3. Thematic group background

3.1 Definitions and scope

Scope and aims:

This thematic group (TG1) discusses, debates and shares experiences and resources related to the sex and gender susceptibility to COVID-19 (incidence and prevalence); gender differences in the perceptions of COVID-19 immune responses, morbidity, mortality, and post-COVID health conditions; gender differences in the uptake of public health measures; gendered uptake of the COVID-19 vaccines; the gendered impact of the pandemic on other health conditions, such as sexual and reproductive health, mental health, and other non communicable diseases (NCDs).

Definitions

Term	Definition
Health Status	an individual's relative level of wellness and illness, taking into account the presence of biological or physiological dysfunction, symptoms, and functional impairment.[1]
Health behavior	an action taken by an individual that affects health or mortality. These actions may be intentional or unintentional, and can promote or detract from the health of the actor or others.[2]
Gender	socially constructed roles, norms, and behaviors we associate with men, women, and non-binary gender diverse people. Expectations and implications of gender are shaped by historical and cultural context and intersect with other facets of identity (e.g., ethnicity or sexual orientation) to influence how people experience life.[3]
Intersectionality	promotes an understanding of human beings as shaped by the interaction of different social stratifications (e.g., ‘race’/ethnicity, Indigeneity, gender, class, sexuality, geography, age, disability/ability, migration status, religion). These interactions occur within a context of connected systems and structures of power (e.g., laws, policies, state governments and other political and economic unions, religious institutions, media). Through such processes, interdependent forms of privilege and oppression shaped by colonialism, imperialism, racism, homophobia, ableism and patriarchy are created.[4]

[1] Health Status, Health Perceptions

[2] Short, S. E., & Mollborn, S. (2015). Social determinants and health behaviors: Conceptual frames and empirical advances. *Current Opinion in Psychology*, *5*, 78–84. https://doi.org/10.1016/j.copsyc.2015.05.002

[3] How to create a gender- responsive pandemic plan

[4] Hankivsky, O., Grace, D., Hunting, G., Giesbrecht, M., Fridkin, A., Rudrum, S., Ferlatte, O., & Clark, N. (2014). An intersectionality-based policy analysis framework: Critical reflections on a methodology for

Sex susceptibility to COVID-19	vulnerability of a particular sex towards COVID-19 due to their sex.
Gender susceptibility to COVID-19	vulnerability of a particular gender towards COVID-19 due to their gender roles, social norms, social expectations.
Digital health interventions	use of technology such as mobile phones, software and applications, and big data used in the prevention, diagnostic, surveillance and management of COVID-19 cases.
COVID-19 vaccine	a substance used to stimulate the production of antibodies and provide immunity against COVID-19.
COVID-19 non-pharmaceutical interventions	behavioural interventions that include the use of face masks, enhanced hygiene, social distancing, self-isolation quarantines, disclosure of CODIS-19 status to close contacts, and self-testing.
Vaccine hesitancy	refers to delay in acceptance or refusal of vaccines despite availability of vaccine services. Vaccine hesitancy is complex and context specific, varying across time, place and vaccines. It is influenced by factors such as complacency, convenience and confidence. (WHO, 2014:7)[5]

3.2 Current understanding, challenges, gaps, and neglected areas (outline of key themes, to be finalized at the end)

3.2.1 Key themes

1. Sex and gender susceptibility to COVID-19 infections, acute morbidity and mortality	- by age, menopause status in women, strain, pre-existing condition (NCDs, HIV, immunosuppressed from other therapies), racial background and other intersectionalities, pregnancy, setting (i.e conflict area, epidemiological transition in country)<br>- risks, complications, severity and mortality in gender-diverse groups, particularly those on ongoing gender-affirming hormone therapy<br>- evolving nature and impact of strains, including on children, adolescents,

advancing equity. *International Journal for Equity in Health*, *13*(1), 119. https://doi.org/10.1186/s12939-014-0119-x

[5] WHO (2014) Report of the Sage Working Group on Vaccine Hesitancy. Available at: https://www.who.int/immunization/sage/meetings/2014/october/1_Report_WORKING_GROUP_vaccine_hesitancy_final.pdf.

	particularly (are the sex and gender-related dimensions involved in MIS-C)
2. Sex and gender differences in the risk of and experience of post-COVID sequalae and condition	- sex/gender distribution of symptoms and duration of experience in intersectional groups of men, women and gender-diverse groups, including pregnant and post-partum women, and<br>- underlying causes that lead to - hormonal, auto-immune etc, including those that are sex and gender differentiated<br>- gender differences and similarities in lived experience, including in functional limitations, quality of life and impact on work productivity<br>- economic cost of illness of post-COVID conditions<br>- rehabilitation needs, strategies, and support systems available- patient groups, peer support etc<br>- mental health and coping strategies
1. Gendered uptake of non-pharmaceutical interventions (wearing masks, hand washing, social distancing, self-isolation quarantines, disclosure to close contacts, self-testing)	- Gendered cognitive differences (beliefs, attitudes and motivation)<br>- Gendered social differences (social norms and stigma)<br>- Contextual barriers and opportunities (access to masks, water and hand sanitiser, social distance)
2. Gendered uptake of the COVID-19 vaccines	- Gender differences in the uptake<br>- Perceptions of the vaccine including myths<br>- Contextual barriers and opportunities in the access to the vaccine
3. Impact on non-COVID outcomes	- Sexual and reproductive health (SRH)<br>- Mental health<br>- Other NCDs and chronic conditions including, cardiovascular conditions, cancer, auto-immune conditions,

1. Sex and gender susceptibility to COVID-19 infections, acute morbidity and mortality

From early in the pandemic, we have been hearing about how COVID-19 affects women, girls, men, boys and non-binary gender groups in many different ways. We also now know that death and severity

differences between men and women are likely influenced by age, obesity, pre-existing conditions, disability status, and occupational exposure amongst other categories of identity and inequities.

With exceptions of a few countries, there is a sex bias in COVID-19 morbidity and mortality. A meta-analysis of 3,111,714 cases revealed that globally there is no gender difference in the proportion confirmed COVID-19 cases, but male patients have almost three times the odds of requiring intensive treatment unit (ITU) admission (OR = 2.84; 95% CI = 2.06, 3.92) and higher odds of death (OR = 1.39; 95% CI = 1.31, 1.47) compared to females[6].

However, data[7] from 139 countries that have reported sex disagregated cases for confirmed cases, have shown that the sex ratio among the confirmed cases varies with age. Assuming that cases are evenly reported by gender, women are overrepresented in both the younger (20-29 year old) and older (80 years and older) age groups, whereas for other age groups (0-9 years, 60-69 years and 70-79 years), men are overrepresented among confirmed cases[8]. As of 13th April 2021, the sex ratio (male to female) among the confirmed cases is 1.03:1. For males, the median age is 52 (IQR 37-65) years, and for females 50 (IQR 35-64) years.

Some possible biological factors underline these sex differences. For example, men have higher levels of human angiotensin-converting enzyme 2 (ACE2) that sits on cell membranes and allow the virus to enter cells more easily, meaning more cells may be vulnerable to the virus, explaining men's higher risk of severe COVID-19 outcomes and death[9].

Biological explanations are related to sex differences in the activity of the immune system and its modulation by sex hormones, with women being less susceptible to viral infections due to intense and prolonged innate, humoral, and cell-mediated immune responses[10]. Other possible biological factors that explain the lower mortality among females are sex differences in cardiovascular diseases, considering the possible role of the cytokine storm in inducing vascular inflammation and atherosclerosisrelated cardiovascular diseases and also sex differences in coagulation, which can be responsible of higher risk of thrombotic/thromboembolic phenomena in men compared to women[11].

There is a strong consensus of a positive correlation between biological age and the risk of severe COVID-19 disease. This correlation was apparent since the early days when European nations with the highest

---

[6] Peckham, H., de Gruijter, N. M., Raine, C., Radziszewska, A., Ciurtin, C., Wedderburn, L. R., Rosser, E. C., Webb, K., & Deakin, C. T. (2020). Male sex identified by global COVID-19 meta-analysis as a risk factor for death and ITU admission. *Nature Communications*, *11*(1), 6317. https://doi.org/10.1038/s41467-020-19741-6

[7] https://globalhealth5050.org/the-sex-gender-and-covid-19-project/the-data-tracker/

[8] https://globalhealth5050.org/the-sex-gender-and-covid-19-project/men-sex-gender-and-covid-19/

[9] https://globalhealth5050.org/the-sex-gender-and-covid-19-project/men-sex-gender-and-covid-19/

[10] Yale IMPACT Research Team, Takahashi, T., Ellingson, M. K., Wong, P., Israelow, B., Lucas, C., Klein, J., Silva, J., Mao, T., Oh, J. E., Tokuyama, M., Lu, P., Venkataraman, A., Park, A., Liu, F., Meir, A., Sun, J., Wang, E. Y., Casanovas-Massana, A., … Iwasaki, A. (2020). Sex differences in immune responses that underlie COVID-19 disease outcomes. *Nature*, *588*(7837), 315–320. https://doi.org/10.1038/s41586-020-2700-3

[11] Capuano, A., Rossi, F., & Paolisso, G. (2020). Covid-19 Kills More Men Than Women: An Overview of Possible Reasons. *Frontiers in Cardiovascular Medicine*, *7*, 131. https://doi.org/10.3389/fcvm.2020.00131

rate of aging populations were hit the strongest by the pandemic (e.g., Italy and Spain). This correlation is explained by aging being associated with co-morbidities and less resistance to infections.

Hormonal levels may play a role in the gendered age patterns. Given the protective effect of estrogen on COVID-19, the infection risk increases with age for women. The risk and severity of infection is higher in menopausal women compared to premenopausal women and it is reduced in women on the combined oral contraceptive pill[12]. Postmenopausal women are also at higher risk of severe COVID-19 infection and present a higher mortality rate than menopausal and premenopausal women[13].

As noted, sex differences in infection and fatality rates are gender-dependent. While there is no gender difference in young adults in terms of infection, premenopausal women have a significantly lower risk of infection and present lower disease severity than men in the same age group. This gender difference disappears as women reach the postmenopausal stage[14]. This ratio changes again in peri- and postmenopausal women, with infection rates converging with men. Although fatality rates increase with age for both genders, they become steeper for men from 50 years-old.

A study of hormone therapy in female COVID-19 patients has shown that the fatality risk for women above 50 years receiving estradiol therapy is reduced by more than 50%, because the hormone 17ß-estradiol influences expression of the human angiotensin-converting enzyme 2 (ACE2) protein, which plays a role in SARS-CoV-2 cellular entry[15].

The fact that mortality levels are higher in males compared to females, add support to the protective role of estrogens in COVID-19 infection and mortality.

Not only biological sex but also and gender roles are important drivers of risk and response to infection and disease[16]. For example, habits of smoking and drinking are higher among men as well as exposure to

---

[12] Costeira, R., Lee, K. A., Murray, B., Christiansen, C., Castillo-Fernandez, J., Lochlainn, M. N., Pujol, J. C., Macfarlane, H., Kenny, L. C., Buchan, I., Wolf, J., Rymer, J., Ourselin, S., Steves, C. J., Spector, T. D., Newson, L. R., & Bell, J. T. (2020). *Estrogen and COVID-19 symptoms: Associations in women from the COVID Symptom Study* [Preprint]. Obstetrics and Gynecology. https://doi.org/10.1101/2020.07.30.20164921

[13] Garg, R., Agrawal, P., Gautam, A., Pursnani, N., Agarwal, M., Agarwal, A., Parihar, A., & Pandey, A. (2020). COVID-19 outcomes in postmenopausal and perimenopausal females: Is estrogen hormone attributing to gender differences? *Journal of Mid-Life Health*, *11*(4), 250. https://doi.org/10.4103/jmh.jmh_287_20

[14] Wang, X.-W., Hu, H., Xu, Z.-Y., Zhang, G.-K., Yu, Q.-H., Yang, H.-L., & Zheng, J.-H. (2021). Association of menopausal status with COVID-19 outcomes: A propensity score matching analysis. *Biology of Sex Differences*, *12*(1), 16. https://doi.org/10.1186/s13293-021-00363-6

[15] Seeland, U., Coluzzi, F., Simmaco, M., Mura, C., Bourne, P. E., Heiland, M., Preissner, R., & Preissner, S. (2020). Evidence for treatment with estradiol for women with SARS-CoV-2 infection. *BMC Medicine*, *18*(1), 369. https://doi.org/10.1186/s12916-020-01851-z

[16] Wenham, C., Smith, J., & Morgan, R. (2020). COVID-19: The gendered impacts of the outbreak. *The Lancet*, *395*(10227), 846–848. https://doi.org/10.1016/S0140-6736(20)30526-2

polluted environments due to occupations and lifestyles.[17] Women and men can also respond differently to the disease, with men delaying seeking healthcare[18].

Several pre-existing conditions that have been associated with severe COVID-19 disease tend to present a higher burden among men due to biology and risk behaviours. For example, hypertension, diabetes, cardiovascular disease, and chronic lung diseases including obstructive pulmonary disease[19]. Nevertheless, some conditions such as acute myocardial infarction, Alzheimer's disease/senile dementia, atrial fibrillation, chronic kidney disease, diabetes, heart failure, and stroke have been found to to contribute high risk of severe infection, in all sexes, and age groups.This risk also is seen to be componded by race and ethnicity. For example in cancer patients, being Afican American also contributes to highest risk of severe illness, particularly in sex-specific conditions such as breast and prostrate cancer. Children with pre-existing conditions however, do not seem to be at higher risk of severe disease.

In children, while COVID-19 infections are found to be predominantly mild and manageable, with slightly higher risk of severe illness in some LMIC settings, there is a rare but serious risk of developing multi-inflammatory syndrome (MIS-C). A study from Latin America finds that hospitalisation, prevalence of and fatality from MIS-C is higher in boys, but only difference in hospitalisation was found to be statistically significant. Other studies and systematic reviews have not reported on any sex-differences in outcomes, but rather point towards the tendencies of older childeren and adolescents and certain ethnic groups (hispanic and African) tendency to develop MIS-C.

The pathways of and quality of care in the health system also depend on the patient's gender. Similarly, occupational segregation by gender also explains a differential exposure to the COVID-19. The health and social employment sector is predominantly dominated by women. ILO reported that in 104 countries analysed, women account for an estimated 67% of the health workforce[20]. Add

2. Sex and gender differences in post-COVID health conditions, and other prolonged changes

The post-infection phase of COVID, is popularly termed by patients as 'Long-COVID' and now formally termed by the WHO as ' Post-COVID Condition'. Critical aspects of this condition are the duration of symptoms, severity and type of symptoms. Some early studies have begun to suggest that more women experience Post-COVID conditions[21], including in LMIC settings. In an early study with patients with symptoms lasting between 4 and 12 weeks, long COVID was characterized by fatigue, headache, dyspnea

---

[17] Capuano, A., Rossi, F., & Paolisso, G. (2020). Covid-19 Kills More Men Than Women: An Overview of Possible Reasons. *Frontiers in Cardiovascular Medicine*, *7*, 131. https://doi.org/10.3389/fcvm.2020.00131

[18] Novak, J. R., Peak, T., Gast, J., & Arnell, M. (2019). Associations Between Masculine Norms and Health-Care Utilization in Highly Religious, Heterosexual Men. *American Journal of Men's Health*, *13*(3), 155798831985673. https://doi.org/10.1177/1557988319856739

[19] Jordan, R. E., Adab, P., & Cheng, K. K. (2020). Covid-19: Risk factors for severe disease and death. *BMJ*, m1198. https://doi.org/10.1136/bmj.m1198

[20] https://apps.who.int/iris/bitstream/handle/10665/311314/WHO-HIS-HWF-Gender-WP1-2019.1-eng.pdf

[21] Torjesen, I. (2021). Covid-19: Middle aged women face greater risk of debilitating long term symptoms. *BMJ*, n829. https://doi.org/10.1136/bmj.n829

and anosmia. A systematic review lists 50 different symptoms that are connected to Post-COVID conditions. These symptoms were more likely to be self-reported among women, and patients with increasing age, body mass index[22]. There are now emerging equivocal findings that conversely suggests little sex-differences in different sub-sets of symptoms. At the same time, only few studies have sex-dissagregated occurence of these symptoms in results.

The probable women-disproportionate prevalence is also similar to the prevalence of auto-immune conditions, and the underlying biological processes and risks such as through estrogen-and androgen linked processes.

Long-COVID began to receive mainstream expert attention through patient-led advocacy, experience sharing and conceptualisation, and was early on considered a 'contested phenomenon'. Similarities in symptoms and the 'contested nature' of the phenomenon can be drawn to chronic fatigue syndrome or myalgic encephalomyelitis, and other post-infection disorders.

Long-covid- patient led conceptualisation, and need for lived experience to inform understanding

*Functional impairment and productivity-

Fertility changes after severe covid, or in long covid- prolonged effects of infection on male and female reproduction

- some suggestion of altered semen production ad parameters, but how long this can last is not known- https://www.ncbi.nlm.nih.gov/pmc/articles/PMC7689309/
-

Auto-immune dimension and similarities- women disproportionate, estrogen linked?

https://www.nature.com/articles/s41420-021-00464-6

Long COVID in pregnant, and post-partum women

Long-COVID in pediatric population

https://www.nature.com/articles/s41420-021-00464-6

https://onlinelibrary.wiley.com/doi/full/10.1111/apa.15673

TBC

3. Gender differences in the prevention and management of COVID-19

[22] Sudre, C. H., Murray, B., Varsavsky, T., Graham, M. S., Penfold, R. S., Bowyer, R. C., Pujol, J. C., Klaser, K., Antonelli, M., Canas, L. S., Molteni, E., Modat, M., Jorge Cardoso, M., May, A., Ganesh, S., Davies, R., Nguyen, L. H., Drew, D. A., Astley, C. M., … Steves, C. J. (2021). Attributes and predictors of long COVID. *Nature Medicine*, *27*(4), 626–631. https://doi.org/10.1038/s41591-021-01292-y

Preventive behaviours are determined by a multitude of factors related to the beliefs and attitude of individuals in relation to the pandemic, the social norms that regulate the uptake of public health measures, their perceptions of effectiveness in containing the pandemic and the structural barriers and opportunities to behave in desired ways[23]. We divide these different determinants into cognitive (attitudes, beliefs and motivation), social (social norms and social stigma) and structural (barriers and opportunities)[24]. The non-pharmaceutical interventions (NPIs)[25] to prevent and manage COVID-19 can be classified into: 1) personal protective measures (face masks, respiratory etiquette, hand hygiene); 2) environmental measures (surface cleaning, increased ventilation), 3) physical distancing measures (physical distancing, self-isolation, quarantines, workplace and school measures and closures) and 4) travel-related measures (border closures, entry and exit screening, internal travel restrictions).

Cognitive determinants

The COVID-19 pandemic has been aggravated by the abundance of false information[26] regarding all aspects of the disease from its etiology and mechanism of spread, to its prevention and treatment. Hoaxes and rumours can lead to adverse health outcomes as they suppress healthy behaviours and create social stigma that can lead to hostility towards patients, health workers[27] and other groups.

Some research has shown that men and older adults (aged over 65 years) are more likely than women and younger generations to believe in misinformation and to spread fake news on social media platforms.[28] Older people present a diminished ability to correctly recall the source of original information due to losses in cognitive functions and memory accuracy[29].

[23] Mao, Y., Chen, H., Wang, Y., Chen, S., Gao, J., Dai, J., Jia, Y., Xiao, Q., Zheng, P., & Fu, H. (2021). How can the uptake of preventive behaviour during the COVID-19 outbreak be improved? An online survey of 4827 Chinese residents. *BMJ Open*, *11*(2), e042954. https://doi.org/10.1136/bmjopen-2020-042954

[24] Moorman, C., & Matulich, E. (1993). A Model of Consumers' Preventive Health Behaviors: The Role of Health Motivation and Health Ability. *Journal of Consumer Research*, *20*(2), 208. https://doi.org/10.1086/209344

[25] Non-pharmaceutical public health measures for mitigating the risk and impact of epidemic and pandemic influenza. Geneva: World Health Organization; 2019 (https://apps.who.int/iris/handle/10665/329438)

[26] Tasnim, S., Hossain, M. M., & Mazumder, H. (2020). Impact of Rumors and Misinformation on COVID-19 in Social Media. *Journal of Preventive Medicine and Public Health*, *53*(3), 171–174. https://doi.org/10.3961/jpmph.20.094

[27] McKay, D., Heisler, M., Mishori, R., Catton, H., & Kloiber, O. (2020). Attacks against health-care personnel must stop, especially as the world fights COVID-19. *The Lancet*, *395*(10239), 1743–1745. https://doi.org/10.1016/S0140-6736(20)31191-0

[28] Wylie, Lindsey & Patihis, Lawrence & Mcculler, Leslie & Davis, Deborah & Brank, Eve & Loftus, Elizabeth & Bornstein, Brian. (2014). 2 Misinformation Effect in Older Versus Younger Adults A Meta-Analysis and Review.
[29] Jacoby, L. L., & Rhodes, M. G. (2006). False Remembering in the Aged. *Current Directions in Psychological Science*, *15*(2), 49–53. https://doi.org/10.1111/j.0963-7214.2006.00405.x

Misinformation is associated with rejection of public health measures, but the opposite is not necessarily true. Evidence from previous outbreaks (Zika and Yellow fever) suggests that counter misconceptions with corrective information also reduces the accuracy of correct beliefs about the disease[30]. Interventions to correct misinformation do not per se lead to support for preventive measures and intentions to engage in preventive behaviours.

Gender differences also account for behavioral changes in response to a new health risk. Evidence from a cross-national representative survey with 21,649 respondents conducted in eight high income countries[31] (Australia, Austria, France, Germany, Italy, New Zealand, the United Kingdom, and the United States) revealed that women are more likely to see COVID-19 as a serious problem, to agree with restraining public policy measures and to comply with them. These results may reflect a general trend for women to be more risk averse and to support more left-wing political views. But even after controlling for factors such as level of trust in science and political ideology, gender differences persist across all countries. These results are not explained by sociodemographic or employment characteristics, offering support to the need of a gender based approach to public health policies and health communication during pandemics.

Social determinants

A study conducted in the United States of America demonstrated that while mask usage has increased rapidly during the initial outbreak, men have substantially reported less usage of face masks.[32] It has been well established in the literature[33] that men's norms of masculinity prevent them from seeking health-care and from following health guidelines[34]. Lower mask usage among men with a strong sense of masculinity may signal that they are unconcerned about getting sick, reinforcing their gender identity.

[30] Carey, J. M., Chi, V., Flynn, D. J., Nyhan, B., & Zeitzoff, T. (2020). The effects of corrective information about disease epidemics and outbreaks: Evidence from Zika and yellow fever in Brazil. *Science Advances*, *6*(5), eaaw7449. https://doi.org/10.1126/sciadv.aaw7449

[31] Galasso, V., Pons, V., Profeta, P., Becher, M., Brouard, S., & Foucault, M. (2020). Gender differences in COVID-19 attitudes and behavior: Panel evidence from eight countries. *Proceedings of the National Academy of Sciences*, *117*(44), 27285–27291. https://doi.org/10.1073/pnas.2012520117

[32] Of Masks and Men? Gender, Sex, and Protective Measures during COVID-19. Cambridge University Press.

[33] Courtenay, W. H. (2000). Constructions of masculinity and their influence on men's well-being: A theory of gender and health. *Social Science & Medicine*, *50*(10), 1385–1401. https://doi.org/10.1016/S0277-9536(99)00390-1

[34] Mahalik, J. R., Burns, S. M., & Syzdek, M. (2007). Masculinity and perceived normative health behaviors as predictors of men's health behaviors. *Social Science & Medicine*, *64*(11), 2201–2209. https://doi.org/10.1016/j.socscimed.2007.02.035

These effects are independent of partisanship, being observed both in Republican and Democratic supporters.

Contextual barriers and enablers

In low and middle income countries (LMICs), less than 30% of people have adequate hand washing facilities at home.[35] The pandemic exacerbates gender inequalities related to WASH globally. Period poverty (lack of access to sanitary products), menstrual hygiene education, toilets, hand washing facilities, and waste management are affecting people globally, and women living in poverty have become more vulnerable.[36] Safely managed WASH services are critical during the recovery phase of a disease outbreak to mitigate secondary impacts on community livelihoods and wellbeing.[37]

The secondary impacts of the pandemic include disruptions in supply chains, inability to pay bills, or panic-buying. They all have negative consequences for the continuity and quality of water and sanitation services, the ability of affected households to access and pay for WASH services and products (for instance, soap, point of use water treatment or menstrual hygiene products) and the ability of schools, workplaces and other public spaces to maintain effective hygiene protocols when they re-open. If not managed, secondary impacts can increase the risk of further spreading water borne diseases, including potential disease outbreaks such as cholera, particularly where the disease will become endemic.

4. Gendered COVID-19 vaccine hesitancy

With the role out of the vaccine in many countries, it has been widely reported that there is a degree of vaccine hesitancy,[38] with vaccine hesitancy being attributed to a lack of knowledge as well as scepticism to its effectiveness and in the case of the Astrazeneca vaccine, safetly.

This noted, in the context of the UK vaccine hesitancy is highest amongst ethinc minority groups with 4 in 10 adults of Black or Black British heritage being hesitant to use the vaccine, this is compared to 1 in 10 White British adults.

[35] https://www.usglc.org/coronavirus/water-sanitation-hygiene-wash-in-slowing-the-spread/

[36] https://www.greaterkashmir.com/news/opnion/period-poverty-breaking-the-shame/

[37] https://www.worldbank.org/en/topic/water/brief/wash-water-sanitation-hygiene-and-covid-19

[38] Wiysonge, C. et al (2021)Vaccine hesitancy in the era of COVID-19: could lessons from the past help in divining the future? *Human Vaccines and Immunotherapeutics.*

Other than race there is a notable difference in how young women wish to engage with the vaccine, with 19% of women aged between 16 and 29 being hesitant.[39] Therefore there is a need for Governments to engage with their citizens so that they have access to the right information and that this information is tailored to the needs of individuals.[40]

Women may be experiencing vaccine side effects more than men, but men may also be reporting them less frequently. As we lack robust evidence for this claim, we do know that it is possible based on what we know about how men behave in relation to health care.

5. Impact on non-COVID health outcomes

The burden that the epidemic has put on health systems globally has had an adverse effect on individuals with negative consequences for women. Experiences from past epidemics, for example Ebola, reveal that women and girls could also be denied access to basic health services. During COVID-19, essential health services were affected across the board. The most frequently disrupted services included routine immunization services – outreach services (70%) and facility-based services (61%) – noncommunicable disease diagnosis and treatment (69%), family planning and contraception (68%), treatment for mental health disorders (61%), antenatal care (56%) and cancer diagnosis and treatment (55%)[41]. In the context of equity of access to health care, women and girls often face delays in getting the services they need due to gender and social norms that are pre-existing in society.

Impact on SRH

There have been numerous advancements in the field of sexual and reproductive health, more women and girls than ever have been, and are able to access family planning, especially women and girls in Sub Saharan Africa.[42] Unlike the Zika virus outbreak, where sexual and mother-to-infant transmission were well-established, much less is known today about these potential transmission routes for COVID-19. In addition, the specific risk to pregnant women and their infants is not yet clear, but these groups are often

[39] ONS (2021)Coronavirus and vaccine hesitancy, Great Britain: 13 January to 7 February 2021. Available at: https://www.ons.gov.uk/peoplepopulationandcommunity/healthandsocialcare/healthandwellbeing/bulletins/coronavirusandvaccinehesitancygreatbritain/13januaryto7february2021 (Accessed, 30th April 2021)

[40] Wiysonge, C. et al (2021)Vaccine hesitancy in the era of COVID-19: could lessons from the past help in divining the future? Human Vaccines and Immunotherapeutics.

[41] https://www.who.int/publications/i/item/WHO-2019-nCoV-essential-health-services-2020

[42]a Kantorová, V. et al (2020) World Family Planning 2020 Highlights. Available at: https://www.un.org/development/desa/pd/sites/www.un.org.development.desa.pd/files/files/documents/2020/Sep/unpd_2020_worldfamilyplanning_highlights.pdf (Accessed 30th April 2021).

particularly vulnerable to infectious disease threats[43]. Also, many of the health care professionals are being put at risk in the midst of the outbreak, while simultaneously contracting it. It may also intensify the difficulty in acquiring physicians in providing sexual and reproductive health care and lengthen their wait times. This will put a severe burden on facilities that service a small number of patients, particularly for non-emergency treatment. This noted, due to the actions of the previous US adminstration, access to family planning, most notably abortion services has been serverily disrupted.[44] This has been exacerbated due to COVID-19.[45]

Other than access to safe abortions being disrupted, access to family planning and contraception services has been also impacted[46]. The reasons for this are numerous, however global lockdowns have disrupted services and there have also been issues with supplies. At the health system level, shortages of medications—such as contraceptives, antiretrovirals for HIV/AIDS and antibiotics to treat STIs—are prevalent due to disruptions in supply chains overall. In April 2020 the UNFPA predicted that in the worst case scenario as many as 47 million women would lose access to contraceptive commodities.[47] Due to family planning often being viewed as a 'womans' issue, the burden of access and lack of access to family planning and sexual and reproductive health services has fallen to women. This has left women in a precarious position with women's choices often being restricted. Not only have family planning services been disrupted, women, as well as men have also seen access to HIV services disrupted with people being unable to access ARVs, testing or psychosocial support. [48] This has especially been the case in the context of countries in Sub Saharan Africa. [49] Similarly, the global gag rule that has now been reversed, hampers

[43] https://www.thelancet.com/journals/lancet/article/PIIS0140-6736(20)30365-2/fulltext
[44] Ushie, B. et al. (2020) Foreign assistance or attack?
Impact of the expanded Global Gag Rule on sexual and reproductive health and rights in Kenya, *Sexual and Reproductive Health Matters.* 28:(3) 23-38.
[45] Endler, M. et al (2020) How the coronavirus disease 2019 pandemic is impacting sexual and reproductive health and rights and response: Results from a global survey of providers, researchers, and policy-makers. *Acta Obstet Gynecol Scand.* 100:571–578.

[46] Burki, T. (2020). The indirect impact of COVID-19 on women. The Lancet Infectious Diseases, 20(8), 904–905. https://doi.org/10.1016/S1473-3099(20)30568-5
[47] UNFPA (2020) "Impact of the COVID-19 Pandemic on Family Planning and Ending Gender-based Violence, Female Genital Mutilation and Child Marriage," Interim Technical Note. Available at: https://www.unfpa.org/resources/impact-covid-19-pandemic-family-planning-and-ending-gender-based-violence-female-genital. (Accessed 30th April 2021).
[48] Waterfield, K. et al.(2021) Consequences of COVID-19 crisis for persons with HIV: the impact of social determinants of health. BMC Public Health. 299.

[49] Dorward et al (2021) The impact of the COVID-19 lockdown on HIV care in65 South African primary care clinics: an interrupted time series analysis. The Lancet.

the ability of foreign nongovernmental organizations to partner with the U.S. government to fill in gaps in sexual and reproductive health services created by the COVID-19 response[50].

These impacts may be compounded by the diversion of financial resources to COVID-19 response, which would take funding away from reproductive health programs and decrease access for patients who rely on free or subsidized care. In the past as well, in many Ebola-affected countries, the national responses did not prioritize sexual and reproductive health and respective programs did not sufficiently adapt to the outbreak, which created delays in the care that pregnant people received and increased maternal mortality and morbidity[51]. This burden may not be distributed equally across the health care system, as certain facilities or geographic areas may be most impacted by the outbreak and need to take more focused action. Adding to this, along with waiting periods and resource shortages, many individuals accessing sexual and reproductive health services are facing additional economic and social obstacles[52]. To begin, citizens have legitimate concerns about taking public transportation during an outbreak, and in severe cases, city governments have cancelled transit services. This may be a significant barrier to accessing reproductive health. Additionally, many people already faced barriers to seeking health care if they could not afford child care. The COVID-19 pandemic has worsened this situation. School and daycare closures have eliminated child care options, as the child care workers might become sick themselves, and many parents may not feel comfortable bringing a child to a health care appointment and risk exposure to the virus.

Impact on mental health

The COVID-19 pandemic has placed a high burden on women as they are more affected by the challenges of childcare and homeschooling, the greater risk of domestic violence, the anxiety of layoffs and furloughs, and the related financial insecurity. Gender inequalities that were already evident have been exacerbated with numerous academics predicting that gains that have been made in the equality discourse risk being lost.[53]

Adding to the anxiety stemming from life changes, financial insecurity and health risks, isolation may also have a negative impact on the mental health of individuals. The social norm of the male 'breadwinner'

[50] https://www.guttmacher.org/gpr/2017/06/when-antiabortion-ideology-turns-foreign-policy-how-global-gag-rule-erodes-health-ethics

[51] https://www.guttmacher.org/article/2020/03/covid-19-outbreak-potential-fallout-sexual-and-reproductive-health-and-rights

[52] https://www.unfpa.org/resources/sexual-and-reproductive-health-and-rights-maternal-and-newborn-health-covid-19-0

[53] Summers, H. (2020) UK society regressing back to 1950s for many women, warn experts
This article is more than 10 months old Inequality has worsened during lockdown, says study, with women saddled with more not less childcare. Available at: https://www.theguardian.com/inequality/2020/jun/18/uk-society-regressing-back-to-1950s-for-many-women-warn-experts-worsening-inequality-lockdown-childcare (Accessed April 30th 2021).

would also put pressure on men during times of job insecurity and economic uncertainty. Lockdowns led to increased rates of domestic violence, which disproportionately are perpetrated by men against women.

People living with chronic diseases and elderly people have been deprived from social contact, with women being over-represented in these groups. Technologies that help people to be connected are not yet equally distributed as more people living in low income countries, women, and elderly people are still being digitally excluded.

A focused survey administered to the Region of the Americas[54] (PAHO, 2020) revealed that mental health prevention and promotion were the core mental health services most severely affected during the pandemic, particularly those aimed at children and adolescents (due to school closures) and pregnant women and new mothers (due to the cessation of community services). In more than half the countries surveyed, psychotherapy and counselling were disrupted. With levels of violence against women and girls rising worldwide during the pandemic (UN Women, 2020), the suspension of psychological support services poses an additional risk for the mental health of women.

- add evidence on gender differences in suicide rates and other mental health issues
- post-COVID stress disorder:

Impact on other NCDs

The nature of COVID-19 may make some NCDs more difficult to recognize. For example, COVID-19 has been associated with cardiovascular complications that can make the accurate diagnosis of myocardial infarction increasingly complex. For patients with existing chronic respiratory disorders such as chronic obstructive pulmonary disease and asthma. It may be difficult to recognize when immediate medical attention can be pursued as such diseases have very common symptoms to COVID-19.

Most conversations till date are about service disruption and resource diversion during lockdown, and pandemic. These disruptions, along with intentional delayed health seeking is likely to have impact on NCD and chronic conditions risks, management, and progression or outcomes. For example higher all-cause mortality has been reported in patients with cardiovascular conditions during and after lock down period had with lower rates of in-hospital, but higher rates of out of hospital deaths compared to same time period in the previous year- Denmark nationwide registries.

More research is required on the impact of COVID-19 infections and the broader dynamics of the pandemic on NCD and other chronic condition outcomes. For example, The pandemic restrictions negatively influence risk factors for NCDs such as (in)ability to exercise, stress and increase in care work in women which are known gendered risk factors in CVD.

3.3 Desired impact of the proposed research on policy, programme, and community responses

[54] The Region of Americas contains six of the ten countries with the highest number of COVID-19 cases and deaths reported globally by October 2020 (WHO, 2020).

Sex and gender susceptibility to COVID-19 infections, acute morbidity and mortality

1. Collect sex, gender and age disagregated data to monitor and analyse susceptibility, morbidity and mortality specifically on women and transgender populations.
2. Collect data across key life stages eg around pregnancy (pre-, during and a post pregnancy), menopause, widowed.
3. Collect longitudinal data so that we can monitor more effectively, determine pathways that worked etc.

Sex and gender differences in post-COVID health conditions

1. Identify pathways that contribute to vulnerability and resilience to the consequences of COVID-19 by sex and gender

Gender differences in the uptake of non-pharmaceutical interventions

2. Develop data algorithms that assist in educating the public about pandemics on social media.
3. Integrate sex and gender differences in platforms design and artificial intelligence models for information targeting.
4. Investing in core public health infrastructure, including water and sanitation systems.

Gendered uptake of the COVID-19 vaccines

Impact on non-COVID health outcomes

1. Understand mental health and other NCDs particularly for women and men, adolescent girls and LGBTQ+ to inform policy and programmes.
2. Understand how restricted access to healthcare services during COVID-19 impact on key health indicators of pregnant women and adolescent girls to inform policy and programmes.
3. As there is gender difference in the prevalence and aetiology of NCDs, the bidirectional relationship with COVID-19 is unknown. Longitudinal data are needed to uncover this relationship.

3.4 Actors and strategies to implement and promote uptake of the research agenda

Core actors with whom to engage

1. Government actors
2. International and national agencies
3. Community health workers
4. Women healthcare workers at frontline and in leadership

Strategies

1. Calls for action
2. Policy briefs
3. Infographics

4. Factsheets
5. Consultative engagements with key actors

3.5 Population, contexts, study design/methodologies
(to be written at the end once research questions are prioritized)

Populations

1. LGBTQ+
2. Women working in formal and informal sectors (particularly from LMIC)
3. Rural and indigenous communities adolescent girls and women
4. LGBTQ+, adolescents girls and women from marginalised ethnic groups
5. Women's groups (formal groups such as self-help groups, village-level committees; informal groups such as mothers' groups etc.)
6. Women health care workers - frontline health workers and community health workers
7. School teachers (elementary/primary and high secondary)
8. Women migrant workers, refugees and floating populations
9. Women and adolescents living in informal settlements
10. Women and adolescents in women's homes and shelters
11. Men in terms of their different risk of death from COVID-19 and risk behaviours

Methodologies

1. Case studies
2. Key informant interviews and other qualitative methods
3. Scoping and systematic reviews, meta-analyses
4. Participatory action research
5. Policy analysis
6. Longitudinal and cross-sectional surveys, cohort/observational studies, including using outcomes registries/database
7. Retrospective and prospective longitudinal surveys
8. Prospective case-control studies
9. Economic costing and cost-of illness studies
10. Social media analysis

Section 4. Research questions proposed for prioritization

<u>Total number of questions: max 41</u>

Sex and gender susceptibility to COVID-19 infections, acute morbidity, and mortality

1. What are the biological and social determinants underpinning sex and gender differences in COVID-19 infections, acute morbidity, and mortality?

2. How do the sex and gender determinants of COVID-19 infections, acute morbidity and mortality intersect with other social categories (such as race, disability, migrant status, age, sexuality, etc) across various contexts?
3. Are there sex and gender differences in the infection, acute morbidity and mortality outcomes of differents strains, particularly due to variants of concern?
4. How do the infection, acute morbidity and mortality outcomes of gender-diverse groups differ to those of cis-gender male and females?
5. What are the infection, acute morbidity and mortality levels of COVID-19 among pregnant and post-partum women, and their foetus/infants across various contexts?
6. What are the gender differences in economic and financial costs of illness with COVID-19 infections, acute morbidity and mortality?
7. What is the effect of pre-existing conditions and therapies that relate to changes in sex-hormones on COVID-19 infections, acute morbidity and mortality?
8. What is the duration and level of immune response, and antibody levels in COVID-19 patients by age, sex, severity and comorbidities (e.g., HIV, TB, NCDs, PCOS, immunocompromised individuals)?
9. What are the sex differences in the prevalence of multisystem inflammatory syndrome (MIS-C), other forms of acute COVID-19 morbidities in boys, girls and adolescents? If yes, what factors contribute to any differences?
10. Does sex and age influence the viral transmission rate in asymptomatic and symptomatic vaccinated individuals?

Sex and gender differences in post-COVID conditions

11. What are the biological and social determinants underpinning sex and gender differences for post-COVID conditions?
12. How do the sex and gender determinants of post-COVID conditions intersect with other social categories (such as race, disability, migrant status, age, sexuality, etc) across various contexts?
13. Are there sex and gender differences in post-COVID outcomes of differents strains, particularly due to variants of concern?
14. How does post-COVID conditions affect pregnant and postpartum women, and their foetus/infants across various contexts?
15. How does post-COVID affect sex specific sexual and reproductive issues such as fertility, erectile dysfuntion, etc?
16. How does post-COVID affect neurological and psychiatric outcomes for men, women and gender diverse individuals?

17. What are the sex and gender differences in post-COVID related impairment, functional ability, rehabilitation needs and the temporal nature of these experiences?
18. What are the gender differences in economic and financial costs of illness due to post-COVID?
19. What are the gender differences in post-COVID illness experience and the coping needs of different groups of women, men and gender-diverse individuals?
20. How does long-COVID manifest in boys, girls, and adolescents? What are the resultant sex differences?

Gender differences in the acceptance and uptake of non-pharmaceutical interventions

21. What are the gender differences in the acceptance and uptake of non-pharmaceutical interventions of COVID-19 and what social determinants underpin them?
22. How do the gender differences in the acceptance and uptake of non-pharmaceutical interventions of COVID-91 vary across social categories (such as race, disability, migrant status, age, sexuality)?
23. What approaches are most effective at successfully integrating gender aspects in social and behavioural change communication to enhance uptake of non-pharmaceutical interventions?
24. What best practices can be used to ensure compliance with public health measures (e.g., physical distance, wearing masks, etc) by different groups of women, men and gender diverse individuals?
25. What are the most effective strategies for debunking myths and countering misinformation about COVID-19 for different groups of women, men and gender diverse individuals ?

Gender differences in the acceptance and uptake of COVID-19 vaccines

26. Are there gender differences in the acceptance and uptake of COVID-19 vaccines? If so, what social determinants underpin them?
27. Do the gender differences in the trust, acceptance and uptake of COVID-19 vaccines vary across social categories (such as race, disability, migrant status, age, sexuality and pre-existing conditions)?
28. What approaches are most effective at successfully integrating gender aspects in risk communication and community engagement to increase the uptake of COVID-19 vaccines?
29. How is sex and gender related vaccine data and emerging evidence communicated to national policy makers and to the public?

30. What channels and platforms for risk communication and community engagement are the most effective to increase the acceptance of COVID-19 vaccines among women, men and gender diverse individuals?

31. What types of incentives for COVID-19 vaccination are the most effective for different groups of women, men and gender diverse individuals and in what contexts?

Impact of pandemic responses on other health conditions

Sexual and reproductive health (SRH)

32. How did contraceptive use change due to the disruption of sexual and reproductive health services during the COVID-19 pandemic?

33. What is the impact of the COVID-19 pandemic, including school closures, on the levels of early pregnancy, miscarriage and low-birth weight babies, unplanned pregnancies, safe and unsafe abortions, and their outcomes?

34. How have people who are living with HIV or TB been affected by treatment and testing disruptions due to COVID-19? Have women and girls and key populations been disproportionately affected?

35. How have women and girls in low resource settings managed their menstrual needs during the COVID-19 lockdown?

Mental Health and other non communicable diseases (NCDs)

36. What was the impact of COVID-19 measures on the mental health outcomes of women, men, women, girls, boys, LGBTQI+ and gender-diverse persons?

37. Were there gender differences in the impact of the closure of educational institutions on the mental health of students?

38. Has vaccination impacted (positively or negatively) the mental health outcomes of men and women at the population level?

39. How has COVID-19 measures affected the mental health of working women when help for childcare is not available, but they are still expected to work and manage their households?

40. What is the gendered impact of COVID-19 stress i.e. unemployment, unpaid care work on different chronic disease experiences and outcomes for cancer, cardiovascular disease, cardiometabolic disease, neurological and auto-immune conditions?

Section 5. Resources shared to date

Notes from meeting - TG-1

- Health Status: Incidence and Prevalence are two epidemiological terms, Same for morbidity and mortality. Describe the first section again! Done
    - Other ways of describing health status:
        - How are we making sure that these questions have not already been worked on?
        - Need to tabulate them to see the extent from repetition and approach the questions from the angle of the TG
        - **Three main indicators:** Cases, deaths and vaccination
        - Sex and age based susceptibility
        - Separate discussion on gender based susceptibility
        - Add hospitalizations - marker of severity
        - Sex/Age in co-morbidities
        - Ensure quarantine, isolation and social distancing are not left out
        - Include HIV as a burden and its impacts in both directions. Others include diabetes, Hypertension
        - Do we include pregnancy and how do we lay out the boundaries
    - Resources: WHO guidelines on resources and priorities
    - Some topics are missing the research questions (SRH)
        - Reach out to other experts
        - Ateeb, Lisa and Claudia to fill in

Section 6: Results from First Round of Prioritisation within Each Thematic Group

Table 6: High Priority Gender and COVID-19 Research Questions for all criteria

RQ	Label	Public Health	Gender equality	Urgency
RQ26	Are there gender differences in the acceptance and uptake of COVID-19 vaccines	X	X	X
RQ27	Do the gender differences in the trust, acceptance and uptake of COVID-19 vaccines vary across social categories (such as race, disability, migrant status, age, sexuality and pre-existing conditions)	X	X	X
RQ5	What are the infection, acute morbidity and mortality levels of COVID-19 among pregnant and postpartum women, and their foetus/infants across various contexts	X	X	X
RQ8	What is the duration and level of immune response, and antibody levels in COVID-19 patients by age, sex, severity and comorbidities (e.g., HIV, TB, NCDs, PCOS, immunocompromised individuals)	X	X	

RQ14	How does post-COVID conditions affect pregnant and postpartum women, and their foetus/infants across various contexts	**X**	**X**	
RQ36	What was the impact of COVID-19 measures on the mental health outcomes of women, men, women, girls, boys, LGBTQI+ and gender-diverse persons	**X**	**X**	
RQ40	What is the gendered impact of COVID-19 stress i.e. unemployment, unpaid care work on different chronic disease experiences and outcomes for cancer, cardiovascular disease, cardiometabolic disease, neurological and auto-immune conditions	**X**	**X**	
RQ2	How do the sex and gender determinants of COVID-19 infections, acute morbidity and mortality intersect with other social categories (such as race, disability, migrant status, age, sexuality, etc) across various contexts	**X**	**X**	
RQ33	What is the impact of the COVID-19 pandemic, including school closures, on the levels of early pregnancy, miscarriage and low-birth weight babies, unplanned pregnancies, safe and unsafe abortions, and their outcomes	**X**	**X**	
RQ25	What are the most effective strategies for debunking myths and countering misinformation about COVID-19 for different groups of women, men and gender diverse individuals	**X**		**X**
RQ35	What was the impact of COVID-19 lockdown on how women and girls in low resource settings managed their menstrual needs?		**X**	
RQ3	Are there sex and gender differences in the infection, acute morbidity and mortality outcomes of differents strains, particularly due to variants of concern			**X**
RQ1	What are the biological and social determinants underpinning sex and gender differences in COVID-19 infections, acute morbidity, and mortality			**X**
RQ6	What are the gender differences in economic and financial costs of illness with COVID-19 infections, acute morbidity and mortality		**X**	
RQ39	How has COVID-19 measures affected the mental health of working women when help for childcare is not available, but they are still expected to work and manage their households		**X**	
RQ29	What approaches are most effective at successfully integrating gender aspects in risk communication and community engagement to increase the uptake of COVID-19 vaccines		**X**	

Supplementary Table A: Sample size, Means, Standard deviations and 95% Confidence Intervals for Gender and COVID-19 Research Questions by Public Health Benefit

RQ	Label	N	Mean	SD	95% CI
RQ27	Do the gender differences in the trust, acceptance and uptake of COVID-19 vaccines vary across social categories (such as race, disability, migrant status, age, sexuality, and pre-existing conditions)	34	3.76	0.43	3.74-3.79
RQ8	What is the duration and level of immune response, and antibody levels in COVID-19 patients by age, sex, severity and comorbidities (e.g., HIV, TB, NCDs, PCOS, immunocompromised individuals)	34	3.71	0.58	3.67-3.74
RQ26	Are there gender differences in the acceptance and uptake of COVID-19 vaccines	33	3.70	0.53	3.67-3.73
RQ14	How does post-COVID conditions affect pregnant and postpartum women, and their foetus/infants across various contexts	35	3.69	0.63	3.65-3.72
RQ36	What was the impact of COVID-19 measures on the mental health outcomes of women, men, women, girls, boys, LGBTQI+ and gender-diverse persons	35	3.66	0.59	3.62 -3.69
RQ5	What are the infection, acute morbidity and mortality levels of COVID-19 among pregnant and post-partum women, and their foetus/infants across various contexts	35	3.66	0.59	3.62-3.69
RQ40	What is the gendered impact of COVID-19 stress i.e. unemployment, unpaid care work on different chronic disease experiences and outcomes for cancer, cardiovascular disease, cardiometabolic disease, neurological and auto-immune conditions	36	3.64	0.54	3.61-3.67
RQ25	What are the most effective strategies for debunking myths and countering misinformation about COVID-19 for different groups of women, men and gender diverse individuals	35	3.60	0.65	3.56-3.64
RQ2	How do the sex and gender determinants of COVID-19 infections, acute morbidity and mortality intersect with other social categories (such as race, disability, migrant status, age, sexuality, etc) across various contexts	39	3.59	0.59	3.56-3.62
RQ29	What approaches are most effective at successfully integrating gender aspects in risk communication and community engagement to increase the uptake of COVID-19 vaccines	35	3.57	0.56	3.54-3.60
RQ33	What is the impact of the COVID-19 pandemic, including school closures, on the levels of early pregnancy, miscarriage and low-birth weight babies, unplanned pregnancies, safe and unsafe abortions, and their outcomes	35	3.57	0.56	3.54-3.60

Gender & Health Hub

RQ23	What approaches are most effective at successfully integrating gender aspects in social and behavioural change communication to enhance uptake of non-pharmaceutical interventions	36	3.56	0.56	3.53-3.59
RQ3	Are there sex and gender differences in the infection, acute morbidity and mortality outcomes of differents strains, particularly due to variants of concern	33	3.55	0.67	3.51-3.58
RQ39	How has COVID-19 measures affected the mental health of working women when help for childcare is not available, but they are still expected to work and manage their households	37	3.54	0.65	3.51-3.57
RQ22	How do the gender differences in the acceptance and uptake of non-pharmaceutical interventions of COVID-91 vary across social categories (such as race, disability, migrant status, age, sexuality)	34	3.53	0.56	3.50-3.56
RQ24	What best practices can be used to ensure uptake of non-pharmaceutical interventions (e.g., physical distance, wearing masks) by different groups of women, men and gender diverse individuals	34	3.53	0.56	3.50-3.56
RQ12	How do the sex and gender determinants of post-COVID conditions intersect with other social categories (such as race, disability, migrant status, age, sexuality, etc) across various contexts	35	3.51	0.61	3.48-3.55
RQ11	What are the biological and social determinants underpinning sex and gender differences for post-COVID conditions	34	3.50	0.56	3.47-3.53
RQ30	What channels and platforms for risk communication and community engagement are the most effective to increase the acceptance of COVID-19 vaccines among women, men and gender diverse individuals	36	3.50	0.61	3.47-3.5
RQ1	What are the biological and social determinants underpinning sex and gender differences in COVID-19 infections, acute morbidity, and mortality	39	3.49	0.64	3.45-3.52
RQ19	What are the gender differences in post-COVID illness experience and the coping needs of different groups of women, men and gender-diverse individuals	33	3.48	0.67	3.45-3.52
RQ21	What are the gender differences in the acceptance and uptake of non-pharmaceutical interventions of COVID-19 and what social determinants underpin them	34	3.47	0.51	3.44-3.50
RQ37	Were there gender differences in the impact of the closure of educational institutions on the mental health of students	35	3.46	0.61	3.42-3.49

RQ31	What types of incentives for COVID-19 vaccination are the most effective for different groups of women, men and gender diverse individuals and in what contexts	33	3.42	0.66	3.38-3.46
RQ20	How does long-COVID manifest in boys, girls, and adolescents	36	3.42	0.73	3.38-3.46
RQ34	How have people who are living with HIV or TB been affected by treatment and testing disruptions due to COVID-19	36	3.42	0.73	3.38-3.46
RQ18	What are the gender differences in economic and financial costs of illness due to post-COVID	34	3.41	0.74	3.37-3.45
RQ16	How does post-COVID affect neurological and psychiatric outcomes for men, women and gender diverse individuals	36	3.39	0.64	3.35-3.42
RQ10	Does sex and age influence the viral transmission rate in asymptomatic and symptomatic vaccinated individuals	34	3.38	0.78	3.34-3.43
RQ28	How is sex and gender vaccine data and evidence from vaccine trials, risk of adverse effects and population effectiveness communicated to national policy makers and to the public	32	3.38	0.79	3.33-3.42
RQ13	Are there sex and gender differences in post-COVID outcomes of different strains, particularly due to variants of concern	36	3.36	0.72	3.32-3.40
RQ9	What are the sex differences in the prevalence of multisystem inflammatory syndrome (MIS-C), other forms of acute COVID-19 morbidities in boys, girls and adolescents	34	3.32	0.73	3.28-3.37
RQ35	What was the impact of COVID-19 lockdown on how women and girls in low resource settings managed their menstrual needs	35	3.31	0.80	3.27-3.36
RQ15	How does post-COVID affect sex specific sexual and reproductive issues such as fertility, erectile dysfuntion, etc	32	3.31	0.69	3.27-3.35
RQ6	What are the gender differences in economic and financial costs of illness with COVID-19 infections, acute morbidity and mortality	36	3.31	0.67	3.27-3.34
RQ17	What are the sex and gender differences in post-COVID related impairment, functional ability, rehabilitation needs and the temporal nature of these experiences	34	3.26	0.67	3.23-3.30
RQ32	How did contraceptive use change due to the disruption of sexual and reproductive health services during the COVID-19 pandemic	34	3.24	0.74	3.19-3.26

RQ4	How do the infection, acute morbidity and mortality outcomes of gender-diverse groups differ to those of cis-gender male and females	32	3.22	0.75	3.17-3.26
RQ7	What is the effect of pre-existing conditions and therapies that relate to changes in sex-hormones on COVID-19 infections, acute morbidity and mortality	33	3.18	0.73	3.14-3.22
RQ38	Has vaccination impacted (positively or negatively) the mental health outcomes of men and women at the population level	36	3.17	0.85	3.12-3.21

Supplementary Table B: Sample size, Means, Standard deviations and 95% Confidence Intervals for Gender and COVID-19 Research Questions by Gender Equality

RQ	Label	N	Mean	SD	95% CI
RQ27	Do the gender differences in the trust, acceptance and uptake of COVID-19 vaccines vary across social categories (such as race, disability, migrant status, age, sexuality and pre-existing conditions)	36	3.56	0.61	3.52-3.59
RQ14	How does post-COVID conditions affect pregnant and postpartum women, and their foetus/infants across various contexts	36	3.53	0.74	3.49-3.57
RQ36	What was the impact of COVID-19 measures on the mental health outcomes of women, men, women, girls, boys, LGBTQI+ and gender-diverse persons	36	3.42	0.77	3.37-3.46
RQ26	Are there gender differences in the acceptance and uptake of COVID-19 vaccines	34	3.41	0.74	3.37-3.45
RQ39	How has COVID-19 measures affected the mental health of working women when help for childcare is not available, but they are still expected to work and manage their households	36	3.39	0.87	3.34-3.44
RQ5	What are the infection, acute morbidity and mortality levels of COVID-19 among pregnant and post-partum women, and their foetus/infants across various contexts	37	3.38	0.89	3.33-3.43
RQ29	What approaches are most effective at successfully integrating gender aspects in risk communication and community engagement to increase the uptake of COVID-19 vaccines	35	3.37	0.69	3.33-3.41

RQ2	How do the sex and gender determinants of COVID-19 infections, acute morbidity and mortality intersect with other social categories (such as race, disability, migrant status, age, sexuality, etc) across various contexts	38	3.37	0.85	3.32-3.41
RQ8	What is the duration and level of immune response, and antibody levels in COVID-19 patients by age, sex, severity and comorbidities (e.g., HIV, TB, NCDs, PCOS, immunocompromised individuals)	34	3.32	0.88	3.27-3.37
RQ33	What is the impact of the COVID-19 pandemic, including school closures, on the levels of early pregnancy, miscarriage and low-birth weight babies, unplanned pregnancies, safe and unsafe abortions, and their outcomes	37	3.30	0.81	3.25-3.34
RQ35	What was the impact of COVID-19 lockdown on how women and girls in low resource settings managed their menstrual needs	37	3.27	0.80	3.23-3.31
RQ40	What is the gendered impact of COVID-19 stress i.e. unemployment, unpaid care work on different chronic disease experiences and outcomes for cancer, cardiovascular disease, cardiometabolic disease, neurological and auto-immune conditions	36	3.25	0.84	3.20-3.30
RQ6	What are the gender differences in economic and financial costs of illness with COVID-19 infections, acute morbidity and mortality	36	3.25	0.77	3.21-3.29
RQ25	What are the most effective strategies for debunking myths and countering misinformation about COVID-19 for different groups of women, men and gender diverse individuals	37	3.24	0.76	3.20-3.28
RQ30	What channels and platforms for risk communication and community engagement are the most effective to increase the acceptance of COVID-19 vaccines among women, men and gender diverse individuals	35	3.20	0.76	3.16-3.24
RQ12	How do the sex and gender determinants of post-COVID conditions intersect with other social categories (such as race, disability, migrant status, age, sexuality, etc) across various contexts	36	3.19	0.82	3.15-3.24
RQ34	How have people who are living with HIV or TB been affected by treatment and testing disruptions due to COVID-19	37	3.19	0.88	3.14-3.24
RQ13	Are there sex and gender differences in post-COVID outcomes of different strains, particularly due to variants of concern	34	3.18	0.90	3.12-3.23
RQ22	How do the gender differences in the acceptance and uptake of non-pharmaceutical interventions of COVID-91 vary across social categories (such as race, disability, migrant status, age, sexuality)	35	3.17	0.71	3.13-3.21

George AS, *et al. BMJ Global Health* 2023; 8:e011315. doi: 10.1136/bmjgh-2022-011315

RQ23	What approaches are most effective at successfully integrating gender aspects in social and behavioural change communication to enhance uptake of non-pharmaceutical interventions	37	3.16	0.87	3.12-3.21
RQ11	What are the biological and social determinants underpinning sex and gender differences for post-COVID conditions	34	3.15	0.86	3.10-3.20
RQ32	How did contraceptive use change due to the disruption of sexual and reproductive health services during the COVID-19 pandemic	35	3.14	0.97	3.09-3.20
RQ16	How does post-COVID affect neurological and psychiatric outcomes for men, women and gender diverse individuals	36	3.14	0.90	3.09-3.19
RQ19	What are the gender differences in post-COVID illness experience and the coping needs of different groups of women, men and gender-diverse individuals	36	3.14	0.90	3.09-3.19
RQ1	What are the biological and social determinants underpinning sex and gender differences in COVID-19 infections, acute morbidity, and mortality	39	3.13	0.83	3.09-3.17
RQ17	What are the sex and gender differences in post-COVID related impairment, functional ability, rehabilitation needs and the temporal nature of these experiences	35	3.11	0.87	3.07-3.16
RQ24	What best practices can be used to ensure uptake of non-pharmaceutical interventions (e.g., physical distance, wearing masks) by different groups of women, men and gender diverse individuals	35	3.09	0.78	3.04-3.13
RQ37	Were there gender differences in the impact of the closure of educational institutions on the mental health of students	35	3.09	0.85	3.04-3.13
RQ28	How is sex and gender vaccine data and evidence from vaccine trials, risk of adverse effects and population effectiveness communicated to national policy makers and to the public	34	3.06	0.95	3.00-3.11
RQ20	How does long-COVID manifest in boys, girls, and adolescents	36	3.06	0.86	3.01-3.10
RQ3	Are there sex and gender differences in the infection, acute morbidity and mortality outcomes of different strains, particularly due to variants of concern	37	3.05	1.00	3.00-3.11
RQ9	What are the sex differences in the prevalence of multisystem inflammatory syndrome (MIS-C), other forms of acute COVID-19 morbidities in boys, girls and adolescents	33	3.03	0.81	2.98-3.08

RQ21	What are the gender differences in the acceptance and uptake of non-pharmaceutical interventions of COVID-19 and what social determinants underpin them	35	3.03	0.79	2.98-3.07
RQ31	What types of incentives for COVID-19 vaccination are the most effective for different groups of women, men and gender diverse individuals and in what contexts	36	3.03	0.84	2.98-3.07
RQ4	How do the infection, acute morbidity and mortality outcomes of gender-diverse groups differ to those of cis-gender male and females	34	3.00	0.95	2.95-3.05
RQ18	What are the gender differences in economic and financial costs of illness due to post-COVID	35	2.97	1.04	2.91-3.03
RQ10	Does sex and age influence the viral transmission rate in asymptomatic and symptomatic vaccinated individuals	35	2.91	1.04	2.86-2.97
RQ38	Has vaccination impacted (positively or negatively) the mental health outcomes of men and women at the population level	37	2.89	1.05	2.84-2.95
RQ7	What is the effect of pre-existing conditions and therapies that relate to changes in sex-hormones on COVID-19 infections, acute morbidity and mortality	33	2.82	1.01	2.76-2.88
RQ15	How does post-COVID affect sex specific sexual and reproductive issues such as fertility, erectile dysfuntion, etc	36	2.75	1.11	2.69-2.81

Supplementary Table C: Sample size, Means, Standard deviations and 95% Confidence Intervals for Gender and COVID-19 Research Questions by Urgency

RQ	Label	N	Mean	SD	95% CI
RQ26	Are there gender differences in the acceptance and uptake of COVID-19 vaccines	**35**	**2.66**	**0.59**	**2.62-2.69**
RQ27	Do the gender differences in the trust, acceptance and uptake of COVID-19 vaccines vary across social categories (such as race, disability, migrant status, age, sexuality and pre-existing conditions)	**36**	**2.61**	**0.69**	**2.57-2.65**
RQ25	What are the most effective strategies for debunking myths and countering misinformation about COVID-19 for different groups of women, men and gender diverse individuals	**37**	**2.51**	**0.73**	**2.47-2.55**

RQ3	Are there sex and gender differences in the infection, acute morbidity and mortality outcomes of differents strains, particularly due to variants of concern	**33**	**2.48**	**0.80**	**2.44-2.53**
RQ1	What are the biological and social determinants underpinning sex and gender differences in COVID-19 infections, acute morbidity, and mortality	**39**	**2.44**	**0.79**	**2.40-2.48**
RQ35	What was the impact of COVID-19 lockdown on how women and girls in low resource settings managed their menstrual needs	**36**	**2.42**	**0.77**	**2.37-2.46**
RQ8	What is the duration and level of immune response, and antibody levels in COVID-19 patients by age, sex, severity and comorbidities (e.g., HIV, TB, NCDs, PCOS, immunocompromised individuals)	**34**	**2.41**	**0.78**	**2.37-2.46**
RQ5	What are the infection, acute morbidity and mortality levels of COVID-19 among pregnant and post-partum women, and their foetus/infants across various contexts	**37**	**2.41**	**0.80**	**2.36-2.45**
RQ24	What best practices can be used to ensure uptake of non-pharmaceutical interventions (e.g., physical distance, wearing masks) by different groups of women, men and gender diverse individuals	**34**	**2.38**	**0.74**	**2.34-2.42**
RQ39	How has COVID-19 measures affected the mental health of working women when help for childcare is not available, but they are still expected to work and manage their households	**36**	**2.36**	**0.64**	**2.33-2.40**
RQ2	How do the sex and gender determinants of COVID-19 infections, acute morbidity and mortality intersect with other social categories (such as race, disability, migrant status, age, sexuality, etc) across various contexts	**39**	**2.36**	**0.78**	**2.32-2.40**
RQ14	How does post-COVID conditions affect pregnant and postpartum women, and their foetus/infants across various contexts	**35**	**2.34**	**0.84**	**2.30-2.39**
RQ30	What channels and platforms for risk communication and community engagement are the most effective to increase the acceptance of COVID-19 vaccines among women, men and gender diverse individuals	**36**	**2.33**	**0.79**	**2.29-2.38**
RQ29	What approaches are most effective at successfully integrating gender aspects in risk communication and community engagement to increase the uptake of COVID-19 vaccines	**36**	**2.31**	**0.71**	**2.27-2.34**
RQ22	How do the gender differences in the acceptance and uptake of non-pharmaceutical interventions of COVID-91 vary across social categories (such as race, disability, migrant status, age, sexuality)	**33**	**2.30**	**0.68**	**2.26-2.34**

George AS, *et al. BMJ Global Health* 2023; 8:e011315. doi: 10.1136/bmjgh-2022-011315

Gender & Health Hub

RQ23	What approaches are most effective at successfully integrating gender aspects in social and behavioural change communication to enhance uptake of non-pharmaceutical interventions	**34**	**2.29**	**0.72**	**2.25-2.34**
RQ9	What are the sex differences in the prevalence of multisystem inflammatory syndrome (MIS-C), other forms of acute COVID-19 morbidities in boys, girls and adolescents	**33**	**2.27**	**0.80**	**2.23-2.32**
RQ21	What are the gender differences in the acceptance and uptake of non-pharmaceutical interventions of COVID-19 and what social determinants underpin them	**34**	**2.26**	**0.75**	**-2.31**<br>**2.22**
RQ31	What types of incentives for COVID-19 vaccination are the most effective for different groups of women, men and gender diverse individuals and in what contexts	**34**	**2.26**	**0.79**	**2.22-2.31**
RQ28	How is sex and gender vaccine data and evidence from vaccine trials, risk of adverse effects and population effectiveness communicated to national policy makers and to the public	**33**	**2.24**	**0.79**	**2.20-2.29**
RQ36	What was the impact of COVID-19 measures on the mental health outcomes of women, men, women, girls, boys, LGBTQI+ and gender-diverse persons	**36**	**2.19**	**0.75**	**2.15-2.24**
RQ4	How do the infection, acute morbidity and mortality outcomes of gender-diverse groups differ to those of cis-gender male and females	**33**	**2.18**	**0.77**	**2.14-2.23**
RQ13	Are there sex and gender differences in post-COVID outcomes of differents strains, particularly due to variants of concern	**34**	**2.18**	**0.87**	**2.13-2.23**
RQ34	How have people who are living with HIV or TB been affected by treatment and testing disruptions due to COVID-19	**37**	**2.16**	**0.76**	**2.12-2.20**
RQ40	What is the gendered impact of COVID-19 stress i.e. unemployment, unpaid care work on different chronic disease experiences and outcomes for cancer, cardiovascular disease, cardiometabolic disease, neurological and auto-immune conditions	**37**	**2.16**	**0.73**	**2.12-2.20**
RQ33	What is the impact of the COVID-19 pandemic, including school closures, on the levels of early pregnancy, miscarriage and low-birth weight babies, unplanned pregnancies, safe and unsafe abortions, and their outcomes	**36**	**2.14**	**0.80**	**2.10-2.18**
RQ32	How did contraceptive use change due to the disruption of sexual and reproductive health services during the COVID-19 pandemic	**34**	**2.09**	**0.79**	**2.04-2.13**

RQ38	Has vaccination impacted (positively or negatively) the mental health outcomes of men and women at the population level	**34**	**2.09**	**0.67**	**2.05-2.13**
RQ10	Does sex and age influence the viral transmission rate in asymptomatic and symptomatic vaccinated individuals	**35**	**2.09**	**0.82**	**2.04-2.13**
RQ11	What are the biological and social determinants underpinning sex and gender differences for post-COVID conditions	**36**	**2.08**	**0.77**	**2.04-2.13**
RQ37	Were there gender differences in the impact of the closure of educational institutions on the mental health of students	**36**	**2.08**	**0.73**	**2.04-2.12**
RQ6	What are the gender differences in economic and financial costs of illness with COVID-19 infections, acute morbidity and mortality	**36**	**2.08**	**0.81**	**2.04-2.13**
RQ20	How does long-COVID manifest in boys, girls, and adolescents	**36**	**2.06**	**0.71**	**2.02-2.09**
RQ16	How does post-COVID affect neurological and psychiatric outcomes for men, women and gender diverse individuals	**35**	**2.03**	**0.75**	**1.99-2.07**
RQ12	How do the sex and gender determinants of post-COVID conditions intersect with other social categories (such as race, disability, migrant status, age, sexuality, etc) across various contexts	**36**	**2.00**	**0.79**	**1.96-2.04**
RQ19	What are the gender differences in post-COVID illness experience and the coping needs of different groups of women, men and gender-diverse individuals	**36**	**1.97**	**0.74**	**1.93-2.01**
RQ17	What are the sex and gender differences in post-COVID related impairment, functional ability, rehabilitation needs and the temporal nature of these experiences	**33**	**1.94**	**0.70**	**1.90-1.98**
RQ7	What is the effect of pre-existing conditions and therapies that relate to changes in sex-hormones on COVID-19 infections, acute morbidity and mortality	**32**	**1.94**	**0.80**	**1.89-1.99**
RQ15	How does post-COVID affect sex specific sexual and reproductive issues such as fertility, erectile dysfuntion, etc	**35**	**1.80**	**0.83**	**1.75-1.85**
RQ18	What are the gender differences in economic and financial costs of illness due to post-COVID	**34**	**1.76**	**0.85**	**1.72-1.81**
