# Supplementary material for: A shared agenda for gender and COVID-19 research: priorities based on broadening engagement in science
Source: BMJ Glob Health. 2023 May 22;8(5):e011315. doi: 10.1136/bmjgh-2022-011315 (PMC10230361; doi:10.1136/bmjgh-2022-011315)
Supplement: Supplementary data [file bmjgh-2022-011315supp004.pdf]

Gender & COVID-19 Research Agenda-Setting

Draft Thematic Reports for External Consultation

Thematic group 2

Therapeutics & diagnostics

Thematic discussions on better integrating biological sex (female and male) and gender (women, men, and gender diverse individuals) into the research and development, testing, analysis, and reporting of COVID-19 prophylactic products, (such as vaccines), therapeutics, medical devices, diagnostics, and digital health interventions. This includes considerations of the actions, decisions and relationships between different stakeholders, and parts of the population (with multiple and intersecting identities) as product users and advocates, governments as product regulators, and the scientist-entrepreneurs who develop, fund research and market these products.

Contents

Therapeutics & diagnostics 1
Section 1: Introduction to the overall collaboration 3
Section 2. Thematic group participation and engagement 3
Section 3. Thematic group background 5
3.1 Definitions 5
3.2 Current understanding, challenges, gaps, and neglected areas (outline of key themes, to be finalised at the end) 6
Key themes 6
3.2 Desired impact of the proposed research on policy, programme, and community responses 14
Therapeutics, vaccines and pharmaceutical prophylaxis 14
Diagnostics 14
Digital health interventions 15
Personal protective equipment 15
Research strategies, relationships, regulations, and commercialisation 15
Target user participation and engagement in product development processes 16
3.3 Actors and strategies to implement and promote uptake of the research agenda 17
3.4 Population, contexts, study design/ methodologies 18
Populations: 18
Context: 19

Study design/methodologies 19
Section 4. Research questions proposed for prioritisation. 19
Therapeutics, vaccines, and other pharmaceutical prophylaxis 19
Diagnostics 21
Digital health interventions 21
Personal protective equipment 21
Research strategy, relationships, regulations, and commercialisation 21
Participation and engagement in product development processes 23
Section 5: References 23
Section 6: Tables with Results of Prioritised Questions 30

Section 1: Introduction to the overall collaboration

From the start of the COVID-19 pandemic, how it affected and continues to affect women, girls, men, boys, and gender-diverse groups are complex and evolving. Apart from the direct effects of COVID-19 illness, pandemic responses also amplified existing gender inequalities across multiple dimensions. Context and the intersecting influence of other social determinants or identities[1,2] also worsened the influence of gender during the pandemic, with combined effects on health.

Early high-level calls and advocacy from researchers[3] such as through the Gender and COVID-19 working group,[4] were made for gender considerations to be integrated into the crisis response. Nevertheless, real-time response to the gender dynamics was limited by extensive invisibility of the evolving situation, incomplete data systems and evidence gaps. As the world steps into the second year of the COVID-19 crises, given the gender dynamics involved, we must include gender in the investments being made in research informing both immediate action and long-term recovery from the health and socio-economic consequences of the pandemic.

The United Nations University International Institute for Global Health is co-convening a collaborative gender and COVID-19 research agenda-setting exercise, as part of its Gender and Health Hub's inaugural scope of work. The process is co-developed through real-time learning, and open calls to a broad range of stakeholders to comment and contribute to its design, scope, and content. Collective contributions and questions for prioritization are supported by a community discussion board (www.ghhbuzzboard.org). Please visit this discussion board for further information.

The output of the exercise will be a shared research agenda that can be utilized by researchers, funders, and policymakers to guide COVID-19 research investments and corresponding programming and policy actions by the health sector.

The draft thematic group reports emerging from this collective endeavour are a synthesized version of the contributions made to the discussion board combined with additional inputs from thematic group coordinators, co-leads, and steering committee members. They document participation and engagement to date, provide a background section outlining definitions, scope, gaps, impact, and audiences, before listing research questions for prioritisation.

We welcome your comments on the discussion board or through google drive to be posted on the discussion board to ensure we respect the inclusive and transparent ethos of the collaboration. If you comment via google drive, please make sure we can identify your comments (please do not comment anonymously). Given the devastating and dynamic nature of COVID-19, we must be inclusive but also timely.

Section 2. Thematic group participation and engagement

Co-leads: Bernadette Ateghang-Awankem (Germany), Evelyne Bischof (China) and Jeannette Wolfe (United States of America)

Steering committee focal point: Lavanya Vijayasingham (Malaysia)

Coordinator: Maryam Rumaney (South Africa)

Other contributors: 23

Name	Country of residence	Role and Organisation
Anjana Bhushan	India	Technical Officer, WHO Regional Office for South-East Asia
Asha George	South Africa	Professor, SARCHI Chair in Health Systems, Complexity and Social Change, University of West Cape
Avni Amin	Switzerland	Technical Officer, World Health Organization
Cara Tannenbaum	Canada	Professor in the Faculties of Medicine and Pharmacy at the Université de Montréal;<br><br>Scientific Director of the Institute of Gender and Health at the Canadian Institutes of Health Research
Carinna Hockham	United Kingdom	Research Fellow in Epidemiology, The George Institute for Global Health
Chris Battison	Canada	Research Database Administrator, Women's College Hospital, Toronto
Claire Standley	Germany/United States of America	Assistant Research Professor, Georgetown University
Diana Nakaweesa	Uganda	Secretary at the BoD, Young Mothers Support Group
Fui Ching Lam	Malaysia	Data Scientist Consultant, United Nations University International Institute for Global Health
Jacqui Stevenson	Switzerland	Gender consultant, World Health Organization
Johanna Riha	Malaysia	Research Fellow, United Nations University International Institute for Global Health
Johnson Jament	India	Postdoctoral Fellow, University of Sussex
Kamayani Bali Mahabal	India	Women and Health Together for Future (WHTF)
Kathryn H. Jacobsen	United States of America	Professor, George Mason University
Mamothena Mothupi	Kenya/South Africa	School of Public Health University of the Western Cape
Manasee Mishra	India	Public Health Researcher, Independent
Marianne Legato	USA	Director of the Foundation for Gender-Specific Medicine<br><br>Professor Emerita of Clinical Medicine at Columbia University College of Physicians & Surgeons and an Adjunct Professor of Medicine at Johns Hopkins Medical School
Marlise Richter	South Africa	Research Consultant, Health Justice Initiative
Prabha Thangaraj	India	Assistant Professor, Community Medicine, Trichy SRM Medical College Hospital and Research Centre
Rosemary Morgan	USA	Associate Scientist at Johns Hopkins Bloomberg School of Public Health
Sabine Oertelt-Prigione	Netherlands	Professor of Gender in Primary and Transmural Care; Radboud Institute of Health Sciences, Radboud UMC
Tasnim Azim	Bangladesh	WHO Regional Office for South-East Asia
Vita Mithi	Malawi	Consultant, Armref Data for Action in Public Health Research

Discussion modalities:

- www.ghhbuzzboard.org
- Thematic group online calls- open and expert

Reporting timelines:

Task	Date
One call with the co-ordinator, steering committee member, and co-leads	14-01-2021
Draft report 1 shared with co-leads	21-01-2021
Draft report 2 shared via the dashboard	29-01-2021
Draft report 3 shared with co-leads	03-02-2021
Draft report 3 shared via the dashboard	08-02-2021
Final report shared with participants	

Section 3. Thematic group background

3.1 Definitions

Term	Definition
Artificial intelligence (AI)	Programmes, designed by humans, that carry out robotic or automated functions.
Biological sex	Male or female assignment based on innate biological factors
Clinical algorithms	A stepwise plan for dealing with the clinical aspects relating to COVID-19.
Data algorithms	Artificial intelligence that uses machine learning principles to develop software and applications relating to COVID-19.
Diagnostics	Tests used to detect the presence of SARS-CoV-2.
Digital health interventions	Software and applications used in the collection, monitoring and evaluation of COVID-19 data.
Electronic devices	Technological hardware that uses software relating to COVID-19.
Gender	The socially constructed characteristics of women and men – such as the norms, roles and relationships that exist between them. Gender expectations vary between cultures and can change over time. It is also important to recognize identities that do not fit into the binary male or female sex categories. Gender norms, relations and roles also impact the health outcomes of people with transgender or intersex identities.

Medical intervention	A measure that is applied to improve health or alter the course of an illness. It can be used to prevent, diagnose, or treat disease.
Post-COVID condition	Also known as 'Long-COVID' or also 'Post-acute sequalae of COVID-19'. This is a condition that occurs where despite the absence of an active infection, those previously infected with COVID-19 do not recover for several weeks or months following the onset of symptoms.
Precision medicine	The study and application of targeted medicine based on specific genetic manifestations and presentations, that is also modified by environment and lifestyle.
Risk assessment tools	Measures used to evaluate the degree of hazards associated with a COVID-19 therapeutic or technological intervention.
Therapeutics	Medicines and treatments for COVID-19 and related symptoms
Vaccine	A prophylactic substance used to stimulate the production of antibodies and provide immunity against COVID-19.

3.2 Current understanding, challenges, gaps, and neglected areas (outline of key themes, to be finalised at the end)

Key themes

Main category	Sub-themes
Therapeutics, vaccines & other pharmaceutical prophylaxis<br><br>(vaccines, pharmaceutical, biologics i.e., antivirals, steroids, plasma etc.)	● Sex-differentiated optimal vaccine dosing<br>● Sex differences in human biology (biochemistry, immunology etc) as factors in target identification for therapeutic development<br>● Sex differences in adverse events, effectiveness, duration of immunity, strain protection, variations based on hormonal cycles associated with vaccine and therapeutics<br>● Contraindications i.e., with hormonal contraception use, long-term hormonal use (transgender, menopause etc) other medications, and other conditions<br>● Sex differences in other medical interventions such as receiving and donating plasma, non-pharmaceutical hospital interventions to treat acute COVID-19<br>● Therapeutics for post-COVID condition and its symptoms
Diagnostics and other medical devices (digital health interventions, personal protective equipment)	● Identifying and reporting sex differences in human biology (biochemistry- concentration and normal ranges of molecules, immunology and immune system reactions or pathways etc) as target identification of biomarkers<br>● Sex differences or heterogeneity in sensitivity, specificity & accuracy in diagnostic products<br>● Integration of sex and gender into clinical algorithm and assessment scale development for clinical diagnosis, and treatment approaches

	• Sex differences and considerations in other medical interventions- device-based and non-pharmaceutical hospital interventions to treat acute COVID-19<br>• Sex and gender considerations in the design of digital health interventions, and datasets- artificial intelligence & machine learning algorithms and training data sets<br>• Personal protective equipment (PPE)- design that integrates sex, gender, and intersectionality differences to meet the needs of different groups of users.
Research strategy, relationships, regulations, and commercialisation	• Dynamics between academia, regulators, and industry<br>• Leveraging existing regulatory strategies, improving enforcement, and empowering regulators to demand relevant analysis to inform their decision-making.<br>• Commercialisation and value framing of sex and gender-sensitive decision-making
User participation and engagement in product development processes	• Representation of multiple groups of women, men, and gender diverse individuals with other intersectional identities i.e., pregnant, lactating, older, from marginalised groups, from the global south, transgender, and intersex groups in clinical trials<br>• Global north-south balance, and ethical conduct of clinical trials<br>• User and patient engagement early in the development process<br>• Trust-building and acceptability of safety profiles by men, women and gender-diverse individuals

Synthesis of gaps and discussions

The global health R&D community rallied swiftly to accelerate research and investments in product innovation[5] for COVID-19 diagnosis and management. Accordingly, product development and market entry for a range of diagnostics, therapeutics, vaccines, and other medical interventions (such as protective equipment for health workers, digital health interventions for disease surveillance and management) occurred at unprecedented speed.

Amidst these achievements, the integration of sex and gender considerations in the mainstream research agendas and blueprints for COVID-19 therapeutics, vaccines, diagnostics and other interventions, have been neglected. This is despite ongoing calls, and early attention to gender inequalities in many other domains of the COVID-19 response.

This is a critical gap because of sex differences in infectious disease pathology and outcomes, that have been consistently documented in other respiratory tract infections (including influenza, SARS, MERS[6–8]), and also extend to therapeutic and vaccine outcomes.[9,10] For instance, evidence from past vaccines show that females tend to mount a stronger immune system response to vaccines and induced immunity[7,11]- a phenomenon that is also observed in current post-market surveillance of COVID-19 vaccines (though rare).[12,13]

A consequence of not having diagnostics, prophylaxis and therapeutics that account for such sex-differences, is the perpetuation of harm, unmet need and sub-optimal management strategies, missed opportunities for novel innovation and limited autonomy and knowledge for informed medical decision-making for half of the global population.[14–17] A classic example of this harm and unmet need is from cardiovascular conditions, where women's historical exclusion and under-representation in trials has led to blindspots in 'sex-specific atypical' presentations,

especially prior to a cardiac event,[18,19] and women's tendency to receive less diagnostic investigation and aggressive interventions.[20]

Such biases and blindspots are also perpetuated by pre-existing global inadequacies and inconsistencies in integrating sex and gender in basic science and its translation intodiagnostics and therapeutics innovation. Females have also been excluded in past medical research due to a male bias in medical research, perceived complexity in accounting for female hormonal heterogeneity, their hesitance to participate in trials, and foetal protectionary ethics that then lead to perceived increased liabilities, risk and costs of including females, especially those of 'child-bearing potential'.[21,22]

Regulatory progress to counter this gender bias in policy, practice and decision-making has been noted in the past twenty years in the USA, Canada, and the European Union. Many top-tier peer-reviewed journals have signalled commitment towards stronger reporting of sex and gender variables in reports, such as through endorsing the SAGER guidelines.[23] Nevertheless, these policies and commitments are often not enforced, and they have been sidelined during this pandemic.[24]

A year and a half into the pandemic, there are still opportunities to improve the science and integrate sex and gender into key domains of research and development, including the global research blueprint and agenda, for vaccine, diagnostics, therapeutics development, personal protective equipment design, basic science research, participant engagement and representation.

COVID-19 presents a policy window to shift stakeholder perceptions, accelerate action, and adapt the ecosystem of policies more broadly. This requires synergistic efforts from regulatory agencies, as well as from journal editors, private and public research funders, academic groups, research laboratories and consortiums, industry bodies.

Therapeutics, vaccines and other pharmaceutical prophylaxis

Vaccine safety and dosing regimens in female adults, adolescents, and children

Group discussions heavily centred on vaccine studies, and the need to explore how sex and gender contribute to dosing, safety, and efficacy profiles, the duration of protection induced, by the different types of vaccines across all sex and gender groups, and strain of the virus.

Females tend to mount a higher and longer anti-body response than males provided the same vaccine dose, potentially ontributing to their experience of more side effects, and also provide cause for a sex-differentiated dosing regimen.[9,11] Sex differences in response have been observed after vaccinations against influenza, Human Papillomavirus (HPV),[25–27] yellow fever, rubella, measles, mumps, hepatitis A, hepatitis B, herpes simplex type 2, rabies, smallpox and dengue viruses.[28] Other factors also influence the response to vaccinations.[29] These include genetic, microbiome, and physiological factors, underlying health and pre-existing conditions, behavioural factors (such as exercise, stress, smoking, environmental factors), and vaccine-related factors (including type, strains, adjuvants, vaccination site, needle size, time of day, etc.) that contribute to sex- and gender-specific vaccine responses and outcomes.[30] More broadly, across all pharmaceuticals, sex-differences in pharmacokinetics and bioavailability, and the subsequent dosing regimens that do not consider these differences, may also contribute to the higher risk of adverse events in females.[31]

The current observations of post-vaccination adverse events (COVID-19) are female-disproportionate.[12,13,32] Serious adverse events including anaphylactic and non-anaphlactic events are rare, and the recent (rare) observations of cerebral venous sinus thrombosis (blood clots) after receipt of Oxford- Astra Zeneca and Johnsons & Johnsons (adenovirus vaccines) that have caused cessation of vaccination roll-out of these products in some countries, are similarly female-disproportionate.

Many possible reasons can contribute to these outcomes, including the women-centric uptake and rollout patterns, given the prioritization of healthcare workers and other frontline workers in the service sectors who are predominantly women.[33] For instance, broader prevalence of cerebral venous sinus thrombosis is known to be female-disproportionate without the specific use of adenovirus vaccines, where use of oral contraception is a known risk factor.[34,35]More focused research is needed on the causes of these manifestations, as well as solutions to optimize the balance of risk and benefits across women, men, and gender-diverse groups.

Indeed, a hypothesis to test is if a lower dose can be just as effective but safer for use in women.[27] This has previously been studied with the use of half doses of influenza vaccines.[27,36]If this is found to be true, then available vaccine supplies can be extended for use to more people, thereby supporting a faster and more equitable supply of scarce vaccines.

Additionally, many paediatric vaccines are known to have protective non-specific effects on resistance to other infections, prevention of complication such as pneumonia and sepsis, and all-cause mortality, but these outcomes are contingent on vaccine-types and sex.[28,37,38] The use of many non-live vaccines have been linked with detrimental non-specific effects in girls, such as increased susceptibility to infection (not related to vaccines), and increases in all-cause deaths from pneumonia and sepsis.[28] These considerations need to be investigated as the vaccines are trialled in adolescent and paediatric populations.

Precision medicine can provide an approach to explore and understand the role of epigenetics in sex-differentiation in vaccine-related adverse events in COVID-19. Pre-prints of a genetic study report that about 85 potential genes are not only sex-differentiated in the expressions, but also are related to COVID-19 as well as the vaccine-related adverse events of clinical consequence. Of note, the NLRP3 inflammasome and the NR3C1 glucocorticoid receptors are suggested to have promising links to sex-differentiation in adverse events.[39] Further work in this space, along with the use of artificial intelligence and machine learning approaches, can also use big data to find approaches to mitigate the rare serious adverse events.

Exclusion of pregnant women in trials and consequent lack of data to support their inclusion in vaccination programmes.

Pregnant women with COVID-19 are known to have a higher risk of preeclampsia/ eclampsia, severe COVID-19 infections, intensive care unit admission, requiring mechanical ventilation, maternal mortality and having a preterm birth.[40–42] Yet, they have been systematically excluded from earlier phase 3 trials, limiting data that would have supported their inclusion in earlier vaccine deployment phases. Safety outcomes have since been established through tracking outcomes in the post-market phase.[43] Results from such databases may be able to support policy change and the formal inclusion of women in vaccination roll-outs.

Pfizer was the first innovator to announce a formal phase two and three study amongst 4000 pregnant women at 24 to 34 weeks of pregnancy.[44] Nevertheless, it is common industry practice to not pursue registration or indications for use in pregnant populations.[45,46] In many cases, the

ethics prioritize the health of the fetus, and the liabilities of testing is perceived to be risky, and unviable for returns of investments.[45] Similar experiences were reported during the Ebola crisis. Even though there was disproportionate mortality amongst pregnant women, they were excluded from clinical trials for vaccines, thereby indirectly leading to many avoidable deaths.[47]

Diagnostics and other medical devices

Diagnostics

Sex differences that emerge from genetic, biochemical, or physiological domains have been established across multiple other diagnostic tests and clinical algorithms in other therapeutic areas. For instance, the use of sex-specific thresholds or reference ranges in the use of high-sensitivity cardiac troponin I assays, can identify up to 5 times more women with myocardial injury than men, suggesting that use of a generic threshold is more sensitive to detect men with myocardial injuries.[48] In another example, serum proteomic biomarkers hold the potential to be used in cancer, neurological and auto-immune disease diagnosis, but are rarely used on account of lack of reproducibility.[49] In a study on the variations of 171 serum proteins, 96 varied by sex and 66 had differed between females who were oral contraception users, post-menopausal and at follicular and luteal phases of the menstrual cycle, and yieled a high number of simulated errors when these aspects were not considered.[49]

In some case, unrelated infections, interactions with other medications or procedures can also influence the test results of diagnostics tests. COVID-19 vaccinations are known to induce false positives in mammograms, when there is lymphadenopathy (swelling of the lymph nodes)- a common immune system occurrence when the body is building protection.[50] Current advice is to schedule vaccinations or mammograms at a gap of four to six weeks of each other. [50]

COVID-19 diagnostics that are based on markers with sex-differented concentrations along the infection and immune response pathway can similarly produce lower accuracy in one sex group. We already know that COVID-19 outcomes differ based on biological sex, and differentiation in immune system reaction pathways have been reported.[51,52] There is hence a likelihood that these differences could also contribute to target biomarker concentrations, or strength of its roles in the diagnostic process. For example, laboratory testing find higher serum calcium and sodium levels, shorter period of virus shedding and a higher, more robust proinflammatory cytokines in females. In contrast in males, there is a lower adaptive T-cell response, and higher serum C-reactive protein levels, elevated erythrocyte sedimentation rate, raised levels of alanine aminotransferase, aspartate aminotransferase, γ-glutamyl transferase, ferritin, fibrinogen, interleukin-8, lactate dehydrogenase, and a more activated partial thromboplastin time.[51,52]

A small observational cohort study reports differences in antibody test sensitivity by sex, age, and test-type, where sensitivity was found to be highest in middle-aged men. Another small observational study reports that antibody levels also differ by sex based on severity of COVID-19 acute infections, where higher plasma concentrations of antibodies are found in men with severe infections, than women. Similarly, a study by Klein et al (2021), suggests that being male sex, and hospitalization for COVID-19 are predictors of higher antibody levels in plasma. Authors of the studies call for further validating studies to determine the influence of sex and gender on

antibodies. This also has implications on how we use and build reference ranges using antibodies, and other similar markers as diagnostic tools in COVID-10.

Recent systematic reviews on COVID-19 have not reported if and how sex influences heterogeneity, accuracy, sensitivity and specificity of polymerase chain reaction and rapid tests, or serological anti-body testing methods.[53,54] One study reports that there were no strong associations from sex in their modelled analysis of time to sero-reversion- the half-life of antibody clearance, time to lower production rate and level of reduction, using two commercial assays.[55] There is more room to ascertain if current and future diagnostic products are sex-differentiated in accuracy.

Medical devices and digital health interventions

Digital health interventions, including smartphone apps, have been used in contact-tracing, tracking vaccine outcomes, symptoms tracking and mental health management. Drawing broadly from literature on the gender-sensitive development of such technologies, product innovators should address gender and biological sex differences in their conceptualisation and design. This can be accomplished by factoring in sex and gender in algorithms, and including balanced representation of women, men and gender diverse people in the training data sets. When products do not account for gender biases, the generated products are likely to have higher rates of errors for applications in women, such as male-coder biases in decision-making,[56] or the lack of training dataset representation of females.[57]

Personal protective equipment (PPE)

Pre-pandemic literature highlights that many PPE products (masks, shields, gowns, bodysuits, respirators, etc) are designed based on the Caucasian male template, and alternative sizing is not often designed, produced or easily accessible.[58,59] Even now, nearly 1.5 year into the pandemic, sex and gender data or considerations are often not factored into product design, resulting in more failed fit-tests in female workers, where masks, goggles and gowns are often too loose for female or ethnic minority workers, and do not fit to provide adequate protection.[60,61] The risk of infection after intubating COVID patients was found to be higher in female healthcare workers, than in male healthcare workers.[62] Further study of the proportion of frontlines that fail fit-tests, and their subjective assessments of needs should be incorporated into the design and procurement processes.

Research strategy, relationships, regulations, and commercialisation

Accountability to strengthening scientific rigour by integrating sex and gender in COVID-19 specific R&D efforts has not been a priority in reporting, enforcement of existing policies by scientist-innovators, journal editors, regulatory and funding agencies. The work to report on these domains may also be seen to delay the timeliness of COVID-19 R&D outputs.

COVID-19 trial protocol design, analysis, and reporting

Authors of COVID-19 trial reports have not done well in analysing and reporting sex-disaggregated data.[24] An analysis up to mid-2020, found that of 2,484 registered COVID-19 trials identified on clinicaltrials.org, 16.7% mentions sex/gender as a recruitment criterion, and 4.1% allude to sex/gender in the reporting phase.[63] Even highly visible global trials, such as SOLIDARITY, have not included sex-disaggregated analysis and outcomes reporting in peer-reviewed publications.[64] Subsequent guidelines and systematic reviews based on trials have similarly not included sex and gender variables in reports, and would be challenged to do so.[65]

Published clinical trial reports of vaccines available in the market indicate the inclusion of males and females, but do not analyse and report sex-disaggregated data for all outcomes, importantly, safety and adverse event data.[66–69] Power and sample size has been used as a rationale for the lack of statistical strength in the subgroup analyses. The interim phase three report of the Pfizer vaccine indicated that the trial was not adequately powered to 'definitively' provide strong evidence of efficacy in the subgroups analysed, including stratification by age, sex, ethnicity, and BMI.[69] For all analysed subgroups, in which more than ten cases of COVID-19 occurred, the lower limit of the 95% confidence interval (CI) for efficacy was more than 30%.[69] Althought the Oxford Astra-Zeneca trial was female-disproportionate to reflect the distribution of the healthcare workforce in the first phase of vaccine deployment, it did not discuss any sex-related factors that could contribute to the results.[13,70] Drawing from past observations where women tend to mount a stronger response to vaccinations, a hypothesis to test is whether the female disproportionate cohort over-represented the efficacy in males.

Even before the pandemic and despite commitments to reporting, such as based on the Sex and Gender Equity in Research (SAGER) guidelines, journals inconsistently publish sex-disaggregated data on drug efficacy, safety, and toxicity, and the industry rarely reports biological sex or gender differences on product labels.[21,24] This is observed during the pandemic, and there are renewed calls to implement and enforce the approach and guidelines more strongly.[24]

Over the years, there are efforts and policy changes from regulatory agencies such as the FDA, EMA and Health Canada, but these are viewed to be inconsistently enforced, or unable to shift practice to the magnitude and scale required.[21,71,72] Amidst COVID-19, there is need for more persuasive requests and better use of sex-disaggregated data in regulatory decision-making processes,[70] such as in the early review of clinical trial protocols by ethics committees[13] and regulators, and in market approval dossiers. Retrospective analysis,[73] and agreed timelines for submissions can be negotiated,[74] so that this data is provided promptly without delaying the market entry and access.

In the commercialisation process, there are suggestions to integrate sex and gender considerations, including any sex- differences in outcomes into the 'value-based therapeutic or diagnostics development' agenda,[75] and the value-framing of products based on tenets of precision medicine.[76] Sex differences in outcomes may inevitably restrict the market in some cases, but can also be framed as a niched competitive advantage (in outcomes) against the comparator or alternative therapeutic options on the market. However, equity concerns will need to be navigated- as in the case of excluding cis-gender women from HIV pre-exposure prophylaxis trials on account of expected low efficacy (Descovy)[77] and the higher cost-effectiveness of HPV vaccination of girls.[78,79] Further work and dialogues on this topic can provide enabling entry points to shift commercial strategies and practice.

There are pockets of academic and advocacy groups that lobby for further study, and more cohesive and consistent integration of sex and gender factors in research and development processes (outlined in Section 3.3: Actors and strategies to implement and promote uptake of the research agenda). Closer synergies between these groups, regulators, funders and journal editors and commercial innovators are required.

Target user participation and engagement in product development processes

COVID-19 trials have included both women and men, but the complex interactions between illness severity, age, ethnicity and race, and pre-existing conditions necessitate better inclusion

and representation of different groups of women, men, and gender-diverse people in COVID-19 trials. The WHO R&D Blueprint Good Participatory Practice for COVID-19 clinical trials: a toolbox provides a framework, methodology and entry point to study and implement better engagement and participatory processes. Agencies such as the National Institute for Health in the USA have published and socialised guidance on facilitating better inclusion.[80] Policy changes implemented in 2017 requires investigators to achieve better representation of ethnic and minority groups, and also provide disaggregated analysis and findings to clinicaltrials.gov by sex, gender, and race/ethnicity.[81–83] This has not yet been observed, or consistently achieved for COVID-19 trials, but provides a regulatory mechanism to advocate for, and implement this type of reporting.

Clinical trials within the past two decades have shown underrepresentation of people of a minority or non-white backgrounds (in the USA), and from other countries.[81,84,85] This is more evident in women of minority or ethnic backgrounds across therapeutic areas, such as cardiovascular diseases and oncology, where women are not adequately represented even based on the sex-distribution of prevalence of conditions. In vaccine trials however, recent analyses show that women have been slightly over-represented across all phases (relative to their population distribution), but older people, and those of ethnic or minority backgrounds (in the USA), were still found to be under-represented to different degrees.[81]

It is also necessary to move away from a binary conceptualisation of sex and gender, and to pay more attention to the inclusion of gender-diverse populations and provide nuanced analysis and reports of the outcomes of COVID-19 interventions, including vaccines in this population. Long-term hormone therapy (estrogen or testosterone) in transgender people produces changes in body physiology, composition and biochemistry, that can affect therapeutics safety, efficacy, pharmacokinetics and produce other interactions with therapeutics and diagnostics biomarkers.[86–88] The specific nuances and outcomes of the transgender population are often not reported in clinical trials beyond the HIV area, stemming also from the use of binary sex and gender categories in data systems. There are very few studies that study and establish the efficacy and safety profiles of medicines in this population.[86,89] At present, regulatory agencies do not identify transgender, intersex or another member of gender-diverse groups that should be included and represented in clinical trials. Alternatives to human testing that can be considered include the 'systems pharmacology' approach and pharmacokinetic modelling including the use of AI, and in vitro microphysiological models, particularly to test metabolism based on cytochrome activity, kidney transporter proteins and absorption kinetic.[86]

It is also imperative that women, gender diverse and ethnic groups (non-white) are represented from across the world. A global north bias is also prevalent in clinical research, where studies are conceptualised in high-income countries, and trials are often conducted on local populations. Geographical biases can exclude genetic, ethnic, or socio-cultural nuances that influence therapeutic data, and consequently affect the transferability or application of the data in particular settings. Regulatory levers are once again useful to shift practice. Regulatory agencies in some countries like Japan, China, and India, tend to request specific local data for market approval of new products. Of note during COVID-19, Pfizer did not proceed with the regulatory submission for market approval of its vaccine in India because the company was not willing to conduct in-country trials.[90]

Low-and middle-income country (LMIC) inclusion must also be coupled with a focus on navigating logistical challenges, as well as ethical research standards and adherence to Good Clinical Practice. Indeed there are known challenges in conducting trials, and recruiting participants in LMICs- limited operational infrastructure, capacity, or motivations, regulatory barriers and ethical conduct challenges.[91] For example, it is common practice to require 'women of childbearing

potential', or susceptible to and becoming pregnant (WoSuP) to be on adequate contraception during the duration of a clinical trial as a way to prevent the exposure of experimental products on foetuses, but there may be supply, structural and cultural restrictions that limit its use particularly in LMICs.[92] These challenges (i.e. the lack of access to contraception) should not prevent women in this category from being well-represented in clinical trials.

One way to achieve better representation, adherence and trial completion, is through patient engagement This is an established strategy across pharmaceutical lifecycle management, including its inclusion in early-phase product development and optimization. More specifically, patient engagement methods are used to understand unmet needs, user preferences, trial protocol development, development of access programmes and strategizing for market entry.[93–96] This approach should be used to engage with target user groups, specifically women, gender-diverse groups of different ethnic backgrounds, and pre-existing health conditions, such as those who are immunocompromised. Engagement processes, alongside participatory research methods, can also support trust-building, willingness to participate,[97] and the establishment of user preferences and socio-cultural acceptability of the product profiles, including safety, and administration method (oral, injection etc) early in the development process.

3.2 Desired impact of the proposed research on policy, programme, and community responses

The prioritised research should inform and support:

Therapeutics, vaccines and pharmaceutical prophylaxis

1. Target therapeutic profiles (TPP) that include a focus on reducing adverse effects in females, while maintaining optimal efficacy in males, using strategies such as differentiated dosing recommendations to achieve this.

2. Active collection, analysis and reporting of data of sex and age disaggregated data.
in pre-and post-market trials, real-world use, systematic reviews, and meta-analyses of studies.

3. Trial design and statistical methods that are adequately powered to anticipate and detect any potential sex-differentiated differences in outcomes.

4. Targeted representation of females, males, and gender-diverse groups in trials that is not just based on sex differences in prevalence, risk exposure etc, but with considerations of sex differences in disease and treatment outcomes, rates of progression, mortality rates etc.

5. Dosing regimens that optimally balance side-effects and effectiveness in females, males, and gender-diverse groups.

Diagnostics

1. Active collection, analysis, and reporting of sex and age disaggregated data
in sensitivity, specificity, and heterogeneity testing, and for real-world use.

2. Calibration of diagnostic products considers that accounts for any known sex differences in biochemistry and biomarker profiles.

3. Statistical methods, trial size and design that provide adequate power to anticipate and detect any potential sex differences in outcomes.

4. Prioritized development of mobile, cheap, and rapid options that are equally sensitive, specific and accurate in all sexes.

Digital health interventions

1. The integration of sex and gender differences within artificial intelligence (AI) product design, such as the adequate representation of all sexes in training datasets, and machine learning algorithms that predict, detect, and offer solutions based on sex and gender considerations.

Personal protective equipment

1. The design, production, and procurement of personal protective equipment (PPE) that reflect different gender, sex, and intersectional considerations, such as face and head sizes, and disabilities etc.

Research strategies, relationships, regulations, and commercialisation

1. The need to reframe this call using a science lens, building on principles of 'do no harm', precision medicine, equity, equality and social responsibility.

2. Active dialogues on the integration of sex and gender between academic experts, regulatory agencies, funders or commercial investors, and journal editors, that facilitate changes in practice.

3. The strengthening of the use of data in journalism will enable better public understanding of the importance, and the role, that biological sex and the full gender spectrum plays in therapeutics, diagnostics, and digital R&D.

4. Human research ethics committees that keep trial sponsors and study investigators accountable towards considering, analysing, and reporting the sex and gender dimensions in the study protocol, and make negative decisions or request revisions where sex and gender and inadequately integrated.

5. The use of behavioural and regulatory incentives (such as design support, tax break etc) and penalties (such as registration delays and rejection) to shift practice in research design, data collection and analysis and reporting, and the use of biological sex as a default variable in research design.

6. Standardized regulatory dossier reporting requirements such as through the International Council for Harmonisation of Technical Requirements for Pharmaceuticals for Human Use (ICH) guidelines and common technical dossier (CTD) standardized templates.

7. Requirements for drug product label (and monographs) to indicate efficacy and safety outcomes for all sexes, that has been reviewed through the regulatory approval processes.

8. Publication of publicly accessible data on sex differences in product outcomes.

9. Establish the commercial value and 'business case' to invest more in sex-disaggregated analysis and reporting such as through precision medicine discussions, and the development or repurposing of therapeutics for Post-COVID condition (women disproportionate).

10. Increased number of women in leadership positions in laboratories and research groups, including as lead and last authors of research reports, heads of regulatory agencies, funding decision-makers, and heads of pharmaceutical/ health technology innovations companies who will work towards and facilitate the shifts in practice required.

11. Awareness that promotes buy-in, and capacity-building through guidance tools and learning modules for scientists and innovators to consider, analyse, and report sex-disaggregated data.

12. Working synergistically with key decision-makers and influencers who can add to the 'amplification pipeline'- to make more 'noise'- frequently, effectively, and using voices of advocacy beyond the limited group of researchers, clinicians, policymakers etc.

Target user participation and engagement in product development processes

1. Scientific communication through the creation of targeted narratives for public understanding, on the importance of including biological sex and the full gender spectrum in therapeutic, diagnostic, and digital R&D.

2. Different groups of women, men and gender diverse individuals are consulted to establish their acceptability of side-effects and effectiveness profiles of prophylactic and therapeutic products.

3. The enhancement of public, regulatory, and expert trust in the R&D process across the gender spectrum.

4. Balanced representation of groups by sex, age, ethnicity, and other relevant personal identifiers in COVID-19 data collection and reporting.

5. The representation of under-represented groups, such as gender diverse individuals, and pregnant and lactating women, thereby preventing their neglect from the COVID-19 research sphere.

6. Establishing guidelines for the protection of pregnant and lactating individuals during their enrolment in COVID-19 clinical trials.

7. The inclusion of sex considerations in the R&D of COVID-19 therapeutics, diagnostics, and digital health interventions, into the curriculum of biomedical degree courses.

3.3 Actors and strategies to implement and promote uptake of the research agenda

A strategic way to embed a sex and gender lens more systematically in COVID-19 R&D is to apply strong requirements for sex-disaggregated analysis and reporting in proposed, ongoing, and completed studies (through retrospective reporting) on COVID-19 therapeutics, vaccines, diagnostics, and other health interventions.

Examples of conducive entry points to better include sex and gender considerations throughout the R&D process include:

- An update of WHO's guidance document to manufacturers of COVID-19 vaccines- "Considerations for evaluation of COVID-19 vaccines (November 2020)" which only calls for sex-disaggregation of efficacy data, and not safety and adverse events data from trials based on the recommended dosing regimen for the population.

- Report of sex-disaggregated data in SOLIDARITY[64] and other COVID-19 intervention trials (RECOVERY, DISCOVERY, REMAP-CAP and ACTIV) https://www.nejm.org/doi/full/10.1056/NEJMoa2023184

- Report of sex-disaggregated data in living systematic reviews and guidelines such as "A living WHO guideline on drugs for covid-19" [98] and "Interventions for the prevention and treatment of COVID-19: a living mapping of research and living network meta-analysis" (Cochrane)[65,99]

- Retrospective data analysis of vaccine studies for pre-market trials that have already been assessed by regulators, and the prospective review of proposed, ongoing and post-market vaccines outcomes databases, and clinical trials- by negotiating timelines, imposing conditional approvals and regulatory penalties to strengthen delivery and importance of sex-disaggregated trial data.

While COVID-19 therapeutics development is the focus of the agenda, COVID-19 is also a highly visible window of opportunity for policy change, and attention from key actors for the future, beyond the pandemic. Core actors that can work synergistically to facilitate longer-term shifts are listed below:

- Dossier evaluation teams, approval and decision-making committees, and leaders of national regulatory bodies such as the US Food and Drug Administration (FDA), the European Medicines Authority (EMA), Japan Agency for Medical Research and Development (AMED), National Medical Products Administration (NMPA) China, Central Drugs Standard Control Organisation (CDSCO) India, Therapeutic Goods Administration (TGA) Australia, and equivalent offices for the regulation of medical devices, blood products, biologics, and other health technologies.

- The pharmaceutical industry, medical device manufacturers, diagnostics innovators, and private companies.

- Regulatory and industry consortiums such as the International Council for Harmonisation of Technical Requirements for Pharmaceuticals for Human Use (ICH).

- National research funders such as NIH (USA) and CIHR (Canada)

- Top tier scientific journal editors can enforce commitment towards sex-disaggregated reporting: The Sex and Gender Equity in Research (SAGER) guidelines provide a roadmap for journal editors to reject manuscripts that do not disaggregate, report or discuss biological sex and gender considerations.

- Networks that create and update reporting checklists such as CONSORT, PRISM, STROBE, CHEERS, and the Cochrane systematic review guidelines, require updates.

- Research networks, think tanks and private consulting companies that study, or support the policy translation of the sex and gender differences in therapeutics and diagnostics, alongside the basic science and epigenetics that underlie the sex differences such as Optimmunize, The Society for Women's Health Research and The Organization for the Study of Sex Differences (OSSD); iGIANT, Matera alliance and other partners [100]

- WHO alongside current global research, and policy networks and initiatives, that have been established for mainstream COVID-19 innovation and product development i.e. The Access to COVID-19 Tools (ACT) Accelerator, SAGE, etc.

- Global health and infectious disease centres of excellence located within research and academia.

- Women's advocacy and patient-led organizations or communities (i.e., Long Covid patient associations, online groups such as Survivors Corps).

- The education sector, particularly at the tertiary (medical school) levels

3.4 Population, contexts, study design/ methodologies

Populations:

- Pregnant women
- Lactating women
- Women who intend to become pregnant in the short term, before and after using COVID-19 vaccine or therapeutics.
- Female adolescents and children
- Post-menopausal women
- Elderly women
- Gender-diverse people including transgender and intersex people.
- Individuals who use exogenous hormones, such as those on gender-affirming treatment, women of childbearing age, and females experiencing menopause who use hormone replacement therapy.
- Groups of people with multiple and intersectional identities and backgrounds i.e., race/ ethnicity, age, disabilities, or pre-existing conditions, etc.

Context:
The inclusion of biological sex and gender, where appropriate, in studies about COVID-19 diagnostics, therapeutics and digital R&D.

There should be balanced representation from the Global South and North, especially where a gender or ethnic difference is anticipated, or signalled to arise.

The main focus is on having existing or upcoming studies designed with biological sex and gender in mind, thereby giving it the necessary attention geared towards outcomes that are more accurate of the differences that occur between males and females.

Study design/methodologies

- Randomised, double-blind clinical trials.
- Observational cohort, and case-control studies
- Retrospective analysis of outcomes databases
- Systematic reviews and meta-analyses, with a focus on sex, age, race disaggregation
- Qualitative research on stakeholders, and users' perceptions, experiences, and preferences
- Health utility, preference and perceptions surveys
- Participatory action research
- Implementation and operational research i.e., on process bottlenecks and implementation challenges
- Using AI, machine learning and big data for predictive and extrapolation-based modelling i.e., for safety, efficacy, and kinetics profiles in gender-diverse groups
- Hypothesis generation and testing
- Costing studies and economic evaluation of intervention value

Section 4. Research questions proposed for prioritisation.

Total number of questions: 42

Therapeutics, vaccines, and other pharmaceutical prophylaxis

1. Are the sex differences established from past and ongoing immunology and other basic science research (genetic, hormonal, biochemical, physiological, microbiota etc) well integrated into the early target identification, and screening (for repurposing products) of potential therapeutic and prophylactic products for acute and post-infection phases of COVID-19?
2. How do genetics, hormones, biochemistry, physiology, microbiota, etc contribute to any sex-differentiated therapeutic or vaccines outcomes?

3. Does dosing, safety (adverse events) and efficacy data of the different classes of therapeutic interventions (pharmaceutical/ biologics/ hormonal supplements/plasma) for acute COVID-19, and Post-COVID condition (Long-COVID) differ by sex/gender, age, race and/or ethnicity?
    - If yes, what are the mechanisms that explain these differences?

4. Does dosing, safety, efficacy, and protective duration of the different Covid-19 vaccines differ by sex, gender and age?

5. What are sex differences in safety, dosing, efficacy and contraindications that emerge from the use of different adjuvants and non-active pharmaceutical ingredients in therapeutics and vaccines formulations?

6. Does biological sex and age influence asymptomatic, and symptomatic viral transmissibility rates in vaccinated patients?

7. Are COVID-19 vaccines safe for pregnant/lactating women, and their foetus/newborns?
   - What are the differences in safety profiles for pregnant women in the different types of COVID-19 vaccines?
   - Do the safety profiles differ by gestational age in the different trimesters?
   - Are there any association and tangible differences between individuals who gave birth after being vaccinated?

8. What are lessons and observations of sex differences in vaccines outcomes in paediatric populations, and how can this be integrated into paediatric dosing, and formulation studies, and product planning?

9. Are there vaccine-associated non-specific effects that either enhance or attenuate mortality and morbidity rates in children who have gotten COVID-19 vaccines and is this related to the biological sex of the child?

10. Is there any long-term impacts on future fertility from vaccinations in all sexes, including children and adolescents?

11. Does an individual's age, reproductive stage, use of hormonal supplementation, or cyclic endogenous hormonal variation influence COVID19 therapeutic and vaccine outcomes?
    - Are there any association with use of hormonal contraceptives?

12. What is the optimal dose, to balance the safety and efficacy of each COVID-19 vaccine in biological males and females?
    - Does this differ amongst intersexed or gender-diverse individuals (who may be taking supplementation or had gender-affirming surgery which may alter their hormonal physiology)?

13. Are there sex-based differences in response to other medical therapeutic procedures, such as non-invasive ventilation (administration of ventilatory support without using an artificial airway), proning (lying on your stomach, face down), and intubation (insertion of an artificial tube into the trachea) or use of prophylactic anticoagulation in hospitalized Covid-19 patients?

14. How does the incorporation and inclusion of sex differences as bio factors, biomarkers, or variables influence the progress of precision medicine in COVID-19 vaccines, therapeutic approaches, diagnostics, and outcomes?

15. How can sex as a variable be implemented into precision medicine approaches (precision multimodal imaging, multiomics), including clinical trials' planning and simulation, drug development and repurposing, development and adjustment of diagnostics and therapeutics, specifically vaccines?

Diagnostics

16. Does sex, age, and ethnicity, race as a source of heterogeneity, influence COVID-19 testing accuracy (sensitivity and specificity) in polymerase chain reaction (PCR) and rapid antigen tests including antibodies, reinfections, and specimens used? If yes, how?

17. Do COVID-19 diagnostic trials include biological sex differences in the standardisation and calibration process of testing equipment and devices?

18. Is there a sex difference in COVID-19 antibody production that produces sex differences in testing? Is this taken into consideration in the development and use of commercial assays, and when calibrating, and reporting results?

Digital health interventions

19. How can digital intervention R&D, including smartphone apps, and wearables consider sex, gender and intersectionality in their design, training data sets, application, and use? Which dimensions need further attention?

20. What indicators can be used to measure the extent of the sex and gender role in COVID-19 health applications?

21. How can algorithms be built to optimize the delivery of unbiased, scientific-based information that is deemed credible and actionable upon by different subgroups based on their gender/age and sociocultural influences?

Personal protective equipment

22. Are the current personal protective equipment (PPE) used for COVID-19 sex/gender-sensitive in design? What are the testing approaches, design principles and modifications that can enhance the gender-sensitivity of the products, that also prioritize affordability, accessibility, sustainability, and socio-cultural acceptability of products?

23. What is the proportion of women, men and gender-diverse people, with intersecting needs (i.e. disabilities, cultural headgear, etc) that fail PPE fit-tests, and what are the consequent alternatives available for them across various settings?

24. How effective are current PPEs in preventing exposure and infection? Are there sex, gender, or other intersectional differences in outcomes?

Research strategy, relationships, regulations, and commercialisation

25. How can study design features (such as power calculations & statistical approaches), be adapted to ensure that there is sufficient engagement and cohort representation of groups previously not included in clinical trials for therapeutics, diagnostics, and digital health interventions, such as adolescents, elderly women, pregnant and lactating females and women using hormonal contraception and those using hormone replacement therapy, gender diverse individuals, women with pre-existing conditions and use of immunosuppressants etc?

26. How can funders, innovators, academic scientists, be incentivized to use artificially intelligence algorithms and big data as training data sets, that include sex and gender and other dimensions of intersectionality as variables in high throughput screening, target validation, commercial value forecasts etc?

27. How can the media be used as a tool to get biological sex prioritised in the therapeutic, diagnostic, and digital R&D landscapes?

28. How can the industry be incentivized to choose and develop gender-sensitive, affordable, accessible, and socio-culturally acceptable products?

29. Do the commercialisation strategies of COVID-19 interventions have a gender bias? If yes, how can market research and commercialisation strategies incorporate sex and gender considerations in key decision-making points?

30. How can scientific journals better support the reporting of sex-differentiated analysis in research that supports the design and use of COVID-19 therapeutics, diagnostics, and digital health interventions?

31. What are the strategic, regulatory, industry-based approaches to create-buy in, incentivise and change the behaviour of scientist innovators, and commercial funders in designing and reporting with a higher consideration of biological sex and gender?
    - What are the science communication and framing approaches that can be used to obtain buy-in and change perceptions on the importance of sex and gender in therapeutics, diagnostics and health technology development?

32. During the COVID-19 product commercialisation process, how much of patented innovations include women's representation and contribution?
    - how can funders, industry and regulators enhance gender representation and inclusiveness in decision making throughout all stages of the research pipeline?

33. How can human research ethics committees play a more prominent role in ensuring sex and gender dimensions are well considered in trial protocols, its implementation, data collection, analysis, and reporting?

34. What are necessary changes in data management systems, clinical trial SOPs, workforce training etc required to support the differentiated collection of gender identity and biological sex at birth in clinical trial participant registration?

35. How can international standards for vaccine and therapeutic intervention trials be developed to incorporate biological sex and gender considerations for the evaluation of therapeutic safety and effectiveness?

36. How can national regulations for in-country therapeutic trials take biological sex, gender, age and ethnicity into account, thereby providing data on efficacy and adverse effects that more accurately reflects the anticipated outcomes in specific national populations?

37. How can regulatory bodies implement legal requirements for age, sex, gender and race data in trial information be made available to the public?

Participation and engagement in product development processes

38. How can researchers optimize research participation such that their study results more accurately reflect real-world populations that vary in gender, race, and age?

39. What safety and effectiveness profiles are acceptable to different groups of women, men and gender-diverse users?

40. What is the extent of the enrolment and participation of women in ongoing and completed COVID-19 clinical trials across various sites and countries? What are enablers and barriers in their willingness, adherences and completion of trial participation?

41. How can pregnant and lactating females be included in COVID-19 therapeutic trials once the adverse profile has been established in the general study cohort in earlier phase studies?

42. How can a user-friendly, interactive dashboard serve as a tool whereby the general public may interact to better understand the role that biological sex and gender plays in therapeutics, diagnostics, and digital health R&D?

Section 5: References

1 Hankivsky O, Kapilashrami A. Beyond sex and gender analysis: an intersectional view of the COVID-19 pandemic outbreak and response. 2020. https://mspgh.unimelb.edu.au/__data/assets/pdf_file/0011/3334889/Policy-brief_v3.pdf (accessed July 3, 2020).

2 Lokot M, Avakyan Y. Intersectionality as a lens to the COVID-19 pandemic: implications for sexual and reproductive health in development and humanitarian contexts. *Sex Reprod Health Matters* 2020; **28**: 1764748.

3 Wenham C, Smith J, Morgan R. COVID-19: the gendered impacts of the outbreak. *The Lancet* 2020; **395**: 846–8.

4 Gender and COVID19 Working Group. Gender & Covid-19 Working Group. Gend. Covid-19. 2020. https://www.genderandcovid-19.org/gender-working-group-page/ (accessed Jan 19, 2021).

5 Word Health Organization. R&D Blueprint and COVID-19. 2020. https://www.who.int/teams/blueprint/covid-19 (accessed July 9, 2020).

6 Channappanavar R, Fett C, Mack M, Eyck PPT, Meyerholz DK, Perlman S. Sex-Based Differences in Susceptibility to Severe Acute Respiratory Syndrome Coronavirus Infection. *J Immunol* 2017; **198**: 4046–53.

7 Klein S, Roberts CW, editors. Sex and Gender Differences in Infection and Treatments for Infectious Diseases. Springer, 2015 https://www-springer-com.link.unu.edu/gp/book/9783319164373 (accessed May 11, 2021).

8 Hawkes S, Buse K. COVID-19 and the gendered markets of people and products: explaining inequalities in infections and deaths. *Can J Dev Stud Rev Can Détudes Dév* 2020; : 1–18.

9 Flanagan KL, Fink AL, Plebanski M, Klein SL. Sex and Gender Differences in the Outcomes of Vaccination over the Life Course. *Annu Rev Cell Dev Biol* 2017; **33**: 577–99.

10 Klein SL, Morgan R. The impact of sex and gender on immunotherapy outcomes. *Biol Sex Differ* 2020; **11**: 24.

11 Klein SL, Pekosz A. Sex-based Biology and the Rational Design of Influenza Vaccination Strategies. *J Infect Dis* 2014; **209**: S114–9.

12 Shimabukuro T. COVID-19 vaccine safety update from CDC COVID-19 Vaccine Task Force. 2021; published online Jan. https://www.cdc.gov/vaccines/acip/meetings/downloads/slides-2021-01/06-COVID-Shimabukuro.pdf.

13 Heidari S, Goodman T. Critical sex and gender considerations for equitable research, development and delivery of COVID-19 Vaccines: Background paper. 2021. https://cdn.who.int/media/docs/default-source/immunization/sage/covid/gender-covid-19-vaccines-sage-background-paper.pdf?sfvrsn=899e8fca_15&download=true.

14 Huded CP, Johnson M, Kravitz K, *et al.* 4-Step Protocol for Disparities in STEMI Care and Outcomes in Women. *J Am Coll Cardiol* 2018; **71**: 2122–32.

15 Mody P, Pandey A, Slutsky AS, *et al.* Gender-Based Differences in Outcomes Among Resuscitated Patients With Out-of-Hospital Cardiac Arrest. *Circulation* 2021; **143**: 641–9.

16 Gendered Innovations in Science, Health & Medicine, Engineering and Environment. Medicine and Health Case Studies Demonstrate Gender Methods in Basic and Applied Research. 2021. https://genderedinnovations.stanford.edu/case-studies-medicine.html (accessed May 12, 2021).

17 Tannenbaum C, Ellis RP, Eyssel F, Zou J, Schiebinger L. Sex and gender analysis improves science and engineering. *Nature* 2019; **575**: 137–46.

18 Eindhoven DC, Hilt AD, Zwaan TC, Schalij MJ, Borleffs CJW. Age and gender differences in medical adherence after myocardial infarction: Women do not receive optimal treatment – The Netherlands claims database. *Eur J Prev Cardiol* 2018; **25**: 181–9.

19 Smolina K, Ball L, Humphries KH, Khan N, Morgan SG. Sex Disparities in Post-Acute Myocardial Infarction Pharmacologic Treatment Initiation and Adherence: Problem for Young Women. *Circ Cardiovasc Qual Outcomes* 2015; **8**: 586–92.

20 Walli-Attaei M, Joseph P, Rosengren A, *et al.* Variations between women and men in risk factors, treatments, cardiovascular disease incidence, and death in 27 high-income, middle-income, and low-income countries (PURE): a prospective cohort study. *The Lancet* 2020; **396**: 97–109.

21 Ravindran TS, Teerawattananon Y, Tannenbaum C, Vijayasingham L. Making pharmaceutical research and regulation work for women. *BMJ* 2020; : m3808.

22 Saenz C, Cheah PY, van der Graaf R, Henry LM, Mastroianni AC. Ethics, regulation, and beyond: the landscape of research with pregnant women. *Reprod Health* 2017; **14**: 173.

23 Heidari S, Babor TF, De Castro P, Tort S, Curno M. Sex and Gender Equity in Research: rationale for the SAGER guidelines and recommended use. *Res Integr Peer Rev* 2016; **1**: 2.

24 Palmer-Ross A, Ovseiko PV, Heidari S. Inadequate reporting of COVID-19 clinical studies: a renewed rationale for the Sex and Gender Equity in Research (SAGER) guidelines. *BMJ Glob Health* 2021; **6**: e004997.

25 Cheng I, Wang Y, Du J. Human Papillomavirus Vaccines: An Updated Review. *Vaccines* 2020; : 1–15.

26 Engler RJ, Nelson MR, Klote MM, VanRaden MJ, Huang C-Y, Cox NJ. Walter Reed Health Care System Influenza Vaccine C. *Arch Intern Med* 2008; : 2405–2414.

27 Klein SL, Pekosz A. Sex-based Biology and the Rational Design of Influenza Vaccination Strategies. *J Infect Dis* 2014; : 114– 119.

28 Aaby P, Benn CS, Flanagan KL, *et al.* The non-specific and sex-differential effects of vaccines. *Nat Rev Immunol* 2020; **20**: 464–70.

29 Zimmermann P, Curtis N. Factors That Influence the Immune Response to Vaccination. American Society for Microbiology, 2019.

30 Flanagan KL, Fink AL, Plebanski M, Klein SL. Sex and Gender Differences in the Outcomes of Vaccination over the Life Course. *Annu Rev* 2017; : 577–599.

31 Zucker I, Prendergast BJ. Sex differences in pharmacokinetics predict adverse drug reactions in women. *Biol Sex Differ* 2020; **11**: 32.

32 Menni C, Klaser K, May A, *et al.* Vaccine side-effects and SARS-CoV-2 infection after vaccination in users of the COVID Symptom Study app in the UK: a prospective observational study. *Lancet Infect Dis* 2021; **0**. DOI:10.1016/S1473-3099(21)00224-3.

33 Ledford H. COVID vaccines and blood clots: five key questions. *Nature* 2021; **592**: 495–6.

34 Hinnell C, Nadeau J, Lam V, Hill MD, Coutts SB. Sex Differences in Adult Cerebral Venous Sinus Thrombosis: A 10-Year Experience. *Can J Neurol Sci* 2012; **39**: 74–7.

35 Zuurbier SM, Middeldorp S, Stam J, Coutinho JM. Sex differences in cerebral venous thrombosis: A systematic analysis of a shift over time. *Int J Stroke* 2016; **11**: 164–70.

36 Engler RJM. Half- vs Full-Dose Trivalent Inactivated Influenza Vaccine (2004-2005): Age, Dose, and Sex Effects on Immune Responses. *Arch Intern Med* 2008; **168**: 2405.

37 Flanagan KL, Jensen KJ. Sex Differences in Outcomes of Infections and Vaccinations in Under Five-Year-Old Children. In: Klein SL, Roberts CW, eds. Sex and Gender Differences in Infection and Treatments for Infectious Diseases. Cham: Springer International Publishing, 2015: 273–312.

38 Flanagan KL, Plebanski M. Sex-differential heterologous (non-specific) effects of vaccines: an emerging public health issue that needs to be understood and exploited. *Expert Rev Vaccines* 2017; **16**: 5–13.

39 Venkatakrishnan AJ, Kumar-M P, Silvert E, *et al.* Female-male differences in COVID vaccine adverse events have precedence in seasonal flu shots: a potential link to sex-associated baseline gene expression patterns. *medRxiv* 2021; : 2021.04.01.21254798.

40 Villar J, Ariff S, Gunier RB, *et al.* Maternal and Neonatal Morbidity and Mortality Among Pregnant Women With and Without COVID-19 Infection: The INTERCOVID Multinational Cohort Study. *JAMA Pediatr* 2021; published online April 22. DOI:10.1001/jamapediatrics.2021.1050.

41 Taylor MM, Kobeissi L, Kim C, *et al.* Inclusion of pregnant women in COVID-19 treatment trials. *The Lancet* 2020; **1–6**.

42 Chmielewska B, Barratt I, Townsend R, *et al.* Effects of the COVID-19 pandemic on maternal and perinatal outcomes: a systematic review and meta-analysis. *Lancet Glob Health* 2021; : S2214109X21000796.

43 Shimabukuro TT, Kim SY, Myers TR, *et al.* Preliminary Findings of mRNA Covid-19 Vaccine Safety in Pregnant Persons. *N Engl J Med* 2021; **0**: null.

44 Pfizer. Pfizer and BioNTech Commence Global Clinical Trial to Evaluate COVID-19 Vaccine in Pregnant Women | pfpfizeruscom. 2021. https://www.pfizer.com/news/press-release/press-release-detail/pfizer-and-biontech-commence-global-clinical-trial-evaluate (accessed May 7, 2021).

45 Amir LH, Grzeskowiak LE, Kam RL. Ethical Issues in Use of Medications During Lactation. *J Hum Lact* 2020; **36**: 34–9.

46 Hamel S, McNair DS, Birkett NJ, Mattison DR, Krantis A, Krewski D. Off-label use of cancer therapies in women diagnosed with breast cancer in the United States. *SpringerPlus* 2015; **4**: 209.

47 Bischof E, Oertelt-Prigione S, Morgan R, Klein S, The Sex and Gender in COVID19 Clinical Trials Working Group (SGC), Gender and COVID19 Working Group. Towards Precision Medicine: Inclusion of Sex and Gender Aspects in COVID-19 Clinical Studies—Acting Now before It Is Too Late—A Joint Call for Action. *Int J Environ Res Public Health* 2020; **17**: 3715.

48 Lee KK, Ferry AV, Anand A, *et al.* Sex-Specific Thresholds of High-Sensitivity Troponin in Patients With Suspected Acute Coronary Syndrome. *J Am Coll Cardiol* 2019; **74**: 2032–43.

49 Ramsey JM, Cooper JD, Penninx BWJH, Bahn S. Variation in serum biomarkers with sex and female hormonal status: implications for clinical tests. *Sci Rep* 2016; **6**: 26947.

50 CDC. COVID-19 vaccination and other medical procedures. Cent. Dis. Control Prev. 2020; published online Feb 11. https://www.cdc.gov/coronavirus/2019-ncov/vaccines/expect/other-procedures.html (accessed May 13, 2021).

51 Wolfe J, Safdar B, Madsen TE, *et al.* Sex- or Gender-specific Differences in the Clinical Presentation, Outcome, and Treatment of SARS-CoV-2. *Clin Ther* 2021; : 1–16.

52 Takahashi T, Ellingson MK, Wong P, *et al.* Sex differences in immune responses that underlie COVID-19 disease outcomes. *Nature* 2020; **588**: 315–20.

53 Böger B, Fachi MM, Vilhena RO, Cobre AF, Tonin FS, Pontarolo R. Systematic review with meta-analysis of the accuracy of diagnostic tests for COVID-19. *Am J Infect Control* 2021; **49**: 21–9.

54 Bastos ML, Tavaziva G, Abidi SK, *et al.* Diagnostic accuracy of serological tests for covid-19: systematic review and meta-analysis. *BMJ* 2020; **370**: m2516.

55 Manisty C, Treibel TA, Jensen M, *et al.* Time series analysis and mechanistic modelling of heterogeneity and sero-reversion in antibody responses to mild SARS-CoV-2 infection. *EBioMedicine* 2021; **65**. DOI:10.1016/j.ebiom.2021.103259.

56 Aneja U, Ali SE, Imafidon A-M, Ram A. Gender Inequality: Making Technology the Solution, Not the Problem. 2019 https://www.chathamhouse.org/events/all/members-event/gender-inequality-making-technology-solution-not-problem.

57 Cirillo D, Catuara-Solarz S, Morey C, *et al.* Sex and gender differences and biases in artificial intelligence for biomedicine and healthcare. *Npj Digit Med* 2020; **3**: 1–11.

58 Ahmed SB, Dumanski SM. Sex, gender and COVID-19: a call to action. *Can J Public Health Rev Can Santé Publique* 2020; **111**: 980–3.

59 Flynn MA, Keller B, DeLaney SC. Promotion of alternative-sized personal protective equipment. *J Safety Res* 2017; **63**: 43–6.

60 Chakladar A, Ascott A. Personal protective equipment is sexist. The BMJ. 2021; published online March 9. https://blogs.bmj.com/bmj/2021/03/09/personal-protective-equipment-is-sexist/ (accessed May 8, 2021).

61 Ascott A, Crowest P, Sausmarez E de, Khan M, Chakladar A. Respiratory personal protective equipment for healthcare workers: impact of sex differences on respirator fit test results. *Br J Anaesth* 2021; **126**: e48–9.

62 El-Boghdadly K, Wong DJN, Owen R, *et al.* Risks to healthcare workers following tracheal intubation of patients with COVID-19: a prospective international multicentre cohort study. *Anaesthesia* 2020; **75**: 1437–47.

63 Brady E, Nielsen MW, Andersen JP, Oertelt-Prigione S. Lack of consideration of sex and gender in clinical trials for COVID-19. *medRxiv* 2020; : 2020.09.13.20193680.

64 WHO Solidarity Trial Consortium. Repurposed Antiviral Drugs for Covid-19 — Interim WHO Solidarity Trial Results. *N Engl J Med* 2021; **384**: 497–511.

65 Boutron I, Chaimani A, Devane D, *et al.* Interventions for the prevention and treatment of COVID-19: a living mapping of research and living network meta-analysis. *Cochrane Database Syst Rev* 2020. DOI:10.1002/14651858.CD013769.

66 Sadoff J, Le Gars M, Shukarev G, *et al.* Interim Results of a Phase 1–2a Trial of Ad26.COV2.S Covid-19 Vaccine. *N Engl J Med* 2021; **0**: null.

67 Baden LR, El Sahly HM, Essink B, *et al.* Efficacy and Safety of the mRNA-1273 SARS-CoV-2 Vaccine. *N Engl J Med* 2021; **384**: 403–16.

68 Voysey M, Clemens SAC, Madhi SA, *et al.* Safety and efficacy of the ChAdOx1 nCoV-19 vaccine (AZD1222) against SARS-CoV-2: an interim analysis of four randomised controlled trials in Brazil, South Africa, and the UK. *The Lancet* 2021; **397**: 99–111.

69 Polack FP, Thomas SJ, Kitchin N, *et al.* Safety and Efficacy of the BNT162b2 mRNA Covid-19 Vaccine. *N Engl J Med* 2020; **383**: 2603–2615.

70 Vijayasingham L, Bischof E, Wolfe J. Sex-disaggregated data in COVID-19 vaccine trials. *The Lancet* 2021; **397**: 966–7.

71 Mazure CM, Jones DP. Twenty years and still counting: including women as participants and studying sex and gender in biomedical research. *BMC Womens Health* 2015; **15**. DOI:10.1186/s12905-015-0251-9.

72 Geller SE, Koch AR, Roesch P, Filut A, Hallgren E, Carnes M. The More Things Change, the More They Stay the Same: A Study to Evaluate Compliance With Inclusion and Assessment of Women and Minorities in Randomized Controlled Trials. *Acad Med* 2018; **93**: 630–635.

73 Raparelli V, Norris CM, Bender U, *et al.* Identification and inclusion of gender factors in retrospective cohort studies: the GOING-FWD framework. *BMJ Glob Health* 2021; **6**: e005413.

74 SDG3 Global Action Plan for Healthy Lives and Well-Being: Gender Equality Working Group, Gender and Health Hub, United Nations University International Institute for Global Health. Guidance note and checklist for tackling gender-related barriers to equitable COVID-19 vaccine deployment. 2021. https://www.genderhealthhub.org/articles/guidance-note-and-checklist-for-tackling-gender-related-barriers-to-equitable-covid-19-vaccine-deployment/ (accessed April 21, 2021).

75 Smith P, Siciliani L, McKee M, Soucat A, Figueras J. Building on value-based health care: Towards a health system perspective. 2020. https://apps.who.int/iris/rest/bitstreams/1312516/retrieve.

76 Ginsburg GS, Phillips KA. Precision Medicine: From Science To Value. *Health Aff (Millwood)* 2018; **37**: 694–701.

77 Goldstein RH, Walensky RP. Where Were the Women? Gender Parity in Clinical Trials. *N Engl J Med* 2019; **381**: 2491–3.

78 Datta S, Pink J, Medley GF, *et al.* Assessing the cost-effectiveness of HPV vaccination strategies for adolescent girls and boys in the UK. *BMC Infect Dis* 2019; **19**: 552.

79 Kim JJ, Andres-Beck B, Goldie SJ. The value of including boys in an HPV vaccination programme: a cost-effectiveness analysis in a low-resource setting. *Br J Cancer* 2007; **97**: 1322–8.

80 US FDA. Dialogues on Diversifying Clinical Trials Successful Strategies for Engaging Women and Minorities in Clinical Trials. 2011 https://www.fda.gov/files/science%20&%20research/published/White-Paper-on-the-Dialogues-on-Diversifying-Clinical-Trials-Conference.pdf.

81 Flores LE, Frontera WR, Andrasik MP, *et al.* Assessment of the Inclusion of Racial/Ethnic Minority, Female, and Older Individuals in Vaccine Clinical Trials. *JAMA Netw Open* 2021; **4**: e2037640.

82 National Institute for Health. NIH Policy and Guidelines on The Inclusion of Women and Minorities as Subjects in Clinical Research | grants.nih.gov. 2017. https://grants.nih.gov/policy/inclusion/women-and-minorities/guidelines.htm (accessed May 8, 2021).

83 National Institute for Health. Inclusion Across the Lifespan. 2017. https://grants.nih.gov/policy/inclusion/lifespan.htm (accessed May 8, 2021).

84 Chen A, Wright H, Itana H, *et al.* Representation of Women and Minorities in Clinical Trials for New Molecular Entities and Original Therapeutic Biologics Approved by FDA CDER from 2013 to 2015. *J Womens Health* 2017; **27**: 418–29.

85 Feldman S, Ammar W, Lo K, Trepman E, van Zuylen M, Etzioni O. Quantifying Sex Bias in Clinical Studies at Scale With Automated Data Extraction. *JAMA Netw Open* 2019; **2**: e196700.

86 Cirrincione LR, Huang KJ. Sex and Gender Differences in Clinical Pharmacology: Implications for Transgender Medicine. *Clin Pharmacol Ther* 2021; **n/a**. DOI:https://doi.org/10.1002/cpt.2234.

87 Moseson H, Zazanis N, Goldberg E, *et al.* The Imperative for Transgender and Gender Nonbinary Inclusion: Beyond Women's Health. *Obstet Gynecol* 2020; **135**: 1059–1068.

88 Legato MJ, Bennett WL, Klein S, *et al.* Roundtable Discussion on COVID-19 Through a Sex and Gender Lens. *Gend Genome* 2020; **4**: 247028972095701.

89 Reisner SL, Poteat T, Keatley J, *et al.* Global health burden and needs of transgender populations: a review. *The Lancet* 2016; **388**: 412–36.

90 TheStar. Pfizer drops India vaccine application after regulator seeks local trial; Covid-19 total goes above 10.8 million. 2021 https://www.thestar.com.my/aseanplus/aseanplus-news/2021/02/05/pfizer-drops-india-vaccine-application-after-regulator-seeks-local-trial-covid-19-total-goes-above-108-million.

91 Alemayehu C, Mitchell G, Nikles J. Barriers for conducting clinical trials in developing countries- a systematic review. *Int J Equity Health* 2018; **17**: 37.

92 Couderc-Pétry M, Eléfant E, Wasunna M, Mwinga A, Kshirsagar NA, Strub-Wourgaft N. Inclusion of women susceptible to and becoming pregnant in preregistration clinical trials in low- and middle-income countries: A proposal for neglected tropical diseases. *PLoS Negl Trop Dis* 2020; **14**: e0008140.

93 Lowe MM, Blaser DA, Cone L, *et al.* Increasing Patient Involvement in Drug Development. *Value Health* 2016; **19**: 869–78.

94 Allarakhia M. Exploring open innovation with a patient focus in drug discovery: an evolving paradigm of patient engagement. *Expert Opin Drug Discov* 2015; **10**: 571–8.

95 Vat LE, Finlay T, Jan Schuitmaker-Warnaar T, *et al.* Evaluating the "return on patient engagement initiatives" in medicines research and development: A literature review. *Health Expect Int J Public Particip Health Care Health Policy* 2020; **23**: 5–18.

96 Harrington RL, Hanna ML, Oehrlein EM, *et al.* Defining Patient Engagement in Research: Results of a Systematic Review and Analysis: Report of the ISPOR Patient-Centered Special Interest Group. *Value Health* 2020; **23**: 677–88.

97 Thetford K, Gillespie TW, Kim Y, Hansen B, Scarinci IC. Willingness of Latinx and African Americans to Participate in Nontherapeutic Trials: It Depends on Who Runs the Research. *Ethn Dis* 2021; **31**: 263–72.

98 Rochwerg B, Siemieniuk RA, Agoritsas T, *et al.* A living WHO guideline on drugs for covid-19. *BMJ* 2020; **370**: m3379.

99 Boutron I, Chaimani A, Meerpohl JJ, *et al.* The COVID-NMA Project: Building an Evidence Ecosystem for the COVID-19 Pandemic. *Ann Intern Med* 2020; **173**: 1015–7.

100 Tannenbaum C, Day D. Age and sex in drug development and testing for adults. *Pharmacol Res* 2017; **121**: 83–93.

Section 6: Tables with Results of Prioritised Questions

Table 4: Prioritised gender and COVID-19 research questions by criteria

RQ	Label	Public Health	Gender equality	Urgency for policy
RQ33	In what way are sex and gender related variables integrated into national and global vaccine safety surveillance systems	**X**	**X**	**X**
RQ8	Does safety, efficacy, optimal dosing regime, and protective duration of the different COVID-19 vaccines differ in pregnant and lactating women, and their foetuses and infants/toddlers	**X**	**X**	**X**
RQ5	Does safety, efficacy and optimal dosing of different therapeutic interventions for COVID-19, and post-COVID conditions differ by sex, age, race	**X**	**X**	**X**
RQ4	Does safety, efficacy, optimal dosing regime and protective duration of the different COVID-19 vaccines differ by sex, age, race	X	X	X
RQ9	Does safety, efficacy and optimal dosing regimens of different therapeutic interventions for COVID-19, and post-COVID conditions differ in pregnant and lactating women, and their foetuses and infants/toddlers	**X**	**X**	**X**
RQ40	How can pregnant and lactating females be ethically, and safely included in phase 3 and 4 studies for COVID-19 R&D	X	X	
RQ1	How do the different aspects of human biology (i.e genetics, immunology, biochemistry, physiology, microbiota) contribute to sex-differences in pharmacokinetics, safety and efficacy of COVID-19 therapeutics and vaccines	**X**		
RQ39	What is the extent of the enrollment and participation of women in ongoing and completed COVID-19 clinical trials across various sites and countries	**X**	**X**	
RQ27	How can research ethics committees play a more prominent role in ensuring sex and gender dimensions are well considered in research trials		**X**	
RQ21	How can DHI algorithms used in the pandemic be built to correct for gender and race bias		**X**	
RQ37	How can scientific journals better promote the reporting of sex-differentiated analysis in research on COVID-19 therapeutics, diagnostics, and digital health interventions		**X**	
RQ38	How can regulatory bodies implement legal requirements for age, sex, gender, and race data in trial information to be made available to the public		**X**	
RQ13	What are the sex differences in COVID-19 vaccine outcomes in paediatric populations, and what are past lessons and experiences in paediatric vaccine studies that can be applied in dosing and formulation studies, and product planning			**X**
RQ22	Are the current personal protective equipment (PPE) used for COVID-19 sex/gender-sensitive in design			**X**
RQ16	Do sex, age and race influence COVID-19 testing accuracy (sensitivity and specificity) in polymerase chain reaction (PCR) and rapid antigen tests, including antibodies, reinfections, and specimens used			**X**
RQ32	How can international standards and templates for regulatory and evaluation dossiers, including for emergency use vaccine and therapeutic products during pandemics, be adapted to incorporate sex and gender considerations in the evaluation of therapeutic safety and effectiveness, and product registration process	**X**		
RQ30	How are regulatory agencies considering the sex and gender related factors in vaccine outcomes in evidence assessments and policy decision-making for emergency use approval	**X**		
RQ23	Given COVID-19, what is the proportion of women, men and gender-diverse people, with intersecting needs (i.e. disabilities, cultural attire, etc) that fail PPE fit-tests, and what are the consequent alternatives available			**X**

Supplementary Table A: Research Questions Prioritised by Public Health Benefit

RQ	Label	n	Mean	SD	95% CI
RQ40	How can pregnant and lactating females be ethically, and safely included in phase 3 and 4 studies for COVID-19 R&D	**26**	**3.85**	**0.46**	**3.81-3.88**
RQ33	In what way are sex and gender related variables integrated into national and global vaccine safety surveillance systems	**24**	**3.75**	**0.44**	**3.71-3.79**
RQ8	Does safety, efficacy, optimal dosing regime, and protective duration of the different COVID-19 vaccines differ in pregnant and lactating women, and their foetuses and infants/toddlers	**25**	**3.72**	**0.54**	**3.68-3.76**
RQ4	Does safety, efficacy, optimal dosing regime and protective duration of the different COVID-19 vaccines differ by sex, age, race	**24**	**3.67**	**0.56**	**3.62-3.71**
RQ5	Does safety, efficacy and optimal dosing of different therapeutic interventions for COVID-19, and post-COVID conditions differ by sex, age, race	**24**	**3.67**	**0.56**	**3.62-3.71**
RQ9	Does safety, efficacy and optimal dosing regimens of different therapeutic interventions for COVID-19, and post-COVID conditions differ in pregnant and lactating women, and their foetuses and infants/toddlers	**24**	**3.63**	**0.49**	**3.58-3.67**
RQ1	How do the different aspects of human biology (i.e genetics, immunology, biochemistry, physiology, microbiota) contribute to sex-differences in pharmacokinetics, safety and efficacy of COVID-19 therapeutics and vaccines	**28**	**3.61**	**0.63**	**3.56-3,56**
RQ39	What is the extent of the enrollment and participation of women in ongoing and completed COVID-19 clinical trials across various sites and countries	**25**	**3.60**	**0.65**	**3.55-3.65**
RQ21	How can DHI algorithms used in the pandemic be built to correct for gender and race bias	**24**	**3.54**	**0.59**	**3.49-3.59**
RQ30	How are regulatory agencies considering the sex and gender related factors in vaccine outcomes in evidence assessments and policy decision-making for emergency use approval	**25**	**3.52**	**0.59**	**3.47-3.57**
RQ15	Are there any long-term impacts on menstrual cycles and future fertility from vaccinations in all sexes, including children and adolescents	**22**	**3.50**	**0.67**	**3.44-3.56**
RQ38	How can regulatory bodies implement legal requirements for age, sex, gender, and race data in trial information to be made available to the public	**26**	**3.50**	**0.58**	**3.46-3.54**
RQ41	Does user-perceived value and acceptability of therapeutics and vaccines (i.e utility, willingness-to-pay and risk-benefit trade-offs in safety and efficacy profiles) differ between various groups of women, men and gender-diverse users	**25**	**3.44**	**0.71**	**3.38-3.50**
RQ25	What clinical trial study design and statistical analysis strategies efficiently and cost-effectively ensure quality sex and gender sensitive analysis and reporting for COVID-19 research	**26**	**3.42**	**0.64**	**3.37-3.47**
RQ27	How can research ethics committees play a more prominent role in ensuring sex and gender dimensions are well considered in research trials	**26**	**3.42**	**0.64**	**3.37-3.47**
RQ37	How can scientific journals better promote the reporting of sex-differentiated analysis in research on COVID-19 therapeutics, diagnostics, and digital health interventions	**26**	**3.42**	**0.64**	**3.37-3.47**
RQ14	Are there vaccine-associated non-specific effects that influence mortality and morbidity rates in children and conversely are there non-specific COVID-19 protection or risks from use of other vaccines (e.g. BCG)	**24**	**3.42**	**0.83**	**3.35-3.48**
RQ12	Does an individual's age, reproductive stage, natural cyclic hormonal variation, use of hormonal supplementation or contraceptives influence COVID-19 therapeutic and vaccine outcomes	**23**	**3.39**	**0.66**	**3.34-3.45**

RQ32	How can international standards and templates for regulatory and evaluation dossiers, including for emergency use vaccine and therapeutic products during pandemics, be adapted to incorporate sex and gender considerations in the evaluation of therapeutic safety and effectiveness, and product registration process	**26**	**3.38**	**0.75**	**3.33-3.44**
RQ13	What are the sex differences in COVID-19 vaccine outcomes in paediatric populations, and what are past lessons and experiences in paediatric vaccine studies that can be applied in dosing and formulation studies, and product planning	**23**	**3.35**	**0.78**	**3.28-3.41**
RQ34	How can funders, industry and regulators enhance gender representation and inclusiveness in decision making throughout all stages of the COVID-19 R&D pipeline	**25**	**3.32**	**0.75**	**3.26-3.38**
RQ26	What are the necessary changes required in data management systems, clinical trial SOPs, and workforce training, to support the collection of gender identity and biological sex at birth in clinical trial participant registration	**26**	**3.31**	**0.79**	**3.25-3.37**
RQ31	What are effective regulatory strategies for ensuring the design and reporting of COVID-19 research that considers sex and gender by scientist innovators, and commercial funders	**26**	**3.31**	**0.79**	**3.25-3.37**
RQ22	Are the current personal protective equipment (PPE) used for COVID-19 sex/gender-sensitive in design	**23**	**3.30**	**0.82**	**3.23-3.37**
RQ6	Do the use of different formulation strategies (i.e use of specific adjuvants, vectors and non-active pharmaceutical ingredients) contribute to any sex differences in safety, efficacy, dosing regimes and contraindications in therapeutics and vaccines	**23**	**3.30**	**0.76**	**3.24-3.37**
RQ7	Are there sex differences in the outcomes of other medical procedures, such as non-invasive ventilation (administration of ventilatory support without using an artificial airway), proning (lying on your stomach, face down), and intubation (insertion of an artificial tube into the trachea) or use of prophylactic anticoagulation in hospitalized COVID-19 patients	**23**	**3.30**	**0.82**	**3.23-3.37**
RQ36	How can media facilitate a stronger consideration of sex and gender in the therapeutic, diagnostic, and digital R&D ecosystems	**25**	**3.28**	**0.68**	**3.23-3.33**
RQ11	Does safety, efficacy, pharmacokinetics and optimal dosing of different therapeutic interventions for COVID-19, and post-COVID conditions differ amongst intersex or gender-diverse individuals who may be taking supplementation or had gender-affirming surgery which can alter their hormonal profile and physiology	**23**	**3.22**	**0.80**	**3.15-3.29**
RQ2	To what extent are the sex differences established in basic science research integrated into the early product development phase of potential therapeutic and prophylactic products for COVID-19	**24**	**3.21**	**0.78**	**3.14-3.27**
RQ18	Are there a sex, age and race differences in COVID-19 antibody production that produces sex differences in testing	**22**	**3.18**	**0.66**	**3.12-3.24**
RQ10	Does safety, efficacy, pharmacokinetics, optimal dosing regime, and protective duration of the different COVID-19 vaccines differ amongst intersex or gender-diverse individuals who may be taking supplementation or had gender-affirming surgery which can alter their hormonal profile and physiology	**24**	**3.17**	**0.76**	**3.10-3.23**
RQ28	Do public research funding, and private product planning and commercialisation strategies of COVID-19 interventions have a gender bias	**24**	**3.17**	**0.70**	**3.11-3.22**
RQ3	How can precision medicine approaches (the study of biofactors and biomarkers, precision multimodal imaging, multiomics) be applied to understand the influence of sex and gender in COVID-19 therapeutics, vaccines and diagnostics outcomes	**26**	**3.15**	**0.78**	**3.09-3.21**
RQ16	Do sex, age and race influence COVID-19 testing accuracy (sensitivity and specificity) in polymerase chain reaction (PCR) and rapid antigen tests, including antibodies, reinfections, and specimens used	**23**	**3.13**	**0.69**	**3.07-3.19**
RQ23	Given COVID-19, what is the proportion of women, men and gender-diverse people, with intersecting needs (i.e. disabilities, cultural attire, etc) that fail PPE fit-tests, and what are the consequent alternatives available	**25**	**3.12**	**0.88**	**3.05-3.19**

RQ35	What user-friendly, interactive tools can enable the general public to better understand the role that sex and gender play in COVID-19 R&D	**26**	**3.12**	**0.82**	**3.05-3.18**
RQ24	Are there sex, gender, or other intersectional differences in how effective PPE is in preventing exposure and infection to COVID-19	**23**	**3.09**	**0.90**	**3.01-3.16**
RQ19	How can DHI R&D, including for smartphone apps and wearables, consider sex, gender and intersectionality in their design, training data sets, output accuracy, etc	**24**	**3.08**	**0.93**	**3.01-3.16**
RQ17	Do COVID-19 diagnostic trials include sex differences in the standardisation and calibration process of testing equipment and devices	**22**	**3.00**	**0.76**	**2.93-3.07**
RQ20	How can digital R&D measure gender sensitivity in the acceptance, uptake and effectiveness of COVID-19 DHI	**25**	**2.88**	**0.88**	**2.81-2.95**
RQ29	How can funders, and innovators be incentivized to use artificial intelligence approaches that incorporate sex, gender and other dimensions of intersectionality in the COVID-19 R&D and commercialisation pipeline	**24**	**2.88**	**0.80**	**2.81-2.94**

Supplementary Table B: : Research Questions Prioritised by Gender Equality

RQ	Label	N	Mean	SD	95% CI
RQ40	How can pregnant and lactating females be ethically, and safely included in phase 3 and 4 studies for COVID-19 R&D	**26**	**3.85**	**0.46**	**3.81-3.88**
RQ8	Does safety, efficacy, optimal dosing regime, and protective duration of the different COVID-19 vaccines differ in pregnant and lactating women, and their foetuses and infants/toddlers	**24**	**3.63**	**0.65**	**3.57-3.68**
RQ39	What is the extent of the enrollment and participation of women in ongoing and completed COVID-19 clinical trials across various sites and countries	**26**	**3.54**	**0.71**	**3.49-3.59**
RQ33	In what way are sex and gender related variables integrated into national and global vaccine safety surveillance systems	**25**	**3.40**	**0.76**	**3.34-3.46**
RQ27	How can research ethics committees play a more prominent role in ensuring sex and gender dimensions are well considered in research trials	**26**	**3.38**	**0.85**	**3.32-3.45**
RQ5	Does safety, efficacy and optimal dosing of different therapeutic interventions for COVID-19, and post-COVID conditions differ by sex, age, race	**26**	**3.35**	**0.75**	**3.29-3.40**
RQ21	How can DHI algorithms used in the pandemic be built to correct for gender and race bias	**24**	**3.33**	**0.82**	**3.27-3.40**
RQ37	How can scientific journals better promote the reporting of sex-differentiated analysis in research on COVID-19 therapeutics, diagnostics, and digital health interventions	**26**	**3.31**	**0.79**	**3.25-3.37**
RQ38	How can regulatory bodies implement legal requirements for age, sex, gender, and race data in trial information to be made available to the public	**26**	**3.31**	**0.79**	**3.25-3.37**
RQ41	Does user-perceived value and acceptability of therapeutics and vaccines (i.e utility, willingness-to-pay and risk-benefit trade-offs in safety and efficacy profiles) differ between various groups of women, men and gender-diverse users	**26**	**3.31**	**0.93**	**3.24-3.38**
RQ32	How can international standards and templates for regulatory and evaluation dossiers, including for emergency use vaccine and therapeutic products during pandemics, be adapted to incorporate sex and gender considerations in the evaluation of therapeutic safety and effectiveness, and product registration process	**26**	**3.27**	**0.87**	**3.20-3.34**
RQ9	Does safety, efficacy and optimal dosing regimens of different therapeutic interventions for COVID-19, and post-COVID conditions differ in pregnant and lactating women, and their foetuses and infants/toddlers	**26**	**3.27**	**0.92**	**3.20-3.34**
RQ4	Does safety, efficacy, optimal dosing regime and protective duration of the different COVID-19 vaccines differ by sex, age, race	**25**	**3.24**	**0.93**	**3.17-3.31**
RQ30	How are regulatory agencies considering the sex and gender related factors in vaccine outcomes in evidence assessments and policy decision-making for emergency use approval	**26**	**3.19**	**1.02**	**3.12-3.27**
RQ34	How can funders, industry and regulators enhance gender representation and inclusiveness in decision making throughout all stages of the COVID-19 R&D pipeline	**26**	**3.15**	**1.01**	**3.08-3.23**
RQ15	Are there any long-term impacts on menstrual cycles and future fertility from vaccinations in all sexes, including children and adolescents	**23**	**3.13**	**1.01**	**3.04-3.22**
RQ28	Do public research funding, and private product planning and commercialisation strategies of COVID-19 interventions have a gender bias	**24**	**3.13**	**1.03**	**3.04-3.21**

RQ12	Does an individual's age, reproductive stage, natural cyclic hormonal variation, use of hormonal supplementation or contraceptives influence COVID-19 therapeutic and vaccine outcomes	**25**	**3.12**	**1.05**	**3.04-3.20**
RQ36	How can media facilitate a stronger consideration of sex and gender in the therapeutic, diagnostic, and digital R&D ecosystems	**26**	**3.12**	**1.07**	**3.03-3.20**
RQ2	To what extent are the sex differences established in basic science research integrated into the early product development phase of potential therapeutic and prophylactic products for COVID-19	**27**	**3.11**	**0.89**	**3.05-3.18**
RQ22	Are the current personal protective equipment (PPE) used for COVID-19 sex/gender-sensitive in design	**25**	**3.08**	**1.12**	**2.99-3.17**
RQ31	What are effective regulatory strategies for ensuring the design and reporting of COVID-19 research that considers sex and gender by scientist innovators, and commercial funders	**26**	**3.08**	**0.98**	**3.00-3.15**
RQ7	Are there sex differences in the outcomes of other medical procedures, such as non-invasive ventilation (administration of ventilatory support without using an artificial airway), proning (lying on your stomach, face down), and intubation (insertion of an artificial tube into the trachea) or use of prophylactic anticoagulation in hospitalized COVID-19 patients	**25**	**3.04**	**0.93**	**2.97-3.11**
RQ25	What clinical trial study design and statistical analysis strategies efficiently and cost-effectively ensure quality sex and gender sensitive analysis and reporting for COVID-19 research	**26**	**3.04**	**1.00**	**2.96-3.11**
RQ1	How do the different aspects of human biology (i.e genetics, immunology, biochemistry, physiology, microbiota) contribute to sex-differences in pharmacokinetics, safety and efficacy of COVID-19 therapeutics and vaccines	**28**	**3.04**	**0.96**	**2.97-3.10**
RQ6	Do the use of different formulation strategies (i.e use of specific adjuvants, vectors and non-active pharmaceutical ingredients) contribute to any sex differences in safety, efficacy, dosing regimes and contraindications in therapeutics and vaccines	**26**	**3.00**	**0.98**	**2.93-3.07**
RQ35	What user-friendly, interactive tools can enable the general public to better understand the role that sex and gender play in COVID-19 R&D	**26**	**2.96**	**0.96**	**2.89-3.03**
RQ26	What are the necessary changes required in data management systems, clinical trial SOPs, and workforce training, to support the collection of gender identity and biological sex at birth in clinical trial participant registration	**26**	**2.92**	**1.02**	**2.85-3.00**
RQ14	Are there vaccine-associated non-specific effects that influence mortality and morbidity rates in children and conversely are there non-specific COVID-19 protection or risks from use of other vaccines (e.g. BCG)	**24**	**2.92**	**1.14**	**2.82-3.10**
RQ23	Given COVID-19, what is the proportion of women, men and gender-diverse people, with intersecting needs (i.e. disabilities, cultural attire, etc) that fail PPE fit-tests, and what are the consequent alternatives available	**26**	**2.85**	**1.19**	**2.76-2.94**
RQ11	Does safety, efficacy, pharmacokinetics and optimal dosing of different therapeutic interventions for COVID-19, and post-COVID conditions differ amongst intersex or gender-diverse individuals who may be taking supplementation or had gender-affirming surgery which can alter their hormonal profile and physiology	**26**	**2.81**	**1.10**	**2.73-2.89**
RQ13	What are the sex differences in COVID-19 vaccine outcomes in paediatric populations, and what are past lessons and experiences in paediatric vaccine studies that can be applied in dosing and formulation studies, and product planning	**25**	**2.80**	**1.04**	**2.72-2.88**
RQ3	How can precision medicine approaches (the study of biofactors and biomarkers, precision multimodal imaging, multiomics) be applied to understand the influence of sex and gender in COVID-19 therapeutics, vaccines and diagnostics outcomes	**25**	**2.80**	**0.87**	**2.73-2.87**
RQ24	Are there sex, gender, or other intersectional differences in how effective PPE is in preventing exposure and infection to COVID-19	**26**	**2.77**	**1.14**	**2.68-2.86**
RQ20	How can digital R&D measure gender sensitivity in the acceptance, uptake and effectiveness of COVID-19 DHI	**25**	**2.76**	**1.01**	**2.68-2.84**

RQ18	Are there a sex, age and race differences in COVID-19 antibody production that produces sex differences in testing	**24**	**2.75**	**0.90**	**2.68-2.82**
RQ10	Does safety, efficacy, pharmacokinetics, optimal dosing regime, and protective duration of the different COVID-19 vaccines differ amongst intersex or gender-diverse individuals who may be taking supplementation or had gender-affirming surgery which can alter their hormonal profile and physiology	**26**	**2.73**	**1.04**	**2.65-2.81**
RQ29	How can funders, and innovators be incentivized to use artificial intelligence approaches that incorporate sex, gender and other dimensions of intersectionality in the COVID-19 R&D and commercialisation pipeline	**24**	**2.71**	**0.86**	**2.64-2.78**
RQ19	How can DHI R&D, including for smartphone apps and wearables, consider sex, gender and intersectionality in their design, training data sets, output accuracy, etc	**26**	**2.65**	**1.16**	**2.57-2.74**
RQ16	Do sex, age and race influence COVID-19 testing accuracy (sensitivity and specificity) in polymerase chain reaction (PCR) and rapid antigen tests, including antibodies, reinfections, and specimens used	**26**	**2.50**	**1.03**	**2.42-2.58**
RQ17	Do COVID-19 diagnostic trials include sex differences in the standardisation and calibration process of testing equipment and devices	**24**	**2.38**	**1.06**	**2.29-2.46**

Gender & Health Hub

Supplementary Table C: Research Questions Prioritised by Urgency

RQ	Label	N	Mean	SD	95% CI
RQ4	Does safety, efficacy, optimal dosing regime and protective duration of the different COVID-19 vaccines differ by sex, age, race	**25**	**2.64**	**0.70**	**2.59-2.69**
RQ13	What are the sex differences in COVID-19 vaccine outcomes in paediatric populations, and what are past lessons and experiences in paediatric vaccine studies that can be applied in dosing and formulation studies, and product planning	**23**	**2.57**	**0.73**	**2.50-2.63**
RQ22	Are the current personal protective equipment (PPE) used for COVID-19 sex/gender-sensitive in design	**25**	**2.56**	**0.77**	**2.50-2.62**
RQ8	Does safety, efficacy, optimal dosing regime, and protective duration of the different COVID-19 vaccines differ in pregnant and lactating women, and their foetuses and infants/toddlers	**25**	**2.48**	**0.77**	**2.42-2.54**
RQ16	Do sex, age and race influence COVID-19 testing accuracy (sensitivity and specificity) in polymerase chain reaction (PCR) and rapid antigen tests, including antibodies, reinfections, and specimens used	**24**	**2.46**	**0.66**	**2.40-2.51**
RQ5	Does safety, efficacy and optimal dosing of different therapeutic interventions for COVID-19, and post-COVID conditions differ by sex, age, race	**24**	**2.46**	**0.78**	**2.39-2.52**
RQ9	Does safety, efficacy and optimal dosing regimens of different therapeutic interventions for COVID-19, and post-COVID conditions differ in pregnant and lactating women, and their foetuses and infants/toddlers	**25**	**2.44**	**0.82**	**2.38-2.50**
RQ23	Given COVID-19, what is the proportion of women, men and gender-diverse people, with intersecting needs (i.e. disabilities, cultural attire, etc) that fail PPE fit-tests, and what are the consequent alternatives available	**22**	**2.41**	**0.80**	**2.34-2.48**
RQ33	In what way are sex and gender related variables integrated into national and global vaccine safety surveillance systems	**24**	**2.38**	**0.82**	**2.31-2.44**
RQ7	Are there sex differences in the outcomes of other medical procedures, such as non-invasive ventilation (administration of ventilatory support without using an artificial airway), proning (lying on your stomach, face down), and intubation (insertion of an artificial tube into the trachea) or use of prophylactic anticoagulation in hospitalized COVID-19 patients	**24**	**2.38**	**0.71**	**2.32-2.43**
RQ24	Are there sex, gender, or other intersectional differences in how effective PPE is in preventing exposure and infection to COVID-19	**24**	**2.33**	**0.82**	**2.27-2.40**
RQ1	How do the different aspects of human biology (i.e genetics, immunology, biochemistry, physiology, microbiota) contribute to sex-differences in pharmacokinetics, safety and efficacy of COVID-19 therapeutics and vaccines	**28**	**2.32**	**0.77**	**2.27-2.38**
RQ25	What clinical trial study design and statistical analysis strategies efficiently and cost-effectively ensure quality sex and gender sensitive analysis and reporting for COVID-19 research	**25**	**2.32**	**0.63**	**2.27-2.37**
RQ37	How can scientific journals better promote the reporting of sex-differentiated analysis in research on COVID-19 therapeutics, diagnostics, and digital health interventions	**25**	**2.32**	**0.75**	**2.26-2.38**
RQ12	Does an individual's age, reproductive stage, natural cyclic hormonal variation, use of hormonal supplementation or contraceptives influence COVID-19 therapeutic and vaccine outcomes	**26**	**2.31**	**0.84**	**2.24-2.37**
RQ2	To what extent are the sex differences established in basic science research integrated into the early product development phase of potential therapeutic and prophylactic products for COVID-19	**26**	**2.31**	**0.79**	**2.25-2.37**

Gender & Health Hub

RQ14	Are there vaccine-associated non-specific effects that influence mortality and morbidity rates in children and conversely are there non-specific COVID-19 protection or risks from use of other vaccines (e.g. BCG)	**23**	**2.30**	**0.76**	**2.24-2.37**
RQ30	How are regulatory agencies considering the sex and gender related factors in vaccine outcomes in evidence assessments and policy decision-making for emergency use approval	**24**	**2.25**	**0.90**	**2.18-2.32**
RQ40	How can pregnant and lactating females be ethically, and safely included in phase 3 and 4 studies for COVID-19 R&D	**24**	**2.25**	**0.90**	**2.18-2.32**
RQ15	Are there any long-term impacts on menstrual cycles and future fertility from vaccinations in all sexes, including children and adolescents	**21**	**2.24**	**0.83**	**2.16-2.32**
RQ21	How can DHI algorithms used in the pandemic be built to correct for gender and race bias	**24**	**2.21**	**0.78**	**2.14-2.27**
RQ6	Do the use of different formulation strategies (i.e use of specific adjuvants, vectors and non-active pharmaceutical ingredients) contribute to any sex differences in safety, efficacy, dosing regimes and contraindications in therapeutics and vaccines	**24**	**2.21**	**0.83**	**2.14-2.28**
RQ18	Are there a sex, age and race differences in COVID-19 antibody production that produces sex differences in testing	**22**	**2.18**	**0.73**	**2.12-2.25**
RQ36	How can media facilitate a stronger consideration of sex and gender in the therapeutic, diagnostic, and digital R&D ecosystems	**23**	**2.17**	**0.78**	**2.11-2.24**
RQ17	Do COVID-19 diagnostic trials include sex differences in the standardisation and calibration process of testing equipment and devices	**21**	**2.14**	**0.85**	**2.06-2.22**
RQ27	How can research ethics committees play a more prominent role in ensuring sex and gender dimensions are well considered in research trials	**24**	**2.08**	**0.78**	**2.02-2.25**
RQ41	Does user-perceived value and acceptability of therapeutics and vaccines (i.e utility, willingness-to-pay and risk-benefit trade-offs in safety and efficacy profiles) differ between various groups of women, men and gender-diverse users	**24**	**2.08**	**0.83**	**2.02-2.25**
RQ39	What is the extent of the enrollment and participation of women in ongoing and completed COVID-19 clinical trials across various sites and countries	**25**	**2.08**	**0.76**	**2.02-2.24**
RQ10	Does safety, efficacy, pharmacokinetics, optimal dosing regime, and protective duration of the different COVID-19 vaccines differ amongst intersex or gender-diverse individuals who may be taking supplementation or had gender-affirming surgery which can alter their hormonal profile and physiology	**25**	**2.04**	**0.73**	**1.98-2.10**
RQ26	What are the necessary changes required in data management systems, clinical trial SOPs, and workforce training, to support the collection of gender identity and biological sex at birth in clinical trial participant registration	**25**	**2.04**	**0.84**	**1.97-2.11**
RQ35	What user-friendly, interactive tools can enable the general public to better understand the role that sex and gender play in COVID-19 R&D	**25**	**2.04**	**0.89**	**1.97-2.11**
RQ11	Does safety, efficacy, pharmacokinetics and optimal dosing of different therapeutic interventions for COVID-19, and post-COVID conditions differ amongst intersex or gender-diverse individuals who may be taking supplementation or had gender-affirming surgery which can alter their hormonal profile and physiology	**26**	**2.00**	**0.75**	**1.94-2.06**
RQ32	How can international standards and templates for regulatory and evaluation dossiers, including for emergency use vaccine and therapeutic products during pandemics, be adapted to incorporate sex and gender considerations in the evaluation of therapeutic safety and effectiveness, and product registration process	**25**	**1.96**	**0.84**	**1.89-2.03**

RQ34	How can funders, industry and regulators enhance gender representation and inclusiveness in decision making throughout all stages of the COVID-19 R&D pipeline	**24**	**1.96**	**0.81**	**1.89-2.02**
RQ38	How can regulatory bodies implement legal requirements for age, sex, gender, and race data in trial information to be made available to the public	**24**	**1.96**	**0.81**	**1.89-2.02**
RQ20	How can digital R&D measure gender sensitivity in the acceptance, uptake and effectiveness of COVID-19 DHI	**23**	**1.96**	**0.77**	**1.89-2.02**
RQ28	Do public research funding, and private product planning and commercialisation strategies of COVID-19 interventions have a gender bias	**22**	**1.95**	**0.84**	**1.88-2.03**
RQ31	What are effective regulatory strategies for ensuring the design and reporting of COVID-19 research that considers sex and gender by scientist innovators, and commercial funders	**23**	**1.87**	**0.87**	**1.80-1.94**
RQ3	How can precision medicine approaches (the study of biofactors and biomarkers, precision multimodal imaging, multiomics) be applied to understand the influence of sex and gender in COVID-19 therapeutics, vaccines and diagnostics outcomes	**25**	**1.84**	**0.80**	**1.78-1.90**
RQ19	How can DHI R&D, including for smartphone apps and wearables, consider sex, gender and intersectionality in their design, training data sets, output accuracy, etc	**22**	**1.73**	**0.70**	**1.66-1.79**
RQ29	How can funders, and innovators be incentivized to use artificial intelligence approaches that incorporate sex, gender and other dimensions of intersectionality in the COVID-19 R&D and commercialisation pipeline	**22**	**1.59**	**0.73**	**1.53-1.66**
