# Supplementary material for: A shared agenda for gender and COVID-19 research: priorities based on broadening engagement in science
Source: BMJ Glob Health. 2023 May 22;8(5):e011315. doi: 10.1136/bmjgh-2022-011315 (PMC10230361; doi:10.1136/bmjgh-2022-011315)
Supplement: Supplementary data [file bmjgh-2022-011315supp005.pdf]

Gender & COVID-19 Research Agenda-Setting

Draft Thematic Report

Thematic Group 3: Health Service Delivery

How gender influences the inputs for, quality of and utilisation of health service delivery for covid-19 and non-covid19 health conditions

Contents

List of Abbreviations 2
Section 1: Introduction to the overall collaboration 3
Section 2. Thematic group participation and engagement 4
Section 3. Thematic group background. 5
3.1 Definitions and scope 5
3.2 Current understanding, challenges, gaps and neglected areas (outline of key themes, to be finalised at the end) 6
3.2.1 Health service delivery, access, and innovation 6
3.2.2. Human resources for health 8
3.2.3 Medical supplies, products and technologies 9
3.2.4 Health information 9
3.2.5 Health Financing 10
3.3 Desired impact of the proposed research on policy, program, and community responses 10
3.4 Actors and strategies to implement and promote uptake of the research agenda (to be completed at end) 11
3.5 Population, contexts, study design/ methodologies (to be written at the end) 11
Section 4. Research questions proposed for prioritisation. 12
Section 5. References 15
Section 6. Resources shared 17
Section 7: Tables with Results of Prioritised Questions

List of Abbreviations

CBO – Community Based Organization
FP – Family planning
NGO – Non-Governmental Organization
NCDs – Non-Communicable Diseases
PPE – Personal Protective Equipment
SRHR – Sexual and Reproductive Health and Rights
SGBV – Sexual and Gender Based Violence
VAW – Violence Against Women
LGBTQIA+ - lesbian, gay, bisexual, transgender, queer, intersex, and asexual

Section 1: Introduction to the overall collaboration

From the start of the COVID-19 pandemic, how it has affected and continues to affect women, girls, men, boys, and non-binary gender diverse groups is complex and evolving. Apart from the direct effects of COVID-19 illness, pandemic responses have also amplified existing gender inequalities across multiple dimensions. Context and the intersecting influence of other social determinants or identities[1], [2] also worsened the influence of gender inequalities during the pandemic, with combined effects on health and related areas.

Early high-level calls and advocacy from researchers[3] such as through the Gender and COVID-19 working group[4] were made for gender considerations to be integrated into the crisis response. Nevertheless, real-time response to the gender dynamics was limited by extensive invisibility of the evolving situation, incomplete data systems and lingering evidence gaps. As the world steps into the second year of the COVID-19 crises, given the gender dynamics involved, we must include gender in the investments being made in research that informs both the immediate response and action and long-term recovery from the health and socio-economic consequences of the pandemic.

The United Nations University International Institute for Global Health is co-convening a collaborative gender and COVID-19 research agenda-setting exercise, as part of its Gender and Health Hub's inaugural scope of work. The process is co-developed through real-time learning, and open calls to a broad range of stakeholders to comment and contribute to its design, scope and content. Collective contributions and questions for prioritization are supported by a community discussion board (www.ghhbuzzboard.org). Please visit this discussion board for further information.

The output of the exercise will be a shared research agenda that can be utilized by researchers, funders, and policymakers to guide COVID-19 research investments and corresponding programming and policy actions by the health sector.

The draft thematic group reports emerging from this collective endeavour are a synthesized version of the contributions made to the discussion board combined with additional inputs from thematic group coordinators, co-leads and steering committee members. They document participation and engagement to date, provide a background section outlining definitions, scope, gaps, impact and audiences, before listing research questions for prioritisation.

Section 2. Thematic group participation and engagement

Thematic group coordinator: Sedighe Hosseinijebeli (Iran)
Thematic group co-leads: Rosemary Morgan (USA), Prabha Thangaraj (India)
Thematic group steering committee focal point: Mamothena Carol Mothupi (Kenya)/ Asha George (South Africa)

Thematic group contributors in alphabetical order:

1. Adanaa Chukwuma
2. Anjana Bhushan
3. Anthony Keedi (workshop)
4. Anuj Kapilashrami (google doc)
5. Avni Amin
6. Awatef Amer Nordin
7. Bernadette Ateghang-Awankem
8. Claire Standley (workshop)
9. Edwine Barasa (google doc)
10. Emilia Antonio
11. Ephram Senkyire
12. Fui Ching Lum
13. Gustavo Nigenda/ Projecto Enfermeria (from google doc)
14. Heather Doyle
15. Jashodara Das Gupta (workshop)
16. Karen Lawford (from google doc)
17. Kathryn H.Jacobsen
18. Kavita Bhatia (from google doc)
19. Kui Muraya (from google doc)
20. Lavanya Vijalakshmi (w)
21. Linget Chepuka
22. Mariam Otmani del Barrio
23. Maryam Rumaney
24. Michelle McIsaac (from google doc)
25. Ranjini CR (from google doc)
26. Ranjini Raghavendra
27. Sarah Nurain Mohd Noh
28. Sophie Witter (from google doc)
29. Stephen Mulupi
30. Teresa Farinha
31. Veloshnee Govender (from google doc)
32. Vidyadhar Bangal
33. Wincent Wagner

First draft report posted on discussion board on 30/01/2021
Second draft report posted on discussion board on date 02/02/2021
Third draft report posted on discussion board on date 25/02/2021

Shared for expert consultation on date (ADD)
Workshop for internal and expert review on 15/04/2021
Fourth draft report posted on discussion board on date 12/05/2021

Section 3. Thematic group background.

3.1 Definitions and scope

Gender is regarded as the socially constructed roles, behaviours, attitudes and values of men, women and other individuals who fall outside of these binary categories. In the context of the covid-19, gender plays a role in how individuals experience the pandemic and the public health measures enacted to control it, as well as health outcomes and access to health services. The focus of this report is to explore the gendered aspects of health service delivery within the context of the pandemic. This entails the gendered experiences of health workers and health system clients at the intrapersonal, interpersonal, household and health system levels. Issues of health governance are discussed under the Thematic Group 5 (TG5), behavioural factors and health outcomes under TG1, research and development under TG2, and social and structural determinants of health under TG4.

To assess health service delivery aspects of the pandemic, we frame the role of gender in terms of the power dynamics between men and women and their influence on access to resources, decision making, values and social norms, and division of labour [5]. This framing helps us to assess access to services and the relevant health system inputs such as financing, medical supplies, commodities and technologies, and the health workforce, among others. The role of gender in decision-making processes within households and health systems, as part of the pandemic response, is also explored (although governance in particular is discussed in the separate TG5 report). It is also important to assess how social values and norms are exercised during the pandemic and their direct impact on access to care and quality of care. And lastly, gender norms have influenced how men and women divide duties in households as caregivers and in the health system as health workers, potentially creating different trajectories of the pandemic experience and health impact.

Alongside gender, the pandemic has affected individuals and populations differently along the present stratifiers of class, race/ethnicity, geographical area, disability, sexuality, immigration status, etc. These stratifiers are created by the power dynamics and structures present in our societies [5], [6] and are the basis on which inequalities entrenched by the pandemic can be assessed. Thus, before the pandemic there were already inequalities and inequities within the health system which can be assessed along these intersectional lines. For analysis of the pandemic response, this means re-acknowledging the multifaceted nature of oppression and marginalization and assessing how health systems can both mitigate and perpetuate them [6], [7]. This can form the basis of a transformative agenda for health systems, which aims to address current inequities and improve readiness and resilience for future pandemics.

We recognize the important role of context in shaping health service access and delivery in various populations, and that a global agenda needs to be aware of these nuances. Macro-contextual factors such as the politics, economies, culture, and legislative frameworks all shape the availability, quality, and responsiveness of health services during the pandemic. And although this

report does not discuss these drivers in depth, the research agenda for health service delivery can be informed by them. This can lead to research priorities that reflect and are adaptable to nuances such as global north/ global south, conflict and crisis affected areas, humanitarian settings, etc. In the context of authoritarianism and discriminative legislative frameworks in various countries, we also adopt a human rights lens to the obligation of the health to deliver accessible, acceptable and equitably distributed services to populations[8].

The specific aspects of health service delivery that are explored in this report includes equitable access and quality of care, innovative service delivery models in response to the pandemic, health worker experiences, issues in medical supplies, technologies and commodities, and health information and health financing issues. While responding to covid-19 cases is important, the health systems also needs to ensure continuity of care for non-Covid 19 health conditions, such as maternal and sexual and reproductive health, HIV and other chronic conditions, and harm reduction services. And while the report does not specify separate roles for the private and public providers, the same gender issues apply to each sector and the role of public-private partnerships is relevant as well.

3.2 Current understanding, challenges, gaps and neglected areas (outline of key themes, to be finalised at the end)

3.2.1 Health service delivery, access, and innovation

a) ***Gender equitable access and quality of health services*****:** Gendered inequities in access and quality of care existed pre-pandemic and the research agenda for covid-19 can characterize them and assess how they changed during the pandemic, as well as highlight the effective strategies to address them.

The COVID-19 pandemic directly impacted health services due to restrictions in mobility, closure of facilities, fear of infection, lack of information, lack of affordability of services due to job losses, reorganization of services to provide covid-19 care, among other factors[9], [10]. These enhance barriers to access and lead to prioritization of covid-19 care at the expense of continuity of essential health services. For instance, research suggests that the pandemic potentially affected the availability and access to services that women use, such as sexual and reproductive health, antenatal and maternity services in low- and middle-income countries[3], [11], [12]. This threatens to reverse gains made in preventing negative outcomes related to unintended pregnancies, unsafe abortions, transmission of sexually transmitted illnesses, and maternal and infant mortality among others[10], [11]. It thus entrenches poor service delivery and gender inequities contributed by the health system and weakens pre-pandemic system strengthening efforts. Innovative strategies that have been recommended to relieve burden on the health system and prevent exposure to covid-19 include community based interventions and telemedicine[13].

The pandemic is expected to entrench harmful practices such as gender based violence, violence against women, and rape, emphasizing the need for the health system to ensure availability of services for survivors [10], [11]. Norms such as toxic masculinity can also play a role in men's care seeking behaviour and access to services, necessitating a response

that expands health promotion/health messaging tailored for men and service expansion for hard to-reach men[16]. A health system response that involves men may also help mitigate violence against women and women's care seeking for SRHR. Poverty, informal dwelling, drug and alcohol abuse, ethnicity, infection status, and access to WASH services are among other factors that can increase the vulnerability of people to infection and limit access to services [11], [14]. For drug and alcohol abusers, for instance, harm reduction services and treatment continuity plans are needed to enable isolation, clinical care and clinical care in an integrated manner [17], [18].

Ensuring the continuity of essential non-covid 19 care, managing surge capacity, and health service integration emerge as some of the most important service delivery indicators of resilient health systems in the face of the current and previous pandemics[19]–[22]. While other factors such as financial protection, cross-sectoral collaboration, and well-functioning surveillance and information systems are also stated, it is not clear the gender equity and human rights dimensions that may influence the pandemic experience for different groups. The Singapore experience highlighted the importance of an inclusive response that protects all individuals to effectively control the outbreak[20]. Studies of pandemic resilience do not sufficiently explore equity dimensions as they are often supply oriented, and future research can address both supply and demand issues tied to resilience and equity, particularly gender equity.

There is more research needed to describe the nature and extent of the gender inequities in access to COVID-19 and non-COVID-19 related care. Further-more, the interaction of gender with other demographic and social stratifiers (such as age, ethnicity, geography, sexuality, socioeconomic status, immigration status, (dis)ability among others) in determining access to and quality of care need to be researched further, to support health system reform efforts to improve services in an equitable manner. It is crucial to maintain availability, acceptability and quality of essential services, especially those that impact on gender and rights dimensions of health care. These include among others, substance abuse, gender based violence, mental health services; sexual and reproductive health services, maternal and child health, as well as injuries, services for people living with disabilities and non-communicable diseases (NCDs) and adolescent health, among others.

b) Innovative health service delivery models: Due to the impact of the pandemic on access and delivery of services, innovative models are necessary to ensure that services reach populations affected. In addition, innovative models need to ensure that gender inequities within the health system are mitigated and not worsened.

The pandemic has highlighted the need to invest in and integrate community-based service delivery platforms (including partnerships with NGOs, CBOs) that are at the forefront of ensuring expanded access to services. An example is a tiered primary care approach encompassing community based screening and treatment (mild symptoms) centres to relieve pressure from primary facilities and hospitals in Taiwan[23]. There are also recommendations for management of sexual and reproductive health services such as antenatal care by community health workers, home births where appropriate midwifery

staff and resources are available, midwifery led units, and guidelines for self-managed use of contraceptives among others[13].

Another type of innovation includes the use of telemedicine and other digital health technologies (including mobile) to expand access to health services including mental health[24], [25], pharmacy [26], dental [27], e-ICU consultations [28], monitoring and follow-up of covid-19 cases[29], SRHR such as antenatal and postnatal care [13], and physiotherapy among others[30]. Gender and other inequities in access and literacy to use digital health innovations existed prior to the pandemic and should be taken into account[31]. There are also system and policy requirements of telemedicine and telehealth that need to be addressed before this innovation is fully embedded in the health system and access expanded[24]. Other strategies to expand care coverage should be explored and their effectiveness studied, especially in resource constrained contexts. For example, in mental health, these include task-shifting to trained lay health workers to provide mental health services LMICs [25]. Like digital technologies, deployment of lay health workers will have gendered implications (given that frontline and community health workers are often women) and thus an equity should be embedded in these innovative strategies. And there is additional research to be done in the implementation of telehealth approaches and patient and provider satisfaction[32].

Other innovative approaches take a multisectoral perspective, such as improving the accessibility of essential services, particularly health, to improve the health and quality of life of the elderly[33]. There is also a need for vertically oriented health systems to be more integrated and broad based. For example, on the African continent, a model was proposed that involves supporting the mixed health systems of public and private health providers, and community based health structures including traditional birth attendants and accredited medicine vendors to maintain services in a people centred approach[34]. This would be a move away from a national, highly strategic and public hospital centered approach that may inadequate in operationalizing all the goals of pandemic control. Again the remaining gap in these literature is the role of equity, particularly gender equity, and how it can be improved and sustained during the pandemic.

3.2.2. Human resources for health

COVID-19 highlighted the role of women from marginalised groups who are at the forefront of responding to the pandemic both at home and in health and social services. Inequitable/ one way decision making, a gendered division of labour, unequal training and supervision, and lack of supportive structures affected community health workers and nurses, most of whom are women, negatively during the pandemic. During the pandemic, increased workload, restrictions on movement, stress, burnout, absenteeism, and violence, stigma and harassment combined with infection and death all impacted health workers. How these COVID-19 pressures combined with the gendered nature of human resources for health (where women are disproportionately represented in patient facing roles but not in the decision making platforms of their health professions, where they have less access to training, protective equipment and other safety measures, have less professional autonomy, and are systematically underpaid if paid at all) needs

to be explored,. While the gendered effects of COVID-19 on the general labour force are covered by TG4, those involving health carers and workers are considered in this section.

Health workers experienced challenges in providing care to patients, based on workplace and household dynamics that are influenced by gender. The influence of family and support roles during the pandemic, for instance, is expected to be observed on health workforce productivity along gender lines. Gender, sex and other intersecting influences also have an effect on workplace experiences during the pandemic, including coping mechanisms, benefits and workloads, and access to resources such as medical supplies and products (e.g. PPEs and vaccines), and transport, among others. Research into how to address these negative impacts of the pandemic on the health workforce is critical to respect the rights of health workers (particularly their voice, safety and remuneration) and to ensure that they do not amplify inequities in service delivery to different gender and marginalized groups.

3.2.3 Medical supplies, products and technologies

Access, utilization and distribution of medical supplies, products and technologies has been impacted by the pandemic. These include medicine shortages or delays, including in relation to contraceptive care, repair and procurement of equipment especially in critical care, vaccine distribution, personal protective equipment for health workers, and use of information technology for health information, among others. Before the pandemic, gender dynamics already influenced access to and regulation and utilization of quality medical supplies, products and technologies[5]. During pandemic also, gender-related barriers and inequities in communities and health systems negatively affect access to health services, including vaccination. The consequences of neglecting these barriers in COVID-19 vaccine deployment include: insufficient or fragmented vaccination delivery hindering the achievement of population-level immunity, increased burden of preventable morbidity and premature mortality from COVID-19 and other conditions, delayed economic recovery, widened gender inequities and inequalities and violations of rights in society. To avoid these consequences, it is necessary to identify and act on the known and context-specific barriers that are influenced by gender inequalities to achieve the highest level of population coverage possible. Prioritize targeted outreach to vulnerable and disadvantaged groups within each priority population eligible for vaccination, and address the additional gender-related barriers they face and partner with women's organizations and other community-based groups to ensure accurate information is available to communities, and gender perspectives are considered in planning, design, and monitoring.

The group raises research questions related to how these issues persisted or changed, including worsening, during the pandemic, and how they impacted health workers and clients along gender lines. In addition, the group is interested in the gendered aspects of design of health technologies. This includes PPE's, which research has shown their inadequacies for female bodies, with impact on how health workers conduct their work.

3.2.4 Health information

Usage of gender sensitive health indicators through gender-affirming data acquisition, mining and interpretation has an impact on ensuring all genders represented in creating key policy change. Gender inclusive data can further develop deeper understanding of social dynamics that may have exacerbated gender inequalities as a result of COVID-19. Data that reflects sex/gender differences and intersections with other factors in access and quality of care is however not used routinely at all levels of the health system. This affects the responsiveness of the health system to populations at the intersections of age, ethnicity, gender identity, sexuality, disability, geographic location, immigration status, among others. The design and strengthening of services during the pandemic relies on timely information that is suitably disaggregated to capture nuances in inequities and improve outcomes (alongside the capacity to analyse and use the data to improve services for underserved groups). The gendered aspects of contact tracing and other data collection related to the pandemic are also important to consider. These include confidentiality and human rights issues in relation to Covid 19 and vaccination status. Other aspects of health information include gendered access to information by populations, such as women and girls when they need it, as well as tailored communication that allows feedback and interaction/two way communication instead of paternalistic messaging from authorities. In addition the implications of data gathering and monitoring for marginalized groups, as well as digital exclusion that disproportionately burden the latter and women should be considered.

3.2.5 Health Financing

This theme explores Covid 19 mitigation measures that have gendered impact on health financing functions (revenue raising, pooling, and purchasing and financial risk protection). For instance, how countries choose to finance covid-19 services may influence access to services in gendered ways. Health financing functions may also be adapted to respond to COVID-19 in ways that have gendered impacts e.g. resources may be reallocated from other services to COVID-19 (with the reallocation affecting gendered services - FP, SRH etc.). The research agenda focuses on studying the health financing effects on gender inequities and measures for protection of vulnerable groups affected by COVID-19. These include financial protection programs, health safety net schemes and reduced out of pocket payments through suspension of co-payments or user fees. At the interface between the health system and patients, the gender and intersectional factors related to ability to pay, and their influence on responsiveness and quality of care, especially for treatment of Covid-19 related conditions are also considered. Finally, the research agenda also should review the gendered dimensions of UHC and how these have been affected by COVID-19. In several countries, health insurance premiums are higher for women than for men; has this changed given COVID-19 health reforms?

3.3 Desired impact of the proposed research on policy, program, and community responses

- **Equitable distribution of services:** The research should address equity, responsiveness and quality of care to ensure that services are available and appropriate for the needs of different genders, with consideration of other stratifying factors. This includes equitable distribution of essential medications, vaccinations and other supplies being developed in response to the pandemic.
- **Strengthened organization and implementation of services:** It is expected that the research would impact on health service delivery in terms of gendered organizational bottlenecks and implementation challenges. In addition, the evidence should demonstrate how to ensure that

essential services are not overlooked during the pandemic, thereby worsening the gendered impact of other diseases and conditions.

- **Human resources for health**: (under construction)
- **Medical supplies, products and technologies:** (under construction)
- **Health information:** (under construction)
- **Health financing:** The research should support more gender equitable health financing incidence during and post-pandemic; reduction of financial barriers for vulnerable groups; reduced catastrophic payment and impoverishment; and more efficient purchasing of gender-sensitive packages of care, among others.

3.4 Actors and strategies to implement and promote uptake of the research agenda (to be completed at end)

Alliance for Health Policy and Systems Research
Health Financing and Governance/ WHO
Health Workforce/ WHO
Gender Equity Hub WHO
UHC2030
GFF/ World Bank
Department of Sexual and Reproductive Health and Research/WHO
ReBUILD for Resilience
Bilateral bodies
Global Fund RSSH grants
GAVI HSS

3.5 Population, contexts, study design/ methodologies (to be written at the end)

Section 4. Research questions proposed for prioritisation.

Based on the comments from participants, the following initial questions are proposed in five main categories, including health service delivery access and innovation, human resources for health, medical supplies, products and technologies, health information, and health financing.

Access and quality of care during the covid 19 pandemic (general)

1. How does access and quality of care for both covid and non-covid 19 services differ across different populations based on gender and other intersecting factors?
2. What is the nature and effectiveness of strategies that were used to improve gender and other inequities in access and quality of care for covid-19 services?
3. How the lack of functioning of other industries like transport does affect the ability of health workers to deliver services during the pandemic and were there gender implications to this?
4. How has the prioritization of covid-19 services and the reorganization of existing service delivery models affected the access to services for non-covid 19 health conditions?

Access and quality of care during the covid 19 pandemic (key health services)

5. To what extent has the demand and utilization of maternal, sexual and reproductive health (SRHR) and violence against women services been changed during the COVID-19 pandemic?
6. What was the extent of prioritization and the effectiveness of different service reorganization models to ensure continuity of maternal health, sexual and reproductive health (SRHR) and violence against women (VAW) services during the pandemic?
7. Were there gender differences in access to harm reduction services for marginalized populations, including people with substance abuse problems, homeless people, and sex workers during the pandemic?
8. How have service delivery measures responded to the needs of pregnant women who have just recovered from COVID-19 or have been tested positive and are quarantined?
9. What challenges has the pandemic posed to the survivor centred approach embedded into SGBV holistic services (health, legal, psycho-social)?
10. What measures are service providers and policymakers enacting to mitigate and safeguard against future service interruptions in several key services including sexual and gender based violence centres, maternity centers and blood banks?

Access and quality of care during the covid 19 pandemic (key populations)

11. How did the pandemic and the health system response influence the availability and utilization of first line services for vulnerable groups (including people with chronic diseases and the elderly, survivors of gender based violence , LGBTQIIA+, migrants, refugee women and institutionalized populations)?
12. How have administrations managed to facilitate care targeted to the elderly affected by COVID-19 and ensured responsiveness to gender and other intersectional factors?

13. What intersectional factors including countries, socioeconomic status, sex, religion, or urban vs rural areas influenced the experiences of LGBTQIIA+ communities in accessing care during the pandemic?
14. What are the experiences of organizations that provide health care services to LGBTQIIA+ people in countries that do not recognize their rights?
15. How did gender diverse people experience services for prevention and control of the pandemic, such as quarantine in male/female wards, and vaccination eligibility based on binary categories?

Innovative Service Delivery Models

16. What innovative service models exist to address gender barriers in accessing services during the pandemic?
17. Which effective service delivery models can support home based care and address gender biases of uneven distribution of labour, which creates a caregiving burden for women?
18. What are the ways in which gender biases in accessing tele-medicine for COVID-19 can be offset to ensure its effectiveness of this service delivery model?
19. Which community-based service delivery models best address gender barriers in access to health services during the COVID-19 pandemic?
20. How did the private and public sector compare in innovation and gender responsiveness of services during the pandemic?

Human Resources for Health

21. What were the gender dynamics of access to protective equipment by health workers throughout the pandemic and how were they addressed by the health system?
22. To what extent are there gendered inequities in access to training opportunities for health workers delivering COVID-19 services at different levels of the health system and across different occupations and how best can they be addressed?
23. Is there a gendered difference in effectively accessing and using e-learning technologies among health workers, given that COVID-19 has increased reliance on digital modes of training?
24. What measures (including safety and security) need to be put in place to ensure protection of health service providers who are mostly women and may be travelling to remote areas to distribute the vaccine?
25. Did measures taken for surge capacity, including recruitment reforms, have different impacts on health workers based on gender and occupation?
26. To what extent did gender norms at different levels including household and health system influence health worker availability and productivity during the pandemic?
27. What are the gendered human resources aspects of delivering care (in terms of staff gender distribution, division of labour, experiences of care workers) in exclusive covid-19 facilities?
28. What measures (including mental health, remuneration, accommodation and transport) effectively improved the gender responsiveness of the health system in supporting health workers during the pandemic, particularly the disproportionate amount of women at the interface with communities and patients?

Medical Supplies, Products and Technologies

29. What are the health system challenges and effective measures employed during vaccine roll out to improve access by vulnerable, marginalized and hard to reach groups?
30. How have service delivery arrangements changed in response to the roll out of vaccines and how has this affected gender equity in access to medicines?

Health Information

31. Are the differences observed in the data for hospitalization and death rates for males and females influenced by gender differences in reporting of deaths and access/affordability of care?
32. What improvements should be made to health information systems in terms of reporting sex-disaggregated data and other intersectional indicators to improve services during and beyond the pandemic?

Health Financing

Revenue Raising

33. How are COVID-19 individual services paid for/financed, and how does it affect access to services and financial protection along gender dimensions?
34. What is the share of public/private expenditures in financing the continuum of COVID-19 care (from screening to intensive care units) and how does it compare along gender dimensions?
35. What are the implications of re-allocation or re-prioritization of funding for access to health care across different gender groups during the pandemic?
36. How are funds in charities and volunteer bodies allocated across different gender and vulnerable groups in order to improve access and quality of better health services?

Purchasing

37. To what extent did purchasing mechanisms (e.g. benefit packages, provider selection and payments, contracting etc.) change to respond to the pandemic, and how did these changes affect health care access and quality across different gender groups?
38. To what extent did public financial management mechanisms for budget formulation, execution, and monitoring facilitate gender responsiveness in addressing service delivery and access challenges?
39. To what extent could purchasing services from private sectors through public resources help gender equality in access to care during and beyond the pandemic?

Pooling and protective measures

40. What are the possible immediate mechanisms (including wider social protection schemes) to protect woman-headed households against health expenditures (and the wider economic shock effects) during the pandemic?

41. In the long run, how can women and vulnerable populations be protected against catastrophic healthcare costs due to COVID-19, especially in countries where gender equitable UHC systems are not well-developed yet?
42. How can cross-subsidy between male and female payers be encouraged in favour of vulnerable groups such as women workers, within different pooled insurance funds during pandemic?

Section 5. References

[1] M. Lokot and Y. Avakyan, "Intersectionality as a lens to the COVID-19 pandemic: implications for sexual and reproductive health in development and humanitarian contexts," *Sexual and Reproductive Health Matters*, vol. 28, no. 1. Taylor and Francis Ltd., Jan. 01, 2020, doi: 10.1080/26410397.2020.1764748.

[2] O. Hankivsky and A. Kapilashrami, "Policy Brief | What is Intersectionality? Beyond sex and gender analysis: an intersectional view of the COVID-19 pandemic outbreak and response."

[3] C. Wenham, J. Smith, and R. Morgan, "COVID-19: the gendered impacts of the outbreak," *Lancet*, vol. 395, no. 10227, pp. 846–848, 2020, doi: 10.1016/S0140-6736(20)30526-2.

[4] Gender Working Group, "Gender Working Group > Gender & Covid-19," 2020. https://www.genderandcovid-19.org/gender-working-group-page/ (accessed May 12, 2021).

[5] R. Morgan, A. George, S. Ssali, K. Hawkins, S. Molyneux, and S. Theobald, "How to do (or not to do)... gender analysis in health systems research," *Health Policy Plan.*, vol. 31, no. 8, pp. 1069–1078, 2016, doi: 10.1093/heapol/czw037.

[6] R. Young, R. M. Ayiasi, M. Shung-King, and R. Morgan, "Health systems of oppression: applying intersectionality in health systems to expose hidden inequities," *Health Policy Plan.*, vol. 35, no. 9, pp. 1228–1230, 2020, doi: 10.1093/heapol/czaa111.

[7] K. Hay, M. G. Foundation, V. Percival, S. Henry, J. Raven, and R. Morgan, "Disrupting gender norms in health systems: making the case for change," *Lancet*, vol. 393, no. 10190, pp. 2535–2549, 2020, doi: 10.1016/S0140-6736(19)30648-8.Disrupting.

[8] C. Vlassoff and R. St. John, "A human rights-based framework to assess gender equality in health systems: the example of Zika virus in the Americas," *Glob. Health Action*, vol. 11, no. sup3, 2018, doi: 10.1080/16549716.2019.1570645.

[9] World Health Organization (WHO), "Maintaining essential health services: operational guidance for the COVID-19 context interim guidance." https://www.who.int/publications/i/item/WHO-2019-nCoV-essential-health-services-2020.1 (accessed May 06, 2021).

[10] UNFPA, "COVID-19: A Gender Lens PROTECTING SEXUAL AND REPRODUCTIVE HEALTH AND RIGHTS, AND PROMOTING GENDER EQUALITY," 2020.

[11] T. Riley, E. Sully, Z. Ahmed, and A. Biddlecom, "Estimates of the Potential Impact of the COVID-19 Pandemic on Sexual and Reproductive Health In Low- and Middle-Income Countries," *Perspect. Sex. Reprod. Health*, vol. 46, pp. 73–76, 2020, doi: 10.1363/46e9020.

[12] C. Wenham, J. Smith, and R. Morgan, "Covid-19 is an opportunity for gender equality within the workplace and at home," doi: 10.1136/bmj.m1546.

[13] L. B. Tolu, G. T. Feyissa, and W. G. Jeldu, "Guidelines and best practice recommendations on reproductive health services provision amid COVID-19 pandemic: scoping review," *BMC Public Health*, vol. 21, no. 1, pp. 1–10, Dec. 2021, doi: 10.1186/s12889-021-10346-2.

[14] World Health Organization, "Gender and Covid-19: Advocacy Brief," 2020. https://apps.who.int/iris/bitstream/handle/10665/332080/WHO-2019-nCoV-Advocacy_brief-Gender-2020.1-eng.pdf (accessed May 06, 2021).

[15] J. Connor *et al.*, "Health risks and outcomes that disproportionately affect women during the Covid-19 pandemic: A review," *Social Science and Medicine*, vol. 266. Elsevier Ltd, p. 113364, Dec. 01, 2020, doi: 10.1016/j.socscimed.2020.113364.

[16] M. Betron, A. Gottert, J. Pulerwitz, D. Shattuck, and N. Stevanovic-Fenn, "Men and COVID-19: Adding a gender lens," *Glob. Public Health*, vol. 15, no. 7, pp. 1090–1092, 2020, doi: 10.1080/17441692.2020.1769702.

[17] M. Karamouzian, C. Johnson, and T. Kerr, "Public health messaging and harm reduction in the time of COVID-19," *The Lancet Psychiatry*, vol. 7, no. 5. Elsevier Ltd, pp. 390–391, May 01, 2020, doi: 10.1016/S2215-0366(20)30144-9.

[18] S. D. Kimmel, A. R. Bazzi, and J. A. Barocas, "Integrating harm reduction and clinical care: Lessons from Covid-19 respite and recuperation facilities," *J. Subst. Abuse Treat.*, vol. 118, p. 108103, Nov. 2020, doi: 10.1016/j.jsat.2020.108103.

[19] H. Legido-Quigley *et al.*, "Are high-performing health systems resilient against the COVID-19 epidemic?," *The Lancet*, vol. 395, no. 10227. Lancet Publishing Group, pp. 848–850, Mar. 14, 2020, doi: 10.1016/S0140-6736(20)30551-1.

[20] A. Q. Chua *et al.*, "Health system resilience in managing the COVID-19 pandemic: lessons from Singapore," *BMJ Glob. Heal.*, vol. 5, no. 9, p. e003317, Sep. 2020, doi: 10.1136/bmjgh-2020-003317.

[21] M.-P. Kieny, D. B. Evans, G. Schmets, and S. Kadandale, "Health-system resilience: reflections on the Ebola crisis in western Africa," *Bull. World Health Organ.*, vol. 92, no. 12, pp. 850–850, Dec. 2014, doi: 10.2471/BLT.14.149278.

[22] H. Legido-Quigley, J. T. Mateos-García, V. R. Campos, M. Gea-Sánchez, C. Muntaner, and M. McKee, "The resilience of the Spanish health system against the COVID-19 pandemic," *The Lancet Public Health*, vol. 5, no. 5. Elsevier Ltd, pp. e251–e252, May 01, 2020, doi: 10.1016/S2468-2667(20)30060-8.

[23] B. B. J. Chang and T. Y. Chiu, "Ready for a long fight against the COVID-19 outbreak: An innovative model of tiered primary health care in Taiwan," *BJGP Open*, vol. 4, no. 2, Jun. 2020, doi: 10.3399/BJGPOPEN20X101068.

[24] C. B. Taylor, E. E. Fitzsimmons-Craft, and A. K. Graham, "Digital technology can revolutionize mental health services delivery: The <scp>COVID</scp> -19 crisis as a catalyst for change," *Int. J. Eat. Disord.*, vol. 53, no. 7, pp. 1155–1157, Jul. 2020, doi: 10.1002/eat.23300.

[25] L. Kola, "Global mental health and COVID-19," *The Lancet Psychiatry*, vol. 7, no. 8. Elsevier Ltd, pp. 655–657, Aug. 01, 2020, doi: 10.1016/S2215-0366(20)30235-2.

[26] H. Li, S. Zheng, F. Liu, W. Liu, and R. Zhao, "Fighting against COVID-19: Innovative strategies for clinical pharmacists," *Research in Social and Administrative Pharmacy*, vol. 17, no. 1. Elsevier Inc., pp. 1813–1818, Jan. 01, 2021, doi:

10.1016/j.sapharm.2020.04.003.
[27] Y. Yang, Y. Zhou, X. Liu, and J. Tan, "Health services provision of 48 public tertiary dental hospitals during the COVID-19 epidemic in China," *Clin. Oral Investig.*, vol. 24, no. 5, pp. 1861–1864, May 2020, doi: 10.1007/s00784-020-03267-8.
[28] D. Kumaraiah, N. Yip, N. Ivascu, and L. Hill, "Innovative ICU Physician Care Models: Covid-19 Pandemic at NewYork Presbyterian," *NEJM Catal.*, Apr. 2020, doi: 10.1056/CAT.20.0158.
[29] P. Echeverría *et al.*, "COVIDApp as an innovative strategy for the management and follow-up of COVID-19 cases in long-term care facilities in Catalonia: Implementation study," *JMIR Public Heal. Surveill.*, vol. 6, no. 3, p. e21163, Jul. 2020, doi: 10.2196/21163.
[30] M. S. Pegorari, D. G. Ohara, A. P. Matos, N. C.R. Iosimuta, V. T.K. Ferreira, and A. Carolina P.N. Pinto, "Barriers and challenges faced by Brazilian physiotherapists during the COVID-19 pandemic and innovative solutions: lessons learned and to be shared with other countries," *Physiother. Theory Pract.*, vol. 36, no. 10, pp. 1069–1076, Oct. 2020, doi: 10.1080/09593985.2020.1818486.
[31] A. Crawford and E. Serhal, "Digital health equity and COVID-19: The innovation curve cannot reinforce the social gradient of health," *Journal of Medical Internet Research*, vol. 22, no. 6. JMIR Publications Inc., p. e19361, Jun. 02, 2020, doi: 10.2196/19361.
[32] E. Monaghesh and A. Hajizadeh, "The role of telehealth during COVID-19 outbreak: A systematic review based on current evidence," *BMC Public Health*, vol. 20, no. 1. BioMed Central, pp. 1–9, Aug. 01, 2020, doi: 10.1186/s12889-020-09301-4.
[33] C. Guida and G. Carpentieri, "Quality of life in the urban environment and primary health services for the elderly during the Covid-19 pandemic: An application to the city of Milan (Italy)," *Cities*, vol. 110, p. 103038, Mar. 2021, doi: 10.1016/j.cities.2020.103038.
[34] A. A. Adamu, R. I. Jalo, A. I. Dahiru, and C. S. Wiysonge, "Implementing COVID-19 response within the context of the broader health system: a proposed framework for Africa´s policy makers," *Pan Afr. Med. J.*, vol. 35, no. Supp 2, May 2020, doi: 10.11604/pamj.supp.2020.35.2.23574.

Section 6. Resources shared

Barasa E , Bennett S , Rao K , Goodman C , Gupta I , Hanvoravongchai P , James C , Maceira D , Witter S , Hanson K (2020) Health Financing in Response to Covid-19: An Agenda For Research. Interagency working paper.

Percival, V., Dusabe-Richards, E., Wurie, H. *et al.* Are health systems interventions gender blind? examining health system reconstruction in conflict affected states. *Global Health* 14, 90 (2018). https://doi.org/10.1186/s12992-018-0401-6

Gilson L, Marchal B, Ayepong I, Barasa E, Dossou JP, George A, Guinaran R, Maceira D, Molyneux S, Prashanth NS, Schneider H. What role can health policy and systems research play in supporting responses to COVID-19 that strengthen socially just health systems?. Health Policy and Planning. 2020 Nov;35(9):1231-6.

Fairgrieve D, Holm S, Howells G, Kirchhelle C, Vanderslott S. In favour of a bespoke COVID-19 vaccines compensation scheme. The Lancet Infectious Diseases. 2021 Apr 1;21(4):448-50.
Cohen IG, Gostin LO, Weitzner DJ. Digital smartphone tracking for COVID-19: public health and civil liberties in tension. Jama. 2020 Jun 16;323(23):2371-2.

Gasser U, Ienca M, Scheibner J, Sleigh J, Vayena E. Digital tools against COVID-19: taxonomy, ethical challenges, and navigation aid. The Lancet Digital Health. 2020 Jun 29.

https://www.chathamhouse.org/events/all/members-event/gender-inequality-making-technology-solution-not-problem

https://humanservices.hawaii.gov/wp-content/uploads/2020/04/4.13.20-Final-Cover-D2-Feminist-Economic-Recovery-D1.pdf

Section 7: Tables with Results of Prioritised Questions

Table 6: High Priority Gender and COVID-19 Research Questions for all criteria

RQ	Label	Public Health	Gender equality	Urgency for policy
RQ12	How did health service delivery measures respond to the needs of pregnant women who tested positive for COVID-19	**X**	X	X
RQ1	How does access and quality of services for COVID-19 differ by gender and its intersection with other social categories (such as race, disability, migrant status, age, sexuality, etc) in various contexts	X	X	X
RQ4	How has the prioritization of COVID-19 services affected access to services for non-COVID-19 health conditions by gender and its intersection with other social categories	**X**	X	X
RQ2	What strategies were used to improve gender and other inequities in access and quality of care for COVID-19 services (testing, facility based care, quarantine care, etc) and how effective were they	**X**	X	X
RQ19	What are the different service reorganization models implemented to ensure continuity of maternal health, sexual, and reproductive and maternal health (SRHR) and violence against women and girls (VAWG) services during the pandemic, and how effective are they	**X**	X	
RQ6	To what extent, and how has, utilization of quality sexual, reproductive and maternal health and violence against women services changed because of COVID-19	X	X	
RQ5	What measures are enacted to maintain and safeguard against interruptions in essential health services by gender and its intersection with other social categories	**X**		X
RQ39	What strategies have been adopted during the pandemic to address disruptions in contraceptive supply, distribution and delivery	X		X

RQ53	How can women and vulnerable populations develop long-term protection against catastrophic healthcare costs due to COVID-19	X	X	
RQ22	Which community-based service delivery models best address gender barriers in access to quality health services during the COVID-19 pandemic	X	X	
RQ37	What are the effective strategies for addressing contextual gender related barriers in vaccine access and delivery to different populations of men, women and gender diverse people	**X**		
RQ17	What is the impact of school closures on access to health services for adolescents by gender across various contexts	**X**		
RQ9	To what extent, and how has, utilization of quality mental health services changed by gender and its intersection with other social categories because of COVID-19	X		
RQ36	What effective measures can be employed to improve the access to key COVID-19 commodities (PPE, vaccines, treatments, oxygen) by vulnerable, marginalized and hard to reach groups	X		
RQ43	How are COVID-19 individual services financed, and how does it affect access to services and financial protection by gender	X		
RQ24	What innovative models exist to address gender barriers in accessing services during the pandemic	X		
RQ18	How has the pandemic and its travel restrictions impacted access to quality health services for migrants by gender and its intersection with other social categories	X		
RQ3	How has access and quality of care for non-COVID-19 conditions changed by gender and its intersection with other social categories from prior to and during the pandemic			X

Supplementary Table 4a: Research Questions for Theme on Health Services ordered by means on Public Health Benefit for Gender and COVID-19 Research Prioritisation

RQ	Label	M	Mean	SD	95% CI
RQ12	How did health service delivery measures respond to the needs of pregnant women who tested positive for COVID-19	**37**	**3.68**	**0.47**	**3.65-3.70**
RQ6	To what extent, and how has, utilization of quality sexual, reproductive and maternal health and violence against women services changed because of COVID-19	**38**	**3.67**	**0.57**	**3.64-3,70**
RQ4	How has the prioritization of COVID-19 services affected access to services for non-COVID-19 health conditions by gender and its intersection with other social categories	**37**	**3.65**	**0.68**	**3.61-3.68**
RQ2	What strategies were used to improve gender and other inequities in access and quality of care for COVID-19 services (testing, facility based care, quarantine care, etc) and how effective were they	**38**	**3.64**	**0.53**	**3.62-3.67**
RQ19	What are the different service reorganization models implemented to ensure continuity of maternal health, sexual, and reproductive and maternal health (SRHR) and violence against women and girls (VAWG) services during the pandemic, and how effective are they	**36**	**3.64**	**0.59**	**3.61-3.67**
RQ5	What measures are enacted to maintain and safeguard against interruptions in essential health services by gender and its intersection with other social categories	**36**	**3.61**	**0.64**	**3.58-3.65**
RQ1	How does access and quality of services for COVID-19 differ by gender and its intersection with other social categories (such as race, disability, migrant status, age, sexuality, etc) in various contexts	**39**	**3.58**	**0.59**	**3.55-3.61**
RQ37	What are the effective strategies for addressing contextual gender related barriers in vaccine access and delivery to different populations of men, women and gender diverse people	**35**	**3.56**	**0.67**	**3.52-3.59**
RQ17	What is the impact of school closures on access to health services for adolescents by gender across various contexts	**38**	**3.54**	**0.62**	**3.51-3.57**
RQ9	To what extent, and how has, utilization of quality mental health services changed by gender and its intersection with other social categories because of COVID-19	**39**	**3.54**	**0.64**	**3.51-3.57**
RQ36	What effective measures can be employed to improve the access to key COVID-19 commodities (PPE, vaccines, treatments, oxygen) by vulnerable, marginalized and hard to reach groups	**35**	**3.53**	**0.65**	**3.49-3.57**
RQ53	How can women and vulnerable populations develop long-term protection against catastrophic healthcare costs due to COVID-19	**37**	**3.53**	**0.62**	**3.49-3.56**

RQ39	What strategies have been adopted during the pandemic to address disruptions in contraceptive supply, distribution and delivery	**36**	**3.51**	**0.71**	**3.48-3.55**
RQ43	How are COVID-19 individual services financed, and how does it affect access to services and financial protection by gender	**32**	**3.48**	**0.69**	**3.44-3.53**
RQ22	Which community-based service delivery models best address gender barriers in access to quality health services during the COVID-19 pandemic	**37**	**3.46**	**0.77**	**3.42-3.50**
RQ24	What innovative models exist to address gender barriers in accessing services during the pandemic	**38**	**3.45**	**0.72**	**3.41-3.48**
RQ18	How has the pandemic and its travel restrictions impacted access to quality health services for migrants by gender and its intersection with other social categories	**37**	**3.45**	**0.68**	**3.41-3.48**
RQ34	What measures improved the gender responsiveness of the health system in supporting health workers during the pandemic	**37**	**3.45**	**0.74**	**3.41-3.49**
RQ41	What improvements should be made to health information systems in terms of reporting COVID-19 sex-disaggregated data and other intersecting social categories	**37**	**3.43**	**0.77**	**3.39-3.47**
RQ26	How were the gendered health risks, co-morbidities and health conditions that health workers themselves had considered when being assigned for COVID-19 duty	**36**	**3.43**	**0.71**	**3.39-3.47**
RQ16	How did gender diverse people experience services for prevention and control of the pandemic, such as quarantine in male/female wards, and vaccination eligibility based on binary categories	**37**	**3.42**	**0.78**	**3.38-3.46**
RQ10	To what extent, and how has utilization of quality services for non-communicable disease and other chronic conditions changed by gender and its intersection with other social categories because of COVID-19	**37**	**3.41**	**0.64**	**3.37-3.44**
RQ14	How has the intersection of social factors, such as national context, socioeconomic status, gender, religion, or geographic location, changed the access of quality health services for LGBTQI+ communities during the pandemic	**34**	**3.40**	**0.76**	**3.35-3.44**
RQ20	How can gender biases in accessing and using digital health interventions, including telemedicine, be addressed during the pandemic	**38**	**3.39**	**0.72**	**3.36-3.43**
RQ28	What measures (including safety and security) need to be put into place to ensure the protection of health service providers who are mostly women and may be travelling to remote areas for COVID-19 services (testing, vaccination etc)	**37**	**3.39**	**0.74**	**3.35-3.43**
RQ15	What are the best ways to support organizations that provide health care services to LGBTQI+ people	**32**	**3.39**	**0.73**	**3.35-3.44**

Gender & Health Hub

	during the pandemic in countries that do not recognize their rights				
RQ27	What were the gender dimensions of vaccine hesitancy among health workers during the pandemic	**33**	**3.38**	**0.67**	**3.34-3.42**
RQ3	How has access and quality of care for non-COVID-19 conditions changed by gender and its intersection with other social categories from prior to and during the pandemic	**37**	**3.38**	**0.76**	**3.34-3.42**
RQ40	Are the differences observed in hospitalization and death rates for males and females influenced by gender differences in the reporting of deaths, and access to, and affordability of care	**35**	**3.37**	**0.69**	**3.33-3.41**
RQ13	How did health service delivery measures respond to the needs of institutionalised adult populations (elderly, disabled, incarcerated)	**38**	**3.36**	**0.73**	**3.32-3.39**
RQ48	To what extent did purchasing mechanisms (e.g. benefit packages, provider selection and payments, and contracting etc.) change to respond to the pandemic, and how did these changes affect health care access and quality across different gender groups	**29**	**3.34**	**0.81**	**3.29-3.40**
RQ33	What are the gendered human resources aspects of delivering care (in terms of staff distribution, division of labour, and experiences of care workers) in COVID-19 facilities	**34**	**3.34**	**0.75**	**3.30-3.38**
RQ44	What is the share of public and private expenditures in financing the continuum of COVID-19 care (from screening to intensive care units) and how does it compare by gender	**29**	**3.33**	**0.78**	**3.27-3.38**
RQ25	What are the gender issues faced by health workers related to personal protective equipment (PPE) throughout the pandemic, and how were they addressed	**36.00**	**3.29**	**0.90**	**3.24-3.34**
RQ32	To what extent did gender norms at household, community and health service level influence health worker availability and productivity during the pandemic	**36.00**	**3.29**	**0.72**	**3.25-3.33**
RQ51	What proportion of the overall vaccine deployment strategy budget and expenditure was allocated and spent on interventions that address specific barriers in access, delivery, and uptake of vaccines among women, men and gender-diverse people	**33.00**	**3.29**	**0.78**	**3.24-3.33**
RQ35	How did the lack of functioning of other sectors, like transport, affect the ability of health workers to deliver services during the pandemic and were there resultant gender implications	**35.00**	**3.29**	**0.75**	**3.24-3.33**
RQ38	How have COVID-19 service delivery changes affected gendered access to non-COVID-19 health commodities	**35.00**	**3.29**	**0.75**	**3.24-3.33**
RQ8	Are there gender differences in access to quality harm reduction services for marginalized populations, including people with substance abuse problems, homeless individuals, and sex workers during the pandemic	**36.00**	**3.28**	**0.74**	**3.24-3.32**

BMJ Publishing Group Limited (BMJ) disclaims all liability and responsibility arising from any reliance placed on this supplemental material which has been supplied by the author(s)

RQ21	Which effective service delivery models support home-based caregiving for COVID-19 and other health conditions during the pandemic in ways that address gender biases in the division of labour	**38.00**	**3.28**	**0.68**	**3.24-3.31**
RQ7	What challenges has the pandemic posed to the survivor-centred approach embedded into holistic gender-based violence services (such as health, legal, and psycho-social)	**37.00**	**3.26**	**0.74**	**3.22-3.30**
RQ11	Has the gendered nature of the access to, and use of, digital health improved or gotten worse due to COVID-19	**39.00**	**3.24**	**0.76**	**3.21-3.28**
RQ49	To what extent did public financial management mechanisms for budget formulation, execution, and monitoring facilitate gender responsiveness in addressing service delivery and access challenges during the pandemic	**31.00**	**3.24**	**0.83**	**3.19-3.29**
RQ52	What are the possible immediate mechanisms (including wider social protection schemes) to protect woman-headed households against health expenditures (and the wider economic shock effects) during the pandemic	**37.00**	**3.19**	**0.84**	**3.14-3.23**
RQ23	How did the private and public sector compare in terms of responsiveness of COVID-19 services for women, men and gender diverse people	**34.00**	**3.18**	**0.83**	**3.13-3.22**
RQ42	How has the increased reliance on digital modes of health information systems during the pandemic changed biases and capability for sex-disaggregated data reporting and analysis	**35.00**	**3.17**	**0.82**	**3.13-3.22**
RQ45	What are the implications of re-allocation or re-prioritization of funding for access to health care across different gender groups during the COVID-19 pandemic	**35.00**	**3.17**	**0.79**	**3.13-3.22**
RQ46	How are funds in charities and volunteer bodies allocated across different gender and vulnerable groups in order to improve access and quality of health services	**30.00**	**3.15**	**0.84**	**3.09-3.21**
RQ29	What kinds of pandemic related stigma affected health workers by gender, occupation, and service location, and how best can these be addressed	**37.00**	**3.15**	**0.84**	**3.10-3.19**
RQ31	Did measures taken for surge capacity, including recruitment reforms, have different impacts on health workers based on gender and occupation	**32.00**	**3.13**	**0.79**	**3.08-3.17**
RQ47	What were the different modalities in financing used to fund vaccine deployment and access, and what are the gender impacts of these different approaches	**32.00**	**3.11**	**0.80**	**3.06-3.16**
RQ50	To what extent did purchasing services from private sectors through public resources help gender equality in access to care during and beyond the pandemic	**29.00**	**3.10**	**0.82**	**3.05-3.16**
RQ54	How can cross-subsidy between male and female payers be encouraged in favour of vulnerable groups				

George AS, *et al. BMJ Global Health* 2023; 8:e011315. doi: 10.1136/bmjgh-2022-011315

	such as women workers, within different pooled insurance funds during pandemic	**34.00**	**3.09**	**0.83**	**3.04-3.14**
RQ30	To what extent are there gendered inequities in access to training opportunities, including e-learning technologies, for health workers delivering COVID-19 services, and how best can they be addressed	**37.00**	**2.99**	**0.75**	**2.95-3.03**

Supplementary Table 4b: Research Questions for Theme on Health Services ordered by means on Gender Equality for Gender and COVID-19 Research Prioritisation

**RQ**	**Label**	**N**	**Mean**	**SD**	**95% CI**
RQ4	How has the prioritization of COVID-19 services affected access to services for non-COVID-19 health conditions by gender and its intersection with other social categories	**40**	**3.59**	**0.72**	**3.55-3.62**
RQ2	What strategies were used to improve gender and other inequities in access and quality of care for COVID-19 services (testing, facility based care, quarantine care, etc) and how effective were they	**40**	**3.54**	**0.71**	**3.50-3.57**
RQ53	How can women and vulnerable populations develop long-term protection against catastrophic healthcare costs due to COVID-19	**38**	**3.51**	**0.78**	**3.47-3.55**
RQ1	How does access and quality of services for COVID-19 differ by gender and its intersection with other social categories (such as race, disability, migrant status, age, sexuality, etc) in various contexts	**41**	**3.48**	**0.81**	**3.44 -3.51**
RQ6	To what extent, and how has, utilization of quality sexual, reproductive and maternal health and violence against women services changed because of COVID-19	**39**	**3.45**	**0.88**	**3.40 -3.49**
RQ19	What are the different service reorganization models implemented to ensure continuity of maternal health, sexual, and reproductive and maternal health (SRHR) and violence against women and girls (VAWG) services during the pandemic, and how effective are they	**37**	**3.45**	**0.70**	**3.41 -3.48**
RQ12	How did health service delivery measures respond to the needs of pregnant women who tested positive for COVID-19	**39**	**3.44**	**0.79**	**3.40 -3.48**
RQ22	Which community-based service delivery models best address gender barriers in access to quality health services during the COVID-19 pandemic	**38**	**3.39**	**0.82**	**3.35 -3.44**
RQ9	To what extent, and how has, utilization of quality mental health services changed by gender and its intersection with other social categories because of COVID-19	**40**	**3.35**	**0.77**	**3.31 -3.39**
RQ52	What are the possible immediate mechanisms (including wider social protection schemes) to	**3**	**3.34**	**0.88**	**3.30 -3.39**

Gender & Health Hub

	protect woman-headed households against health expenditures (and the wider economic shock effects) during the pandemic	**8**			
RQ5	What measures are enacted to maintain and safeguard against interruptions in essential health services by gender and its intersection with other social categories	**37**	**3.34**	**0.73**	**3.30 -3.38**
RQ3	How has access and quality of care for non-COVID-19 conditions changed by gender and its intersection with other social categories from prior to and during the pandemic	**39**	**3.32**	**0.73**	**3.28 -3.36**
RQ17	What is the impact of school closures on access to health services for adolescents by gender across various contexts	**40**	**3.31**	**0.90**	**3.27 -3.36**
RQ7	What challenges has the pandemic posed to the survivor-centred approach embedded into holistic gender-based violence services (such as health, legal, and psycho-social)	**39**	**3.29**	**0.84**	**3.25-3.34**
RQ37	What are the effective strategies for addressing contextual gender related barriers in vaccine access and delivery to different populations of men, women and gender diverse people	**38**	**3.29**	**0.90**	**3.24 -3.34**
RQ21	Which effective service delivery models support home-based caregiving for COVID-19 and other health conditions during the pandemic in ways that address gender biases in the division of labour	**39**	**3.28**	**0.72**	**3.25 -3.32**
RQ41	What improvements should be made to health information systems in terms of reporting COVID-19 sex-disaggregated data and other intersecting social categories	**38**	**3.26**	**0.86**	**3.22 -3.31**
RQ43	How are COVID-19 individual services financed, and how does it affect access to services and financial protection by gender	**37**	**3.26**	**0.91**	**3.21 -3.30**
RQ24	What innovative models exist to address gender barriers in accessing services during the pandemic	**39**	**3.26**	**0.91**	**3.21 -3.30**
RQ20	How can gender biases in accessing and using digital health interventions, including telemedicine, be addressed during the pandemic	**39**	**3.23**	**0.84**	**3.19 -3.27**
RQ34	What measures improved the gender responsiveness of the health system in supporting health workers during the pandemic	**38**	**3.22**	**0.79**	**3.18 -3.26**
RQ28	What measures (including safety and security) need to be put into place to ensure the protection of health service providers who are mostly women and may be travelling to remote areas for COVID-19 services (testing, vaccination etc)	**38**	**3.20**	**0.78**	**3.16 -3.24**
RQ39	What strategies have been adopted during the pandemic to address disruptions in contraceptive supply, distribution and delivery	**38**	**3.20**	**0.91**	**3.15 -3.24**

Gender & Health Hub

RQ18	How has the pandemic and its travel restrictions impacted access to quality health services for migrants by gender and its intersection with other social categories	**39**	**3.17**	**0.93**	**3.12 -3.21**
RQ51	What proportion of the overall vaccine deployment strategy budget and expenditure was allocated and spent on interventions that address specific barriers in access, delivery, and uptake of vaccines among women, men and gender-diverse people	**35**	**3.16**	**0.90**	**3.11 -3.21**
RQ36	What effective measures can be employed to improve the access to key COVID-19 commodities (PPE, vaccines, treatments, oxygen) by vulnerable, marginalized and hard to reach groups	**37**	**3.15**	**0.90**	**3.10 -3.20**
RQ38	How have COVID-19 service delivery changes affected gendered access to non-COVID-19 health commodities	**37**	**3.14**	**0.92**	**3.09 -3.18**
RQ26	How were the gendered health risks, co-morbidities and health conditions that health workers themselves had considered when being assigned for COVID-19 duty	**38**	**3.13**	**0.84**	**3.09 -3.18**
RQ11	Has the gendered nature of the access to, and use of, digital health improved or gotten worse due to COVID-19	**40**	**3.13**	**0.82**	**3.08 -3.17**
RQ33	What are the gendered human resources aspects of delivering care (in terms of staff distribution, division of labour, and experiences of care workers) in COVID-19 facilities	**36**	**3.13**	**0.81**	**3.08 -3.17**
RQ32	To what extent did gender norms at household, community and health service level influence health worker availability and productivity during the pandemic	**38**	**3.12**	**0.91**	**3.07 -3.17**
RQ10	To what extent, and how has utilization of quality services for non-communicable disease and other chronic conditions changed by gender and its intersection with other social categories because of COVID-19	**38**	**3.11**	**0.89**	**3.06 -3.15**
RQ49	To what extent did public financial management mechanisms for budget formulation, execution, and monitoring facilitate gender responsiveness in addressing service delivery and access challenges during the pandemic	**35**	**3.10**	**0.97**	**3.05 -3.15**
RQ54	How can cross-subsidy between male and female payers be encouraged in favour of vulnerable groups such as women workers, within different pooled insurance funds during pandemic	**36**	**3.10**	**0.86**	**3.05 -3.14**
RQ40	Are the differences observed in hospitalization and death rates for males and females influenced by gender differences in the reporting of deaths, and access to, and affordability of care	**37**	**3.09**	**0.85**	**3.05 -3.14**
RQ16	How did gender diverse people experience services for prevention and control of the pandemic,	**3**	**3.09**	**0.97**	**3.04-3.14**

	such as quarantine in male/female wards, and vaccination eligibility based on binary categories	**9**			
RQ14	How has the intersection of social factors, such as national context, socioeconomic status, gender, religion, or geographic location, changed the access of quality health services for LGBTQI+ communities during the pandemic	**38**	**3.07**	**1.03**	**3.01 -3.12**
RQ13	How did health service delivery measures respond to the needs of institutionalised adult populations (elderly, disabled, incarcerated)	**40**	**3.06**	**0.92**	**3.02-3.11**
RQ48	To what extent did purchasing mechanisms (e.g. benefit packages, provider selection and payments, and contracting etc.) change to respond to the pandemic, and how did these changes affect health care access and quality across different gender groups	**33**	**3.06**	**1.03**	**3.00 -3.12**
RQ15	What are the best ways to support organizations that provide health care services to LGBTQI+ people during the pandemic in countries that do not recognize their rights	**36**	**3.04**	**0.99**	**2.99-3.10**
RQ29	What kinds of pandemic related stigma affected health workers by gender, occupation, and service location, and how best can these be addressed	**38**	**3.00**	**0.96**	**2.95 -3.05**
RQ8	Are there gender differences in access to quality harm reduction services for marginalized populations, including people with substance abuse problems, homeless individuals, and sex workers during the pandemic	**39**	**2.99**	**0.83**	**2.95 -3.03**
RQ35	How did the lack of functioning of other sectors, like transport, affect the ability of health workers to deliver services during the pandemic and were there resultant gender implications	**36**	**2.99**	**0.93**	**2.94 -3.04**
RQ44	What is the share of public and private expenditures in financing the continuum of COVID-19 care (from screening to intensive care units) and how does it compare by gender	**36**	**2.99**	**0.96**	**2.93 -3.04**
RQ45	What are the implications of re-allocation or re-prioritization of funding for access to health care across different gender groups during the COVID-19 pandemic	**38**	**2.97**	**0.88**	**2.93 -3.02**
RQ23	How did the private and public sector compare in terms of responsiveness of COVID-19 services for women, men and gender diverse people	**37**	**2.97**	**1.04**	**2.92 -3.03**
RQ31	Did measures taken for surge capacity, including recruitment reforms, have different impacts on health workers based on gender and occupation	**36**	**2.97**	**0.91**	**2.92 -3.02**
RQ42	How has the increased reliance on digital modes of health information systems during the pandemic changed biases and capability for sex-disaggregated data reporting and analysis	**36**	**2.97**	**0.91**	**2.92 -3.02**
RQ27	What were the gender dimensions of vaccine hesitancy among health workers during the pandemic	**37**	**2.95**	**0.94**	**2.90 -3.00**

RQ30	To what extent are there gendered inequities in access to training opportunities, including e-learning technologies, for health workers delivering COVID-19 services, and how best can they be addressed	**38**	**2.93**	**0.77**	**2.89 -2.97**
RQ25	What are the gender issues faced by health workers related to personal protective equipment (PPE) throughout the pandemic, and how were they addressed	**39**	**2.90**	**1.05**	**2.84 -2.95**
RQ46	How are funds in charities and volunteer bodies allocated across different gender and vulnerable groups in order to improve access and quality of health services	**36**	**2.85**	**1.05**	**2.79 -2.90**
RQ47	What were the different modalities in financing used to fund vaccine deployment and access, and what are the gender impacts of these different approaches	**35**	**2.73**	**1.04**	**2.67 -2.79**
RQ50	To what extent did purchasing services from private sectors through public resources help gender equality in access to care during and beyond the pandemic	**35**	**2.69**	**1.02**	**2.63 -2.74**

Supplementary Table 4c: Research Questions for Theme on Health Services ordered by means on Urgency for Gender and COVID-19 Research Prioritisation

**RQ**	**Label**	**M**	**Mean**	**SD**	**95% CI**
RQ12	How did health service delivery measures respond to the needs of pregnant women who tested positive for COVID-19	**40**	**2.50**	**0.72**	**2.46-2.54**
RQ1	How does access and quality of services for COVID-19 differ by gender and its intersection with other social categories (such as race, disability, migrant status, age, sexuality, etc) in various contexts	**40**	**2.49**	**0.76**	**2.45 -2.52**
RQ4	How has the prioritization of COVID-19 services affected access to services for non-COVID-19 health conditions by gender and its intersection with other social categories	**39**	**2.44**	**0.75**	**2.40 -2.47**
RQ5	What measures are enacted to maintain and safeguard against interruptions in essential health services by gender and its intersection with other social categories	**37**	**2.43**	**0.73**	**2.39 -2.47**
RQ39	What strategies have been adopted during the pandemic to address disruptions in contraceptive supply, distribution and delivery	**35**	**2.33**	**0.83**	**2.28-2.38**
RQ2	What strategies were used to improve gender and other inequities in access and quality of care for COVID-19 services (testing, facility based care, quarantine care, etc) and how effective were they	**39**	**2.32**	**0.77**	**2.28 -2.36**
RQ3	How has access and quality of care for non-COVID-19 conditions changed by gender and its intersection with other social categories from prior to and during the pandemic	**37**	**2.30**	**0.70**	**2.26-2.33**

RQ13	How did health service delivery measures respond to the needs of institutionalised adult populations (elderly, disabled, incarcerated)	**39**	**2.29**	**0.79**	**2.26 -2.33**
RQ43	How are COVID-19 individual services financed, and how does it affect access to services and financial protection by gender	**34**	**2.28**	**0.79**	**2.23-2.32**
RQ25	What are the gender issues faced by health workers related to personal protective equipment (PPE) throughout the pandemic, and how were they addressed	**33**	**2.27**	**0.88**	**2.22-2.32**
RQ27	What were the gender dimensions of vaccine hesitancy among health workers during the pandemic	**35**	**2.27**	**0.82**	**2.23-2.32**
RQ6	To what extent, and how has, utilization of quality sexual, reproductive and maternal health and violence against women services changed because of COVID-19	**39**	**2.26**	**0.79**	**2.22-2.30**
RQ37	What are the effective strategies for addressing contextual gender related barriers in vaccine access and delivery to different populations of men, women and gender diverse people	**37**	**2.23**	**0.82**	**2.19-2.27**
RQ28	What measures (including safety and security) need to be put into place to ensure the protection of health service providers who are mostly women and may be travelling to remote areas for COVID-19 services (testing, vaccination etc)	**36**	**2.22**	**0.83**	**2.18-2.27**
RQ36	What effective measures can be employed to improve the access to key COVID-19 commodities (PPE, vaccines, treatments, oxygen) by vulnerable, marginalized and hard to reach groups	**36**	**2.22**	**0.83**	**2.18-2.27**
RQ9	To what extent, and how has, utilization of quality mental health services changed by gender and its intersection with other social categories because of COVID-19	**38**	**2.18**	**0.73**	**2.15-2.22**
RQ26	How were the gendered health risks, co-morbidities, and health conditions that health workers themselves had considered when being assigned for COVID-19 duty	**35**	**2.17**	**0.79**	**2.13-2.22**
RQ38	How have COVID-19 service delivery changes affected gendered access to non-COVID-19 health commodities	**35**	**2.17**	**0.75**	**2.13-2.21**
RQ34	What measures improved the gender responsiveness of the health system in supporting health workers during the pandemic	**37**	**2.15**	**0.84**	**2.10-2.19**
RQ14	How has the intersection of social factors, such as national context, socioeconomic status, gender, religion, or geographic location, changed the access of quality health services for LGBTQI+ communities during the pandemic	**33**	**2.14**	**0.72**	**2.09-2.18**
RQ31	Did measures taken for surge capacity, including recruitment reforms, have different impacts on health workers based on gender and occupation	**31**	**2.13**	**0.81**	**2.08-2.18**
RQ21	Which effective service delivery models support home-based caregiving for COVID-19 and other health conditions during the pandemic in ways that address gender biases in the division of labour	**36**	**2.11**	**0.78**	**2.07-2.15**
RQ2	Which community-based service delivery models best address gender barriers in access to quality health services during the COVID-19 pandemic	**36**	**2.11**	**0.82**	**2.07-2.16**

Gender & Health Hub

RQ40	Are the differences observed in hospitalization and death rates for males and females influenced by gender differences in the reporting of deaths, and access to, and affordability of care	**36**	**2.11**	**0.82**	**2.07-2.16**
RQ7	What challenges has the pandemic posed to the survivor-centred approach embedded into holistic gender-based violence services (such as health, legal, and psycho-social)	**36**	**2.11**	**0.75**	**2.07-2.15**
RQ46	How are funds in charities and volunteer bodies allocated across different gender and vulnerable groups in order to improve access and quality of health services	**32**	**2.11**	**0.78**	**2.06-2.16**
RQ17	What is the impact of school closures on access to health services for adolescents by gender across various contexts	**40**	**2.10**	**0.87**	**2.06-2.14**
RQ29	What kinds of pandemic related stigma affected health workers by gender, occupation, and service location, and how best can these be addressed	**35**	**2.10**	**0.89**	**2.05-2.15**
RQ45	What are the implications of re-allocation or re-prioritization of funding for access to health care across different gender groups during the COVID-19 pandemic	**35**	**2.09**	**0.82**	**2.04-2.13**
RQ16	How did gender diverse people experience services for prevention and control of the pandemic, such as quarantine in male/female wards, and vaccination eligibility based on binary categories	**37**	**2.08**	**0.68**	**2.04-2.12**
RQ23	How did the private and public sector compare in terms of responsiveness of COVID-19 services for women, men and gender diverse people	**35**	**2.06**	**0.87**	**2.01-2.11**
RQ44	What is the share of public and private expenditures in financing the continuum of COVID-19 care (from screening to intensive care units) and how does it compare by gender	**31**	**2.05**	**0.80**	**2.00-2.10**
RQ48	To what extent did purchasing mechanisms (e.g. benefit packages, provider selection and payments, and contracting etc.) change to respond to the pandemic, and how did these changes affect health care access and quality across different gender groups	**31**	**2.05**	**0.78**	**2.00-2.10**
RQ33	What are the gendered human resources aspects of delivering care (in terms of staff distribution, division of labour, and experiences of care workers) in COVID-19 facilities	**34**	**2.04**	**0.87**	**1.99-2.09**
RQ20	How can gender biases in accessing and using digital health interventions, including telemedicine, be addressed during the pandemic	**38**	**2.04**	**0.79**	**2.00-2.08**
RQ32	To what extent did gender norms at household, community and health service level influence health worker availability and productivity during the pandemic	**35**	**2.03**	**0.86**	**1.98-2.08**
RQ41	What improvements should be made to health information systems in terms of reporting COVID-19 sex-disaggregated data and other intersecting social categories	**37**	**2.03**	**0.87**	**1.98-2.07**
RQ52	What are the possible immediate mechanisms (including wider social protection schemes) to protect woman-headed households against health expenditures (and the wider economic shock effects) during the pandemic	**38**	**2.03**	**0.82**	**1.98-2.07**
RQ19	What are the different service reorganization models implemented to ensure continuity of maternal health, sexual, and reproductive and maternal health (SRHR) and violence against women and girls (VAWG)	**36**	**2.01**	**0.76**	**1.97-2.06**

	services during the pandemic, and how effective are they				
RQ24	What innovative models exist to address gender barriers in accessing services during the pandemic	**36**	**2.00**	**0.83**	**1.95-2.05**
RQ30	To what extent are there gendered inequities in access to training opportunities, including e-learning technologies, for health workers delivering COVID-19 services, and how best can they be addressed	**35**	**2.00**	**0.87**	**1.95-2.05**
RQ35	How did the lack of functioning of other sectors, like transport, affect the ability of health workers to deliver services during the pandemic and were there resultant gender implications	**37**	**1.99**	**0.85**	**1.94-2.03**
RQ51	What proportion of the overall vaccine deployment strategy budget and expenditure was allocated and spent on interventions that address specific barriers in access, delivery, and uptake of vaccines among women, men and gender-diverse people	**35**	**1.99**	**0.86**	**1.94-2.03**
RQ50	To what extent did purchasing services from private sectors through public resources help gender equality in access to care during and beyond the pandemic	**31**	**1.98**	**0.78**	**1.93-2.03**
RQ15	What are the best ways to support organizations that provide health care services to LGBTQI+ people during the pandemic in countries that do not recognize their rights	**33**	**1.97**	**0.73**	**1.93-2.01**
RQ47	What were the different modalities in financing used to fund vaccine deployment and access, and what are the gender impacts of these different approaches	**36**	**1.96**	**0.81**	**1.91-2.00**
RQ11	Has the gendered nature of the access to, and use of, digital health improved or gotten worse due to COVID-19	**38**	**1.95**	**0.84**	**1.90-1.99**
RQ42	How has the increased reliance on digital modes of health information systems during the pandemic changed biases and capability for sex-disaggregated data reporting and analysis	**35**	**1.94**	**0.84**	**1.90-1.99**
RQ18	How has the pandemic and its travel restrictions impacted access to quality health services for migrants by gender and its intersection with other social categories	**40**	**1.93**	**0.83**	**1.88-1.97**
RQ10	To what extent, and how has utilization of quality services for non-communicable disease and other chronic conditions changed by gender and its intersection with other social categories because of COVID-19	**37**	**1.92**	**0.76**	**1.88-1.96**
RQ8	Are there gender differences in access to quality harm reduction services for marginalized populations, including people with substance abuse problems, homeless individuals, and sex workers during the pandemic	**37**	**1.92**	**0.83**	**1.87-1.96**
RQ53	How can women and vulnerable populations develop long-term protection against catastrophic healthcare costs due to COVID-19	**37**	**1.88**	**0.79**	**1.84-1.92**
RQ54	How can cross-subsidy between male and female payers be encouraged in favour of vulnerable groups such as women workers, within different pooled insurance funds during pandemic	**34**	**1.84**	**0.75**	**1.80-1.88**
RQ49	To what extent did public financial management mechanisms for budget formulation, execution, and monitoring facilitate gender responsiveness in addressing service delivery and access challenges during the	**32**	**1.83**	**0.77**	**1.78-1.88**

pandemic

Gender &
Health Hub

George AS, *et al. BMJ Global Health* 2023; 8:e011315. doi: 10.1136/bmjgh-2022-011315
