# Supplementary material for: A shared agenda for gender and COVID-19 research: priorities based on broadening engagement in science
Source: BMJ Glob Health. 2023 May 22;8(5):e011315. doi: 10.1136/bmjgh-2022-011315 (PMC10230361; doi:10.1136/bmjgh-2022-011315)
Supplement: Supplementary data [file bmjgh-2022-011315supp006.pdf]

Gender & COVID-19 Research Agenda-Setting

Draft Thematic Reports for External Consultation

Responses invited through www.ghhbuzzboard.org or via Google drive

Thematic group 4

Social and structural determinants

Social and structural determinants of health and gender dynamics impacted by COVID-19

Contents

Section 1: Introduction to the overall collaboration 2
Section 2. Thematic group participation and engagement 3
Section 3. Thematic group background 4
2.1 Definitions and scope 4
2.2 Current understanding, challenges, gaps and neglected areas 6
2.3 Desired impact of the proposed research 12
2.4 Actors and strategies to implement and promote uptake 13
2.5 Population, contexts, study design/ methodologies 13
Section 4. Research questions proposed for prioritisation. 15
Section 5. References 18
Section 6. Resources shared 25

Section 7: Results of prioritised research questions

Section 1: Introduction to the overall collaboration

From the start of the COVID-19 pandemic, how it affected and continues to affect women, girls, men, boys, and non-binary gender groups are complex and evolving. Apart from the direct effects of COVID-19 illness, pandemic responses also amplified existing gender inequalities across multiple dimensions. Context and the intersecting influence of other social determinants or identities[1,2] also worsened the influence of gender during the pandemic, with combined effects on health.

Early high-level calls and advocacy from researchers[3] such as through the Gender and COVID-19 working group,[4] were made for gender considerations to be integrated in the crisis response. Nevertheless, real-time response to the gender dynamics was limited by extensive invisibility of the evolving situation, incomplete data systems and evidence gaps. As the world steps into the second year of the COVID-19 crisis, given the gender dynamics involved, we must include gender in the investments being made in research informing both immediate action and long-term recovery from the health and socio-economic consequences of the pandemic.

The United Nations University International Institute for Global Health is co-convening a collaborative gender and COVID-19 research agenda-setting exercise, as part of its Gender and Health Hub's inaugural scope of work. The process is co-developed through real-time learning, and open calls to a broad range of stakeholders to comment and contribute to its design, scope and content. Collective contributions and questions for prioritization are supported by a community discussion board (www.ghhbuzzboard.org). Please visit this discussion board for further information.

The output of the exercise will be a shared research agenda that can be utilized by researchers, funders, and policy-makers to guide COVID-19 research investments and corresponding programming and policy actions by the health sector.

The draft thematic group reports emerging from this collective endeavour are a synthesized version of the contributions made to the discussion board combined with additional inputs from thematic group coordinators, co-leads and steering committee members. They document participation and engagement to date, provide a background section outlining definitions, scope, gaps, impact and audiences, before listing research questions for prioritisation.

We welcome your comments on the discussion board or through the google drive to be posted on the discussion board to ensure we respect the inclusive and transparent ethos of the collaboration. If you comment via the google drive please make sure we can identify your comments (do not use anonymous). Given the devastating and dynamic nature of COVID-19, we must be inclusive but also timely.

Section 2. Thematic group participation and engagement

Thematic group coordinator: Oluwapelumi Adeyera (Nigeria)

Thematic group co-leads: Atria Mier (Spain), Peace Musiimenta (Uganda)

Thematic group steering committee focal point: Michelle Remme (Malaysia)

Additional editorial/writing support from UNU-IIGH: Hyecinn Cecilia Loh (UK) & Fatima Ghani (Malaysia)

Other thematic group contributors:

1. Amany Refaat
2. Amber Peterman
3. Anjana Bhushan
4. Apurvakumar Pandya
5. Apurvakumar Pandya
6. Boladale Mapayi
7. Carmen Logie
8. Clara Rodriguez Ribas
9. Comfort Hajra Mukasa
10. Diana Pinzón
11. Dr Ranjini Raghavendra
12. Fui Ching Lam
13. Godwin Aja
14. Graça Viegas
15. Hilda Mwakatumbula
16. Jackielyn Ruiz
17. Jashodhara Dasgupta
18. Jean Providence Nzabonimpa
19. Johnson Jament
20. Kene Esom
21. Khawar Mumtaz
22. Kimberly Dickman
23. Lorena Santos
24. Madalina Grigorovici
25. Maison Hassan
26. Mamothena Mothupi
27. Manasee Mishra
28. Marcos Signorelli
29. Maria da Graça Viegas
30. Mariam Otmani Del Barrio
31. Maya Gislason
32. Megan O'Donnell
33. Mercy Makpor
34. Mini P. Thomas
35. Nadia
36. Prabha Thangaraj
37. Rai Sow
38. Raul Mercer
39. Renu Khanna
40. Rizwanah Souket
41. Ronald Musizvingoza

42. Sharon Attipoe-Dorcoo
43. Sukanya Mohanty
44. Tanya Jacobs
45. Teresa Farinha
46. Vincent Wagner
47. Yasmeen Qazi

First draft report posted on discussion board on date: 29/01/2021
Second draft report posted on discussion board on date: 05/02/2021
Third draft report posted on discussion board on date: 18/03/2021
Fourth draft posted on discussion board on date: 12/05/2021

Section 3. Thematic group background

2.1 Definitions and scope

The WHO Commission on the Social Determinants of Health (CSDH) defined the structural determinants of health as "those that generate or reinforce stratification in the society and that define individual socioeconomic position within hierarchies of power, prestige and access to resources". [5] Gender stands out as a critical determinant that intersects with several others, including social class, ethnicity, level of education and income. These socioeconomic positions in turn shape specific social determinants of health status. Individuals experience different levels of exposure and vulnerability to health-compromising conditions, based on their respective social status. The main social determinants of health relate to material circumstances, psychosocial factors, behavioural and/or biological factors, and the health system itself. [5]

This thematic report considers the impact of the COVID-19 pandemic on specific social and structural determinants of health with strong gendered manifestations, including gender-based violence; income, occupation, labour and unpaid care; social norms and structures; environmental determinants of health; education and schooling, etc. The focus is also on solutions and approaches to mitigate and tackle these negative impacts and amplify any positive effects on gender dynamics.

Issues related to health behaviour and health status are discussed under Thematic Group 1, while issues related to health service delivery through the health system and health system governance are covered by Thematic Group 3 and Thematic Group 5 respectively.

Figure 1. Social and structural determinants of health Conceptual Framework

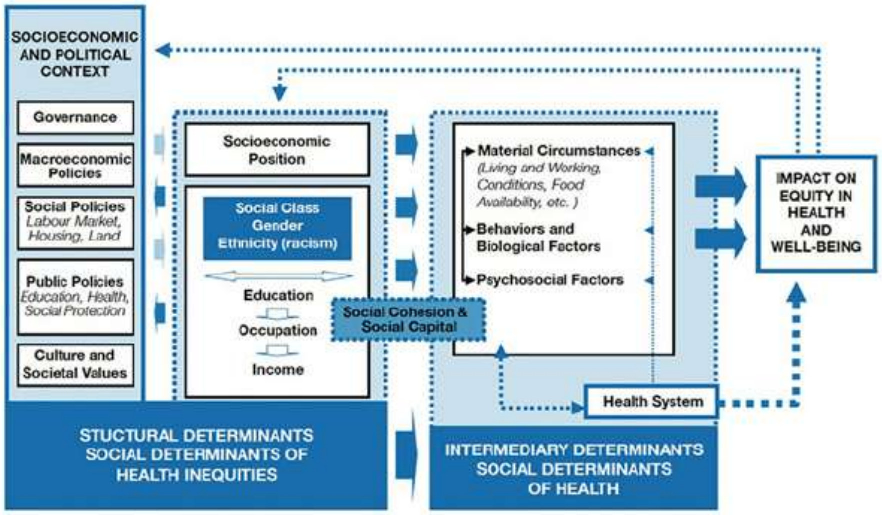

Source: Solar O, Irwin A. A conceptual framework for action on the social determinants of health. Social Determinants of Health Discussion Paper 2 (Policy and Practice). Geneva: World Health Organization.

Table 1. Key definitions[8–12]

Term	Definition
Violence against women (VAW)	Violence against women and girls is defined as any act of gender-based violence that results in, or is likely to result in, physical, sexual or mental harm or suffering to women and girls, including threats of such acts, coercion or arbitrary deprivation of liberty, whether occurring in public or in private life.
Intimate Partner Violence (IPV)	Intimate partner violence is a specific type of GBV that refers to any behavioural pattern that enables one to exercise control/power over an intimate partner.
Child Marriage	Child marriage refers to any marriage where one or both of the spouses are below the age of 18.
Online or digital violence	Online or digital violence against women refers to any act of violence that is committed, assisted or aggravated by the use of information and communication technology (mobile phones, the Internet, social media, computer games, text messaging, email, etc) against a woman because she is a woman.
Gender	Gender refers to the socially constructed attributes and roles associated with being male or female. These attributes and roles define what is expected, allowed, and valued in women and men.
Social norms	Shared expectations or informal rules among a set of people (a reference group) as to how people should behave.
Gender norms	Gender norms are social norms that relate specifically to gender differences: informal rules and shared social expectations that distinguish expected behaviour on the basis of gender
Power	Power includes the ability to make decisions about one's life and the capacity to influence and/or effect desired goals.
Masculinity	Masculinity is the particular pattern of social behaviors or practices that is associated with ideals about how men should behave and their position within gender relations.
Patriarchy	A system of social structures and practices, in which men hold primary power and dominate in roles of political leadership, moral authority, social privilege and control of resources.
Intersectionality	The way people's social identities can overlap; a prism for seeing the way in which various forms of inequality often operate together and exacerbate each other,. Intersectionality

	promotes an understanding of human beings as shaped by the interaction of different social layers/identities e.g., 'race'/ ethnicity, Indigeneity, gender, class, sexuality, geography, age, ableism, migration status, marital status, religion, level of literacy, etc. Some intersections are believed to be universal (gender, age, race among other) meanwhile others are contextual.
Health-related stigma	The negative association between a person or group of people who share certain characteristics and a specific disease.

2.2 Current understanding, challenges, gaps and neglected areas *(outline of key themes, to be finalised at the end)*

Key themes

- Gender Based Violence (GBV)
- Occupation, Income and Labour
- Social and gender norms
- Stigma and discrimination
- Education, Social Infrastructure and Support
- Environmental determinants of health (indoor/outdoor air pollution, WASH)

Gender Based Violence (GBV)

As one of the worst manifestations of gender inequality, the predicted, reported and experienced increase in gender-based violence following the first wave of lockdowns was identified as an acute priority within this research agenda. The World Health Organization defines Gender-Based Violence (GBV) as "any harmful act that is perpetrated against a person's will and that is based on socially ascribed (gender) differences between males and females"[6]. Sometimes GBV is used interchangeably with the term 'violence against women' (VAW), although men, boys and non-conforming gender populations may also experience GBV[7]. GBV manifests in different forms, including physical violence, sexual violence, harmful traditional practices (including female genital mutilation, child marriage, forced pregnancies), economic violence, emotional and psychological violence[7]. It occurs in the home, workplace, public and even online space; and is perpetrated by a range of perpetrators, from intimate partners to strangers.

Gender-based violence (GBV) was a pandemic before COVID-19, with 1 in every 3 women (aged 15-49) reporting that they had experienced physical and/or sexual partner violence in their lifetime[13]. Pandemics as well as other periods of civil unrest and disaster have been known to favour a range of risk factors that increase and aggravate different forms of GBV[14]. Early on in the COVID-19 pandemic, anecdotal evidence and media reports on increased GBV were widespread in countries like China, the United States, Brazil and Australia[14].

The pathways through which GBV may be exacerbated include pandemic-related economic insecurity; inability to escape partners during quarantines and isolation; reduced access to health and social services addressing GBV; and exposure to violence and coercion as a result of pandemic response efforts[14].

Economic insecurity and poverty that are aggravated during pandemics have been found to increase household stress, enabling conflicts, arguments and strife in the home. Loss of jobs and lower income by male heads of households have also been associated with increased abusive and manipulative behaviours towards women[14]. There is more mixed evidence on the relative impact of male unemployment and female unemployment on IPV. Globally, male unemployment has been a trigger

of IPV, whereas female unemployment has been protective[15]. In some studies in high-income countries, however, the opposite was observed, where an increase in a woman's relative economic position in the household led to a decrease in IPV[14,16].

Quarantine and isolation measures during pandemics and health crises (such as SARS) can increase poor mental health in both the short and long term, with negative coping strategies (including substance abuse), which are risk factors for the perpetration and experience of IPV[17,18]. These measures further increase the exposure of women, girls and gender-diverse people to potential perpetrators on a daily basis. As evidenced in refugee camps and humanitarian settings, being in close proximity under conditions of stress and duress for long periods of time heightens GBV risk[19–21]. It also makes it difficult for survivors to call helplines or other support mechanisms during the abuse, or to leave after crises[22]. While only 7% of GBV female survivors and 1-25% of child survivors of violence in developing countries seek formal support[23,24], pandemic measures and limits on mobility have further disrupted access to legal, social or other support services for survivors[14,25]. Consequently, help and services required to limit exposure to violence or help women navigate healing from it has been hard to access[14].

In addition, the breakdown in societal infrastructure and social safety nets during pandemic outbreaks can increase risky, unsafe situations and exploitative relationships that result in sexual violence and harassment, as observed during the Ebola outbreak in West Africa and aid distributions in Lebanon and Uganda, among others[26–28]. The threat of the crisis on existing informal and formal structures has also been associated with increases in child labour, child marriage, marital rape, and diverse forms of exploitation and abuse[14].

Before this pandemic, the GBV field had built a substantial body of evidence on effective interventions and approaches to respond to the needs of survivors, and even to prevent violence against women and girls[29]. The WHO and UN Women RESPECT Women framework[30] highlights these effective strategies, which include economic support to poor households (e.g. cash transfers or food support), couples interventions encouraging conflict resolution skills and shared decision-making, and accessible survivor-centred services.

To date, a growing evidence base on the impact of the COVID-19 pandemic on the level of GBV has generally estimated significant increases in IPV and domestic violence[31–33]. Some research has also been conducted on the determinants and risk factors of GBV during the pandemic, but most of these studies have relied on small and non-representative samples[31]. In addition, although the pandemic response has been characterised by physical distancing, isolation and new online spaces, there is a lack of evidence on how some forms of GBV have changed in terms of frequency/severity; or how different populations with intersecting vulnerabilities have been affected. Moreover, insufficient attention has considered what has worked in the prevention of and response to GBV during the pandemic.

Occupation, Income, and Labour

COVID-19 has caused a global economic recession, with widespread economic insecurity and job losses[34] in high-, middle- and low-income countries. Past evidence from economic crises suggest that men tend to be most affected by the resulting unemployment, as their employment is more sensitive to cyclical downturns[35]. However, due to the unique feature of social distancing and stay-at-home orders, the COVID-19 pandemic has had a greater impact on sectors with high female employment shares[34,36], such as retail, hospitality and informal sectors. Early estimates suggested that women's jobs were 1.8 times more vulnerable to this economic crisis than men's, because although women

made up 54% of overall job losses in the first waves of the pandemic, while they accounted for 39% of global employment[37]. A multi-country study in China, Italy, Japan, South Korea, the United Kingdom, and the United States, found that women were 24% more likely than men to permanently lose their jobs because of the COVID-19 outbreak[38]. The economic impact of the pandemic on women has been compounded by their lower earnings, lower ability to save, and less secure jobs, primarily in the informal sector[25,39].

Globally and across all regions, the burden of unpaid care work falls largely on women, who perform 76% of it, or 3.2 times more than men[40]. This burden increases during crises and disease outbreaks, as seen with the HIV pandemic and the Ebola outbreak, among others[41]. Emerging research suggests that the COVID-19 crisis, lockdown measures, stay-at-home orders, closures of schools and childcare facilities have dramatically increased this burden by reducing the formal and informal care supply[42,43]. In particular, single parents (primarily women) and women working in essential services, including healthcare workers, have had to cope with their paid work and caring for their household members, including home-schooling children, domestic work, and care of the elderly[25]. Data from the United States indicates that the pandemic's impact on women's unpaid care work has been greater in lower-income households and among racial and ethnic minorities[42]. These trends raise serious concerns about women's coping strategies and mental health.

Previous evidence before COVID-19 suggests that such a sharp exposure to care work by men can have long-term effects on their involvement in their household's unpaid care work[44–46]. Some data from high-income countries indicate that during the COVID-19 pandemic, although women have taken on more home-schooling and overall unpaid care work, there are some egalitarian trends with men increasing their relative share of childcare and domestic work[42]. However, more globally representative data and trends are lacking.

The pandemic has accelerated digital transformations in the workplace, and greater reliance on flexible work arrangements. Such a flexible 'future of work' could benefit women's labour force participation in the long run, allowing them to better combine their paid and unpaid care work. However, globally, more men than women have access to and use the internet.[47] This gender digital divide could further affect women's employment and wage gap in increasingly digital workplaces, that require digital skills to either stay on the job or get re-employed (ref).

Social and gender norms

Social and gender norms and inequalities are entrenched in daily life and across several spheres, including employment, education, political participation, and access to health care. They are reflected in both underpaid formal economy care jobs (most teachers and nurses are female), and the unpaid and invisible informal domestic work mostly performed by women[25]. Economic crises, periods of unrest and economic transitions have often reinforced inequitable gender norms in favour of men, with more societal acceptability of norms implying that women have less value and fewer rights than men[41]. They have also exacerbated gender tensions through their negative impacts on male employment, undermining norms of masculinity related to the 'male breadwinner'[48,49], and have been associated with increases in domestic violence[14,41,50] .

The COVID-19 pandemic is limiting and even regressing past decades gains by deepening pre-existing gender inequalities and exposing vulnerabilities in social, political and economic systems[25]. Discriminatory social norms, gender roles and power dynamics appear to have worsened during the pandemic,[51] due to lockdowns and economic insecurity.[52] In addition to women's increased unpaid care burden[43], the loss of employment could lead to greater gender power imbalance, implying less

economic autonomy for women, and an inability to meet social expectations with respect to masculinity for men. This is likely contributing to increases in men's perpetration of violence against women, along with the loss of control resulting from pandemic-related movement restrictions accentuating men's controlling behaviours over women[14].

Gender, stigma and discrimination

Often arising from fear, stigmatisation in the wake of a new illness is far from novel. Historically, social reactions to epidemics, pandemics, and 'plagues' have revealed the perception that these diseases originate elsewhere. Considering the context of those already marginalised, this compounds the risk of them being further alienated and "othered" due to their overlaying vulnerabilities[53]. The effect of gender inequality is exacerbated when it intersects with other social identities like gender identity, sexual orientation, age, race, class, dis/ability, socio-economic status, occupation, and immigration status and practices, such as sex work, drug use, alcohol consumption[54].

The negative effects of COVID-19 are disproportionately borne by the marginalised and vulnerable people and communities[55], whose existing stigma intersects with health-related stigmas. In different parts of the world, people with disabilities and elderly people have been discriminated against in terms of medical care and social norms[55,56]. Certain minority groups, such as the LGBTQIA community, who already face deep-rooted and widespread stigma and discrimination, have experienced further restrictions to their access to resources (health care, education, benefits, housing, social support, amongst others), according to studies from high-income countries[57–62]. In addition, the pandemic has fuelled discrimination against Asian people or Asian descendants in different parts of the world[63]. Such discriminatory behaviours can undermine strategies to mitigate COVID-19 and drive individuals or communities to disengage from the health system by hiding the illness to avoid discrimination, preventing individuals from seeking help, and reject healthy behaviours (such as wearing masks)[64].

Stigma is intersectional and multi-dimensional, and is produced by drivers (such as misinformation) and facilitators (such as inequitable social and gender norms, health policies, institutional practices)[65,66]. For interventions to be effective, they must target both the removal of the drivers of stigma and the shifting of norms and policies which facilitate the stigmatisation process. In the context of COVID-19, stigma reduction must consider the multiple levels and dimension at which stigma is formed and enacted. There has been some research on GBV and other social determinants of health in the context of COVID-19 that has included intersectional analysis. However, it remains limited and mostly correlational [1,67–69], thus limiting the potential of policies and responses to be informed by the experiences of those most marginalised and most affected, and effectively targeted at the most disadvantaged and discriminated in the community.

Education, Social Infrastructure, Structure and Support.

Formal and informal structures of support are threatened during pandemic situations, characterized by reductions in or lack of access to social services and infrastructure, like education, law enforcement, legal services and other social protection/safety nets[14]. As a result of social distancing, quarantine as well as economic strains, frontline organisations delivering vital services may be incapacitated to support women in need[14,51]. In addition, during pandemics, there is often a reallocation of resources towards the pandemic response, at the expense of other women-focused health and social services. For example, law enforcement operations, first responders, crisis hotlines, women's groups and other channels of legal help, crisis support, and financial assistance for women in IPV situations can be threatened[14].

One of the unique and pervasive COVID-19 pandemic measures has been school closures, which have affected over 1.5 billion children worldwide[47]. School closures have been found to reduce the protection that schools provide for girls, both in terms of their health, experience of violence and future learning and economic opportunities[51]. From past outbreaks, violence against girls who are not in school is known to be heightened[51,70]. The potential consequences of prolonged periods out-of-school for girls include school drop-outs, learning gaps, increased burden of unpaid care work, forced marriage, unplanned and early pregnancy, as observed during the last Ebola outbreak in Sierra Leone, for example[51]. The crisis and resulting poverty can drive parents to resort to child marriages for girls and child labour for boys and girls, as negative coping mechanisms. Girls may also be pressured into transactional sex and are at a higher risk of been trafficked[51,70]. School closures also impact access to school meals, school immunisation (like HPV) programmes for girls, and school programmes for sexual and reproductive health services, including menstrual health services.

COVID-19 has also ushered in a new digital world of online education, with distinct gendered risks. Girls have less access to technology and also lower digital literacy. Children in remote and rural areas are particularly disadvantaged for lack of internet and digital devices, but even within households and communities with constrained access, boys' education is likely to be prioritised[47,71].

Environmental determinants of health

Past public health emergencies have found women to be disproportionately exposed to environmental health risks, particularly in developing countries and in humanitarian crisis settings with limited healthcare infrastructure[72]. The COVID-19 pandemic has had expected and unexpected impacts on outdoor and indoor air pollution, and access to water, sanitation and hygiene (WASH), with gender-related implications.

Air pollution exposure significantly increased the risk of dying from SARS during the 2003 outbreak[73], and seems to also increase the likelihood of dying from COVID-19[74]. COVID-19 related lockdowns reduced outdoor air pollution dramatically[75] - one of the indirect positive impacts of the pandemic response with benefits for population health. While evidence on the gendered effect of outdoor air pollution is limited, certain European and Latin American governments are now rethinking their urban settings from a mobility perspective, along with air pollution regulations, to foster public transport and low-pollution transportation[76,77]. Since women are more likely than men to use public transport, this could catalyse more gender-responsive urban spaces and increased mobility for women[78].

In-door air pollution from cooking (mainly undertaken by women) using inefficient polluting cook stoves is likely to increase vulnerability to COVID-19, through the increased risk for co-morbidities, particularly in developing countries[79]. In India, indoor pollution is associated with COPD and lung cancer in women and acute lower respiratory disease in children under 5 years, who stay indoors for longer periods[80].

Furthermore, effective access to water, sanitation and hygiene (WASH) is required to control the spread of infections. As previously observed in humanitarian settings and in low-income countries[81], the COVID-19 pandemic may reduce women's access to WASH due to decreased household income, increased competition for resources and disrupted supply chains. Interrupted water and sanitary supplies services might impact women's ability to meet their own hygienic needs and prevent household infections[72]. In humanitarian settings, women might be forced to travel further to collect

water and obtain essential hygiene materials, increasing their vulnerability to GBV, sexual exploitation and abuse[82]. However, WHO's WASH recommendations to control the COVID-19 pandemic[83] have been challenging to implement in low-income countries[84].

The gendered division of labour determines women's role as primary water purveyors and their responsibility for all water-related tasks, further increasing their unpaid care burden, limiting their access to education, income generation or leisure. Water scarcity and the COVID-related rise in water and soap prices, disproportionally affect women and girls by increasing their domestic workload and time spent to find water[85]. While women bear the main WASH responsibilities, they are absent at decision-making levels. The pandemic has exacerbated existing gendered barriers to WASH access that create unique WASH and menstrual hygiene management (MHM) challenges for women and girls every day[72,82].

2.3 Desired impact of the proposed research on policy, program, and community responses

(to be written after the research prioritisation)

Evidence-informed advocacy and COVID-19 response and recovery policies

- Data to inform and track the gendered impacts and effectiveness of policies, including sex-disaggregated data on the economic impacts, distribution of unpaid care work and GBV among others
- Pro-active policy advocacy at country level
- Contribution to understanding and finding targeted solutions to the gendered burden of COVID-19, and ensuring gender-responsive policies are part of reconstruction efforts.
- Provision of future direction for a sustained effort to address the basic needs (especially protection, water and sanitation, food security, shelter, livelihoods and education) of deprived communities post-COVID.
- Evidence and best practices on how to design and maintain GBV services for survivors at all times.

Social Accountability

- Accountability framework: accountability of decision-makers during the crisis towards key affected populations (healthcare workers, patients, families of deceased and caretakers, affected population)
- Emphasis on intersectional equity and how this approach to the social determinants of health will add value, to determine who is included/not included in the pandemic responses and act consequently.
- Inclusion of all minorities or underrepresented groups, including racialized, Indigenous People, migrants, stateless, internally displaced persons (IDPs), asylum seekers and refugees, transgender, children (especially girls), non-binary, LGTBQIA people and their families, sex workers, , people with functional and mental diversity and any other context-related intersectional vulnerability.
- Moving beyond binary heteronormative/cisnormative thinking about the gendered impacts, to include LGBTQIA and non-binary/gender non-conforming people and their families and considering how they already experience social and economic exclusion that is amplified by the pandemic

Shifts in policies with demonstrated negative gendered impacts

- Policy makers and the public acknowledge the adverse effects of school closures and how it continues to widen the gender gap in education, and therefore prioritise education and schooling in their responses.
- Quantifying the effect of school disruptions on SRH, socioeconomic status and violence against girls would help proper planning to meet the SDGs.

Better gender-responsive preparedness for next pandemics

- Inform international and national guidance on health emergency preparedness and responses to ensure gender inequalities are both addressed as part of the response, and not exacerbated by pandemic response measures.
- Enhanced understanding and knowledge of shifts in gender norms and their determinants during and post-pandemic to learn for subsequent periods of this pandemic and future pandemics.

- Recognise fully the social, economic and human value of care-takers and design care-centered models

2.4 Actors and strategies to implement and promote uptake of the research agenda

- Researchers need to build alliances and partnerships with CSOs, specially grassroots feminist/women/LGTBIQ+/youth movements to implement mixed-methods research and reach grassroots communities.

- Transforming further entrenched gender norms and deeper structural inequalities will require broad-based multi-stakeholder engagement, including policy-makers, implementers, community-based groups, LGTBQI organisations, youth champions, sex workers and trade unions, social media activists, women/feminist groups, migrants, refugee representatives and advocates.

- Consider synergies between members in this initiative that can inform future collaborations, briefs, blogs, and research collaborations, etc.

2.5 Population, contexts, study design/ methodologies *(to be written at the end once research questions are prioritised)*

Population and Context:

- Representative populations of all gender (gender identity, non-binarism, sexual orientation), ages, ableism and social conditions mentioned above.
- Several contexts should be analysed to be representative, and as we live in a global patriarchy with local adaptations, this could mean everywhere.

Design/Methods:

- Research should use an intersectional lens to assess need and leave no one behind.
- Participatory action methods should be incorporated into research designs in which such methods fit to answer the research questions.
- Gender and social impact assessments
- Mixed-method approaches: combining quantitative (samples, surveys, national statistics) with qualitative methods (in-depth interviews, disaggregated focus group discussions)
- Apply different methods to:
    a) identify data gaps;
    b) conduct qualitative research to hear from the people whose experiences are missing from the data;
    c) use these qualitative data to conduct and analyse quantitative data;
    d) produce quantitative data and stories to meet policy-makers' needs.
- Comparative research design to analyse commonalities, differences and trends across regions and globally
- Network analysis and econometric tools can be used in the analysis of occupation, income, and gender
- Extra consideration should be given to safety of participants with adherence to ethical principles.

Data

- Sex-and age-disaggregated data will be needed across themes and levels
- Representative surveys in target countries, including real-time norms-monitoring survey tools
- Data required for environmental health are indoor and outdoor air quality measurements: surveys on the perception of air quality before and after isolation measures.

Section 4. Research questions proposed for prioritisation.

Gender-Based Violence

Prevalence, Incidence, Types & Impacts

1. How has the prevalence, severity and frequency of different types of GBV (including online violence, early marriage) changed during different phases of the COVID-19 pandemic, and has this varied by sub-population?
2. Which women and girls facing intersecting forms of discrimination (including age, poverty, disability, sexuality, etc) were the most vulnerable to and affected by different types of GBV during the pandemic?
3. What were the costs of GBV for survivors, perpetrators and their communities in the context of COVID-19?

Drivers & pathways

4. What were the drivers/determinants and pathways of increased GBV in the context of COVID-19 (including gender norms and stereotypes, norms of masculinity, intersectional stigma and discrimination) ?
5. What were the lived experiences and narratives of GBV survivors and of men who perpetrated GBV during the pandemic?

Policy Responses, interventions and research

6. What was the nature, scale, coverage and financing of policy, institutional and social responses that governments, communities and other stakeholders adopted to prevent GBV and respond to the needs of GBV survivors during the pandemic?
7. What policies, programmes and interventions were successful and most cost-effective in preventing GBV during the COVID-19 pandemic, and over the long-term (including for which target groups and types of GBV)?
8. What challenges, gaps, failures, successes and opportunities did these policy responses reveal?
9. To what extent have women-led organisations and CSOs been involved in the delivery of non-health services for GBV prevention and response during the pandemic?
10. How did government and community responses during the pandemic affect the availability, access and utilization of GBV services by survivors, compared to pre-pandemic levels ?
11. How have interventions that use community mobilisation and peer-to-peer outreach been adapted given social distancing measures, and how did this affect their quality, effectiveness, and cost?
12. How has research on GBV in the context of the pandemic considered violence against LGBTQI persons and other marginalized groups?

Occupation, Income and Labour

Employment

13. Did the rate of job loss and re-employment during the pandemic, and in the medium term, differ by gender and other intersecting social stratifiers (e.g. geographic location, parenthood, race, ethnicity, migrant status, etc.)?
14. How has automation and the use of new digital technologies affected (positively and negatively) the severity of COVID-19 impacts on female labour and unemployment?
15. What was the impact of COVID-19 on women's access to financial and employment interventions?
16. Did the pandemic affect the gender composition of certain labour sectors (such as healthcare) in the short and medium-term?
17. What policies, programmes and interventions effectively promoted more gender-equitable employment outcomes?

Unpaid care work

18. What coping mechanisms have women adopted during the pandemic to manage paid and unpaid work, including domestic chores, caregiving and home-schooling of children?
19. How did the pandemic affect the total and relative time spent by women and men on different types of unpaid domestic and care work?
20. To what extent has the pandemic's impact on unpaid care work influenced women's full employment, partial employment or unemployment?
21. What policies, programmes and interventions effectively promoted more gender-equitable outcomes in relation to unpaid care work?

Economic autonomy, opportunities and support

22. How has COVID-19 impacted women's economic autonomy, and how has this further impacted their autonomy over their bodies, and in decision-making on public issues?
23. How did women and girls' limited access to economic opportunities before the pandemic (including access to education at all levels, technical and specialised training, credit, land, ownership and heritage, business opportunities, IT and technologies, decision-making areas, control over resources) further constrain their economic opportunities and resilience during the pandemic?
24. Did loss of jobs or income motivate women to look for different job opportunities or set up their own small-scale businesses?
25. In what ways have new digital spaces provided more gender-inclusive, diverse and equitable work spaces?

Social protection

26. What was the level of coverage of cash transfers and safety net programmes among women and men during the pandemic, and to what extent did they improve gender-equitable outcomes?

27. How did the pandemic affect the delivery and receipt of pre-pandemic gender-responsive social support or social protection?
28. How gender-responsive were governments' economic and social protection policies and interventions during the pandemic, and how effective were they at reducing gender inequalities (including for women-headed households)?
29. Did the private sector in female-dominated industries (e.g. transnational personal service providers) provide wage protection and safety nets for their workers?
30. To what extent have past gains in key gender equality indicators (female labour market participation, shared domestic responsibilities, child marriages, school drop-outs) been reversed during the pandemic?

Social and gender norms

31. How has the pandemic affected gender norms in different regions and contexts (e.g. redefinition of social norms, appearance of new social representations or stereotypes)?
32. What are the determinants and pathways to the shift in gender norms during and post-pandemic?
33. What steps can be taken to reverse the effect of COVID-19 on women's autonomy (over their bodies, economic autonomy and decision-making in public issues)?
34. What policies, programmes and interventions were effective in promoting gender-equitable norms?

Stigma and discrimination

35. How did the pandemic affect the stigma, discrimination and marginalisation experienced by different groups in different regions and contexts, including LGBTQI+ persons, sex workers, people who use drugs, people living with HIV, persons with disabilities, refugees, asylum seekers, migrants, IDPs, people incarcerated, homeless people (and those at the intersection of these identities)?
36. Were there gender differences in the stigma and discrimination faced by people who tested positive for COVID-19 (especially women, LGBTQIA people and gender-diverse people) and when they were re-joined their families, return to their jobs or their communities?
37. What policies, programmes and interventions were effective in reducing gendered stigma and discrimination during the pandemic?

Education.

38. How did lockdowns and school closures differentially impact girls' and boys' education opportunities, including enrolment, drop-out rates and negative coping mechanisms in response to economic constraints (such as child marriage, child labour)?
39. What are the potential impacts of the COVID-induced digitalization of education on the future of young women and their future families, and how can digital technologies be used to increase

the self-efficacy of girls and make them less vulnerable to harmful gender norms and inequitable gender roles?

40. What policies, programmes and interventions were effective in mitigating the negative gendered impacts of school closures and online schooling?

Environmental health (indoor/outdoor air pollution, WASH)

41. Have social isolation measures affected indoor and outdoor air quality, and if so, how has this impact varied from a gender perspective?
42. How have changes in cleaning practices (e.g. increased use of bleach) affected women's occupational health hazards related to domestic work?
43. What is the impact of COVID-19 pandemic responses on the environmental determinants of health and their gendered manifestations?

Section 5. References

1 Hankivsky O, Kapilashrami A. Beyond sex and gender analysis: an intersectional view of the COVID-19 pandemic outbreak and response. 2020. https://mspgh.unimelb.edu.au/__data/assets/pdf_file/0011/3334889/Policy-brief_v3.pdf (accessed July 3, 2020).

2 Lokot M, Avakyan Y. Intersectionality as a lens to the COVID-19 pandemic: implications for sexual and reproductive health in development and humanitarian contexts. *Sex Reprod Health Matters* 2020; **28**: 1764748.

3 Wenham C, Smith J, Morgan R. COVID-19: the gendered impacts of the outbreak. *The Lancet* 2020; **395**: 846–8.

4 Gender and COVID19 Working Group. Gender & Covid-19 Working Group. Gend. Covid-19. 2020. https://www.genderandcovid-19.org/gender-working-group-page/ (accessed Jan 19, 2021).

5 Solar O, Irwin AA. A conceptual framework for action on the social determinants of health. *Soc Determinants Health Discuss Pap 2 Policy Pract* 2010. http://apps.who.int/iris/bitstream/10665/44489/1/9789241500852_eng.pdf (accessed Feb 22, 2021).

6 Inter-Agency Standing Committee. Guidelines for Integrating Gender-Based Violence Interventions in Humanitarian Action: Reducing risk, promoting resilience and aiding recovery. 2015.

7 United Nations Population Fund. Managing Gender-based Violence Programmes in Emergencies. 2012. /publications/managing-gender-based-violence-programmes-emergencies (accessed Feb 22, 2021).

8 United Nations Women. Frequently asked questions: Types of violence against women and girls. UN Women. https://www.unwomen.org/what-we-do/ending-violence-against-women/faqs/types-of-violence (accessed Feb 22, 2021).

9 Overseas Development Institute. Social norms, gender norms and adolescent girls: a brief guide. 2015. https://www.odi.org/sites/odi.org.uk/files/odi-assets/publications-opinion-files/9818.pdf.

10 Bernard C. Why intersectionality matters for social work practice in adult services - Social work with adults. 2020. https://socialworkwithadults.blog.gov.uk/2020/01/31/why-intersectionality-matters-for-social-work-practice-in-adult-services/ (accessed Feb 22, 2021).

11 MenEngage Alliance. Men, Masculinities, and Changing Power: A Discussion Paper on Engaging Men in Gender Equality From Beijing 1995 to 2015. 2016. https://www.unfpa.org/sites/default/files/resource-pdf/Men-Masculinities-and-Changing-Power-MenEngage-2014.pdf.

12 UN Women. Intersectional feminism: what it means and why it matters right now. UN Women. 2020; published online July 1. https://www.unwomen.org/en/news/stories/2020/6/explainer-intersectional-feminism-what-it-means-and-why-it-matters (accessed March 17, 2021).

13 Devries KM, Mak JYT, García-Moreno C, *et al.* Global health. The global prevalence of intimate partner violence against women. *Science* 2013; **340**: 1527–8.

14 Peterman A, Potts A, O'Donnell M, *et al.* Pandemics and Violence Against Women and Children. *CGD Work Pap 528Washington DC Cent Glob Dev* 2020. https://www.cgdev.org/publication/ pandemics-and-violence-against-women-and-children.

15 Bhalotra S, Kambhampati U, Rawlings S, Siddique Z. Intimate Partner Violence: The Influence of Job Opportunities for Men and Women. *World Bank Econ Rev* 2019; published online Nov 7. DOI:10.1093/wber/lhz030.

16 Anderberg D, Rainer H, Wadsworth J, Wilson T. Unemployment and Domestic Violence: Theory and Evidence. *Econ J* 2016; **126**: 1947–79.

17 Lau JTF, Yang X, Pang E, Tsui HY, Wong E, Wing YK. SARS-related Perceptions in Hong Kong. *Emerg Infect Dis* 2005; **11**: 417–24.

18 Glonti K, Gordeev VS, Goryakin Y, *et al.* A Systematic Review on Health Resilience to Economic Crises. *PLOS ONE* 2015; **10**: e0123117.

19 Wako E, Elliott L, De Jesus S, Zotti ME, Swahn MH, Beltrami J. Conflict, Displacement, and IPV: Findings From Two Congolese Refugee Camps in Rwanda. *Violence Women* 2015; **21**: 1087–101.

20 Falb KL, McCormick MC, Hemenway D, Anfinson K, Silverman JG. Violence against refugee women along the Thai–Burma border. *Int J Gynecol Obstet* 2013; **120**: 279–83.

21 Horn R. Responses to intimate partner violence in Kakuma refugee camp: refugee interactions with agency systems. *Soc Sci Med 1982* 2010; **70**: 160–8.

22 European Institute for Gender Equality (EIGE). Coronavirus puts women in the frontline. Eur. Inst. Gend. Equal. 2020. https://eige.europa.eu/news/coronavirus-puts-women-frontline (accessed Feb 22, 2021).

23 Palermo T, Bleck J, Peterman A. Tip of the Iceberg: Reporting and Gender-Based Violence in Developing Countries. *Am J Epidemiol* 2014; **179**: 602–12.

24 Pereira A, Peterman A, Neijhoft AN, *et al.* Disclosure, reporting and help seeking among child survivors of violence: a cross-country analysis. *BMC Public Health* 2020; **20**: 1051.

25 United Nations. Policy Brief: The Impact of COVID-19 on Women. 2020.

26 The Alliance for Child Protection in Humanitarian Action. Guidance Note: Protection of children during infectious disease outbreaks. Guid. Note Prot. Child. Infect. Dis. Outbreaks. 2018; published online July 21. https://alliancecpha.org/en/child-protection-online-library/guidance-note-protection-children-during-infectious-disease (accessed March 11, 2021).

27 Kostelny K, Lamin D, Manyeh M, *et al.* 'Worse than the War': An ethnographic study of the impact of the Ebola crisis on life, sex, teenage pregnancy, and a community-driven intervention in rural Sierra Leone. Resour. Cent. 2018; published online Sept 12. https://resourcecentre.savethechildren.net/library/worse-war-ethnographic-study-impact-ebola-crisis-life-sex-teenage-pregnancy-and-community (accessed March 11, 2021).

28 Holt K, Ratcliffe R. Ebola vaccine offered in exchange for sex, Congo taskforce meeting told. The Guardian. 2019; published online Feb 12. http://www.theguardian.com/global-development/2019/feb/12/ebola-vaccine-offered-in-exchange-for-sex-say-women-in-congo-drc (accessed March 11, 2021).

29 Kerr-Wilson A, Gibbs A, McAslan Fraser E, *et al.* What Works to prevent violence against women and girls? A rigorous global evidence review of interventions to prevent violence against women and girls. Rigorous Glob. Evid. Rev. Interv. Prev. Violence Women Girls. 2020; published online Feb 19. https://www.whatworks.co.za/resources/evidence-reviews/item/693-a-rigorous-global-evidence-review-of-interventions-to-prevent-violence-against-women-and-girls (accessed March 11, 2021).

30 WHO. WHO | RESPECT women: preventing violence against women. WHO. 2019; published online May 29. http://www.who.int/reproductivehealth/topics/violence/respect-women-framework/en/ (accessed March 11, 2021).

31 Peterman A, O'Donnell M. COVID-19 and Violence against Women and Children: A Third Research Round Up for the 16 Days of Activism. *Domest Violence* 2020; : 12.

32 Peterman A, O'Donnell M, Palermo T. COVID-19 and violence against women and children. what have we learned so far. *Wash DC Cent Glob Dev* 2020.

33 National Commission on COVID-19 and Criminal Justice. NCCCJ - Impact Report: COVID-19 and Domestic Violence Trends. Natl. Comm. COVID-19 Crim. Justice. 2021; published online Feb 23. https://covid19.counciloncj.org/2021/02/23/impact-report-covid-19-and-domestic-violence-trends/ (accessed April 12, 2021).

34 ILO. ILO Monitor: COVID-19 and the world of work. Fifth edition. 2020; published online June 30. https://www.ilo.org/wcmsp5/groups/public/---dgreports/---dcomm/documents/briefingnote/wcms_749399.pdf (accessed March 11, 2021).

35 Sabarwal S, Sinha N, Buvinic M. How Do Women Weather Economic Shocks? A Review of the Evidence. Rochester, NY: Social Science Research Network, 2010 https://papers.ssrn.com/abstract=1722009 (accessed March 11, 2021).

36 Alon T, Doepke M, Olmstead-Rumsey J, Tertilt M. The Impact of COVID-19 on Gender Equality. Cambridge, MA: National Bureau of Economic Research, 2020.

37 Madgavkar A, White O, Krishnan M, Mahajan D, Azcue X. COVID-19 and gender equality: Countering the regressive effects. COVID-19 Gend. Equal. Countering Regressive Eff. 2020; published online July 15. https://www.mckinsey.com/featured-insights/future-of-work/covid-19-and-gender-equality-countering-the-regressive-effects (accessed March 11, 2021).

38 Dang H-AH, Viet Nguyen C. Gender inequality during the COVID-19 pandemic: Income, expenditure, savings, and job loss. *World Dev* 2021; **140**: 105296.

39 Vijayasingham L, Govender V, Witter S, Remme M. Employment based health financing does not support gender equity in universal health coverage. *BMJ* 2020; **371**: m3384.

40 Charmes J. The Unpaid Care Work and the Labour Market. An analysis of time use data based on the latest World Compilation of Time-use Surveys. Geneva, 2019 http://www.ilo.org/gender/Informationresources/Publications/WCMS_732791/lang--en/index.htm (accessed March 11, 2021).

41 Seguino S. Plus Ça Change? Evidence on Global Trends in Gender Norms and Stereotypes. *Fem Econ* 2007; **13**: 1–28.

42 Dugarova E. Unpaid care work in times of the COVID-19 crisis: Gendered impacts, emerging evidence and promising policy responses. *UN* 2020; : 19.

43 Power K. The COVID-19 pandemic has increased the care burden of women and families. *Sustain Sci Pract Policy* 2020; **16**: 67–73.

44 Farré L, González L. Does paternity leave reduce fertility? *J Public Econ* 2019; **172**: 52–66.

45 OECD. Women at the core of the fight against COVID-19 crisis. OECD. 2020; published online April 1. https://www.oecd.org/coronavirus/policy-responses/women-at-the-core-of-the-fight-against-covid-19-crisis-553a8269/ (accessed March 11, 2021).

46 Tamm M. Fathers' parental leave-taking, childcare involvement and labor market participation. *Labour Econ* 2019; **59**: 184–97.

47 UNESCO. Addressing the gender dimensions of COVID-related school closures - UNESCO Digital Library. 2020. https://unesdoc.unesco.org/ark:/48223/pf0000373379 (accessed Feb 22, 2021).

48 Chant S. ¿Crisis de la familia? ¿Crisis de la masculinidad? Reflexiones sobre las masculinidades, el trabajo y la familia en el noroeste de Costa Rica. [Crisis in the Family? Crisis in Masculinity? Reflections on Masculinities, Work, and Family in Northwestern Costa Rica]. In: Rodríguez E, ed. Un siglo de luchas

femeninas en América Latina. San Jose, Costa Rica: Universidad de Costa Rica, 2002. https://core.ac.uk/display/210915 (accessed March 11, 2021).

49 Kabeer N. The Power to Choose: Bangladeshi Women and Labour Market Decisions in London and Dhaka, New edition. London and New York: Verso, 2002.

50 Asian Development Bank, UN Women. Gender Equality and the Sustainable Development Goals in Asia and the Pacific: Baseline and Pathways for Transformative Change by 2030. Asian Development Bank, 2018 https://www.adb.org/publications/gender-equality-sdgs-asia-pacific (accessed March 11, 2021).

51 Akmal M, Hares S, O'Donnell M. Gendered Impacts of COVID-19 School Closures: Insights from Frontline Organizations. *CGD Policy Pap 175* 2020; : 23.

52 Align Platform. Gender norms and the coronavirus. Align Platf. 2020. https://www.alignplatform.org/gender-norms-and-coronavirus (accessed Feb 22, 2021).

53 Logie CH, Turan JM. How Do We Balance Tensions Between COVID-19 Public Health Responses and Stigma Mitigation? Learning from HIV Research. *AIDS Behav* 2020; **24**: 2003–6.

54 Heller L. Gender Equality in the human rights to water and sanitation. 2016. https://www.ohchr.org/Documents/Issues/Water/Pamplet_GenderEquality.pdf.

55 Harper C. Gender norms, intersectionality and a COVID-19 'global reset' > Gender & Covid-19. Gend. Covid-19. 2020. https://www.genderandcovid-19.org/norms/gender-norms-intersectionality-and-a-covid-19-global-reset/ (accessed Feb 23, 2021).

56 Soto-Perez-de-Celis E. Social media, ageism, and older adults during the COVID-19 pandemic. *EClinicalMedicine* 2020; **29**: 100634.

57 Graeme Reid. LGBTQ Inequality and Vulnerability in the Pandemic. Hum. Rights Watch. 2020; published online June 18. https://www.hrw.org/news/2020/06/18/lgbtq-inequality-and-vulnerability-pandemic (accessed March 11, 2021).

58 Hetherly M. COVID while gay: 'Horrificness' for an already marginalized community. 2020; published online Oct 19. https://news.wbfo.org/post/covid-while-gay-horrificness-already-marginalized-community (accessed March 11, 2021).

59 Banerjee D, Nair VS. "The Untold Side of COVID-19": Struggle and Perspectives of the Sexual Minorities. *J Psychosexual Health* 2020; **2**: 113–20.

60 Gibb JK, DuBois LZ, Williams S, McKerracher L, Juster R, Fields J. Sexual and gender minority health vulnerabilities during the COVID-19 health crisis. *Am J Hum Biol* 2020; **32**. DOI:10.1002/ajhb.23499.

61 Salerno JP, Williams ND, Gattamorta KA. LGBTQ populations: Psychologically vulnerable communities in the COVID-19 pandemic. *Psychol Trauma Theory Res Pract Policy* 2020; **12**: S239–42.

62 Santos G-M, Ackerman B, Rao A, *et al.* Economic, Mental Health, HIV Prevention and HIV Treatment Impacts of COVID-19 and the COVID-19 Response on a Global Sample of Cisgender Gay Men and Other Men Who Have Sex with Men. *AIDS Behav* 2021; **25**: 311–21.

63 Chen JA, Zhang E, Liu CH. Potential Impact of COVID-19-Related Racial Discrimination on the Health of Asian Americans. *Am J Public Health* 2020; **110**: 1624–7.

64 Turner-Musa J, Ajayi O, Kemp L. Examining Social Determinants of Health, Stigma, and COVID-19 Disparities. *Healthcare* 2020; **8**: 168.

65 Stangl AL, Earnshaw VA, Logie CH, *et al.* The Health Stigma and Discrimination Framework: a global, crosscutting framework to inform research, intervention development, and policy on health-related stigmas. *BMC Med* 2019; **17**: 31.

66 Logie CH. Lessons learned from HIV can inform our approach to COVID-19 stigma. *J Int AIDS Soc* 2020; **23**: e25504.

67 Dasgupta J, Schaaf M, Contractor SQ, *et al.* Axes of alienation: applying an intersectional lens on the social contract during the pandemic response to protect sexual and reproductive rights and health. *Int J Equity Health* 2020; **19**: 130.

68 Nazilla Khanlou, Andrew Ssawe, Yvonne Bohr, *et al.* COVID-19 pandemic guidelines for mental health support of racialized women at risk of gender-based violence. *CIHR Knowl Synth Grant* 2020; : 52.

69 Maestripieri L. The Covid-19 Pandemics: why Intersectionality Matters. *Front Sociol* 2021; **6**. DOI:10.3389/fsoc.2021.642662.

70 Burzynska K, Contreras G. Gendered effects of school closures during the COVID-19 pandemic. *The Lancet* 2020; **395**: 1968.

71 Williams E. Digital education poverty in the time of Covid-19. 2020; : 10.

72 Fuhrman S, Kalyanpur A, Friedman S, Tran NT. Gendered implications of the COVID-19 pandemic for policies and programmes in humanitarian settings. *BMJ Glob Health* 2020; **5**: e002624.

73 Cui Y, Zhang Z-F, Froines J, *et al.* Air pollution and case fatality of SARS in the People’s Republic of China: an ecologic study. *Environ Health* 2003; **2**: 15.

74 Wu X, Nethery RC, Sabath BM, Braun D, Dominici F. Exposure to air pollution and COVID-19 mortality in the United States: A nationwide cross-sectional study. *medRxiv* 2020; published online April 7. DOI:10.1101/2020.04.05.20054502.

75 Liu F, Wang M, Zheng M. Effects of COVID-19 lockdown on global air quality and health. *Sci Total Environ* 2021; **755**: 142533.

76 Manisalidis I, Stavropoulou E, Stavropoulos A, Bezirtzoglou E. Environmental and Health Impacts of Air Pollution: A Review. *Front Public Health* 2020; **8**. DOI:10.3389/fpubh.2020.00014.

77 Ollivier G, Gupta N. Rethinking India’s public transport after the COVID-19 lockdown is over. World Bank Blogs. 2020; published online May 26. https://blogs.worldbank.org/endpovertyinsouthasia/rethinking-indias-public-transport-after-covid-19-lockdown-over (accessed March 17, 2021).

78 Duchene C. Gender and Transport. 2011; published online April 1. DOI:10.1787/5kg9mq47w59w-en.

79 WHO. Burden of Disease from Ambient and Household Air Pollution. Ambient Househ. Air Pollut. Health. http://www.who.int/phe/health_topics/outdoorair/databases/en/ (accessed March 11, 2021).

80 Dherani M, Pope D, Mascarenhas M, Smith KR, Weber M, Bruce N. Indoor air pollution from unprocessed solid fuel use and pneumonia risk in children aged under five years: a systematic review and meta-analysis. *Bull World Health Organ* 2008; **86**: 390–8.

81 VanLeeuwen C, Torondel B. Improving menstrual hygiene management in emergency contexts: literature review of current perspectives. *Int J Womens Health* 2018; **10**: 169–86.

82 Sommer M, Ferron S, Cavill S, House S. Violence, gender and WASH: spurring action on a complex, under-documented and sensitive topic. *Environ Urban* 2015; **27**: 105–16.

83 WHO. Water, sanitation, hygiene, and waste management for SARS-CoV-2, the virus that causes COVID-19. 2020; published online July 29. https://www.who.int/publications/i/item/WHO-2019-nCoV-IPC-WASH-2020.4 (accessed March 11, 2021).

84 Donde OO, Atoni E, Muia AW, Yillia PT. COVID-19 pandemic: Water, sanitation and hygiene (WASH) as a critical control measure remains a major challenge in low-income countries. *Water Res* 2021; **191**: 116793.

85 Benini D. The gendered impacts of the COVID-19 pandemic on women and girls. WASH Matters. 2021; published online Jan 18. https://washmatters.wateraid.org/blog/the-gendered-impacts-of-the-covid-19-pandemic-on-women-and-girls (accessed March 17, 2021).

Section 6. Resources shared

To be completed

Section 7: Results of research questions prioritised

Table 6: High Priority Gender and COVID-19 Research Questions for all criteria

RQ	Label	Public Health	Gender equality	Urgency for policy
RQ1	How has the prevalence, incidence, severity and frequency of different types of GBV (including online violence, and child marriage) changed during the different phases of the COVID-19 pandemic	**X**	X	X
RQ2	Which women and girls facing intersecting forms of discrimination (including age, poverty, disability, and sexuality, etc) are the most vulnerable to, and affected by, different types of GBV during the pandemic	X	X	X
RQ4	What are the determinants and pathways of increased GBV in the context of COVID-19	**X**	X	
RQ7	What policies, programmes and interventions have been successful and most cost-effective in preventing GBV during the pandemic, and over the long-term	**X**	X	
RQ18	What coping mechanisms have women adopted during the pandemic to manage paid and unpaid work, including domestic chores, caregiving and the home-schooling of children		X	X
RQ35	How has the pandemic affected the stigma, discrimination and marginalization experienced by different groups in various contexts, including LGBTQI+ persons, sex workers, people who use drugs, people living with HIV, persons with disabilities, refugees, migrant status, people incarcerated, homeless people and those at the intersection of these identities?	**X**		X
RQ33	What steps can be taken to reverse the effect of COVID-19 on womenâ€™s autonomy (over their bodies, their economic condition and their decision-making on public issues)	**X**		
RQ38	How have lockdowns and school closures differentially impacted girlsâ€™ and boysâ€™ educational opportunities, including enrolment, drop-out rates and negative coping mechanisms (such as child marriage, and child labour)		X	
RQ20	What policies, programmes and interventions have effectively promoted more gender-equitable outcomes in relation to unpaid care work during the pandemic		X	
RQ34	What policies, programmes and interventions are effective in promoting gender-equitable norms during the pandemic		X	

RQ14	What is the impact of COVID-19 on womenâ€™s access to financial and employment interventions		X	
RQ16	What policies, programmes and interventions have effectively promoted more gender-equitable employment outcomes during the pandemic		X	
RQ17	How has the pandemic affected the total and relative time spent by women and men on different types of unpaid domestic and care work		X	
RQ19	To what extent has the pandemic's impact on unpaid care work influenced women's full time employment, partial employment or unemployment		X	
RQ34	What policies, programmes and interventions are effective in promoting gender-equitable norms during the pandemic		X	
RQ25	What has the level of coverage of cash transfers and safety net programmes been among women and men during the pandemic			X
RQ26	How has COVID-19 affected the delivery and receipt of pre-pandemic gender-responsive social support or social protection			X

Supplementary Table A: Sample size, Means, Standard deviations and 95% Confidence Intervals for Gender and COVID-19 Research Questions by Public Health Benefit

RQ	Label	N	Mean	SD	95% CI
RQ1	How has the prevalence, incidence, severity and frequency of different types of GBV (including online violence, and child marriage) changed during the different phases of the COVID-19 pandemic	**81**	**3.63**	**0.62**	**3.61-3.64**
RQ4	What are the determinants and pathways of increased GBV in the context of COVID-19	**77**	**3.56**	**0.62**	**3.54-3.57**
RQ7	What policies, programmes and interventions have been successful and most cost-effective in preventing GBV during the pandemic, and over the long-term	**79**	**3.56**	**0.64**	**3.54-3.57**
RQ2	Which women and girls facing intersecting forms of discrimination (including age, poverty, disability, and sexuality, etc) are the most vulnerable to, and affected by, different types of GBV during the pandemic	**76**	**3.55**	**0.62**	**3.54-3.57**
RQ35	How has the pandemic affected the stigma, discrimination and marginalization experienced by different groups in various contexts, including LGBTQI+ persons, sex workers, people who use drugs, people living with HIV, persons with disabilities, refugees, migrant status, people incarcerated, homeless people and those at the intersection of these identities?	**69**	**3.51**	**0.66**	**3.49-3.53**
RQ33	What steps can be taken to reverse the effect of COVID-19 on women's autonomy (over their bodies, their economic condition and their decision-making on public issues)	**66**	**3.48**	**0.59**	**3.47-3.50**
RQ38	How have lockdowns and school closures differentially impacted girls' and boys' educational opportunities, including enrolment, drop-out rates and negative coping mechanisms (such as child marriage, and child labour)	**71**	**3.42**	**0.67**	**3.40-3.44**
RQ34	What policies, programmes and interventions are effective in promoting gender-equitable norms during the pandemic	**70**	**3.40**	**0.65**	**3.38-3.42**
RQ10	How have interventions that use community mobilisation and peer-to-peer outreach to address GBV been adapted given social distancing measures, and how did this affect their quality, effectiveness, and cost	**73**	**3.38**	**0.70**	**3.36-3.40**
RQ22	How has women and girls' limited access to resources before COVID-19 further constrained their economic opportunities and resilience during the pandemic	**69**	**3.38**	**0.75**	**3.36-3.40**
RQ17	How has the pandemic affected the total and relative time spent by women and men on different types of unpaid domestic and care work	**70**	**3.37**	**0.71**	**3.35-3.39**
RQ18	What coping mechanisms have women adopted during the pandemic to manage paid and unpaid work, including domestic chores, caregiving and the home-schooling of children	**70**	**3.37**	**0.71**	**3.35-3.39**
RQ16	What policies, programmes and interventions have effectively promoted more gender-equitable employment outcomes during the pandemic	**70**	**3.36**	**0.68**	**3.34-3.38**
RQ14	What is the impact of COVID-19 on women's access to financial and employment interventions	**73**	**3.36**	**0.82**	**3.33-3.38**
RQ6	What is the nature, scale, coverage and financing of responses that governments, communities and other stakeholders have adopted to prevent GBV and respond to the needs of GBV survivors during the pandemic	**76**	**3.36**	**0.74**	**3.34-3.37**
RQ9	How have government and community responses during the pandemic affected the availability, access and utilization of GBV services by survivors, when compared to pre-pandemic levels	**76**	**3.36**	**0.78**	**3.34-3.38**

RQ3	What are the costs of GBV for survivors, perpetrators and their communities in the context of COVID-19	**76**	**3.34**	**0.66**	**3.32-3.36**
RQ19	To what extent has the pandemic's impact on unpaid care work influenced women's full time employment, partial employment or unemployment	**69**	**3.33**	**0.76**	**3.31-3.35**
RQ20	What policies, programmes and interventions have effectively promoted more gender-equitable outcomes in relation to unpaid care work during the pandemic	**69**	**3.33**	**0.70**	**3.31-3.35**
RQ37	What policies, programmes and interventions are effective in reducing gendered stigma and discrimination during the pandemic	**67**	**3.33**	**0.77**	**3.31-3.35**
RQ40	What policies, programmes and interventions are effective in mitigating the negative gendered impacts of school closures and online schooling	**71**	**3.31**	**0.75**	**3.29-3.33**
RQ12	Has the rate of job loss and re-employment during the pandemic, and in the medium term, differed by gender and other forms of social inequality (e.g. geographic location, parenthood, race, ethnicity, and migrant status, etc.)	**74**	**3.30**	**0.77**	**3.28-3.32**
RQ32	How can digital technologies be used to increase the self-efficacy of girls and make them less vulnerable to harmful gender norms and inequitable gender roles	**71**	**3.30**	**0.74**	**3.28-3.32**
RQ21	How has COVID-19 impacted women's economic autonomy, and how has this further impacted their autonomy over their bodies, and in decision-making on public issues	**71**	**3.27**	**0.74**	**3.25-3.29**
RQ36	Are there gender differences in the stigma and discrimination faced by people who test positive for COVID-19 (especially women, LGBTQI+ and gender-diverse people) when they re-join their families, return to their jobs or their communities	**68**	**3.26**	**0.70**	**3.24-3.29**
RQ43	What is the impact of COVID-19 responses on the environmental determinants of health and their gendered manifestations	**68**	**3.25**	**0.76**	**3.23-3.27**
RQ27	How gender-responsive have governments' economic and social protection policies and interventions been during the pandemic, and how effective were they at reducing gender inequalities	**68**	**3.24**	**0.77**	**3.21-3.26**
RQ5	What are the lived experiences and narratives of GBV survivors and of men who perpetrate GBV during the pandemic	**77**	**3.23**	**0.76**	**3.21-3.25**
RQ30	How has the pandemic affected gender norms in different regions and contexts	**70**	**3.21**	**0.63**	**3.20-3.23**
RQ29	To what extent have past gains in key gender equality indicators (such as female labour market participation, shared domestic responsibilities, child marriages, and school drop-outs) been reversed during the pandemic	**67**	**3.21**	**0.84**	**3.18-3.23**
RQ26	How has COVID-19 affected the delivery and receipt of pre-pandemic gender-responsive social support or social protection	**66**	**3.20**	**0.77**	**3.17-3.22**
RQ13	How has automation and the use of new digital technologies affected (positively and negatively) the severity of COVID-19 impacts on female labour and unemployment	**70**	**3.19**	**0.77**	**3.16-3.21**
RQ24	In what ways have new digital technologies provided more gender-inclusive, diverse and equitable work spaces during the pandemic	**66**	**3.18**	**0.82**	**3.16-3.21**
RQ42	How have changes in cleaning practices (e.g. increased use of bleach) affected occupational health hazards due to sex and gender	**65**	**3.17**	**0.80**	**3.15-3.19**

RQ39	What are the potential impacts of the COVID-19-induced digitalization of education on young women's learning and employment opportunities,:The potential health benefit of answering this question is�	**73**	**3.15**	**0.81**	**3.13-317**
RQ11	How has research on GBV in the context of the pandemic considered violence against LGBTQI+ persons and other marginalized groups	**70**	**3.14**	**0.75**	**3.12-3.16**
RQ31	What are the determinants and pathways of changes in gender norms during and post pandemic	**69**	**3.12**	**0.74**	**3.09-3.14**
RQ8	To what extent have women-led organisations and civil society organisations (CSOs) been involved in the delivery of non-health services for GBV prevention and response during the pandemic	**75**	**3.09**	**0.74**	**3.07-3.11**
RQ15	Has the pandemic affected the gender composition of certain labour sectors (such as healthcare) in the short and medium-term	**68**	**3.03**	**0.86**	**3.00-3.05**
RQ25	What has the level of coverage of cash transfers and safety net programmes been among women and men during the pandemic	**65**	**3.00**	**0.83**	**2.97-3.03**
RQ23	Has the loss of jobs and income led to women starting different earning opportunities, or to set up their own small-scale businesses during the pandemic	**69**	**2.99**	**0.83**	**2.96-3.01**
RQ41	Have social isolation measures affected indoor and outdoor air quality, and if so, how has this impact varied from a gender perspective	**67**	**2.97**	**0.80**	**2.95-2.99**
RQ28	Has the private sector in female-dominated industries (e.g. transnational personal service providers) provided wage protection and safety nets for their workers during the pandemic	**65**	**2.95**	**0.78**	**2.93-2.98**

Supplementary Table B: Sample size, Means, Standard deviations and 95% Confidence Intervals for Gender and COVID-19 Research Questions by Gender Equality

RQ	Label	N	Mean	N	95% CI
RQ4	What are the determinants and pathways of increased GBV in the context of COVID-19	**80**	**3.43**	**0.81**	**3.41-3.44**
RQ2	Which women and girls facing intersecting forms of discrimination (including age, poverty, disability, and sexuality, etc) are the most vulnerable to, and affected by, different types of GBV during the pandemic	**81**	**3.42**	**0.74**	**3.40-3.44**
RQ7	What policies, programmes and interventions have been successful and most cost-effective in preventing GBV during the pandemic, and over the long-term	**79**	**3.42**	**0.76**	**3.40-3.44**
RQ38	How have lockdowns and school closures differentially impacted girls' and boys' educational opportunities, including enrolment, drop-out rates and negative coping mechanisms (such as child marriage, and child labour)	**73**	**3.41**	**0.80**	**3.39-3.43**
RQ1	How has the prevalence, incidence, severity and frequency of different types of GBV (including online violence, and child marriage) changed during the different phases of the COVID-19 pandemic	**80**	**3.40**	**0.81**	**3.38-3.42**
RQ20	What policies, programmes and interventions have effectively promoted more gender-equitable outcomes in relation to unpaid care work during the pandemic	**73**	**3.40**	**0.72**	**3.38-3.42**
RQ34	What policies, programmes and interventions are effective in promoting gender-equitable norms during the pandemic	**72**	**3.31**	**0.82**	**3.28-3.33**
RQ14	What is the impact of COVID-19 on women's access to financial and employment interventions	**76**	**3.29**	**0.80**	**3.27-3.31**
RQ16	What policies, programmes and interventions have effectively promoted more gender-equitable employment outcomes during the pandemic	**73**	**3.29**	**0.81**	**3.27-3.31**
RQ17	How has the pandemic affected the total and relative time spent by women and men on different types of unpaid domestic and care work	**73**	**3.29**	**0.77**	**3.27-3.31**
RQ19	To what extent has the pandemic's impact on unpaid care work influenced women's full time employment, partial employment or unemployment	**71**	**3.28**	**0.80**	**3.26-3.30**
RQ18	What coping mechanisms have women adopted during the pandemic to manage paid and unpaid work, including domestic chores, caregiving and the home-schooling of children	**73**	**3.27**	**0.79**	**3.25-3.30**
RQ6	What is the nature, scale, coverage and financing of responses that governments, communities and other stakeholders have adopted to prevent GBV and respond to the needs of GBV survivors during the pandemic	**78**	**3.24**	**0.76**	**3.22-3.26**
RQ5	What are the lived experiences and narratives of GBV survivors and of men who perpetrate GBV during the pandemic	**80**	**3.23**	**0.78**	**3.21-3.24**
RQ12	Has the rate of job loss and re-employment during the pandemic, and in the medium term, differed by gender and other forms of social inequality (e.g. geographic location, parenthood, race, ethnicity, and migrant status, etc.)	**76**	**3.22**	**0.81**	**3.20-3.24**
RQ32	How can digital technologies be used to increase the self-efficacy of girls and make them less vulnerable to harmful gender norms and inequitable gender roles	**73**	**3.22**	**0.84**	**3.20-3.24**
RQ3	What are the costs of GBV for survivors, perpetrators and their communities in the context of COVID-19	**79**	**3.22**	**0.81**	**3.20-3.24**
RQ29	To what extent have past gains in key gender equality indicators (such as female labour market participation, shared domestic responsibilities, child marriages, and school drop-outs) been reversed during the pandemic	**68**	**3.19**	**0.82**	**3.17-3.21**
RQ40	What policies, programmes and interventions are effective in mitigating the negative gendered impacts of school closures and online schooling	**74**	**3.19**	**0.82**	**3.17-3.21**

RQ30	How has the pandemic affected gender norms in different regions and contexts	**72**	**3.18**	**0.83**	**3.16-3.20**
RQ9	How have government and community responses during the pandemic affected the availability, access and utilization of GBV services by survivors, when compared to pre-pandemic levels	**78**	**3.18**	**0.80**	**3.16-3.20**
RQ37	What policies, programmes and interventions are effective in reducing gendered stigma and discrimination during the pandemic	**71**	**3.17**	**0.91**	**3.14-3.19**
RQ33	What steps can be taken to reverse the effect of COVID-19 on women's autonomy (over their bodies, their economic condition and their decision-making on public issues)	**72**	**3.17**	**0.84**	**3.14-3.19**
RQ21	How has COVID-19 impacted women's economic autonomy, and how has this further impacted their autonomy over their bodies, and in decision-making on public issues	**73**	**3.16**	**0.94**	**3.14-3.19**
RQ27	How gender-responsive have governments' economic and social protection policies and interventions been during the pandemic, and how effective were they at reducing gender inequalities	**73**	**3.16**	**0.88**	**3.14-3.19**
RQ35	How has the pandemic affected the stigma, discrimination and marginalization experienced by different groups in various contexts, including LGBTQI+ persons, sex workers, people who use drugs, people living with HIV, persons with disabilities, refugees, migrant status, people incarcerated, homeless people and those at the intersection of these identities?	**70**	**3.16**	**0.96**	**3.13-3.18**
RQ22	How has women and girls' limited access to resources before COVID-19 further constrained their economic opportunities and resilience during the pandemic	**72**	**3.15**	**0.87**	**3.13-3.18**
RQ39	What are the potential impacts of the COVID-19-induced digitalization of education on young women's learning and employment opportunities,:The potential health benefit of answering this question is�	**74**	**3.15**	**0.79**	**3.13-3.17**
RQ10	How have interventions that use community mobilisation and peer-to-peer outreach to address GBV been adapted given social distancing measures, and how did this affect their quality, effectiveness, and cost	**77**	**3.13**	**0.86**	**3.11-3.15**
RQ8	To what extent have women-led organisations and civil society organisations (CSOs) been involved in the delivery of non-health services for GBV prevention and response during the pandemic	**78**	**3.09**	**0.76**	**3.07-3.11**
RQ36	Are there gender differences in the stigma and discrimination faced by people who test positive for COVID-19 (especially women, LGBTQI+ and gender-diverse people) when they re-join their families, return to their jobs or their communities	**70**	**3.09**	**0.91**	**3.06-3.11**
RQ31	What are the determinants and pathways of changes in gender norms during and post pandemic	**72**	**3.08**	**0.83**	**3.06-3.11**
RQ26	How has COVID-19 affected the delivery and receipt of pre-pandemic gender-responsive social support or social protection	**69**	**3.07**	**0.77**	**3.05-3.09**
RQ13	How has automation and the use of new digital technologies affected (positively and negatively) the severity of COVID-19 impacts on female labour and unemployment	**74**	**3.03**	**0.83**	**3.01-3.05**
RQ24	In what ways have new digital technologies provided more gender-inclusive, diverse and equitable work spaces during the pandemic	**74**	**3.03**	**0.88**	**3.00-3.05**
RQ11	How has research on GBV in the context of the pandemic considered violence against LGBTQI+ persons and other marginalized groups	**74**	**2.97**	**0.88**	**2.95-3.00**
RQ15	Has the pandemic affected the gender composition of certain labour sectors (such as healthcare) in the short and medium-term	**74**	**2.91**	**0.94**	**2.88-2.93**
RQ23	Has the loss of jobs and income led to women starting different earning opportunities, or to set up their own small-scale businesses during the pandemic	**72**	**2.88**	**0.79**	**2.85-2.90**

RQ43	What is the impact of COVID-19 responses on the environmental determinants of health and their gendered manifestations	**69**	**2.86**	**0.96**	**2.83-2.88**
RQ25	What has the level of coverage of cash transfers and safety net programmes been among women and men during the pandemic	**67**	**2.85**	**0.89**	**2.82-2.88**
RQ28	Has the private sector in female-dominated industries (e.g. transnational personal service providers) provided wage protection and safety nets for their workers during the pandemic	**70**	**2.80**	**0.84**	**2.78-2.82**
RQ42	How have changes in cleaning practices (e.g. increased use of bleach) affected occupational health hazards due to sex and gender	**69**	**2.57**	**0.99**	**2.54-2.59**
RQ41	Have social isolation measures affected indoor and outdoor air quality, and if so, how has this impact varied from a gender perspective	**69**	**2.54**	**0.92**	**2.51-2.56**

Gender & Health Hub

Supplementary Table C: Sample size, Means, Standard deviations and 95% Confidence Intervals for Gender and COVID-19 Research Questions by Urgency

RQ	Label	N	Mean	SD	95% CI
RQ1	How has the prevalence, incidence, severity and frequency of different types of GBV (including online violence, and child marriage) changed during the different phases of the COVID-19 pandemic	**81**	**2.43**	**0.77**	**2.41-2.45**
RQ18	What coping mechanisms have women adopted during the pandemic to manage paid and unpaid work, including domestic chores, caregiving and the home-schooling of children	**74**	**2.35**	**0.69**	**2.33-2.37**
RQ2	Which women and girls facing intersecting forms of discrimination (including age, poverty, disability, and sexuality, etc) are the most vulnerable to, and affected by, different types of GBV during the pandemic	**79**	**2.33**	**0.78**	**2.31-2.35**
RQ35	How has the pandemic affected the stigma, discrimination and marginalization experienced by different groups in various contexts, including LGBTQI+ persons, sex workers, people who use drugs, people living with HIV, persons with disabilities, refugees, migrant status, people incarcerated, homeless people and those at the intersection of these identities?	**69**	**2.30**	**0.79**	**2.28-2.33**
RQ25	What has the level of coverage of cash transfers and safety net programmes been among women and men during the pandemic	**64**	**2.30**	**0.73**	**2.27-2.32**
RQ26	How has COVID-19 affected the delivery and receipt of pre-pandemic gender-responsive social support or social protection	**66**	**2.27**	**0.78**	**2.25-2.30**
RQ4	What are the determinants and pathways of increased GBV in the context of COVID-19	**77**	**2.25**	**0.80**	**2.23-2.27**
RQ17	How has the pandemic affected the total and relative time spent by women and men on different types of unpaid domestic and care work	**74**	**2.24**	**0.72**	**2.22-2.26**
RQ38	How have lockdowns and school closures differentially impacted girls' and boys' educational opportunities, including enrolment, drop-out rates and negative coping mechanisms (such as child marriage, and child labour)	**72**	**2.24**	**0.74**	**2.22-2.26**
RQ6	What is the nature, scale, coverage and financing of responses that governments, communities and other stakeholders have adopted to prevent GBV and respond to the needs of GBV survivors during the pandemic	**78**	**2.23**	**0.80**	**2.21-2.25**
RQ27	How gender-responsive have governments' economic and social protection policies and interventions been during the pandemic, and how effective were they at reducing gender inequalities	**70**	**2.21**	**0.72**	**2.19-2.23**
RQ36	Are there gender differences in the stigma and discrimination faced by people who test positive for COVID-19 (especially women, LGBTQI+ and gender-diverse people) when they re-join their families, return to their jobs or their communities	**67**	**2.21**	**0.75**	**2.19-2.23**

Gender & Health Hub

RQ39	What are the potential impacts of the COVID-19-induced digitalization of education on young women’s learning and employment opportunities,:The potential health benefit of answering this question is�	**71**	**2.20**	**0.75**	**2.18-2.22**
RQ14	What is the impact of COVID-19 on women’s access to financial and employment interventions	**74**	**2.19**	**0.79**	**2.17-2.21**
RQ19	To what extent has the pandemic's impact on unpaid care work influenced women's full time employment, partial employment or unemployment	**72**	**2.18**	**0.74**	**2.16-2.20**
RQ40	What policies, programmes and interventions are effective in mitigating the negative gendered impacts of school closures and online schooling	**72**	**2.18**	**0.79**	**2.16-2.20**
RQ12	Has the rate of job loss and re-employment during the pandemic, and in the medium term, differed by gender and other forms of social inequality (e.g. geographic location, parenthood, race, ethnicity, and migrant status, etc.)	**73**	**2.18**	**0.77**	**2.16-2.20**
RQ28	Has the private sector in female-dominated industries (e.g. transnational personal service providers) provided wage protection and safety nets for their workers during the pandemic	**67**	**2.16**	**0.71**	**2.14-2.28**
RQ3	What are the costs of GBV for survivors, perpetrators and their communities in the context of COVID-19	**80**	**2.14**	**0.78**	**2.12-2.26**
RQ10	How have interventions that use community mobilisation and peer-to-peer outreach to address GBV been adapted given social distancing measures, and how did this affect their quality, effectiveness, and cost	**76**	**2.13**	**0.82**	**2.11-2.15**
RQ29	To what extent have past gains in key gender equality indicators (such as female labour market participation, shared domestic responsibilities, child marriages, and school drop-outs) been reversed during the pandemic	**69**	**2.13**	**0.78**	**2.11-2.15**
RQ34	What policies, programmes and interventions are effective in promoting gender-equitable norms during the pandemic	**71**	**2.11**	**0.80**	**2.09-2.13**
RQ16	What policies, programmes and interventions have effectively promoted more gender-equitable employment outcomes during the pandemic	**73**	**2.11**	**0.74**	**2.09-2.13**
RQ33	What steps can be taken to reverse the effect of COVID-19 on women’s autonomy (over their bodies, their economic condition and their decision-making on public issues)	**67**	**2.10**	**0.74**	**2.08-2.13**
RQ5	What are the lived experiences and narratives of GBV survivors and of men who perpetrate GBV during the pandemic	**80**	**2.08**	**0.78**	**2.06-2.09**
RQ37	What policies, programmes and interventions are effective in reducing gendered stigma and discrimination during the pandemic	**69**	**2.07**	**0.79**	**2.05-2.09**
RQ30	How has the pandemic affected gender norms in different regions and contexts	**70**	**2.07**	**0.77**	**2.05-2.09**

BMJ Publishing Group Limited (BMJ) disclaims all liability and responsibility arising from any reliance placed on this supplemental material which has been supplied by the author(s)

RQ20	What policies, programmes and interventions have effectively promoted more gender-equitable outcomes in relation to unpaid care work during the pandemic	**71**	**2.07**	**0.76**	**2.05-2.09**
RQ21	How has COVID-19 impacted women's economic autonomy, and how has this further impacted their autonomy over their bodies, and in decision-making on public issues	**72**	**2.07**	**0.81**	**2.05-2.09**
RQ11	How has research on GBV in the context of the pandemic considered violence against LGBTQI+ persons and other marginalized groups	**74**	**2.05**	**0.83**	**2.03-2.08**
RQ9	How have government and community responses during the pandemic affected the availability, access and utilization of GBV services by survivors, when compared to pre-pandemic levels	**77**	**2.05**	**0.86**	**2.03-2.07**
RQ7	What policies, programmes and interventions have been successful and most cost-effective in preventing GBV during the pandemic, and over the long-term	**80**	**2.05**	**0.86**	**2.03-2.07**
RQ22	How has women and girls' limited access to resources before COVID-19 further constrained their economic opportunities and resilience during the pandemic	**73**	**2.04**	**0.81**	**2.02-2.06**
RQ32	How can digital technologies be used to increase the self-efficacy of girls and make them less vulnerable to harmful gender norms and inequitable gender roles	**70**	**2.01**	**0.77**	**1.99-2.04**
RQ42	How have changes in cleaning practices (e.g. increased use of bleach) affected occupational health hazards due to sex and gender	**65**	**1.97**	**0.87**	**1.94-2.00**
RQ43	What is the impact of COVID-19 responses on the environmental determinants of health and their gendered manifestations	**62**	**1.97**	**0.85**	**1.94-1.99**
RQ24	In what ways have new digital technologies provided more gender-inclusive, diverse and equitable work spaces during the pandemic	**70**	**1.94**	**0.81**	**1.92-1.97**
RQ23	Has the loss of jobs and income led to women starting different earning opportunities, or to set up their own small-scale businesses during the pandemic	**70**	**1.93**	**0.75**	**1.91-1.95**
RQ8	To what extent have women-led organisations and civil society organisations (CSOs) been involved in the delivery of non-health services for GBV prevention and response during the pandemic	**77**	**1.92**	**0.76**	**1.90-1.94**
RQ15	Has the pandemic affected the gender composition of certain labour sectors (such as healthcare) in the short and medium-term	**70**	**1.91**	**0.76**	**1.89-1.94**
RQ31	What are the determinants and pathways of changes in gender norms during and post pandemic	**69**	**1.91**	**0.78**	**1.89-1.94**
RQ41	Have social isolation measures affected indoor and outdoor air quality, and if so, how has this impact varied from a gender perspective	**67**	**1.91**	**0.83**	**1.89-1.93**
RQ13	How has automation and the use of new digital technologies affected (positively and negatively) the severity of COVID-19 impacts on female labour and unemployment	**70**	**1.90**	**0.71**	**1.88-1.92**

Gender & Health Hub
