# Supplementary material for: A shared agenda for gender and COVID-19 research: priorities based on broadening engagement in science
Source: BMJ Glob Health. 2023 May 22;8(5):e011315. doi: 10.1136/bmjgh-2022-011315 (PMC10230361; doi:10.1136/bmjgh-2022-011315)
Supplement: Supplementary data [file bmjgh-2022-011315supp007.pdf]

1

Gender & COVID-19 Research Agenda-Setting

Draft Thematic Reports for Initial Research Prioritisation

Thematic group 5

Gender and health governance for COVID-19

Understanding relationships across actors influencing power and decision making in health systems.

Table of Contents

Section 1: Introduction to the overall collaboration 2
Section 2. Thematic group participation and engagement 3
Section 3. Thematic group background 4
3.1 Definitions and scope 4
3.2 Current understanding, challenges, gaps and neglected areas 5
A.1. Socio-political ideology, values, and health system orientations 6
A.2. Government policies, regulatory bodies and legislative measures 7
A.3. Actor alignment and accountability 7
A.4. Citizen participation and inclusive governance 8
A.5. Data and research governance 9
3.3 Desired impact of the proposed research on policy, program, and community responses 10
3.4 Actors and strategies to implement and promote uptake of the research agenda 10
3.5 Population, contexts, study design/ methodologies (to be written at the end once research questions are prioritised) 10
Section 4. Research questions proposed for prioritisation. 11
B.1. Socio-political ideology, values, and health system orientations 11
B.2. Actor alignment and accountability 11
B.3. Citizen participation and inclusive governance 12
B.4. Government policies, regulatory bodies and legislative measures 12
B.5. Data and research governance 12
Section 5. References 13
Section 6. Resources shared 16
Section 7: Results of prioritised questions 17

Section 1: Introduction to the overall collaboration

From the start of the COVID-19 pandemic, how it affected and continues to affect biological sex in terms of male, female, intersex, as well as gender in terms of women, girls, men, boys, trans or gender fluid/ queer/ diverse persons is complex and evolving. Apart from the direct effects of COVID-19 illness, pandemic responses also amplified previously existing gender inequalities across multiple dimensions. Context and the intersecting influence of other social determinants and/or identities[1,2] also worsened the influence of gender during the pandemic, with combined effects on health. Unless mentioned otherwise, the reference to gender diverse intersectional exploration is implicit throughout this report.

Early high-level calls and advocacy from researchers[3] such as through the Gender and COVID-19 working group,[4] were made for gender considerations to be integrated in the crisis response. Nevertheless, real-time responses to gender dynamics were limited by ongoing bias by decision-makers combined with a weak evidence base due to incomplete data systems and evidence gaps. As the world steps into the second year of the COVID-19 crises, given how pre-existing gender discrimination has been amplified by the pandemic, we must include gender in the investments being made in research informing COVID-19 immediate action and long-term recovery to ensure transformation of how health systems serve their populations to accelerate health and well-being for all.

The United Nations University International Institute for Global Health is co-convening a collaborative gender and COVID-19 research agenda-setting exercise, as part of its Gender and Health Hub's inaugural scope of work. The process is co-developed through real-time learning, and open calls to a broad range of stakeholders to comment and contribute to its design, scope and content. Collective contributions and questions for prioritization are supported by a community discussion board (www.ghhbuzzboard.org). Please visit this discussion board for further information.

The output of the exercise will be a shared research agenda for civil society, program implementers, policy makers, funders, and researchers to guide COVID-19 research investments and corresponding programming and policy actions by primarily the health sector, but also those sectors that directly determine the social determinants of health where relevant.

The draft thematic group reports emerging from this collective endeavour are synthesized versions of the contributions made to the discussion board combined with additional inputs from thematic group coordinators, co-leads and steering committee members. They document participation and engagement to date, provide a background section outlining definitions, scope, gaps, impact and audiences, before listing research questions for prioritisation.

We welcome your comments on the discussion board or through the Google document which will be subsequently also shared on the discussion board to ensure we respect the inclusive and transparent ethos of the collaboration. If you comment via the Google document please make sure we can identify your comments (do not use anonymous). Given the devastating and dynamic nature of COVID-19, we must be inclusive but also timely.

Section 2. Thematic group participation and engagement

Thematic group coordinator: Mmabatho Motsamai (Botswana)

Thematic group co-leads: Manasee Mishra (India), Claire Standley (USA/Germany)

Thematic group steering committee focal point: Asha George (South Africa)

Other thematic group contributors:

1. ***Anjana Bhushan*** (India) **-** Technical Officer, WHO Regional Office for South-East Asia
2. ***Apurvakumar Pandya*** (India) **-** Scientist, Indian Institute of Public Health Gandhinagar
3. ***Arush Lal*** (United States of America/United Kingdom) **-** International Consultant, PAHO
4. ***Ateeb Ahmad Parray*** (Bangladesh) **-** Fellow, BRAC James P Grant School of Public Health, BRAC University
5. ***Avni Amin*** (Switzerland) **-** Technical Officer, WHO Regional Office for South-East Asia
6. ***Bernadette Ateghang-Awankem*** (Germany) **-** Executive Director, African Women 4 Empowerment
7. ***Clara Rodriguez Ribas*** (Argentina) **-** PhD Candidate, Universitat Pompeu Fabra
8. ***Dheepa Rajan (Switzerland) Health Financing and Governance, WHO*** (via googledoc)*
9. ***Fatima Bashir*** (Switzerland) **-** Programme Support Consultant, UNDP
10. ***Godelieve van Heteren (The Netherlands) WHO Health Governance Collaborative*** (via googledoc)
11. ***Kenechukwu Esom (USA) UNDP*** (via googledoc)*
12. ***Lignet Chepuka*** (Malawi) **-** Lecturer, University of Malawi, Kamuzu College of Nursing
13. ***Lucy Gilson (South Africa) University of Cape Town*** (via googledoc)*
14. ***Michelle McIsaac (Switzerland) Human Resources for Health, WHO*** (via googledoc)*
15. ***Minah Kang*** (South Korea) **-** Professor, Ewha Womans University
16. ***Nutan Prabha Jain*** (India) **-** Professor, IIHMR University, Jaipur
17. ***Rosana Lescrauwart Noboa, WEMOS, Netherlands*** *
18. ***Sambit Dash*** (India) **-** Assistant Professor of Biochemistry, Melaka Manipal Medical College
19. ***Sharon Attipoe-Dorcoo*** (United States of America) **-** Co-Principal, TERSHA LLC
20. ***Suresh Jungari*** (India) **-** Assistant Professor, Pune University
21. ***Tanya Jacobs*** (South Africa) **-** PhD Candidate, School of Public Health, University of the Western Cape
22. ***Theodora Koller (Switzerland) Gender, Equity and Rights, WHO*** (via googledoc)*

First draft report posted on discussion board on date 22 January 2021
Second draft report posted on discussion board on date 29 January 2021
Third draft report posted on discussion board on date 11 January 2021
Fourth draft report posted on discussion board on 19 March 2021 and shared with external experts

Section 3. Thematic group background

3.1 Definitions and scope

COVID-19 is both a moment of crisis and opportunity of change for health systems[2]. Amidst political and public health uncertainties, exacerbated by deepening social inequalities, the mandates of health systems have expanded, while the environments in which they operate are rapidly changing. These contextual dynamics require that health systems broker relationships across a broader range of interconnected and diverse actors[3]. As such, governance as an intrinsic and indispensable element of health systems, remains critically important to the COVID-19 pandemic response[30]

Governance refers to the social and political relationships and processes that bind health system actors together ideally towards a common purpose. Efforts to better define, understand and advance governance approaches[91112] grounded in action to improve health systems reflect multiple networks and collaborations between academics, practitioners and activists (Health Systems Governance Collaborative, COPASAH, Health Systems Global). Evolving understandings of governance in health systems include consideration of formal, as well as informal rules and norms. There is also recognition that governance is exercised not just at a central level, but across multiple levels of the health system and embedded in everyday decision-making.[14] Collective action and moral foundations are key to ensuring adaptive governance models that balance and broker negotiated relationships and sense-making across health system actors. Political economy perspectives that highlight analysis of interests, institutions and ideas remind us that governance efforts must address these and cannot be constrained to a technocratic, managerial function alone. Threaded through these articulations defining health governance is the core understanding that it entails the mediation of power between diverse actors to influence the design and implementation of health policies and services[15].

With regards to COVID-19, authors have noted the usefulness of adaptive governance frameworks[31] that are more inclusive of non-traditional actors[4] and that encourage bottom up change and learning which is key for resilience. Such perspectives are also relevant for advancing governance of One Health approaches[15] and other types of multi-sectoral action[5], including ‘Health in All Policies’, critical for COVID-19 responses and preventing future epidemics. Others note broader concerns such as the need to align global health security and universal health coverage agendas[6], the importance of considering the underlying political economy of COVID-19 with its historical linkages to extractive colonial legacies[7]; alongside the failure of global governance modalities[8].

Another key health governance issue with regards to pandemics is the importance of building resilience in health systems. Health systems resilience is understood as ensuring institutions, health actors and populations having preparedness and effective responses to pandemics and crises, as well as in chronically stressed health systems[8]. However, it also relates to the everyday processes of decision-making, leadership, and inter-personal/institutional networks that exist within all health systems, whether affected by chronic stress or not.

The gender elements across the spectrum of resilience considerations remain largely unexplored, with the exception of initial work examining gender and health sector leadership[20]. Critical commentaries on the lack of women in key COVID-19 decision-making bodies[32] , the nature of gendered national leadership during the pandemic[33] , and that largely gender blind or gender insensitive nature of COVID-19 pandemic national responses[36] have been the only gender analysis undertaken in this emergent literature on health governance and COVID-19.

A key aspect of gender responsive COVID-19 health systems is ensuring that gender is mainstreamed throughout. More mapping is required to understand whether and how gender has been taken into consideration in existing COVID-19 planning. Much remains unknown about how to support the capacities of decision makers at multiple levels of the health system to ensure gender responsiveness to COVID-19. What kinds of evidence are required and what kinds of best practices are helpful?. What kinds of accountabilities are required to ensure adequate financing of gender responsive measures?

While there is a body of work that applies a gender perspective into governance broadly[5], gendered analyses of health governance has been less substantial. Areas of work include attention to gender mainstreaming in the health sector[6]; gender dynamics in social accountability in the health sector[7–10] and most recently the role of gender in health sector leadership, including during the COVID-19[11–16] response. This research prioritisation effort on gender and the governance of health systems during the pandemic therefore provides an opportunity to more deeply consider gender dynamics in health systems governance that have remained neglected for far too long.

A mapping of gender and governance issues can be done across different health system levels. For example, while micro-level governance dynamics includes gendered patient-provider interpersonal relations, meso-level factors include gendered organisational dynamics that limit women's roles in health sector leadership, and macro-level considerations include those related to political ideology shaping gender norms in party politics, governmental decision-making and legislative matters. These dynamics also interact across health systems levels, enabling gender power dynamics to be reconfigured or retrenched. While gender dynamics in health systems governance were contested prior to the pandemic, critical analysis is needed to understand how the pandemic has changed this for better or worse.

Other thematic reports developed under this research-setting initiative, centre around individual and population health behaviour (TG1), health service delivery (TG3), and social determinants (TG4). In this report we will therefore focus largely on interactions between meso- and macro-level factors, operating both within and from outside the health sector, which influence the gendered nature of health systems governance as they respond to and recover from the COVID-19 pandemic.

3.2 Current understanding, challenges, gaps and neglected areas

Given that there are multiple dimensions and levels of health governance that are relevant to gender and COVID-19 dynamics, we review the gendered governance architecture of COVID-19 health systems

- Socio-political ideology, values, and health system orientations
- Government policies, regulatory bodies and legislative measures
- Actor alignment and accountability
- Citizen participation and inclusive governance
- Regulatory and legislative measures
- Data and research governance.

Figure 1: Gendered health systems governance web enabling gender mainstreamed, responsive and resilient health systems

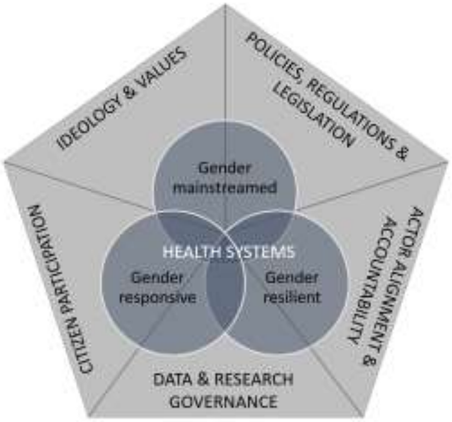

A.1. Socio-political ideology, values, and health system orientations

This section is about how gendered governance dynamics at the broadest level in terms of ideology and values shapes health systems and the basis for health reforms, including those related to COVID-19

Health systems are shaped by political ideology and this has gendered implications. The rise of far-right movements, embedded in repressive ideologies (patriarchy, colonialism, misogyny, etc.) has resulted in the curtailing of recognition and rights of marginalised women and gender diverse persons to quality health services, as flagged by ongoing contestation of the global gag rule. Ideology not only manifests itself in shaping visible policies determining entitlements and resource priorities, but also through soft power[28]. The latter implicitly shapes the framing and values with which decisions are articulated and made. The role of political ideology and its impact on explicit and implicit gendered decision making in COVID-19 government policy reforms, budgeting priorities, and policy implementation requires further conceptualisation and examination.

By exposing the extent of social inequalities undermining pandemic policies and the impact of interventions, COVID-19 also presents an opportunity for political leaders to reaffirm commitments to a renewed social contract that energises action addressing the social determinants of health. Further understanding of how gender and intersectionality have featured in these political windows of opportunity is needed.

Ideology, history and political systems all shape how health systems are organised in varied ways, with different balances between curative, preventive, and multi-sectoral elements. Primary health care (PHC), universal health coverage (UHC) and global health security (GHS) are different framings that direct such orientations. Lal et al., by assessing the ability of health systems to manage COVID-19 demonstrate how global health security (GHS) and universal health coverage (UHC) align priorities and objectives in strengthening health systems in different ways.[25] While these framings for health systems may or may not intrinsically address aspects of inequity, they do not preclude the need for targeted policies, programmes or actions to ensure inclusivity for marginalis ed and vulnerable groups. Research is needed to ensure that gender, among other inequities, is addressed by health systems reforms informed by PHC, UHC, GHS and taken into account during pandemic response, as well as subsequent recovery efforts.

A.2. Government policies, regulatory bodies and legislative measures

The formal ‘rules of the game’ are expressed in health governance through government policies, regulatory bodies and legislative measures

Government policies, regulatory bodies and legislative measures are never gender-neutral. They are laden with assumptions, social prejudices and gender-stereotypes prevalent in a particular context. The under-representation of women and gender non-binary people in legislative and policymaking bodies reinforces and perpetuates the disproportionate impact of regulatory and legislative measures on women and girls. Lessons can be drawn from HIV about how different legal systems and aspects of law negatively impact women and girls and their health.

COVID-19 policies, regulatory bodies and legislative measures are no different. The COVID-19 Global Gender Response Tracker monitors national pandemic responses taken by governments and examines which have integrated a gender lens. It found that less than half of the over 3000 policies examined were gender sensitive (addressing either women’s economic and social security, unpaid care work or violence against women). A separate policy tracked found that 91% of the 450 policies reviwed were gender blind. For example, many countries initially began to ease lockdown measures by designating days when people could go out for essential shopping etc by gender. In Colombia, men got four days and women three days of the week, without any rationalisation provided for why men require more days out than women. Gender-diverse people were not mentioned at all.

Also important to note is how COVID-19 impacted the functioning of parliaments and judiciaries where capacities for integrating gender lens to lawmaking and law interpreting have been developing for a while. With a majority of COVID-19 laws and regulations being made by the Executive through executive orders and fiats, the accountability around gender inclusiveness that existed to some degree in Parliaments was thus absent, and escaped legislative scrutiny of gender considerations in these laws and regulations.

A.3. Actor alignment and accountability

Governance is also about ensuring coordination, alignment and accountability across the diverse actors that contribute to health. This sections reviews

One of the challenges facing governance in health, is that many of the global, national and sub-national actors contributing to health are outside of the authority of Ministries of Health or the World Health Organisation. Few have examined the effect that fragmented governance for health, where disparate actors are not coordinated, has had on COVID-19 responses[8]. This includes navigating corporate interests, supremacist government tendencies, under-resourced and divided social movements, and donor priorities that may or may not align with local gender and COVID-19 needs. Research that can inform the political strategies required to align actors to advance gender considerations within this complex fragmented landscape is required.

One area where further alignment or coordination is needed is multi-sectoral action for health, made even more relevant by COVID-19. Not just because of its basis in human-animal transmission exacerbated by climate-stressed globalised economies, unsustainable exploitation of natural resources, and devastating losses of habitat and biodiversity, but also because the short and long

term gendered impacts of COVID-19 lockdown require multi-sectoral action. Gender equality measures were missing from over half of global pandemic social policy responses (ref UNDP policy tracker). It is therefore critical to understand how to advance gender concerns in emergent multi-sectoral pandemic responses. Appropriate indicators and tools must be developed to measure and assess cross-sectoral progress towards more integrated, resilient, less fragmented and more equitable health governance systems during COVID-19 and beyond[34].

The urgency of COVID-19 responses has led to contributions from many non-state actors, providing opportunities as well as risks for gender. Corruption in the tendering processes for protective equipment[37,38,39] not only disproportionately affect women because they constitute the majority of health workers at the patient and community interface of COVID-19 but also indirectly affects their morale. The unregulated nature of pricing of health care services and commodities by the private for profit sector may also disproportionately affect women who have less disposable income for health.

Community-based organisations may be best placed to respond to gendered community needs, with regards to housing and food security during lockdown, as well as protection and prevention of gender based violence[17]. Co-production efforts with communities was essential in responding to the Ebola pandemic (Anoko et al. 2020). However, community leadership and organisations may also reflect patriarchal or other conservative social norms that exclude key marginalised populations. Research is needed to examine what forms of governance ensures oversight over these non-state actors supporting service delivery from a gender/intersectional and COVID-19 perspective.

Social movements play a critical role in giving voice to marginalised groups and protecting social liberties. Due to the measures undertaken to restrict the spread of COVID-19, the ability of social movements and civil society to politically mobilise and represent marginalised groups is critical, yet it has often been curtailed in the name of public health security[18]. Not only does governance research need to track the effects of the pandemic on feminist movements, but they also understand the social and political fissures that exist across social movements, and how these are overcome to advance gender concerns in pandemic responses.

A.4. Citizen participation and inclusive governance

A key aspect of governance is its democratic potential enabling broader citizen participation and representation of voices among those most marginalised.

While governance includes democratic principles enabling broad participation in decision making, the inclusive nature of such participation is difficult to ensure. The presence of systemic patriarchy and exclusion of women and gender diverse people in health governance remains a key concern during the COVID-19 pandemic. While Fioramonti et al. [21] argues that countries with female leaders have suffered only a sixth as many COVID-19 deaths as those led by men, others have been more cautious about such analysis and the possible biases involved[24]. It is imperative to view women and gender diverse persons' inclusion with a critical eye, so as to not lump leadership trust and participation with stereotyped biases or expectations. Rather than essentialise women leaders, the social contract and nature of politics in their countries may enable more representative and better governance of health systems during times of crisis. The aim for inclusion and representation of women, gender-diverse voices, and non-traditional stakeholders in health decision-making primarily looks towards ensuring gender equity and intersectional justice in pandemic preparedness and management. Research is needed to examine how best to advance more

equitable representation in decision making processes in ways that are transparent and accountable to health equity goals[26].

Add para on community participation as above para is about individual participation FYI – see UHC2030's Civil Society Engagement Mechanism survey results: https://csemonline.net/results-from-the-civil-society-participation-in-the-covid-19-response/

Gender sensitive, equitable and resilient health systems require continual monitoring and evaluation that supports feedback to all actors to inform decision making and implementation. These feedback mechanisms contribute to ensuring transparency and accountability of COVID-19 services and policies. Similarly, citizen-centred monitoring can provide critical checks and balances. Adaptations to how data can be used by citizens to affirm gendered COVID-19 responses must be explored. Multi-layered observation, documentation and dialogue is required to track and steer progress towards gendered health systems, throughout the COVID-19 response and recovery.

A.5. Data and research governance

Sound decision making is ideally informed by representative data and information systems. This section covers the gendered nature of the information systems that support health governance ranging from governance of digital information systems, infodemics and research infrastructure.

Technology and ICT are key factors in health governance for COVID-19, given the use of digital technology and the need for streamlining electronic databases to ensure more effective health sector responses in terms of surveillance, testing, mapping emerging health needs, regulating providers, etc. The rise of ICT has led to more synthesized and accessible data, enabling the possibility of big data and requiring an understanding of information and data governance, including the ethics of data mining, information management, anonymity and cost effectiveness[24]. The gendered vulnerabilities and capabilities involved require further elucidation, particularly given the gendered nature of digital health access and use prior to COVID-19.

COVID-19 is a pandemic that has spurred an infodemic. A gender analysis of press coverage of COVID-19, media influencers and the modalities by which fake news on COVID-19 is propagated is yet to be undertaken[19]. Has COVID-19 amplified or provided opportunity for gender biases within the media to be addressed? These issues further extend into the academic sphere with respect to gauging research quality, particularly with the rise in the popularity of pre-print servers, where papers are often picked up by media sources and widely disseminated prior to undergoing peer-review. Are there implications for gender and COVID-19 lay knowledge and research?

COVID-19 has cemented itself as the largest, most impactful pandemic in the 21st century on the global economy. This has led to many industries facing uncertainty of work, reduced financing and funding. Research institutions, whether based in universities or not, already impacted by uncertain and declining funding streams, underlined with a disinvestment in public knowledge generation and investment, are further marginalised in the political economy of COVID-19 resource allocation. Gendered dimensions of this include the predominance of female faculty in less desirable contract positions in research organisations, the lack of childcare and family support throughout and global inequities in global health research, all exacerbated by COVID-19[19]. The gendered production of COVID-19 knowledge generation has implications for the evidence base from which future policy and decision making are supported.

3.3 Desired impact of the proposed research on policy, program, and community responses
TO FILL OUT AT THE END ONCE THE OVERALL STRUCTURE AND CONTENT IS CLEAR

3.4 Actors and strategies to implement and promote uptake of the research agenda
Implementation
Once the research agenda is set, there is an opportunity for collaboration with universities and colleges with existing *Gender and COVID-19* research groups.

Types of institutions and inclusion
Global research institutions such as the Health Systems Governance Collaborative, WHO Alliance for Health Policy and Systems Research as well as identifying national and local level actors such as civil society and community engagement actors can implement variances of the research agenda once questions are prioritized.

WHO's DG, Dr.Tedros, acknowledged and apologised for the insufficient civil society consultations and engagement through the COVID-19 pandemic[29], particularly around gender. In rectifying consultations, a long-term impact would be a formal dialogue such as a WHO-Civil Society Commission that keeps gender and intersectionality as a standing agenda item, relevant to any health topic that arises.

The Lancet Commission on Maximizing Synergies, GPMB, and UHC2030 have adapted elements of this research agenda and further work can be done through collaboration.

WIGH

3.5 Population, contexts, study design/ methodologies (to be written at the end once research questions are prioritised)

Section 4. Research questions proposed for prioritisation.

35 QUESTIONS

A. COVID-19 Cross-cutting gendered governance

1. What do gender mainstreamed, responsive and resilient health systems look like? And what aspects are important with respect to COVID-19 and other pandemic threats?
2. To what extent and how is gender considered in the current decision making and learning processes for prioritization and reorganisation of services due to COVID-19?
3. What kind of evidence on gender and COVID-19 is needed for decision-makers, and for what type of decision makers (government, donor organization, civil society etc.)?
4. How can we formulate and integrate gendered social and economic analyseis to better aid/support gender-responsive decision-making for COVID-19?

B. COVID-19 Gendered governance architecture

B.1. Socio-political ideology, values, and health system orientations

5. How does political ideology influence the gendered experience of health systems, including the extent to which health systems respond to gendered COVID-19 needs?
6. What are the gendered implications of health system alignment with either global health security vs. universal health care paradigms when responding to COVID-19?
7. To what extent can COVID-19 provide a political window of opportunity for addressing the social and economic determinants of health, particularly related to gender and intersectionality? What political economy factors that can enable this?
8. What is required to strengthen political will and feasibility for investing in gender mainstreamed health systems to address COVID-19 and future pandemic threats?

B.2. Actor alignment and accountability

9. Who are the actors shaping COVID-19 health policies, and what is their affinity and/or accountability for advancing gender considerations in pandemic responses?
10. What capacities are needed to support decision makers, who may not be gender experts themselves, to enable more gender-responsive decisions for COVID-19 and future pandemic threats?
11. What best practices can be used for parliamentarians, decision makers in MoH (health ministries) and MoF (finance ministries) in coordination and prioritization of gender and COVID-19 issues?
12. What approaches are most effective at successfully integrating multiple sectors into pandemic planning and response, and ensuring gender is centrally considered throughout these processes?
13. What is the gendered impact of having non-health background officials in a multipronged health problem like Covid-19?
14. What governance mechanisms are required to ensure oversight of non-state actors (community based and private health providers) in service delivery from a gender and COVID-19 perspective?

15. How is the gendered nature of corruption impacted in terms of access and procurement of vaccines/services?
16. How have social movements advancing gender equality and intersectional justice been affected by COVID-19 and COVID-19 measures?
17. What factors shape opportunities for social movements to align and affirm gender concerns in pandemic responses?

B.3. Citizen participation and inclusive governance

18. To what extent have citizens and community members been involved in the implementation of COVID-19 measures – especially in vulnerable areas? Which voices are heard more and why and what are the implications for gender and COVID-19 concerns?
19. What principles and strategies of engagement for linking marginalised communities with local, sub national and national government health administration and political decision makers best support the advancement of gender and COVID-19 issues?
20. What are the best ways of ensuring gender and intersectional balance and inclusion in decision-making bodies governing COVID-19 responses?
21. What are the strengths and limitations of relying soley on "female stewardship" to implement a gender and rights-based approach to COVID-19?
22. What principles and strategies are most effective in supporting male allyship in advancing a gender and rights based approach to COVID-19?

B.4. Government policies, regulatory bodies and legislative measures

23. What are the gender implications of the emergency regulatory and legislative changes made due to COVID-19, both positive and negative?
24. How gender responsive are COVID-19 response and recovery plans, the budgets allocated to them, and the expenditures realised?

25. What is the gendered impact of the current patent and trade agreements on Covid-19 vaccinesfor disadvantaged populations?
26. How can regulations on local, in-country therapeutic trials take biological sex, gender and ethnicity into account, thereby providing data on efficacy and adverse effects that more accurately reflects specific populations?
27. How can regulatory bodies be legally bound to include age, sex, gender and race data in trial information that is made available to the public?
28. What is the governing role of international assistance, foundations and other donors in providing social protection in LMICs – how has the pandemic affected their policy provisions in realizing gender equality?

B.5. Data and research governance

29. What are the gendered elements of data privacy and protection given COVID-19 digital health tracking and surveillance measures?
30. What regulations are needed on the extent of COVID-19 data that should be made legally available to the public? What are the gendered and intersectional considerations?
31. Has the gendered nature of access and use of digital health improved or gotten worse due to COVID-19?
32. What are the gender dynamics involved in COVID-19 infodemics? How best to address them?
33. What is the nature of understanding of gender and COVID-19 dynamics in the media? How best to improve gendered understanding and reporting of COVID-19 issues by the media?
34. What strategies best redress the gendered nature of knowledge production, ie how those with family commitments during COVID-19 have not been able to contribute to knowledge production compared to those without family commitments?
35. Has COVID-19 provided an opportunity to reform health research organisations, providing more gender equitable ways of working? If so what supported such reforms?

Section 5. References

1 Hankivsky O, Kapilashrami A. Beyond sex and gender analysis: an intersectional view of the COVID-19 pandemic outbreak and response. 2020. https://mspgh.unimelb.edu.au/__data/assets/pdf_file/0011/3334889/Policy-brief_v3.pdf (accessed July 3, 2020).

2 Lokot M, Avakyan Y. Intersectionality as a lens to the COVID-19 pandemic: implications for sexual and reproductive health in development and humanitarian contexts. *Sex Reprod Health Matters* 2020; **28**: 1764748.

3 Wenham C, Smith J, Morgan R. COVID-19: the gendered impacts of the outbreak. *The Lancet* 2020; **395**: 846–8.

4 Gender and COVID19 Working Group. Gender & Covid-19 Working Group. Gend. Covid-19. 2020. https://www.genderandcovid-19.org/gender-working-group-page/ (accessed Jan 19, 2021).

5 Goetz - 2008 - Who answers to women gender & accountability.pdf. https://www.unwomen.org/-/media/headquarters/media/publications/unifem/poww08reportfulltext.pdf?la=en&vs=1016 (accessed Feb 23, 2021).

6 Ravindran TKS, Kelkar-Khambete A. Gender mainstreaming in health: looking back, looking forward. *Glob Public Health* 2008; **3**: 121–42.

7 Murthy RK. Strengthening accountability to citizens on gender and health. *Glob Public Health* 2008; **3 Suppl 1**: 104–20.

8 Lodenstein E, Pedersen K, Botha K, Broerse JEW, Dieleman M. Gendered norms of responsibility: reflections on accountability politics in maternal health care in Malawi. *Int J Equity Health* 2018; **17**: 131.

9 van Daalen et al. - 2020 - Symptoms of a broken system the gender gaps in CO.pdf. https://gh.bmj.com/content/bmjgh/5/10/e003549.full.pdf (accessed Feb 23, 2021).

10 Snapshot. https://bulletin.ids.ac.uk/index.php/idsbo/article/view/2967/Online%20article (accessed Feb 23, 2021).

11 Zeinali Z, Muraya K, Govender V, Molyneux S, Morgan R. Intersectionality and global health leadership: parity is not enough. *Hum Resour Health* 2019; **17**. DOI:10.1186/s12960-019-0367-3.

12 Shung-King M. Leadership experiences and practices of South African health managers: what is the influence of gender? -a qualitative, exploratory study. 2018; : 12.

13 Dada S, Ashworth HC, Bewa MJ, Dhatt R. Words matter: political and gender analysis of speeches made by heads of government during the COVID-19 pandemic. *BMJ Glob Health* 2021; **6**: e003910.

14 Muraya KW, Govender V, Mbachu C, Uguru NP, Molyneux S. 'Gender is not even a side issue…it's a non-issue': career trajectories and experiences from the perspective of male and female healthcare managers in Kenya. *Health Policy Plan* 2019; **34**: 249–56.

15 One Health. https://www.who.int/news-room/q-a-detail/one-health (accessed Feb 21, 2021).

16 Sheikh K, Gilson L, Agyepong IA, Hanson K, Ssengooba F, Bennett S. Building the Field of Health Policy and Systems Research: Framing the Questions. *PLOS Med* 2011; **8**: e1001073.

17 World Health Organization, editor. Innovative care for chronic conditions: building blocks for action: global report. Geneva: World Health Organization, 2002.

18 Gender Differences and the ACA. Cent. Health Econ. 2014; published online April 16. https://healthandeconomy.org/gender-differences-and-the-aca/ (accessed Feb 25, 2021).

19 Barker et al. - 2020 - Community engagement for health system resilience.pdf. - (accessed Feb 21, 2021).

20 Snapshot. https://www.ghhbuzzboard.org/forum/tg5-governance/completed-5-1-what-should-be-the-scope-of-research-for-this-group-please-suggest-boundaries-for-exclusion-and-inclusion (accessed Feb 24, 2021).

21 Women in power: it's a matter of life and death – Lorenzo Fioramonti, Luca Coscieme and Katherine Trebeck. Soc. Eur. 2020; published online June 1. https://www.socialeurope.eu/women-in-power-its-a-matter-of-life-and-death (accessed Feb 21, 2021).

22 Stiglitz - Conquering the Great Divide.pdf. https://www.imf.org/external/pubs/ft/fandd/2020/09/pdf/COVID19-and-global-inequality-joseph-stiglitz.pdf (accessed Feb 24, 2021).

23 Crenshaw KW. Intersectionality, Identity Politics and Violence Against Women of Color. *Kvind Køn Forsk* 2006; published online June 23. DOI:10.7146/kkf.v0i2-3.28090.

24 Tiffin N, George A, LeFevre AE. How to use relevant data for maximal benefit with minimal risk: digital health data governance to protect vulnerable populations in low-income and middle-income countries. *BMJ Glob Health* 2019; **4**. DOI:10.1136/bmjgh-2019-001395.

25 Lal A, Erondu NA, Heymann DL, Gitahi G, Yates R. Fragmented health systems in COVID-19: rectifying the misalignment between global health security and universal health coverage. *The Lancet* 2021; **397**: 61–7.

26 Erondu et al. - 2021 - Improving National Intelligence for Public Health .pdf. https://gh.bmj.com/content/bmjgh/6/1/e004227.full.pdf (accessed Feb 24, 2021).

27 Suter E, Oelke ND, Adair CE, Armitage GD. Ten Key Principles for Successful Health Systems Integration. *Healthc Q Tor Ont* 2009; **13**: 16–23.

28 Vuving - 2009 - How Soft Power Works.pdf. https://apcss.org/Publications/Vuving%20How%20soft%20power%20works%20APSA%202009.pdf (accessed Feb 22, 2021).

29 Input-paper-for-DG-Dialogue-on-social-accountability-final.pdf. https://csemonline.net/wp-content/uploads/2020/10/Input-paper-for-DG-Dialogue-on-social-accountability-final.pdf (accessed Feb 22, 2021).

30 Bennett S, Agyepong IA, Sheikh K, Hanson K, Ssengooba F, Gilson Building the Field of Health Policy and Systems Research: An Agenda for Action L (2011). PLoS Med 8(8): e1001081. https://doi.org/10.1371/journal.pmed.1001081

31 Rao NV, Prashanth N, Hebbar PB, Beyond numbers, coverage and cost: adaptive governance for post-COVID-19 reforms in India, BMJ Global Health 2021;6:e004392.

32 van Daalen KR, Bajnoczki C, Chowdhury M, et al Symptoms of a broken system: the gender gaps in COVID-19 decision-making (2020) BMJ Global Health 2020;5:e003549.

33 Windsor LC, Yannitell Reinhardt G, Windsor AJ, Ostergard R, Allen S, Burns C, et al. Gender in the time of COVID-19: Evaluating national leadership and COVID-19 fatalities (2020). PLoS ONE 15(12): e0244531. https://doi.org/10.1371/journal.pone.0244531

34 Rajan D, Koch K, Rohrer K, et al Governance of the Covid-19 response: a call for more inclusive and transparent decision-making BMJ Global Health 2020;5:e002655

36 Snapshot https://data.undp.org/gendertracker/ UNDP COVID-19 Global Gender Response Tracker (accessed May 28, 2021)

37 Covid-19 corruption: There's still a great deal of it going down in Gauteng. 17 November 2020 Published online https://www.dailymaverick.co.za/article/2020-11-17-covid-19-corruption-theres-still-a-great-deal-of-it-going-down-in-gauteng/ (accessed at May 28, 2021)

38 Abbasi K. Covid-19: politicisation, "corruption," and suppression of science BMJ 2020; 371 :m4425 doi:10.1136/bmj.m4425 (accessed May 28, 2021)

39 COVID-19 has created conditions in which corruption in health procurement can flourish. Here's how Open Contracting would help. 7 JuLY 2020. Published online

https://www.transparency.org.uk/covid-19-has-created-conditions-which-corruption-health-procurement-can-flourish-heres-how-open (accessed May 28, 2021)

Section 6. Resources shared

1Mikkelsen-Lopez I, Wyss K, de Savigny D. An approach to addressing governance from a health system framework perspective. *BMC Int Health Hum Rights* 2011; **11**: 13. https://bmcinthealthhumrights.biomedcentral.com/track/pdf/10.1186/1472-698X-11-13.pdf (accessed Feb 25, 2021).

2Lal A, Erondu NA, Heymann DL, Gitahi G, Yates R. Fragmented health systems in COVID-19: rectifying the misalignment between global health security and universal health coverage. *The Lancet* 2021; **397**: 61–7.

3Siddiqi S, Masud TI, Nishtar S, *et al.* Framework for assessing governance of the health system in developing countries: Gateway to good governance. *Health Policy* 2009; **90**: 13–25.

4Kickbusch I, Gleicher D. Governance for health in the 21st century. Copenhagen: World Health Organization, Regional Office for Europe, 2013. Kickbusch and Gleicher - 2013 - Governance for health in the 21st century.pdf. https://www.euro.who.int/__data/assets/pdf_file/0019/171334/RC62BD01-Governance-for-Health-Web.pdf (accessed Feb 25, 2021).

5Lewis M, Pettersson G. Governance In Health Care Delivery : Raising Performance. The World Bank, 2009 DOI:10.1596/1813-9450-5074.. http://documents1.worldbank.org/curated/en/792741468330936271/pdf/WPS5074.pdf (accessed Feb 25, 2021).

6Fryatt R, Bennett S, Soucat A. Health sector governance: should we be investing more? *BMJ Glob Health* 2017; **2**: e000343.full.pdf. https://gh.bmj.com/content/bmjgh/2/2/e000343.full.pdf (accessed Feb 25, 2021).

7Abimbola S. Health system governance: a triangle of rules. *BMJ Glob Health* 2020; **5**: e003598.

8Meessen B. Health system governance: welcoming the reboot. *BMJ Glob Health* 2020; **5**: e002404.

9Veillard JHM, Brown AD, Barış E, Permanand G, Klazinga NS. Health system stewardship of National Health Ministries in the WHO European region: Concepts, functions and assessment framework. *Health Policy* 2011; **103**: 191–9.

10Bigdeli M, Rouffy B, Lane BD, Schmets G, Soucat A. Health systems governance: the missing links. *BMJ Glob Health* 2020; **5**: e002533.

11HSAA_Manual_Version_2_Sept_20121.pdf. https://www.hfgproject.org/wp-content/uploads/2015/02/HSAA_Manual_Version_2_Sept_20121.pdf (accessed Feb 25, 2021).

12Abimbola S, Negin J, Martiniuk AL, Jan S. Institutional analysis of health system governance. *Health Policy and Planning* 2017; **32**: 1337–44. Abimbola et al. - 2017 - Institutional analysis of health system governance.pdf. (accessed Feb 25, 2021).

13Smith PC, Anell A, Busse R, *et al.* Leadership and governance in seven developed health systems. *Health Policy* 2012; **106**: 37–49.

14George A, LeFevre AE, Jacobs T, *et al.* Lenses and levels: the why, what and how of measuring health system drivers of women's, children's and adolescents' health with a governance focus. *BMJ Glob Health* 2019; **4**: e001316. https://gh.bmj.com/content/bmjgh/4/Suppl_4/e001316.full.pdf (accessed Feb 25, 2021).

15Bump JB, Baum F, Sakornsin M, Yates R, Hofman K. Political economy of covid-19: extractive, regressive, competitive. *BMJ* 2021; : n73.https://www.bmj.com/content/bmj/372/bmj.n73.full.pdf (accessed Feb 25, 2021).

16 Research: Women Are Better Leaders During a Crisis. *Harvard Business Review* 2020; published online Dec 30. https://hbr.org/2020/12/research-women-are-better-leaders-during-a-crisis (accessed Feb 25, 2021).

17Kirigia and Kirigia - 2011 - The essence of governance in health development.pdf. https://www.ncbi.nlm.nih.gov/pmc/articles/PMC3072323/pdf/1755-7682-4-11.pdf (accessed Feb 25, 2021).

18 Abimbola S, Negin J, Jan S, Martiniuk A. Towards people-centred health systems: a multi-level framework for analysing primary health care governance in low- and middle-income countries. *Health Policy and Planning* 2014;**29**: ii29–39.

19 Gilson L, Marchal B, Ayepong I, *et al.* What role can health policy and systems research play in supporting responses to COVID-19 that strengthen socially just health systems? *Health Policy and Planning* 2020; **35**: 1231–6.

20 Will the Pandemic Reshape Notions of Female Leadership? *Harvard Business Review* 2020; published online June 26. https://hbr.org/2020/06/will-the-pandemic-reshape-notions-of-female-leadership (accessed Feb 25, 2021).

Section 7: Results of prioritised questions

Table 6: High Priority Gender and COVID-19 Research Questions per criteria

RQ	Label	Public Health	Gender equality	Urgency for policy
RQ11	To what extent, and how, is gender considered in the current decision making and learning processes for COVID-19	**X**	**X**	**X**
RQ28	What are the gender implications of COVID-19 response and recovery plans, the budgets allocated, and the resultant expenditures	**X**	**X**	**X**
RQ6	What forms of gendered abuse of power in health systems are made worse during pandemic health system responses and how can they best be addressed	**X**	**X**	**X**
RQ16	What kind of evidence on gender and COVID-19 is needed for decision-makers, and does this vary by type of decision maker (government, donor organization, civil society etc.)	**X**	**X**	**X**

RQ24	Have vaccine deployment strategy plans included ways to consult and engage with gender experts, womenâ€™s and high-risk marginalized groups	X	X	X
RQ33	How to best support national statistical systems to produce and use sex and gender data during COVID-19 and future pandemics	**X**	**X**	
RQ1	What do responsive and resilient health systems that address gender bias and advance gender equality look like	**X**	**X**	
RQ12	Who are the actors shaping COVID-19 health policies, and what is their affinity and/or accountability for addressing gender bias in pandemic responses		**X**	**X**
RQ23	What principles and strategies for linking marginalised communities with government health administrators and political decision makers best supports the advancement of gender and COVID-19 issues	**X**	**X**	
RQ22	Which voices are heard more and why when involving citizens and community members in the planning and implementation of COVID-19 measures and what are the implications for gender and COVID-19 concerns	**X**	**X**	
RQ5	What is required to strengthen political will for investing in health systems that address gender bias and advance gender equality	**X**	**X**	
RQ13	What capacities are needed to support decision makers, who may not be gender experts themselves, to enable responses to COVID-19 and future pandemic threats that address gender bias	**X**	**X**	
RQ4	To what extent can COVID-19 provide a political window of opportunity for addressing the social and economic determinants of health, particularly related to gender and intersectionality		**X**	**X**
RQ27	What are the positive and negative gender implications of the emergency regulatory and legislative policy changes made due to COVID-19		**X**	
RQ18	What are the best ways of ensuring gender and intersectional balance and inclusion in decision-making bodies governing COVID-19 responses		**X**	
RQ10	What approaches are most effective at successfully integrating different sectors into pandemic planning and response, and to ensuring that gender is centrally considered throughout in this multi-sectoral action	**X**		
RQ32	Has COVID-19 provided an opportunity to reform health research organisations, thereby providing more gender equitable ways of working		**X**	
RQ36	What data collaborative frameworks are needed to share responsibly and ethically COVID-19 sex and gender data between governments, academia and the private sector		**X**	
RQ3	What are the gendered implications of different health system paradigms such as global health security or universal health care when responding to COVID-19		**X**	
RQ29	What is the gendered impact of the current patent and trade agreements on COVID-19 vaccines for disadvantaged populations			**X**
RQ9	How has the pandemic affected international donors and what have been the implications for gender and COVID-19 issues			**X**

Supplementary Table A: Sample size, Means, Standard deviations and 95% Confidence Intervals for Gender and COVID-19 Research Questions by Public Health Benefit (*12 out of 35 as statistically significant top research questions)

RQ	Label	M	Mean	SD	95% CI
*RQ10	What approaches are most effective at successfully integrating different sectors into pandemic planning and response, and to ensuring that gender is centrally considered throughout in this multi-sectoral action	**31**	**3.58**	**0.56**	**3.54-3.62**
*RQ33	How to best support national statistical systems to produce and use sex and gender data during COVID-19 and future pandemics	**27**	**3.56**	**0.70**	**3.50-3.61**
*RQ1	What do responsive and resilient health systems that address gender bias and advance gender equality look like	**35**	**3.54**	**0.61**	**3.51-3.58**
*RQ11	To what extent, and how, is gender considered in the current decision making and learning processes for COVID-19	**30**	**3.50**	**0.68**	**3.46-3.54**
*RQ23	What principles and strategies for linking marginalised communities with government health administrators and political decision makers best supports the advancement of gender and COVID-19 issues	**29**	**3.50**	**0.60**	**3.46-3.54**
*RQ24	Have vaccine deployment strategy plans included ways to consult and engage with gender experts, women's and high-risk marginalized groups	**27**	**3.48**	**0.70**	**3.43-3.53**
*RQ28	What are the gender implications of COVID-19 response and recovery plans, the budgets allocated, and the resultant expenditures	**27**	**3.44**	**0.64**	**3.40-3.49**
*RQ22	Which voices are heard more and why when involving citizens and community members in the planning and implementation of COVID-19 measures and what are the implications for gender and COVID-19 concerns	**27**	**3.41**	**0.69**	**3.36-3.46**
*RQ5	What is required to strengthen political will for investing in health systems that address gender bias and advance gender equality	**31**	**3.39**	**0.76**	**3.34-3.44**
*RQ6	What forms of gendered abuse of power in health systems are made worse during pandemic health system responses and how can they best be addressed	**29**	**3.38**	**0.68**	**3.33-3.43**
*RQ16	What kind of evidence on gender and COVID-19 is needed for decision-makers, and does this vary by type of decision maker (government, donor organization, civil society etc.)	**28**	**3.38**	**0.70**	**3.33-3.42**
*RQ13	What capacities are needed to support decision makers, who may not be gender experts themselves, to enable responses to COVID-19 and future pandemic threats that address gender bias	**29**	**3.34**	**0.77**	**3.29-3.40**
RQ18	What are the best ways of ensuring gender and intersectional balance and inclusion in decision-making bodies governing COVID-19 responses	**28**	**3.34**	**0.67**	**3.29-3.39**
RQ4	To what extent can COVID-19 provide a political window of opportunity for addressing the social and economic determinants of health, particularly related to gender and intersectionality	**30**	**3.33**	**0.84**	**3.28-3.39**
RQ9	How has the pandemic affected international donors and what have been the implications for gender and COVID-19 issues	**30**	**3.33**	**0.71**	**3.29-3.38**
RQ36	What data collaborative frameworks are needed to share responsibly and ethically COVID-19 sex and gender data between governments, academia and the private sector	**28**	**3.29**	**0.71**	**3.24-3.34**

RQ30	What kinds of oversight of non-state health providers (i.e. community-based and private providers) in service delivery is needed from a gender and COVID-19 perspective	**25**	**3.28**	**0.74**	**3.22-3.34**
RQ32	Has COVID-19 provided an opportunity to reform health research organisations, thereby providing more gender equitable ways of working	**27**	**3.22**	**0.80**	**3.16-3.28**
RQ21	Are there gendered impacts of having non-health officials, rather than health officials, lead pandemic responses such as COVID-19	**26**	**3.19**	**0.85**	**3.13-3.26**
RQ25	How have social movements advancing gender equality and intersectional justice been affected by COVID-19 and COVID-19 measures	**27**	**3.19**	**0.79**	**3.13-3.24**
RQ12	Who are the actors shaping COVID-19 health policies, and what is their affinity and/or accountability for addressing gender bias in pandemic responses	**30**	**3.18**	**0.75**	**3.13-3.23**
RQ8	What are the gender dimensions of how global neoliberalism combined with nationalist populist leadership has shaped policy responses to pharmaceutical companies and their decisions on vaccine patents, pricing and supply	**28**	**3.18**	**0.82**	**3.12-3.24**
RQ14	What best practices can be used for parliamentarians and decision makers in health and finance ministries to prioritize gender and COVID-19 issues	**30**	**3.17**	**0.75**	**3.12-3.22**
RQ27	What are the positive and negative gender implications of the emergency regulatory and legislative policy changes made due to COVID-19	**28**	**3.16**	**0.73**	**3.11-3.21**
RQ29	What is the gendered impact of the current patent and trade agreements on COVID-19 vaccines for disadvantaged populations	**25**	**3.16**	**0.80**	**3.10-3.22**
RQ15	What is the nature of the understanding of gender and COVID-19 issues within the media	**29**	**3.14**	**0.83**	**3.08-3.19**
RQ3	What are the gendered implications of different health system paradigms such as global health security or universal health care when responding to COVID-19	**32**	**3.13**	**0.79**	**3.08-3.17**
RQ2	How do values and political ideology influence how health systems respond to gendered COVID-19 needs	**33**	**3.09**	**0.88**	**3.04-3.14**
RQ19	What are the strengths and limitations of relying solely on "female stewardship" to implement a gender and rights-based approach to COVID-19	**26**	**3.02**	**0.75**	**2.96-3.08**
RQ35	What regulations are needed to allow open access to COVID-19 data	**27**	**3.02**	**0.77**	**2.96-3.07**
RQ34	What are the gendered elements of data privacy and protection to be considered in COVID-19 digital health tracking and surveillance measures	**27**	**3.00**	**0.83**	**2.94-3.06**
RQ17	How can we formulate and integrate gendered economic analyses to better address gender bias in decision-making for COVID-19	**28**	**2.96**	**0.84**	**2.91-3.02**
RQ26	What factors shape opportunities for other social movements to align and affirm gender concerns in pandemic responses	**27**	**2.91**	**0.78**	**2.85-2.96**
RQ20	What principles and strategies are most effective in supporting male allyship in advancing a gender and rights-based approach to COVID-19	**27**	**2.89**	**0.70**	**2.84-2.94**

RQ31	What strategies best redress the gendered nature of knowledge production, i.e. how those with family commitments during COVID-19 have not been able to contribute to knowledge production when compared to those without family commitments	**27**	**2.89**	**0.70**	**2.84-2.94**
RQ7	Does the gendered nature of corruption impact procurement of pandemic commodities and services, and if yes, how	**28**	**2.71**	**0.76**	**2.66-2.77**

Gender & Health Hub

Supplementary Table B: Sample size, Means, Standard deviations and 95% Confidence Intervals for Gender and COVID-19 Research Questions by Gender Equality (*18 out of 35 as statistically significant top research questions)

RQ	Label	n	Mean	SD	95% CI
*RQ11	To what extent, and how, is gender considered in the current decision making and learning processes for COVID-19	**30**	**3.30**	**0.79**	**3.25-3.35**
*RQ1	What do responsive and resilient health systems that address gender bias and advance gender equality look like	**34**	**3.29**	**0.80**	**3.25-3.34**
*RQ13	What capacities are needed to support decision makers, who may not be gender experts themselves, to enable responses to COVID-19 and future pandemic threats that address gender bias	**29**	**3.29**	**0.80**	**3.24-3.35**
*RQ33	How to best support national statistical systems to produce and use sex and gender data during COVID-19 and future pandemics	**29**	**3.28**	**0.84**	**3.22-3.33**
*RQ5	What is required to strengthen political will for investing in health systems that address gender bias and advance gender equality	**31**	**3.23**	**0.80**	**3.17-3.28**
*RQ24	Have vaccine deployment strategy plans included ways to consult and engage with gender experts, women's and high-risk marginalized groups	**28**	**3.21**	**0.83**	**3.16-3.27**
*RQ6	What forms of gendered abuse of power in health systems are made worse during pandemic health system responses and how can they best be addressed	**30**	**3.18**	**0.72**	**3.14-3.23**
*RQ4	To what extent can COVID-19 provide a political window of opportunity for addressing the social and economic determinants of health, particularly related to gender and intersectionality	**31**	**3.18**	**0.80**	**3.13-3.23**
*RQ27	What are the positive and negative gender implications of the emergency regulatory and legislative policy changes made due to COVID-19	**29**	**3.14**	**0.83**	**3.08-3.19**
*RQ28	What are the gender implications of COVID-19 response and recovery plans, the budgets allocated, and the resultant expenditures	**29**	**3.14**	**0.79**	**3.08-3.19**
*RQ18	What are the best ways of ensuring gender and intersectional balance and inclusion in decision-making bodies governing COVID-19 responses	**29**	**3.12**	**0.82**	**3.07-3.18**
*RQ23	What principles and strategies for linking marginalised communities with government health administrators and political decision makers best supports the advancement of gender and COVID-19 issues	**29**	**3.12**	**0.75**	**3.07-3.17**
*RQ12	Who are the actors shaping COVID-19 health policies, and what is their affinity and/or accountability for addressing gender bias in pandemic responses	**29**	**3.09**	**0.85**	**3.03-3.14**
*RQ32	Has COVID-19 provided an opportunity to reform health research organisations, thereby providing more gender equitable ways of working	**29**	**3.07**	**0.88**	**3.01-3.13**
*RQ36	What data collaborative frameworks are needed to share responsibly and ethically COVID-19 sex and gender data between governments, academia and the private sector	**29**	**3.07**	**0.88**	**3.01-3.13**

George AS, *et al. BMJ Global Health* 2023; 8:e011315. doi: 10.1136/bmjgh-2022-011315

Gender & Health Hub

*RQ3	What are the gendered implications of different health system paradigms such as global health security or universal health care when responding to COVID-19	**32**	**3.06**	**0.84**	**3.01-3.11**
*RQ16	What kind of evidence on gender and COVID-19 is needed for decision-makers, and does this vary by type of decision maker (government, donor organization, civil society etc.)	**29**	**3.05**	**0.97**	**2.99-3.12**
*RQ22	Which voices are heard more and why when involving citizens and community members in the planning and implementation of COVID-19 measures and what are the implications for gender and COVID-19 concerns	**29**	**3.03**	**0.91**	**2.97-3.10**
*RQ10	What approaches are most effective at successfully integrating different sectors into pandemic planning and response, and to ensuring that gender is centrally considered throughout in this multi-sectoral action	**30**	**3.03**	**0.85**	**2.98-3.09**
RQ17	How can we formulate and integrate gendered economic analyses to better address gender bias in decision-making for COVID-19	**29**	**3.02**	**0.89**	**2.96-3.08**
RQ25	How have social movements advancing gender equality and intersectional justice been affected by COVID-19 and COVID-19 measures	**29**	**3.00**	**0.80**	**2.95-3.05**
RQ9	How has the pandemic affected international donors and what have been the implications for gender and COVID-19 issues	**31**	**3.00**	**0.89**	**2.94-3.06**
RQ20	What principles and strategies are most effective in supporting male allyship in advancing a gender and rights-based approach to COVID-19	**29**	**2.97**	**0.68**	**2.92-3.01**
RQ2	How do values and political ideology influence how health systems respond to gendered COVID-19 needs	**34**	**2.94**	**0.81**	**2.89-2.99**
RQ30	What kinds of oversight of non-state health providers (i.e. community-based and private providers) in service delivery is needed from a gender and COVID-19 perspective	**29**	**2.93**	**1.07**	**2.86-3.00**
RQ14	What best practices can be used for parliamentarians and decision makers in health and finance ministries to prioritize gender and COVID-19 issues	**29**	**2.90**	**0.82**	**2.84-2.95**
RQ29	What is the gendered impact of the current patent and trade agreements on COVID-19 vaccines for disadvantaged populations	**27**	**2.89**	**0.89**	**2.82-2.05**
RQ19	What are the strengths and limitations of relying solely on "female stewardship" to implement a gender and rights-based approach to COVID-19	**29**	**2.88**	**0.84**	**2.82-2.94**
RQ35	What regulations are needed to allow open access to COVID-19 data	**29**	**2.88**	**0.82**	**2.82-2.93**
RQ26	What factors shape opportunities for other social movements to align and affirm gender concerns in pandemic responses	**29**	**2.83**	**0.85**	**2.77-2.88**
RQ34	What are the gendered elements of data privacy and protection to be considered in COVID-19 digital health tracking and surveillance measures	**29**	**2.83**	**0.93**	**2.76-2.89**
RQ15	What is the nature of the understanding of gender and COVID-19 issues within the media	**29**	**2.81**	**0.89**	**2.75-2.87**

George AS, *et al. BMJ Global Health* 2023; 8:e011315. doi: 10.1136/bmjgh-2022-011315

RQ8	What are the gender dimensions of how global neoliberalism combined with nationalist populist leadership has shaped policy responses to pharmaceutical companies and their decisions on vaccine patents, pricing and supply	**31**	**2.77**	**0.96**	**2.71-2.83**
RQ31	What strategies best redress the gendered nature of knowledge production, i.e. how those with family commitments during COVID-19 have not been able to contribute to knowledge production when compared to those without family commitments	**29**	**2.76**	**0.83**	**2.70-2.81**
RQ21	Are there gendered impacts of having non-health officials, rather than health officials, lead pandemic responses such as COVID-19	**29**	**2.60**	**0.94**	**2.54-2.67**
RQ7	Does the gendered nature of corruption impact procurement of pandemic commodities and services, and if yes, how	**30**	**2.48**	**0.79**	**2.43-2.54**

Supplementary Table C: Sample size, Means, Standard deviations and 95% Confidence Intervals for Gender and COVID-19 Research Questions by Urgency (*9 out of 35 as statistically significant top research questions)

RQ	Label	N	Mean	SD	95% CI
*RQ11	To what extent, and how, is gender considered in the current decision making and learning processes for COVID-19	**30**	**2.47**	**0.68**	**2.42-2.51**
*RQ12	Who are the actors shaping COVID-19 health policies, and what is their affinity and/or accountability for addressing gender bias in pandemic responses	**29**	**2.45**	**0.69**	**2.40-2.49**
*RQ29	What is the gendered impact of the current patent and trade agreements on COVID-19 vaccines for disadvantaged populations	**27**	**2.44**	**0.75**	**2.39-2.50**
*RQ6	What forms of gendered abuse of power in health systems are made worse during pandemic health system responses and how can they best be addressed	**30**	**2.43**	**0.68**	**2.39-2.48**
*RQ4	To what extent can COVID-19 provide a political window of opportunity for addressing the social and economic determinants of health, particularly related to gender and intersectionality	**31**	**2.40**	**0.76**	**2.36-2.45**
*RQ28	What are the gender implications of COVID-19 response and recovery plans, the budgets allocated, and the resultant expenditures	**29**	**2.38**	**0.62**	**2.34-2.42**
*RQ9	How has the pandemic affected international donors and what have been the implications for gender and COVID-19 issues	**30**	**2.37**	**0.72**	**2.32-2.41**
*RQ16	What kind of evidence on gender and COVID-19 is needed for decision-makers, and does this vary by type of decision maker (government, donor organization, civil society etc.)	**28**	**2.29**	**0.71**	**2.24-2.34**
*RQ24	Have vaccine deployment strategy plans included ways to consult and engage with gender experts, women's and high-risk marginalized groups	**28**	**2.25**	**0.84**	**2.19-2.31**
RQ18	What are the best ways of ensuring gender and intersectional balance and inclusion in decision-making bodies governing COVID-19 responses	**29**	**2.21**	**0.73**	**2.16-2.26**

Gender & Health Hub

RQ22	Which voices are heard more and why when involving citizens and community members in the planning and implementation of COVID-19 measures and what are the implications for gender and COVID-19 concerns	**29**	**2.21**	**0.82**	**2.15-2.26**
RQ8	What are the gender dimensions of how global neoliberalism combined with nationalist populist leadership has shaped policy responses to pharmaceutical companies and their decisions on vaccine patents, pricing and supply	**28**	**2.20**	**0.82**	**2.14-2.25**
RQ13	What capacities are needed to support decision makers, who may not be gender experts themselves, to enable responses to COVID-19 and future pandemic threats that address gender bias	**28**	**2.18**	**0.77**	**2.12-2.23**
RQ27	What are the positive and negative gender implications of the emergency regulatory and legislative policy changes made due to COVID-19	**29**	**2.17**	**0.76**	**2.12-2.22**
RQ23	What principles and strategies for linking marginalised communities with government health administrators and political decision makers best supports the advancement of gender and COVID-19 issues	**29**	**2.16**	**0.77**	**2.10-2.21**
RQ10	What approaches are most effective at successfully integrating different sectors into pandemic planning and response, and to ensuring that gender is centrally considered throughout in this multi-sectoral action	**30**	**2.15**	**0.68**	**2.11-2.19**
RQ15	What is the nature of the understanding of gender and COVID-19 issues within the media	**28**	**2.14**	**0.80**	**2.09-2.20**
RQ5	What is required to strengthen political will for investing in health systems that address gender bias and advance gender equality	**31**	**2.13**	**0.76**	**2.08-2.18**
RQ30	What kinds of oversight of non-state health providers (i.e. community-based and private providers) in service delivery is needed from a gender and COVID-19 perspective	**28**	**2.11**	**0.69**	**2.06-2.16**
RQ14	What best practices can be used for parliamentarians and decision makers in health and finance ministries to prioritize gender and COVID-19 issues	**29**	**2.10**	**0.77**	**2.05-2.16**
RQ1	What do responsive and resilient health systems that address gender bias and advance gender equality look like	**34**	**2.07**	**0.74**	**2.03-2.12**
RQ19	What are the strengths and limitations of relying solely on "female stewardship" to implement a gender and rights-based approach to COVID-19	**28**	**2.07**	**0.77**	**2.02-2.13**
RQ25	How have social movements advancing gender equality and intersectional justice been affected by COVID-19 and COVID-19 measures	**28**	**2.07**	**0.72**	**2.02-2.12**
RQ33	How to best support national statistical systems to produce and use sex and gender data during COVID-19 and future pandemics	**29**	**2.07**	**0.84**	**2.01-2.13**
RQ3	What are the gendered implications of different health system paradigms such as global health security or universal health care when responding to COVID-19	**31**	**2.06**	**0.85**	**2.01-2.12**
RQ35	What regulations are needed to allow open access to COVID-19 data	**28**	**2.02**	**0.73**	**1.97-2.07**

George AS, *et al. BMJ Global Health* 2023; 8:e011315. doi: 10.1136/bmjgh-2022-011315

Gender & Health Hub

RQ17	How can we formulate and integrate gendered economic analyses to better address gender bias in decision-making for COVID-19	**29**	**2.00**	**0.85**	**1.94-2.06**
RQ21	Are there gendered impacts of having non-health officials, rather than health officials, lead pandemic responses such as COVID-19	**29**	**2.00**	**0.76**	**1.95-2.05**
RQ31	What strategies best redress the gendered nature of knowledge production, i.e. how those with family commitments during COVID-19 have not been able to contribute to knowledge production when compared to those without family commitments	**27**	**2.00**	**0.73**	**1.95-2.05**
RQ36	What data collaborative frameworks are needed to share responsibly and ethically COVID-19 sex and gender data between governments, academia and the private sector	**29**	**1.97**	**0.78**	**1.91-2.02**
RQ7	Does the gendered nature of corruption impact procurement of pandemic commodities and services, and if yes, how	**29**	**1.97**	**0.78**	**1.91-2.02**
RQ2	How do values and political ideology influence how health systems respond to gendered COVID-19 needs	**34**	**1.93**	**0.82**	**1.88-1.97**
RQ20	What principles and strategies are most effective in supporting male allyship in advancing a gender and rights-based approach to COVID-19	**29**	**1.91**	**0.73**	**1.86-1.96**
RQ34	What are the gendered elements of data privacy and protection to be considered in COVID-19 digital health tracking and surveillance measures	**28**	**1.91**	**0.79**	**1.86-1.97**
RQ32	Has COVID-19 provided an opportunity to reform health research organisations, thereby providing more gender equitable ways of working	**29**	**1.86**	**0.64**	**1.82-1.91**
RQ26	What factors shape opportunities for other social movements to align and affirm gender concerns in pandemic responses	**29**	**1.69**	**0.76**	**1.64-1.74**
