# Supplementary material for: A shared agenda for gender and COVID-19 research: priorities based on broadening engagement in science
Source: BMJ Glob Health. 2023 May 22;8(5):e011315. doi: 10.1136/bmjgh-2022-011315 (PMC10230361; doi:10.1136/bmjgh-2022-011315)
Supplement: Supplementary data [file bmjgh-2022-011315supp008.pdf]

Gender and COVID-19 Research Prioritisation Survey Analysis

The analysis was guided by the following research questions:

- What are the high-priority research questions per theme? Do these research questions vary across the three criteria?
- What are the top-priority research questions across all themes as per the three criteria?
- Are there any differences in how respondent groups score top-priority questions?

The analysis was done inUsing R Studio Pro through PositCloud, a platform to write code collaboratively, the language-specific datasets were merged and processed. The version used for RStudio Pro was 2022.02.0 and for R was 4.2.2 The language-specific datasets were merged and processed. The responses received from the unique invitations and responses through the general "open" link were analysed jointly. The data was explored through frequency tables, histograms/bar graphs, and descriptive statistics (e.g., means, standard deviations). Further statistical analysis as detailed below involved comparison of means to identify high-priority questions within each theme, top-priority questions across all themes, and differences in the scores of top-priority questions by respondent type.

To identify high-priority questions within each theme, we calculated the means and standard deviations of research questions for each criterion and corresponding confidence intervals. The mean was chosen as the central tendency statistics due to the scale type (intervals). The research questions were listed by descending order of means from the most priority to the least priority for public health, gender equality and urgency criteria respectively. To select the high priority questions, we established a cut-off point statistically for each criteria usedcriterion. Starting with the research question with the highest criteria- specific mean, we tested – through a paired samples t-test – whether the mean for that question was significantly different from the mean of the subsequent question. This analysis was done for each If the difference between the means was not significantly different at the 0.05 level of significance, we retained both research questions in the high priority group. This analysis was done specifically for each criteria. We continued until a significant difference was found. The cut-off point was set at that juncture, resulting in the identification of high priority questions whose criteria specific means were above this juncture.

To identify the top priority research questions across themes, twe took the high-priority questions that emerged within each theme and centered their means to ensure that the means on the public health, gender equality and urgency scales were the same across themes. Since there were different respondents for different themes, this process canceled differences in response styles that may otherwise distort comparisons across themes.

The centered means of these high-priority research questions (n=89) with scoresscore above the cut-off points for all three criteria were plotted in a scatterplot with its axes contrasting mean scores for public health benefit and gender equality. All these high-priority research questions scored the highest in urgency and therefore they are short to medium-term urgent. The objective was to identify questions that scored the highest in allboth criteria simultaneously and placed at the top right quadrant in the scatterplot (n=25). To determine cut-off points for the top-priority research questions, we followed the same procedure with the paired samples t-test as done previously for the thematic groups focusing on the 25 research questions in the top right quadrant.

As the last step, we ran chi-squared tests and multivariate analysis of variance (MANOVA) to analyse the association between respondent types (gender, organisational affiliation and country income groups) and top-priority research questions. The de-identifed data from survey responses are

available on GitHub. The code for analysis will also be publicly available on GitHub and in Markdown format in RPubs.

Survey participants

With regards to the thematic surveys, 224 responses were received from 173 participants, as some participants were free to choose which thematic questionnaire they would respond to and some responded to more than one thematic questionnaire. Participation was defined as responding to at least 10 percent of the questions for at least one theme. The English version of the questionnaire was answered by 158 participants and the translated versions by 6 participants in Spanish, 5 in French and 4 in Portuguese.

Survey participants were largely from LMICs (63%), women (72.2%), and based in universities (40.5%) and NGOs (23.1%). Nonetheless, substantial proportions of participants also included men (27.2%) and those from governments, donors and multilateral organizations (22.0%). Participation across WHO regions was relatively even, except for the Eastern and Mediterranean region despite a regional webinar in English and Arabic with 57 participants (Table 2). When examining the gender profile of respondents by other social categories, the participation of men was proportionally higher in lower income countries. Men also participated more than women in the Western Pacific region, but the reverse was observed for the other WHO regions. (Supplemental table 1).

Table 2: Gender, organizational base, country of residence, World Bank income group of the country, and WHO region of 173 gender and COVID-19 research prioritization survey participants.

Variable	Percentage	Number
**Gender**		
He/Him/His	27.2	47
She/Her/Hers	72.2	125
Other	0.6	1
**Organizational Base**		
Donor/Govt/Multilateral	22.0	38
Independent	14.5	25
NGO	23.1	40
University	40.5	70
**Country Income**		
High income	37.0	64
Upper middle income	23.7	55
Lower middle income	31.8	41
Low income	7.5	13
**WHO Regions**		

WHO AFRO	22.0	38
WHO AMRO	20.8	36
WHO EMRO	4.6	8
WHO EURO	16.2	28
WHO SEARO	19.1	33
WHO WPRO	17.3	30
**Total**	**100**	**173**

Survey responses

The analysis of the research questions showed that the distributions of mean scores are negatively skewed for all thematic groups, reflecting halo effect (40,41) in the response styles, with respondents tending to answer scales in the same direction. Therefore, the means are close to the high end of the three scales: above 3.2 for public health, 3.0 for gender and above 2.1 for urgency. The standard deviations are small, denoting that the mean scores do not vary much among research questions within themes. The total high-priority research questions (ie with mean scores above the cut-off point) retained was 89 from the original set of 214 which corresponds to 42% of the questions (Table 3, Supplemental tables 2-6), highlighting that prioritization was not an easy task. The mean scores for three scales and sample sizes for each of the thematic groups are depicted in Table 3.

Table 3. Means and standard deviations of scores for research questions and sample size per thematic group in the gender and COVID-19 research prioritization surveys

Thematic group	Total respondents	Total research questions	Means (standard deviations)			Total hHigh priority research questions	Percentile for the cut-off point
			Public health benefit (1: very low to 4: very high)	Gender equality (1: not likely to 4: highly likely)	Urgency (1: long to 3: short term)		
Health status & behavior	39	40	3.47 (0.15)	3.17 (0.18)	2.22 (0.20)	16	40
Research and Development	28	41	3.35 (0.23)	3.07 (0.30)	2.19 (0.24)	18	44
Health services	41	54	3.37 (0.17)	3.16 (0.20)	2.11 (0.16)	18	33
Social determinants	81	43	3.28 (0.16)	3.14 (0.21)	2.12 (0.13)	16	37
Governance	35	36	3.23 (0.21)	3.01 (0.19)	2.13 (0.18)	21	58

Total	224	214	-	-	-	89	42

For each theme, between 16 and 21 research questions made the cut-off point identifying them as high-priority for public health and/or gender equality and/or urgency. The total high-priority research questions retained was 89 from the original set of 214 which corresponds to 42% of the questions (Supplemental tables 2-6). This result reinforces the fact that the mean scores for research questions are very similar and the prioritization process was not an easy task.

Top priority research questions

The 89 high-priority research questions within each theme were plotted in a scatter plot according to their centered means for public health benefit and gender equality. All these questions scored the highest in urgency and therefore are short to mid-term urgent (cf Table 4).

We took the 25 high- priority research questions in the top right quadrant (defined by the median for the respective scale) and used paired samples t-tests to yield 21 top priority research questions across all themes (Table 4). Top priority research questions are questions that score the highest in all three criteria. While the 21 top priority questions included questions from all themes, the numbers of top priority research questions per theme varied by descending order: research and development (7); health service delivery (6); health status and behavior (5); social determinants (4) and governance (3). Across all questions, there is a general trend for the public health criteria scores to be higher than gender equality scores, though in many cases the differences are too small and/or not statistically significant. Research question 40 from the research and development theme was ranked the highest across all questions: *How can pregnant and lactating females be ethically, and safely included in phase 3 and 4 studies for COVID-19 R&D*?

Comparisons among demographic groups

The MANOVA results showed that only three questions presented differences in the scores by gender, organisational base or income groups. The follow-up test (Tukey HSD) showed the groups that differ significantly. Two questions from the theme on social determinants related to gender-based violence were given higher scores by women compared to men, and a question on gender mainstreaming from the theme on governance was scored higher by middle income country participants than low income country participants (Supplementary Table 7).
